# Supplementary material for: Cost-effectiveness of habit-based advice for weight control versus usual care in general practice in the Ten Top Tips (10TT) trial: economic evaluation based on a randomised controlled trial
Source: BMJ Open. 2018 Aug 13;8(8):e017511. doi: 10.1136/bmjopen-2017-017511 (PMC6091904; doi:10.1136/bmjopen-2017-017511)
Supplement: Author's manuscript [file bmjopen-2017-017511.draft_revisions.pdf]

BMJ Open

BMJ Open is committed to open peer review. As part of this commitment we make the peer review history of every article we publish publicly available.

When an article is published we post the peer reviewers' comments and the authors' responses online. We also post the versions of the paper that were used during peer review. These are the versions that the peer review comments apply to.

The versions of the paper that follow are the versions that were submitted during the peer review process. They are not the versions of record or the final published versions. They should not be cited or distributed as the published version of this manuscript.

BMJ Open is an open access journal and the full, final, typeset and author-corrected version of record of the manuscript is available on our site with no access controls, subscription charges or pay-per-view fees (http://bmjopen.bmj.com).

If you have any questions on BMJ Open's open peer review process please email info.bmjopen@bmj.com

BMJ Open

Cost-effectiveness of habit-based advice for weight control versus usual care in general practice in the Ten Top Tips (10TT) Trial: economic evaluation based on a randomised controlled trial

Journal:	*BMJ Open*
Manuscript ID	bmjopen-2017-017511
Article Type:	Research
Date Submitted by the Author:	04-May-2017
Complete List of Authors:	Patel, Nishma; UCL, Department of Applied Health Research<br>Beeken, Rebecca; University College London, Dept. of Epidemiol<br>Leurent, Baptiste; London School of Hygiene and Tropical Medicine, Department of Infectious Disease Epidemiology<br>Omar, Rumana; University College London,<br>Nazareth, Irwin; UCL, Primary Care and Population Health<br>Morris, Stephen; University College London, Department of Applied Health Research
**Primary Subject Heading**:	Health economics
Secondary Subject Heading:	Public health, General practice / Family practice
Keywords:	HEALTH ECONOMICS, PRIMARY CARE, PUBLIC HEALTH

SCHOLARONE™ Manuscripts

For peer review only

Cost-effectiveness of habit-based advice for weight control versus usual care in general practice in the Ten Top Tips (10TT) Trial: economic evaluation based on a randomised controlled trial

Nishma Patel[1], Rebecca J. Beeken[2], Baptiste Leurent[3], Rumana Z. Omar[4], Irwin Nazareth[2] and Stephen Morris[1]

[1] Department of Applied Health Research, University College London, Gower Street, London, WC1E 6BT, UK

[2] Department of Behavioural Science & Health, University College London, Gower Street, London, WC1E 6BT, UK

[3] London School of Hygiene and Tropical Medicine, Keppel Street, WC1E 7HT

[4] Department of Statistical Science, University College London, Gower Street, London, WC1E 6BT, UK

Correspondence to: Nishma Patel, Department of Applied Health Research, University College London WC1E 6BT, UK. Telephone: +44 2031 083027 (email: nishma.patel@ucl.ac.uk)

For peer review only - http://bmjopen.bmj.com/site/about/guidelines.xhtml
For peer review only

Abstract

Objective: Ten Top Tips (10TT) is a primary care led behavioural intervention, which aims to help adults reduce and manage their weight by following ten weight loss tips. The intervention promotes habit formation to encourage long-term behavioural changes. The aim of this study was to estimate the cost-effectiveness of 10TT in general practice from the perspective of the UK National Health Service.

Design: An economic evaluation was conducted alongside an individually randomised controlled trial.

Setting: 14 General Practitioner (GP) practices in England.

Participants: All patients were aged ≥18 years, with BMI ≥30kg/m^2. A total of 537 patients were recruited; 270 received the usual care offered by their practices and 267 received the 10TT intervention.

Outcomes measures: Health service use and quality-adjusted life years (QALY) were measured over two years. Analysis was conducted in terms of incremental Net Monetary Benefit (NMB), using non-parametric bootstrapping and multiple imputation.

Results: Over a two-year time horizon, mean costs and QALYs per patient in the 10TT group were £1889 (95% CI: £1522 to £2566) and 1.51 (95% CI: 1.44 to 1.58). The mean costs and QALYs for usual care were £1925 (95% CI: £1599 to £2251) and 1.51 (95% CI: 1.45 to 1.57), respectively. This generated a mean cost difference of -£36 (95%CI: -£511 to £440) and a mean QALY difference of 0.0007 (95%CI: -0.080 to 0.082). The incremental NMB for 10TT versus usual care was £49 (95%CI: -£1709 to £1800) at a maximum willingness to pay for a QALY of £20,000. 10TT had a 52% probability of being cost-effective at this threshold.

For peer review only - http://bmjopen.bmj.com/site/about/guidelines.xhtml

For peer review only

Conclusions: Costs and QALYs for 10TT were not significantly different from usual care and therefore 10TT is as cost-effective as usual care. There was no evidence to recommend nor advice against offering 10TT to obese patients in general practices based on cost-effectiveness considerations.

Trial Registration: ISRCTN registry ISRCTN16347068

Article summary

Strengths and limitations of this study

- The analysis is based on a large multicentre randomised trial with detailed information on resource use and utility values for a median follow-up period of two years.
- Individual data on standard weight-loss interventions received by the participant were not recorded.
- The analysis took a UK NHS/PSS perspective but a wider perspective (e.g., societal) could have been taken, including costs to patients and families.
- The time horizon was two years but could have taken a longer time horizon.
- Missing NHS resource use and utility data was accounted for using multiple imputation.

For peer review only - http://bmjopen.bmj.com/site/about/guidelines.xhtml

For peer review only

Introduction

The worldwide prevalence of obesity nearly doubled between 1980 and 2008. Based on recent estimates in the European Union, overweight affects 30-70% and obesity affects 10-30% of adults(1). For both women and men aged 18 years and over, the lowest proportions of the population considered to be obese in 2008 were observed in Romania (8.0 % for women and 7.6 % for men), Italy (9.3 % and 11.3 %), Bulgaria (11.3 % and 11.6 %) and France (12.7 % and 11.7 %). The highest proportions of women who were obese were recorded in the UK (23.9 %), Malta (21.1 %), Latvia (20.9 %) and Estonia (20.5 %); and of men in Malta (24.7 %), the UK (22.1 %) and Hungary (21.4 %)(2).

Obesity is a costly threat to health and places a huge financial burden on health services. Health risks associated with obesity range from heart disease, stroke, and type 2 diabetes to gall bladder disease. It is estimated that health problems associated with being overweight or obese cost the National Health Service (NHS) in England more than £5 billion every year(3). As the prevalence of obesity continues to rise(4), the financial burdens are also likely to increase.

Non-surgical interventions for weight loss are available and include dietary advice, physical activity and behaviour modification(5). These can be accessed mainly through primary care. Despite considerable investment in such interventions, there is little evidence about their cost-effectiveness(5). Fuller et al(6) evaluated the cost-effectiveness of standard care versus a commercial weight loss programme (Weight Watchers (CP)) in a population of 772 participants from Australia, UK and Germany, over a timeframe of one year. The cost per kilogram of weight loss was US$122 (Australia), US$90 (UK) and US$180 (Germany) for the commercial provider and US$138 (Australia), US$151 (UK) and US$133 (Germany) for

For peer review only

For peer review only - http://bmjopen.bmj.com/site/about/guidelines.xhtml

standard care. The incremental cost per QALY gained of the commercial programme versus standard care was US$18,266 (Australia), US$12,100 (UK) and US$40,933 (Germany). A further collection of follow-up data at two-years was used to develop a decision analytic model to estimate the lifetime costs and outcomes in the Australian population. Over a lifetime of the patient, the commercial weight loss programme resulted in an incremental cost saving of US$47 per patient(7), suggesting that the commercial programme may be cost effective(8).

An evaluation of obesity treatment in Pennsylvania (USA), with 390 participants looked at the cost-effectiveness of usual care (quarterly visits to the primary care provider), lifestyle counselling (quarterly visits to the primary care provider plus monthly weight loss counselling) and enhanced lifestyle counselling (all the above plus meal replacements and weight loss medication). The study showed the incremental cost per kilogram lost was US$292 for the enhanced lifestyle counselling compared to usual care. Long-term estimates using published data and trial data suggested Brief Lifestyle Counselling would fall somewhere between US$7000 and US$12600 cost per QALY; Enhanced Brief lifestyle counselling the cost per kilogram could be as high as US$22000(9).

The cost-effectiveness of the Counterweight intervention in the UK (a nurse-delivered patient education programme) showed that the intervention was dominant (less costly and more effective) compared with no active intervention; it produced a gain in QALYs (0.06 per participant) and cost savings of £27 per participant (10).

For overweight and obese women, a three component intervention of diet, exercise, and behaviour change in the USA produced a cost per QALY gained of US$12,600 compared with

For peer review only

For peer review only - http://bmjopen.bmj.com/site/about/guidelines.xhtml

usual care, exercise, and diet alone(5). The authors concluded the three-component programme was good value for money.

Ten Top Tips (10TT) is a simple leaflet-based intervention for patients in primary care designed to help with weight loss. The underpinning theory behind 10TT is habit formation. Psychological research shows that repetition of a simple action in a consistent context leads, through associative learning, to the action being activated upon subsequent exposure to those contextual cues(11). A small pilot trial consisting of 100 people showed that those who received the Ten Top Tips leaflet lost significantly more weight than those who did not receive it(12). Although the absolute weight loss was modest, the intervention had the potential to be disseminated at minimal cost to large numbers simply by making the leaflet available. In response to this a large randomised trial was undertaken. Participants were given a leaflet, which provided weight loss tips based on scientific evidence on how to turn the tips into healthy habits, during a 30-minute appointment with a practice nurse. The ten healthy tips included: keeping to meal routines; reduced fat intake; walking off weight; packing a health snack; looking at food labels; taking caution with portion size; standing up; thinking about drinks; focusing on food; and, not forgetting ‘five a day’. Participants were asked to record their progress in a logbook. Using routinely collected data from primary care practices, the aim of the present study was to analyse the cost and cost-effectiveness of 10TT.

Methods

Trial background

For peer review only - http://bmjopen.bmj.com/site/about/guidelines.xhtml

The 10TT trial was a two-arm, individually randomised, controlled trial in which 537 obese men and women were enrolled. Practices across England (n=14) were recruited through the MRC General Practice Research Framework (GPRF). They were located in: Wellingborough, Southampton, Bradford-on-Avon, Bromsgrove, Frome, Guisborough, Glastonbury, Ivybridge, Dunstable, Liskeard, Ledbury, New Mills, and London and recruitment occurred between August 2010 and October 2011. Participants were randomly assigned to 10TT (n=267) or usual care (n=270), and followed for up to two years. The primary outcome was weight loss at three months. Secondary outcomes included BMI, waist circumference, the number of people achieving a 5% reduction in weight, clinical markers for potential co-morbidities (blood pressure, total cholesterol/low-density lipoprotein and blood glucose), and maintenance of weight loss over 24 months. At three months participants in the 10TT group lost significantly more weight than those receiving usual care (mean difference in weight change=-0·87kg, 95%CI -1·47 to -0·27 p=0·004). There was no difference in weight change between the two groups at 24 months; while weight loss in the usual care group was slower in the first 6 months, it continued until 18 months, whereas the 10TT group experienced a greater weight loss in the first 6 months, but did not lose any additional weight after this point.

Overview of economic evaluation

We undertook a cost-utility analysis to compare the costs and outcomes associated with 10TT and usual care. The outcome measure was quality-adjusted life years (QALYs), which combines length of life and quality of life, and is consistent with the National Institute for Health and Care Excellence (NICE) recommendations(13).The primary outcomes were the incremental costs and effects and the incremental net monetary benefit (NMB) of 10TT

For peer review only

versus usual care. Costs were measured from a NHS and personal social services (PSS) perspective; since PSS costs were negligible, we focused on NHS costs. These were calculated in 2013/14 UK£. The time horizon for costs and effects was two years, reflecting the trial follow-up period. Extrapolation beyond this time period was not undertaken because the within-trial analysis found no evidence of significant differences in costs or outcomes between groups; two years was considered long enough to reflect all important differences in costs or outcomes between treatments. Costs and outcomes in the second year were discounted at 3.5%.

Resource use and costs

Resource use was recorded for two years prior to randomisation on: the number and type of contacts with the GP (surgery visit, home visit, phone call); practice nurse contacts (practice visit, home visit, phone call); dietician visits; hospital outpatient visits; hospital inpatient stay; accident and emergency (A&E) visits; and outpatient services. These data were retrieved from GP records. Data on use of drugs included dose, type and frequency of administration. All resource use data was extracted by the practice nurse at participating centres from patient records.

Unit costs were attached to each resource item. The cost per visit to primary care (GP surgery visit, GP home visit, GP phone call, practice nurse visit, nurse home visit, practice nurse phone call and dietician visits were taken from Personal Social Services Research Unit (PSSRU) estimates(14). The cost of inpatient episodes, outpatient visits and A&E visits were taken from the PSSRU and based on NHS Reference Costs(15). They included cost of medical staff, equipment, consumables, and diagnostics. The weighted average cost of inpatient

For peer review only - http://bmjopen.bmj.com/site/about/guidelines.xhtml

stays was calculated combining the cost of inpatient long and short stay. Drug costs were obtained from British National Formulary(16).

The cost of the 10TT intervention included the following, which were valued using market prices: a logbook (£2.80); time patients spent with the practice nurse to introduce the programme (£20 for a 30-minute visit); wallet sized food label guidance (£0.09), and a 10TT leaflet (£0.05). The total cost of 10TT was £22.94 per participant. The cost of the food label guidance was based on the total cost of printing (£1,392) divided by the number of labels printed (15,000). Similarly, the cost of 10TT leaflets was based on a total cost of printing (£1,540) divided by the number of leaflets printed (30,000).

All practices offered standard weight-loss interventions as part of usual care. Standard weight loss interventions consisted of a range of interventions including referrals to community programmes, gym prescription and referral to a dietician and/or psychologist, among others. While data were provided by practices on interventions typically offered to their patient, the uptake of these interventions was not recorded. In our base case, we made the conservative assumption (possibly biased against 10TT) that no patients received these interventions. In a sensitivity analysis, we included the costs of these interventions assuming each patient in the control group received the standard weight-loss intervention offered by the practice. Where single interventions were offered by a practice these were costed accordingly, based on the average cost of participating in Weight Watchers (assumed to cost £78.22 per patient(17)) or Slimming World (£72.62 per patient). Where multiple interventions were offered by a practice these were costed based on the average cost of participating in the Size Down Programme at a cost of £93.48 per patient(17).

For peer review only - http://bmjopen.bmj.com/site/about/guidelines.xhtml

The unit costs for each cost component are shown in Table 2. Unit costs were multiplied by resource volume and summed across all cost components to obtain a total cost per patient.

Utilities and QALYs

Health utilities were based on the EQ-5D-3L descriptive system (18, 19). This is a five dimensional questionnaire (mobility, self-care, usual activities, pain and discomfort), with three levels in each dimension (severe problems, some problems, no problems). Each EQ-5D state was converted into a single utility score based on valuations from the UK general population (18, 20). Utility values of one represent full health, values of zero are equivalent to death, negative values represent states worse than death. The EQ-5D questionnaire was completed at baseline, three months, six months, 12 months, 18 months and 24 months. A utility profile was constructed for participants assuming a straight-line relation between their utility values at each measurement point. QALYs for every patient from baseline to two years were calculated as the area under the utility profile.

Dealing with Missing Data

There were missing data for NHS resource use and utility scores. Multiple imputation by multivariate normal regression used to impute missing data (21, 22). The imputation method used an iterative Markov Chain Monte Carlo (MCMC) technique to simulate from the posterior predictive distribution of missing data, generating 20 imputed datasets(23). We imputed missing data for the following variables: weight; BMI; waist circumference; EQ-5D at baseline, 3 months, 6 months, 12 months, 24 months, NHS visits; GP, nurse, dietician, hospital inpatient stay, outpatient clinic, A&E visits and all other health care service visits (Table 2 and 3). Age, sex and study centre were included in the imputation model as

For peer review only - http://bmjopen.bmj.com/site/about/guidelines.xhtml

additional explanatory variables. Imputations were undertaken using the –mi impute mvn (24)– command in Stata SE version 13 (20).

Statistical analysis

Two sample t-tests were carried out for complete data. A linear regression model for imputed data was used to test for differences in mean resource use, costs, EQ-5D scores at each time point, and QALYs. Non-parametric bootstrapping was used to calculate the difference in mean costs, QALYs and incremental NMBs with confidence intervals between groups. The incremental NMBs were calculated as the difference in mean QALYs per participant (Q) with 10TT(T) versus usual care(U) multiplied by the maximum willingness to pay for a QALY (R) minus the difference in mean cost per participant (C), i.e., incremental NMB = $(Q_T - Q_U)*R - (C_T - C_U)$. We used the cost-effectiveness threshold range recommended by NICE (£20 000 to £30 000 (12)) as the lower and upper limits of the maximum willingness to pay for a QALY (R). Negative incremental NMBs indicate that usual care is preferred on cost-effectiveness grounds and positive incremental NMBs indicate that 10TT is preferred (21).

QALYs gained were initially adjusted for age, gender, practice and baseline utility values using regression analysis (ordinary least squares); incremental costs were initially adjusted for age, gender, practice and prior costs in the previous two years (generalised linear model with gamma family and log link to account for skewness). Adjusting for these factors did not make much difference to the results (Table 1) and were therefore not included in the final model.

For peer review only - http://bmjopen.bmj.com/site/about/guidelines.xhtml

For each of the 20 imputed datasets we ran 1000 bootstrap replications using non-parametric bootstrapping, resampling observations with replacement. The bootstrapped results were combined using the formula described by Briggs et al (22), to calculate standard errors around mean values allowing for uncertainty around the imputed values, skewness of cost and utility data, and sampling variation. Standard errors were used to calculate 95% CIs around point estimates.

The probability that 10TT was cost-effective in comparison to usual care at different values of the cost-effectiveness threshold was presented in form of a cost-effectiveness acceptability curve (CEAC)(25). The CEAC was based on the proportion of bootstrap replications across all twenty imputed datasets that were below the cost-effectiveness threshold, which was varied from £0 to £50,000.

Several deterministic sensitivity analyses were carried out to assess the uncertainty around key components of the analysis. An analysis was undertaken based on complete cases without any imputation. Cost components varied were primary and secondary care costs and drug costs. The total costs of each component were amended by ±10%. We also conducted analyses including the cost of standard weight loss interventions for usual care only.

Results

Baseline description

Table 4 illustrates the mean costs, two years prior to the trial. The mean cost for the usual care group was £1848 (95% CI: 1615, 2082), and £2052 (95% CI: 1756, 2349) in the 10TT

For peer review only - http://bmjopen.bmj.com/site/about/guidelines.xhtml

group. Baseline characteristics- age, gender, weight, BMI blood pressure and cholesterol did not differ between groups(26).

Base case

Table 2 shows mean imputed resource use by treatment group. The intervention and control groups had the same mean GP practice visits per participant over the two-year period (9.4 versus 9.4), as well as similar GP home visits (0 versus 0.1), GP phone contacts (1.3 versus 1.3), nurse practice visits (6.6 versus 6.9), nurse home visits (0 versus 0.1), inpatient episodes (0.2 versus 0.2), outpatient clinic visits (2.0 versus 2.1) and accident and emergency visits (0.4 versus 0.4). There was no statistically significant difference between groups for any category of health service resource use.

Table 4 shows the total cost per group. The non-parametric bootstrapping including imputed resource use produced mean total costs per participant in the intervention group of £1889 (95% CI: £1522 to £2566), compared to £1925 (95% CI: £1599 to £2251) in the control group. The mean difference in cost between 10TT and control group was-£36 (95%CI: -£511 to £440), which was not statistically significant (p=0.88) (Table 5).

Undertaking non-parametric bootstrapping after multiple imputation produced 1.51 (95% CI: 1.44 to 1.57) QALYs in the intervention group and 1.51 (95% CI: 1.45 to 1.57) in the control group. Generating a mean difference in QALYs of 0.0007 (95%CI: -0.080 to 0.082) (p=0.85) (Table 5) which was not statistically significant. Hence, patients receiving 10TT accrued non-significantly lower costs and the difference in QALYs between the groups was very small.

For peer review only - http://bmjopen.bmj.com/site/about/guidelines.xhtml

The incremental net monetary benefit for 10TT versus usual care was £49 (95% CI: -£1709 to £1800) at a maximum willingness to pay for a QALY of £20,000 and £55 (95% CI: -2489, 2583) at a maximum willingness to pay for a QALY of £30,000 (Table 5).

Of the 20,000 bootstrap replications produced under base case assumptions, 24% fell into south-west quadrant of the cost-effectiveness plane (10TT was less costly and less effective than usual care); 20% fell into the north-east quadrant (10TT was more costly and more effective than usual care); 25% fell into the north-west quadrant (10TT was more costly and less effective than usual care); and 31% fell into the south-east quadrant (10TT was less costly and more effective than usual care) (Figure 1). The CEAC derived from Figure 1 is in Figure 2. Under base case assumptions, 10TT has a 52% probability of being cost-effective at a willingness to pay threshold of £20000 and 52% at a willingness to pay threshold of £30000 (Table 5).

Sensitivity analyses

Including the cost of the standard weight-loss interventions (£87) in the usual care group the mean costs per patient in the control group increased to £2012, which was higher than the mean cost in the intervention group (£1889). The mean incremental cost difference between groups was -£122 (95% CI: -£598 to £353). The incremental net monetary benefit for 10TT versus usual care was £140 (95% CI: (-£1666 to £1902) at a maximum willingness to pay for a QALY of £20,000 and £148 (95% CI: -£2463 to £2693) at a maximum willingness to pay of £30,000. Increasing and decreasing costs by 10% did not affect the findings appreciably (Table 5).

A complete case analysis was undertaken using 163 of 537 participants with complete utility and cost data. The incremental NMB was -£877 (95% CI: -£2338 to £3404) at a maximum

For peer review only - http://bmjopen.bmj.com/site/about/guidelines.xhtml

willingness to pay for a QALY of £20,000 and -£1349 (95% CI: -£3269 to £4972) at a maximum willingness to pay for a QALY of £30,000.

Discussion

Our economic analysis of 10TT showed that this intervention had similar costs and QALYs as usual care. Sensitivity analyses showed little uncertainty in this finding. On the one hand the findings mean there is no reason to prefer 10TT or usual care on the basis of differences in quality of life or cost, or on cost-effectiveness grounds. On the other hand, this means that 10TT is as cost-effective as usual care.

Previous studies have reported the cost-effectiveness of primary care led programmes such as lifestyle counselling(9), commercial weight loss programmes(6, 27), patient education(10) and the combination of diet and exercise(5) over 12 months to 24 month period, compared with usual care. For example, Trueman et al(10) found small increase in QALYs (0.006) and cost savings (-£27) in the Counterweight study in comparison to usual care, however these results were not statistically significant. When analysing completed Quality of Life questionnaires (IWQOL-Lite) Fuller et al (6)found there was a significantly greater change in utility for commercial weight loss programme (Weight Watchers (CP)) for Australia and Germany but not in the UK. However, this may have been due to the small sample size. When an intention to treat analysis was undertaken, CP as the preferred approach and the difference in utility scores between CP and standard care (SC) was reported statistically significant for Australia (0.0006) and the UK (0.033). A long-term analysis of the trial suggested over a lifetime of the patient resulted in a cost saving of US$47 per patient and an incremental 0.03 QALY gained per patient(27), however these differences were not statistically significant. Tsai et al(9) evaluated the long-term cost-effectiveness of

For peer review only - http://bmjopen.bmj.com/site/about/guidelines.xhtml

For peer review only

behavioural lifestyle medication compared with a combined diet, exercise and behaviour modification programme in Pennsylvania. The results suggested the costs associated with behavioural lifestyle modification were statistically significant ($p<0.05$). The difference in the results of these studies may be explained by different settings in which the intervention is executed, the type of interventions being evaluated and finally, the practice of usual care, which may differ across countries and healthcare practitioners. Given the evidence on primary care led programmes, there is a possibility that 10TT alongside other programmes would be effective at a low-cost.

The main strength of our analysis is that it is based on a large multicentre randomised trial with detailed information on resource use and utility values for a median follow-up period of two years. There were several limitations to our study. While data were recorded in the trial on standard weight-loss interventions for obese patients at each practice, individual data on standard weight-loss interventions received by the participant were not recorded. Under base case assumptions, excluding standard weight-loss interventions, the incremental NMB of 10TT versus usual care was not significantly different from zero, and this did not change when intervention costs in the control group were included. Hence, these costs did not make an appreciable difference to our conclusions. Secondly, the analysis took a UK NHS/PSS perspective. A wider perspective (e.g., societal) could have been taken, including costs to patients and families. Third, the time horizon was two years. We could have taken a longer time horizon, but due to the lack of difference in costs or benefits between groups, it appeared unlikely that incremental analyses would have been affected. Finally, there were missing NHS resource use and utility data in our analysis. We accounted for this using multiple imputation, assuming data to be missing at random.

For peer review only - http://bmjopen.bmj.com/site/about/guidelines.xhtml

In conclusion, the 10TT trial found that after three months' participants receiving 10TT lost significantly more weight than those receiving usual care, but there was no difference in weight change between the two groups at 24 months. Consistent with these findings, the present study found that differences in costs and QALYs between 10TT and usual care were not significantly different over the two-year period. There is no evidence to either recommend or advise against 10TT, based on cost-effectiveness considerations.

Acknowledgements

Weight Concern and Cancer Research UK developed the 10 Top Tips intervention and materials, and Weight Concern provided training on how to deliver the intervention. The General Practice Research Framework provided advice on the study design and was involved in both recruiting sites and training the health professionals at each site on the trial procedures, along with providing quality control visits. The authors would like to acknowledge the substantial intellectual contribution made by Professor Jane Wardle who sadly passed away prior to publication, and is deeply missed by all of her co-authors, colleagues and students

Contributors

NP undertook data analysis, interpreted the results and drafted and wrote the manuscript. RJB helped design the study, undertook data collection and critically reviewed the manuscript. BL helped design the study, critically reviewed the data and manuscript. RZO helped design the study and critically reviewed the manuscript. IN helped design the study and critically reviewed the manuscript. SM undertook the data analysis, devised the analysis plan, interpreted the results and critically reviewed the manuscript.

Funding

For peer review only - http://bmjopen.bmj.com/site/about/guidelines.xhtml

This work was funded by the Medical Research Council (MRC) – National Prevention Research Initiative (NPRI) (UK) grant number G080202. RJB's post was funded through this grant. SM, NP and IN are HEFCE-funded. RZO was funded by the National Institute for Health Research.

Disclaimer

The views expressed are those of the authors and not necessarily those of the funding bodies.

Competing interests

All authors have completed the ICMJE uniform disclosure form at http://www.icmje.org/coi_disclosure.pdf (available on request from the corresponding author) and declare that: NP, RJB, BL, RZO, IN and SM have nothing to disclose.

Ethics approval

This study has been approved by the South East London REC 2, 23/08/2010 (Reference number 10/H0802/59). All participants gave informed consent before taking part.

Provenance and peer review

Not commissioned; externally peer reviewed.

Data sharing statement

No additional data are available.

For peer review only

Table 1. Difference between groups in age, sex, costs and health-related quality of life at baseline

	Usual care				10TT			
	N	Mean	SE	95% CI	N	Mean	SE	95% CI
Age	270	58	0.77	(56.77, 59.79)	267	57	0.79	(55.93, 59.79)
Sex	270	0.35	0.03	(0.29, 0.41)	267	0.33	0.03	(0.03, 0.39)
Prior costs	270	1848	119	(1615, 2082)	267	2052	151	(1756, 2349)
Baseline EQ-5D	257	0.76	0.02	(0.73, 0.79)	255	0.74	0.02	(0.71, 0.76)

Table 2. Resource use, unit cost, and mean cost per participant for primary and secondary care services.

		Usual Care					10TT					UC	10TT	
	N	Mean	(SE)	Median	(IQR)	N	Mean	(SE)	Median	(IQR)	Unit cost (£)	Mean cost (£)	Mean cost (£)	P value~
**No imputation for missing values**														
GP surgery visit	204	9.4	(0.6)	7	(7.00)	182	9.7	(0.7)	7	(9.00)	45	417	425	0.84
GP home visit	196	0.1	(0.1)	0	(0.00)	176	0.0	(0.0)	0	(0.00)	292	37	9	0.11
GP phone	199	1.3	(0.2)	0	(1.00)	179	1.3	(0.2)	0	(1.00)	27	34	34	0.99
GP practice nurse	206	6.9	(0.6)	4	(6.00)	184	6.8	(0.7)	4	(6.50)	40	274	268	0.86
Nurse home visit	195	0.1	(0.1)	0	(0.00)	175	0.0	(0.0)	0	(0.00)	70	5	1	0.42
GP practice nurse phone	197	0.5	(0.2)	0	(0.00)	176	0.4	(0.1)	0	(0.00)	10	5	4	0.48
Dietician	195	0.1	(0.0)	0	(0.00)	175	0.1	(0.1)	0	(0.00)	35	4	4	0.92
Additional nurse visit	197	1.1	(0.2)	0	(1.00)	179	1.6	(0.2)	0	(1.00)	4	45	44	0.98
Inpatient admission	196	0.2	(0.0)	0	(0.00)	175	0.2	(0.0)	0	(0.00)	1713	372	387	0.90
Outpatient clinic	200	2.2	(0.2)	1	(3.00)	178	2.1	(0.2)	1	(3.00)	135	288	269	0.67
Accident & Emergency visit	199	0.4	(0.1))	0	(0.00)	175	0.4	(0.1)	0	(1.00)	177	65	67	0.93
Other outpatient services	194	1.0	(0.1)	0	(1.00)	179	1.0	(0.1)	0	(1.00)	135	124	129	0.85
**Missing values imputed***														**P value>**
GP surgery visit	270	9.4	(0.6)	9	(10.82)	267	9.4	(0.7)	9	(11.68)	45	419	420	0.98

For peer review only

For peer review only - http://bmjopen.bmj.com/site/about/guidelines.xhtml

GP home visit	270	0.1	(0.5)	0	(0.68)	267	0.0	(0.2)	0	(0.67)	292	36	13	0.20
GP phone	270	1.3	(0.2)	1	(3.31)	267	1.3	(0.2)	1	(3.28)	27	35	33	0.76
GP practice nurse	270	6.9	(0.6)	6	(10.30)	267	6.6	(0.7)	6	(11.19)	40	274	266	0.80
Nurse home visit	270	0.1	(1.0)	0	(0.87)	267	0.0	(0.1)	0	(0.98)	70	6	2	0.45
GP practice nurse phone	270	0.5	(0.6)	0	(2.00)	267	0.4	(0.1)	0	(2.19)	10	5	4	0.44
Dietician	270	0.1	(0.0)	0	(0.76)	267	0.1	(0.6)	0	(0.80)	35	4	4	0.10
Additional nurse visit	270	1.2	(0.2)	1	(4.00)	267	1.8	(0.2)	0	(3.48)	4	46	47	0.99
Inpatient admission	270	0.2	(0.1)	0	(0.90)	267	0.2	(0.1)	0	(0.85)	1713	373	379	0.96
Outpatient clinic	270	2.1	(0.3)	2	(4.29)	267	2.0	(0.2)	0	(4.11)	135	287	266	0.62
Accident & Emergency visit	270	0.4	(0.9)	0	(1.10)	267	0.4	(0.7)	0	(1.33)	177	67	69	0.92
Other outpatient services	270	1	(0.1)	1	(2.38)	267	1	(0.2)	0	(2.43)	135	122	129	0.83
**Weight loss interventions**														
10TT		-	-	-	-		-	-	-	-	23	-	-	-
Weight Watchers		-	-	-	-		-	-	-	-	78	-	-	-
Slimming World		-	-	-	-		-	-	-	-	93	-	-	-
Size Down Programme		-	-	-	-		-	-	-	-	73	-	-	-

GP = general practitioner. SD = standard deviation. IQR = inter-quartile range. Costs are in 2013/14 UK£. ~ From independent t-test, comparing mean cost between groups. *Missing values imputed using multiple imputation with 20 imputed datasets (see text). > From a linear regression model comparing mean between groups.

For peer review only - http://bmjopen.bmj.com/site/about/guidelines.xhtml

Table 3. Utility values and QALYs per patient

	Usual Care				10TT				
	N	Mean	SE	95% CI	N	Mean	SE	95% CI	p value~
**No imputation for missing values**									
3 months	206	0.77	0.017	(0.74, 0.80)	190	0.77	0.019	(0.73, 0.80)	0.96
6 months	163	0.76	0.019	(0.72, 0.80)	158	0.78	0.020	(0.75, 0.82)	0.35
12 months	150	0.78	0.019	(0.74, 0.81)	136	0.75	0.023	(0.71, 0.80)	0.37
18 months	137	0.76	0.021	(0.71, 0.80)	122	0.75	0.024	(0.71, 0.80)	0.93
24 months	155	0.77	0.020	(0.74, 0.81)	129	0.77	0.023	(0.73, 0.82)	0.99
Discounted QALYs	92	1.52	0.041	(1.44 1.61)	73	1.47	0.054	(1.36, 1.58)	0.45
**Missing values imputed***									**p value>**
3 months	270	0.75	0.015	(0.72, 0.78)	267	0.74	0.017	(0.71, 0.78)	0.98
6 months	270	0.77	0.016	(0.73, 0.80)	267	0.73	0.80	(0.73, 0.81)	0.55
12 months	270	0.76	0.016	(0.73, 0.80)	267	0.75	0.020	(0.71, 0.80)	0.70
18 months	270	0.76	0.019	(0.72, 0.79)	267	0.75	0.020	(0.71, 0.79)	0.77
24 months	270	0.76	0.019	(0.71, 0.79)	267	0.76	0.018	(0.72, 0.79)	0.93
Discounted QALYs	270	1.51	0.030	(1.45, 1.57)	267	1.51	0.033	(1.44, 1.58)	0.86

QALY = quality-adjusted life year. SD = standard deviation. CI = confidence interval. Costs are in 2013/14 UK£. CI were based on non-parametric bootstrapping for observed data and imputed data.

* Missing values imputed using multiple imputation with 20 imputed datasets (see text).

~ From independent t-test, comparing mean between groups.

> From a linear regression model comparing mean between groups.

For peer review only

Table 4. Cost description

	Usual care			10TT			P value~
	N	Mean	SE	N	Mean	SE	
**Cost, two years prior**							
Cost of primary and secondary care contacts	270	1516	103	267	1735	134	0.19
Drugs costs	270	332	46	267	317	38	0.80
Total cost	270	1848	119	267	2052	151	0.29
**Costs after randomisation (imputed)***							**P value>**
Cost of primary and secondary care contacts	270	1675	150	267	1631	169	0.42
Drugs costs	270	249	44	267	236	68	0.87
Intervention costs	-	-	-	267	23	-	-
Total cost	270	1925	165	267	1889	186	0.88

~ From independent t-test, comparing mean cost between groups. > From a linear regression model comparing mean between groups. * Missing values imputed using multiple imputation with 20 imputed datasets (see text).

For peer review only

For peer review only - http://bmjopen.bmj.com/site/about/guidelines.xhtml

Table 5. Incremental cost-effectiveness of 10TT versus usual care

	Incremental cost		QALYs gained		Incremental net monetary benefit (NMB)				Probability cost-effective	
					£20,000		£30,000			
	Mean (£)	(95% CI)	Mean	(95% CI)	Mean (£)	(95% CI)	Mean (£)	(95% CI)	£20,000	£30,000
Base case*	-36	(-511, 440)	0.0007	(-0.080, 0.816)	49	(-1709, 1800)	55	(-2489, 2583)	52%	52%
Base case with control group intervention cost~	-122	(-598, 353)	0.0007	(-0.080, 0.082)	140	(-1666, 1902)	148	(-2463, 2693)	56%	54%
Complete case analysis§	-66	(-907, 774)	-0.0472	(-0.180, 0.086)	-877	(-2338, 3404)	-1349	(-3269, 4972)	66%	67%
Primary and secondary care cost increased #	-40	(-556, 475)	0.0007	(-0.080, 0.081)	48	(-1743, 1851)	53	(-2520, 2623)	52%	51%
Drug cost increased #	23	(-396, 442)	0.0007	(-0.080, 0.077)	-14	(-1734, 1716	-9	(-2511, 2510)	49%	50%
All costs increased #	-39	(-554, 476)	0.0007	(-0.080, 0.082)	43	(-1756, 1851)	46	(-2537, 2641)	52%	51%
Primary and secondary care cost decreased ¥	-31	(-463, 401)	0.0007	(-0.080, 0.081)	54	(-1683, 1804)	64	(-2453, 2587)	52%	52%
Drug costs decreased ¥	26	(-387, 438)	0.0007	(-0.080, 0.081)	0	(-1741, 1727)	11	(-2530, 2520)	50%	51%
All costs decreased ¥	-32	(-460, 395)	0.0007	(-0.080, 0.081)	50	(-1682, 1811)	59	(-2458, 2599)	52%	52%

*N= 537. Data include values imputed using multiple imputation (see text). The 95% confidence intervals were derived from 1000 bootstrap replications of each of the 20 imputed datasets (see text). The incremental net monetary benefit and the probability 10TT is cost-effective are based on the QALYs gained and incremental costs and calculated at a maximum willingness to pay for a QALY of £20,000 and £30,000.

~ As for the base case analysis except standard weight-loss interventions (Weight Watchers, Slimming World, Size Down programme) are included in the control group.

§As for the base case analysis except there is no multiple imputation of missing values and the 95% confidence intervals were derived from 1000 bootstrap replications of a single dataset containing the n=72 participants in the 10TT group and n=91 participants in the usual care group with no missing values. The costs of interventions in the control group (Weight Watchers, Slimming World, Size Down programme) were excluded.

# As for the base case analysis except costs are increased by 10% for 10TT.

¥As for the base case analysis except costs are decreased by 10% for 10TT.

For peer review only - http://bmjopen.bmj.com/site/about/guidelines.xhtml

For peer review only

Figure 1. Cost-Effectiveness plane, base case analysis assumptions. Based on 20,000 bootstrap replications, from 20 imputed datasets.

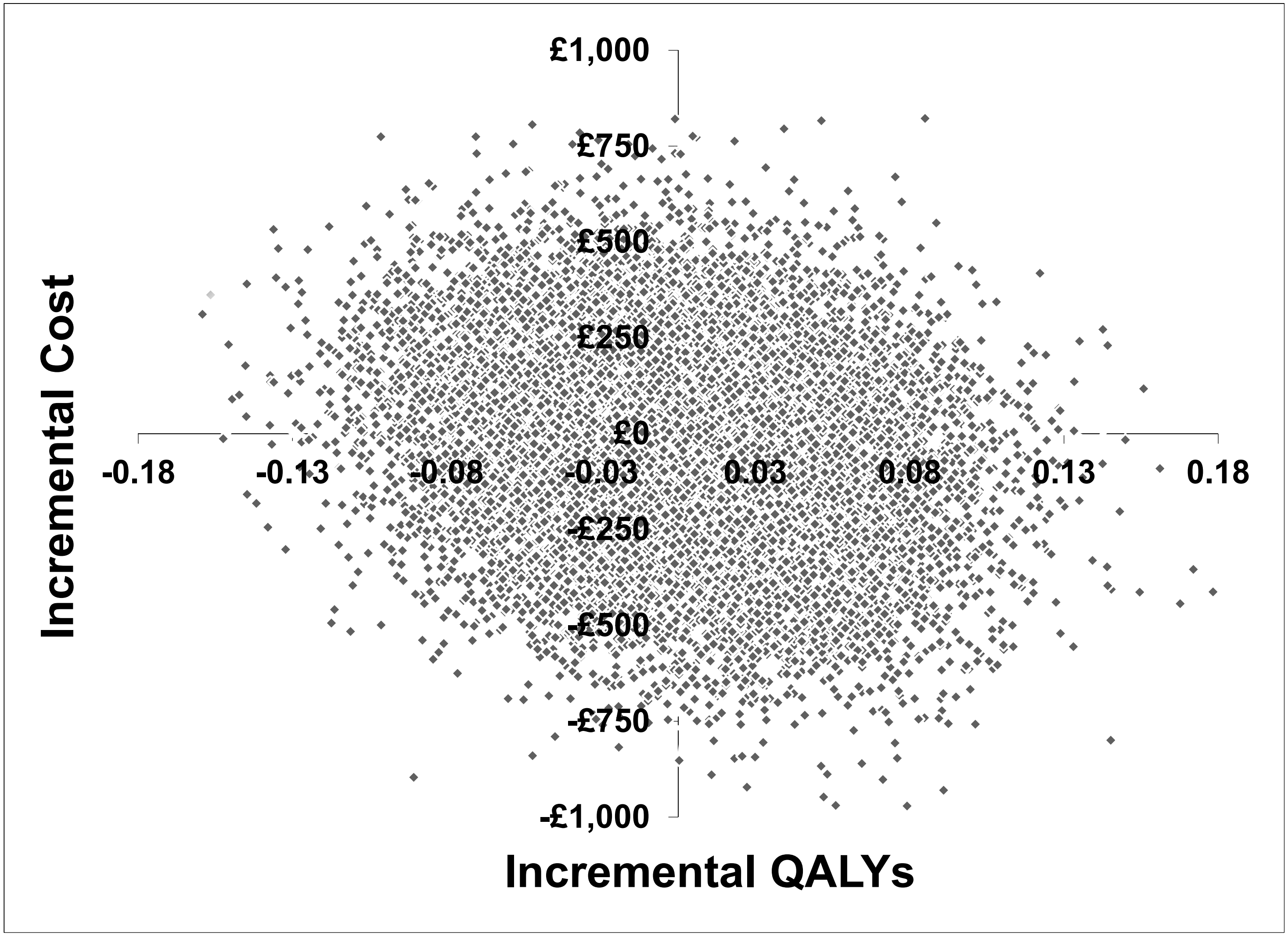

Figure 2. Cost-effectiveness acceptability curve showing the probability that 10TT is cost-effective vs usual care at different values of the maximum willingness to pay for a QALY (base-case assumptions)

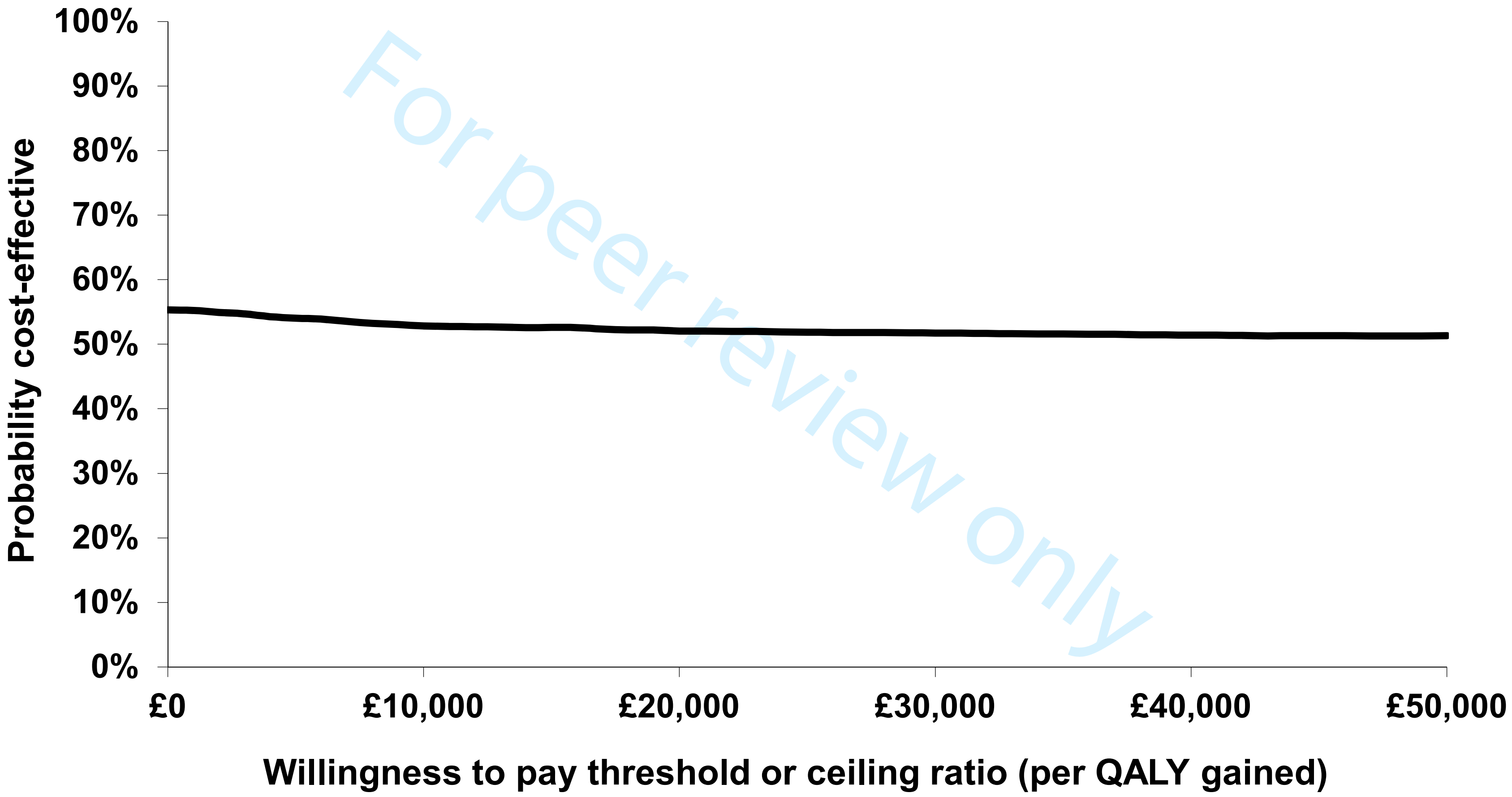

QALY = quality adjusted life year.

For peer review only - http://bmjopen.bmj.com/site/about/guidelines.xhtml

References

1. http://www.euro.who.int/en/health-topics/noncommunicable-diseases/obesity/data-and-statistics.
2. http://ec.europa.eu/eurostat/statistics-explained/index.php/Overweight_and_obesity_-_BMI_statistics.
3. Reducing obesity and improving diet. https://www.gov.uk/government/policies/reducing-obesity-and-improving-diet. Accessed 07.03.15.
4. UK and Ireland prevalence and trends. http://www.noo.org.uk/NOO_about_obesity/adult_obesity/UK_prevalence_and_trends. Accessed 07.03.15 [
5. Roux L, Kuntz KM, Donaldson C, Goldie SJ. Economic evaluation of weight loss interventions in overweight and obese women. Obesity (Silver Spring, Md). 2006;14(6):1093-106.
6. Fuller NR, Colagiuri S, Schofield D, Olson AD, Shrestha R, Holzapfel C, et al. A within-trial cost-effectiveness analysis of primary care referral to a commercial provider for weight loss treatment, relative to standard care--an international randomised controlled trial. International journal of obesity (2005). 2013;37.
7. http://data.worldbank.org/indicator/pa.nus.pppc.rf.
8. Fuller NR, Carter H, Schofield D, Hauner H, Jebb SA, Colagiuri S, et al. Cost effectiveness of primary care referral to a commercial provider for weight loss treatment, relative to standard care: a modelled lifetime analysis. Int J Obes. 2014;38(8):1104-9.
9. Tsai AG, Wadden TA, Volger S, Sarwer DB, Vetter M, Kumanyika S, et al. Cost-effectiveness of a primary care intervention to treat obesity. International journal of obesity (2005). 2013;37 Suppl 1:S31-7.
10. Trueman P, Haynes SM, Felicity Lyons G, Louise McCombie E, McQuigg MS, Mongia S, et al. Long-term cost-effectiveness of weight management in primary care. International journal of clinical practice. 2010;64(6):775-83.
11. Gardner B, Lally P, Wardle J. Making health habitual: the psychology of 'habit-formation' and general practice. The British journal of general practice : the journal of the Royal College of General Practitioners. 2012;62(605):664-6.
12. Lally P, Chipperfield A, Wardle J. Healthy habits: efficacy of simple advice on weight control based on a habit-formation model. Int J Obes. 2007;32(4):700-7.
13. National Institute for Health and Care Excellence (NICE). Guide to the methods of technology appraisal. London: NICE; 2013.
14. Curtis L. Unit costs of health and social care 2013. Kent: Personal Social Services Research Unit; 2013.
15. Department of Health. National Schedule of Reference Costs- 2012-2013. NHS trusts and NHS foundation trusts. London Department of Health; 2012.
16. Joint Formulary Committee . British National Formulary, 68th edition. London: BMJ Group and Pharmaceutical Press; 2013 [Available from: https://www.medicinescomplete.com/mc/bnf/current/.
17. Jolly K, Lewis A, Beach J, Denley J, Adab P, Deeks JJ, et al. Comparison of range of commercial or primary care led weight reduction programmes with minimal intervention control for weight loss in obesity: lighten Up randomised controlled trial. BMJ (Clinical research ed). 2011;343:d6500.
18. Rabin R, de Charro F. EQ-5D: a measure of health status from the EuroQol Group. Annals of medicine. 2001;33(5):337-43.
19. Brooks R. EuroQol: the current state of play. Health policy (Amsterdam, Netherlands). 1996;37.

For peer review only

For peer review only - http://bmjopen.bmj.com/site/about/guidelines.xhtml

20. Brooks R. EuroQol: the current state of play. Health policy (Amsterdam, Netherlands). 1996;37(1):53-72.
21. Petrou S, Dakin H, Abangma G, Benge S, Williamson I. Cost-utility analysis of topical intranasal steroids for otitis media with effusion based on evidence from the GNOME trial. Value in health : the journal of the International Society for Pharmacoeconomics and Outcomes Research. 2010;13(5):543-51.
22. Briggs A, Clark T, Wolstenholme J, Clarke P. Missing... presumed at random: cost-analysis of incomplete data. Health economics. 2003;12(5):377-92.
23. P R. Multiple imputation of missing values: update of ice. Stata J 2005;5:527-36.
24. Morris S, Patel NV, Dobson J, Featherstone RL, Richards T, Luengo-Fernandez R, et al. Cost-utility analysis of stenting versus endarterectomy in the International Carotid Stenting Study. International journal of stroke : official journal of the International Stroke Society. 2016;11(4):446-53.
25. Briggs AH, Gray AM. Handling uncertainty when performing economic evaluation of healthcare interventions. Health technology assessment (Winchester, England). 1999;3(2):1-134.
26. Beeken RJ, Leurent B, Vickerstaff V, Wilson R, Croker H, Morris S, et al. A brief intervention for weight control based on habit-formation theory delivered through primary care: results from a randomised controlled trial. International journal of obesity (2005). 2017.
27. Fuller NR, Carter H, Schofield D, Hauner H, Jebb SA, Colagiuri S, et al. Cost effectiveness of primary care referral to a commercial provider for weight loss treatment, relative to standard care: a modelled lifetime analysis. International journal of obesity (2005)2013.

For peer review only

For peer review only - http://bmjopen.bmj.com/site/about/guidelines.xhtml

Figure 1. Cost-Effectiveness plane, base case analysis assumptions. Based on 20,000 bootstrap replications, from 20 imputed datasets.

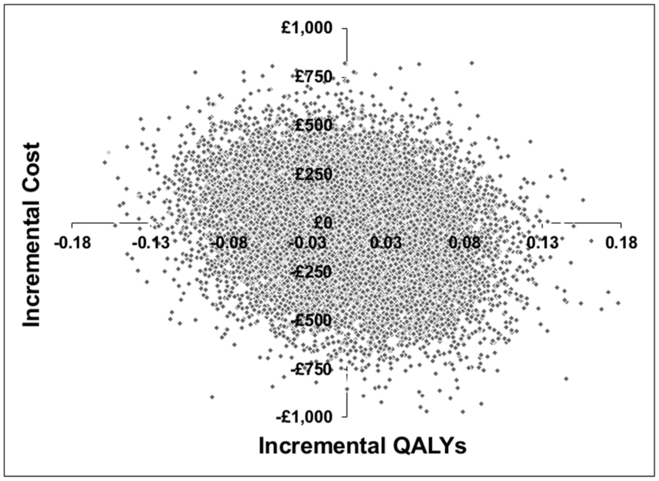

1

Figure 1. Cost-Effectiveness plane, base case analysis assumptions. Based on 20,000 bootstrap replications, from 20 imputed datasets.

296x209mm (100 x 100 DPI)

Figure 2. Cost-effectiveness acceptability curve showing the probability that 10TT is cost-effective vs usual care at different values of the maximum willingness to pay for a QALY (base-case assumptions)

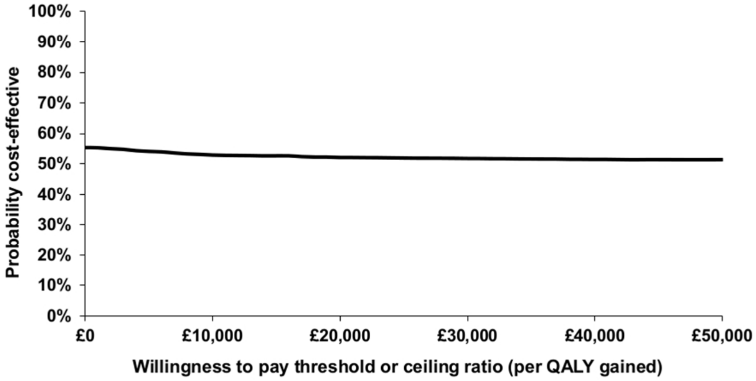

QALY = quality adjusted life year

Figure 2. Cost-effectiveness acceptability curve showing the probability that 10TT is cost-effective vs usual care at different values of the maximum willingness to pay for a QALY (base-case assumptions)

296x209mm (100 x 100 DPI)

For peer review only - http://bmjopen.bmj.com/site/about/guidelines.xhtml

Beeken et al. BMC Public Health 2012, 12:667
http://www.biomedcentral.com/1471-2458/12/667

BMC Public Health

STUDY PROTOCOL Open Access

Study protocol for the 10 Top Tips (10TT) Trial: Randomised controlled trial of habit-based advice for weight control in general practice

Rebecca J Beeken[1], Helen Croker[1], Stephen Morris[1], Baptiste Leurent[2], Rumana Omar[3], Irwin Nazareth[2] and Jane Wardle[1*]

For peer review only

Abstract

Background: Primary care is the first port of call for advice about weight control. There is hence a need for simple, effective interventions that can be delivered without specialist skills. We have developed such an intervention; the 10 Top Tips (10TT). This intervention was effective with respect to weight loss in a volunteer population, but has yet to be tested in primary care. The aim of this trial is therefore to test the effectiveness of the 10TT intervention in primary care, incorporating clinical outcomes and health economic analyses.

Methods/Design: The trial is a two-arm, individually-randomised, controlled trial in obese (BMI ≥ 30) adults (n = 520) in primary care, comparing weight loss in patients receiving the 10TT intervention with weight loss in a control group of patients receiving usual care. The intervention is based on habit formation theory, using written materials to take people through a set of simple weight control behaviours with strategies to make them habitual; an approach that could make it more successful than others in establishing long-term behaviour change. Patients will be recruited from 14 General Practices across England. Randomisation will be through telephoning a central randomisation service using a computer-generated list of random numbers. Patients are followed up at 3, 6, 12, 18 and 24 months. The primary outcome is weight loss at 3 months, with assessment by a health professional who is blind to group allocation. Other follow-ups will be un-blinded. We will examine whether weight loss is maintained up to 24 months. We will also assess changes in the automaticity of the 10TT target behaviours and improvement in clinical markers for potential co-morbidities. Finally, we will undertake a full economic evaluation to establish cost-effectiveness in the NHS context.

Discussion: If proven to be effective when delivered through primary care, 10TT could make a highly cost-effective contribution to improvements in population health.

Trial Registration: ISRCTN16347068

Keywords: Obesity, Primary care, Weight control, Habits

Background

Obesity presents an enormous public health burden [1] contributing to morbidity and mortality through Type 2 diabetes, CVD, and cancer [2], and being associated with a range of disabilities that increase use of health services [3]. It also places substantial economic costs on society [4,5]. The cross-government strategy *Healthy Weight, Healthy Lives* [6] identifies Primary Care as the 'first port of call' for advice about weight control, creating a need for simple, effective interventions that can be delivered by the primary care team without specialist therapeutic skills. Current non-medical treatments (e.g. cognitive behaviour therapy) require considerable expertise on the part of the practitioner and a high level of commitment from the patient; limiting their value for first-line intervention [7,8]. Maintenance of weight loss is also notoriously difficult [9].

* Correspondence: j.wardle@ucl.ac.uk
[1]Department of Epidemiology & Public Health, University College London, Gower Street, London WC1E 6BT, UK
Full list of author information is available at the end of the article

BioMed Central
© 2012 Beeken et al.; licensee BioMed Central Ltd. This is an Open Access article distributed under the terms of the Creative Commons Attribution License (http://creativecommons.org/licenses/by/2.0), which permits unrestricted use, distribution, and reproduction in any medium, provided the original work is properly cited.

A novel, theory-based, self-guided, approach to weight control

Healthy 'habits' in the domains of diet and physical activity are the goal of most weight-loss programmes, but few if any draw explicitly on the theory of habit-formation to create healthy habits. According to psychological theory, the essential feature of habits is that they are stimulated by environmental contextual cues. Subjectively, they appear 'automatic', i.e. they require minimal willpower or effort [10]. Psychological research shows that repetition of a behaviour in a consistent context enables it to become automatic, and once automatic, it is more resistant to change than deliberative (intentional) behaviours [10,11]. There has been growing interest in the application of habit theory in health behaviour change, with evidence that habit strength is associated with lower dependence on deliberative/intentional processes [12-14].

In the first therapeutic application of habit theory, we developed a simple intervention promoting a set of negative energy balance behaviours. This took the form of a leaflet (called '*Ten Top Tips*'; 10TT) listing the target behaviours alongside advice on repetition and context-stability. Self-monitoring was recommended during the habit acquisition phase. No further clinical contact was involved. In a pilot study, outcomes and acceptability were very positive, so we carried out a small-scale, randomised controlled trial in a volunteer population [15]. The intervention produced significantly greater weight loss than the no-treatment control condition at follow up (8 weeks) using a last-observation-carried–forward (LOCF) analysis (habit group: -2.0 kg; control: -0.4 kg). Unusually for behavioural treatments, modest weight loss continued after the end of the active treatment period, reaching–3.6 kg in completers at 32 week follow-up (LOCF = –2.6 kg), with 54% (LOCF = 26%) of participants achieving the 5% weight loss associated with beneficial health effects. Weight loss appeared to be associated with increased automaticity of behaviours, supporting the idea that habit formation underpinned the effectiveness of the intervention and the maintenance of effects after the intervention period. Importantly, the intervention was rated as easy and pleasant to implement by the participants. These findings provide a strong case for testing the intervention in a full-size randomised trial in primary care and including health outcomes.

Study Objectives

Primary Research Objective

The main aim of the study is to test the hypothesis that a simple weight control intervention in people with a BMI ≥ 30 based on habit-formation theory (10TT) will achieve clinically significant loss in body weight over 3 months in obese primary care patients, compared with patients placed receiving 'usual care'.

Secondary Research Objectives

The main secondary research objectives are to test for differences in waist circumference, BMI and the number of people achieving a 5% reduction in weight over the trial period, and to examine whether the effects on weight loss are maintained up to 12 and 24 months. We will also explore whether the intervention leads to improvements in, and increased automaticity of, diet and physical activity behaviours at the end of the treatment period and over the 24 month follow up period. Improvements over 3 months in clinical markers for potential co-morbidities (blood pressure, total cholesterol/low-density lipoprotein (LDL) and blood glucose) will be investigated.

Exploratory analyses will look at differences between the two groups over the trial period in health-related quality of life, self-efficacy, restraint, self-regulation and social support, as well as considering whether baseline scores on these measures contribute to differences in how effective the intervention is. Potential mediators will also be explored.

Finally, at 24 months we will undertake a full economic evaluation to establish cost-efficacy in the NHS context.

Methods/Design

The trial is a two-arm, individually-randomised, controlled trial in obese adults in primary care, comparing the 10TT intervention with usual care. Figure 1 illustrates the pathway through the trial.

Recruitment

Participants

Obese (BMI ≥ 30) primary care patients identified from electronic General Practice records recruited from 14 General Practices.

Recruitment strategy/recruitment rate

Practices will be recruited through the General Practice Research Framework (GPRF) and selected to represent urban and rural areas, and to include practices serving ethnically diverse or socio-economically deprived populations. In each practice we will identify all people with recorded BMI ≥ 30 through electronic computer searches and invite them to take part. We expect BMI data to be available on 30-40% of patients, though it may have increased in recent months [16]. In practices where the number of patients with a recorded BMI ≥30 exceeds 500, a random sample of 500 patients will be selected to be invited. The GP will check the list for exclusions. Eligible patients will be sent a letter from their practice,

For peer review only

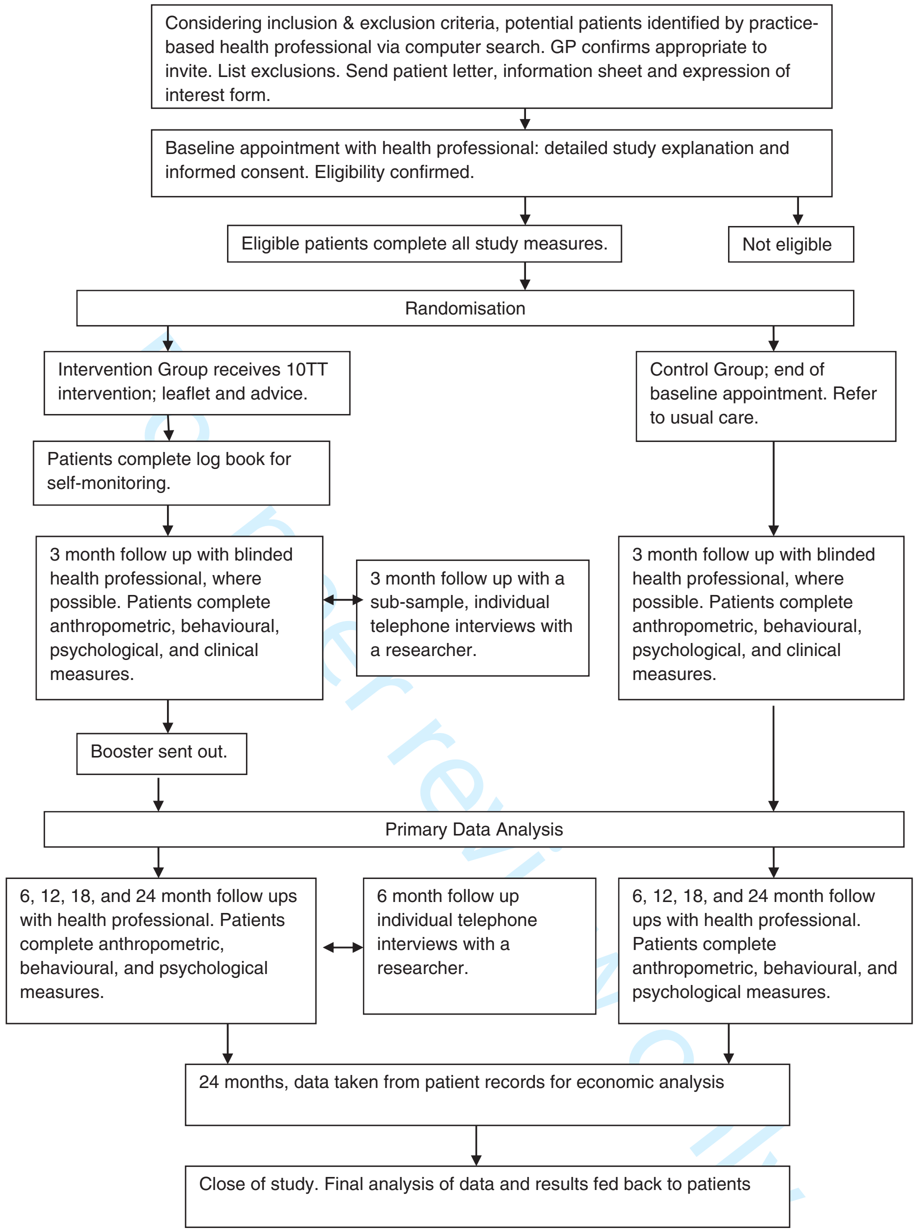

Figure 1 Flow chart of patient involvement in the 10TT trial.

with an enclosed information sheet, which states that the study is concerned with promoting healthy eating and activity habits to help with weight loss and that we are specifically interested in whether a leaflet describing behaviours known to be associated with weight control, along with strategies to make them habitual/automatic ('10 Top Tips') will lead to more weight loss compared with usual care. No further details of the intervention will be given. The letter will include an 'expression of interest' form on which to state whether or not they would like to take part in the study and a stamped addressed envelope. Patients may also be invited opportunistically if they have their BMI recorded after the mail-out and they are eligible. Recruitment is expected to take place over 12 months to achieve the required sample size.

Inclusion criteria

We will restrict the study to adults (age ≥ 18) who are able to consent for themselves, and for whom linear growth has stopped and therefore weight loss could not compromise growth. We are directing the intervention towards the obese population (BMI ≥ 30) because obesity is recognised as conferring health risk, regardless of

For peer review only - http://bmjopen.bmj.com/site/about/guidelines.xhtml

comorbidities, and therefore the offer of treatment in the primary care context is justified.

Exclusion criteria

We will exclude anyone who is i) unable to provide informed consent due to mental incapacity or active psychotic illness, ii) pregnant, or iii) terminally ill. We will not have an upper age limit because we believe that interested older patients would be able to benefit.

Consent

Consent to enter the study will be sought from each participant after they have received an information sheet in the post beforehand and a full explanation has been given in person, with the opportunity to ask questions. The right to decline participation without giving reasons will be respected. Signed participant consent will be obtained. After the participant has entered the study their clinician remains free to give alternative treatment to that specified in the protocol at any stage if they feel it is in the participant's best interest, but the reasons for doing so will be recorded. In these cases, participants will remain within the study for the purposes of follow-up and data analysis. All participants are free to withdraw at any time from the protocol treatment without giving reasons and without prejudicing further treatment.

Measures

Demographics

Demographic data (gender, date of birth, post code, ethnicity, qualifications) will be collected at baseline.

Anthropometric measures

Body weight (kg) will be measured using TANITA scales supplied to practices specifically for use in this study. Patients will be asked to remove any outer garments, take off shoes and empty pockets. The usual practice equipment will be used to measure height (cm). Patients will be instructed to remove their shoes, stand with feet flat on the floor, feet together and heels against the wall, and with shoulder blades and buttocks also touching the wall, arms hanging loosely by their side, facing straight ahead. They will be instructed to breathe in deeply and stretch to their fullest height when the measurement is taken. BMI will be calculated from these measurements using the standard formula of weight (kg)/(height (m))^2.

Waist circumference will be measured over light clothing, but patients will again be asked to remove outer layers, shoes, and tight garments. The waist is defined as the point midway between the iliac crest (top of the hip bone) and the lower rib, and measurements will be taken at the end of normal expiration.

Behavioural measures

Behaviour will be assessed using self-report questionnaires for diet and physical activity. Diet will be assessed using questions from the Dietary Instrument for Nutrition Education [17] on fat intake, a validated tool that has been shown to correlate with food diaries. We will also ask additional questions on fruit and vegetable intake, snacks, and type of drinks consumed (including number of units from alcohol) as these aspects of diet (along with fat intake) are specifically targeted by the intervention. We will assess physical activity using the Recent Physical Activity Questionnaire [18], a well-validated questionnaire that assesses physical activity over the past 4 weeks in 4 domains (work, travel, recreation, and domestic life). Changes in the 10TT target behaviours will be measured using 2 items that ask about the frequency of the behaviour over the past two weeks, and automaticity of the behaviours (item taken from the Self-Report Habit Index [19]).

Psychological measures

Brief, standardised questionnaires with good reliability and validity will be used. Health-related quality of life will be measured using the EuroQuol –5 domains (EQ-5D [20]), which asks respondents to first rate their health status on 5 domains (mobility, self-care, usual activities, pain/discomfort and anxiety/depression) and then rate their overall health state using a visual analogue scale that ranges from 0 (worst imaginable state) to 100 (best imaginable state). Self-efficacy will be assessed using the Weight Efficacy Lifestyle Questionnaire [21], a validated 20-item measure that asks participants to rate how confident they are that they can resist eating in response to five situational factors (negative emotions, availability, social pressure, physical discomfort, and positive activities). The 10-item restraint sub-scale of the Dutch Eating Behaviour Questionnaire [22] will be used to measure restrained eating. This scale has been used extensively and has strong reliability and validity. Self-regulation of eating will be assessed using a version of the Shortened Self-Regulation Questionnaire [23] adapted for the purposes of this study. The wording of the original 31 items, which assessed general self-regulation skills, was changed to apply specifically to weight regulation behaviours. For example 'I have trouble making plans to help me reach goals' was changed to 'I have trouble making plans to help me reach my weight loss goals'. We also removed the 'uncertain or unsure' response option, so that responses are on a 4-point scale ('strongly disagree'/'disagree'/'agree'/'strongly agree'). Finally, we will assess social support for physical activity and healthy eating using brief, theory-based scales with demonstrated adequate reliability and validity [24].

For peer review only - http://bmjopen.bmj.com/site/about/guidelines.xhtml

Beeken et al. BMC Public Health 2012, 12:667
http://www.biomedcentral.com/1471-2458/12/667

Clinical measures

Blood pressure and blood cholesterol/LDL/glucose levels will be assessed with standard practice procedures. Blood samples are being analysed within the NHS (as opposed to by a single laboratory) so there may be some variation in assessment. Due to practical considerations we are measuring random glucose rather than fasting, which may overestimate risk in our sample.

Economic measures

Economic measures will include cost of the intervention, volume of resource use for primary and secondary health services (obtained from GP records) and unit costs (to be attached to the resource use data, obtained using routinely available national sources where possible and local estimates where required), and EQ-5D scores [20].

Randomisation

The unit of randomisation is the patient. Randomisation will be done by telephoning a central randomisation service (Health Service Research Unit at Aberdeen) ensuring allocation concealment, after the participant has provided informed consent and baseline data. A computer-generated list of random permuted blocks of varying sizes will be used. Randomisation will be stratified by practice to ensure socioeconomic balance between randomised groups.

Patients randomised to the intervention group will be given the 10TT leaflet and self-monitoring log book to use in the acquisition phase, with additional brief information on the idea of forming healthy habits. Following this appointment they will be sent a booster letter with a second copy of the 10TT leaflet and can request a second log book if they would like one.

Patients randomised to the control group will be referred to the Practice's usual care. We will ask participating Practice's to inform us of what their usual care consists of, and this will be recorded. This will at least be a referral to a practice-based health professional not involved in the trial for a discussion on healthy eating, but in some Practices, may also be a referral to Weight Watchers.

The intervention

10TT is a self-guided leaflet for weight management focusing on making simple diet and exercise behaviours habitual (see [15]). It is the first behaviour change intervention to be explicitly based on habit-formation theory. The component energy balance behaviours that are intended to become habitual reflected the consensus among researchers, clinicians and policy makers on healthy diet and lifestyle and were developed with input from these groups. They were also selected as practical on the basis that they were relatively common in the general population.

Table 1 outlines the tips along with their estimated calorie deficit or purpose. Seven of the 10 tips are the energy balance behaviours (intended to become habitual); three help to promote habit formation, nutrition awareness and avoidance of slips. Each 'tip' has a memorable name, an explanation of why it helps weight control, and suggestions on incorporating it into daily activities. Participants are also provided with a simple logbook for self-monitoring during the habit acquisition phase and a wallet sized card with guidance on food labels.

Practice-based health professionals (nurses or health care assistants) in each centre will attend a training session run by the charity Weight Concern and the General Practice Research Framework (GPRF) to enable them to describe the intervention to patients and carry out all necessary study procedures. Health professionals will also be provided with a study manual. Deliverers of the intervention will be instructed to spend 30 minutes taking patients through the leaflet using a flip chart and defined script contained in the manual. Manualising the treatment in such a way ensures that the delivery is standardized. Quality checks will also be carried out and ongoing support will be provided to practice staff where required.

The active treatment period is 3 months, defined in terms of the appropriate duration for habit formation and the period over which participants are recommended to keep self-monitoring records (logbook) to aid habit acquisition.

Follow up

Proposed duration of follow-up

The primary end-point will be 3 months; other time points will provide information on maintenance of health benefits following the intervention. Data will be collected at baseline, 3, 6, 12, 18 and 24 months. We propose to assess maintenance up to 24 months to provide comparability with other interventions for obesity, and to be long enough for participants to have settled into a pattern of use and for the intervention to have lost its novelty. The study will end when all patients still involved in the study have attended their 24 month follow-up.

Rate of loss to follow-up

In the earlier efficacy trial, the follow-up rate was 75% at two months. Primary care research participants usually have good follow-up rates although it may be lower than in the efficacy trial because of the programme being offered rather than being volunteered for. Reminders and

For peer review only - http://bmjopen.bmj.com/site/about/guidelines.xhtml

Beeken *et al. BMC Public Health* 2012, **12**:667
http://www.biomedcentral.com/1471-2458/12/667

Table 1 Scientific justification for the '10 Top Tips'

Tip	Scientific justification	Estimated daily calorie deficit
**1. Keep to your meal routine**		
Try to eat at roughly the same times each day, whether this is two or five times a day.	People who succeed at long term weight loss tend to have a regular meal rhythm (avoidance of snacking and nibbling) and show 'flexible' rather than 'rigid' control' of eating [25]. A consistent diet regimen across the week and year also predicts subsequent long-term weight loss maintenance [26].	This tip helps encourage habit development.
**2. Go reduced fat**		
Choose reduced fat foods (e.g. dairy foods, spreads, salad dressings) where you can. Use high fat foods (e.g. butter and oils) sparingly, if at all.	There is a great deal of evidence to support the effectiveness of low-fat diets (where 30 % or less of total daily energy is from fat), which produce weight loss by decreasing calorie intake [27]. Following a low-fat diet is also associated with better weight maintenance [28].	- 200 Kcal
**3. Walk off the weight**		
Walk 10,000 steps (equivalent to 60–90 minutes moderate activity) each day. You can use a pedometer to help count the steps.	Achieving the UK government recommendation of at least 30 minutes of at least moderate intensity physical activity on 5 or more days a week would increase most people's energy expenditure and contribute to weight management [29]. More activity (45–60 mins) may be required to prevent the transition to overweight and obesity and maximize weight loss [30]. People who have lost weight may need to do 60–90 minutes of activity a day to maintain their weight loss [29,30]. Doing 10,000 steps/day is approximately the equivalent to at least 60 minutes of walking at a brisk pace (4.5 mph) [31].	- 100 to 200 Kcal
**4. Pack a healthy snack**		
If you snack, choose a healthy option such as fresh fruit or low calorie yogurts instead of chocolate or crisps.	Readily-available snack foods and drinks are often high in energy and tend to be used to supplement rather than replace meals. Between 1993 and 1998 sales of snacks more than tripled in the UK from £173 million to £541 million [4]. Snack consumption is related to a higher daily energy intake [32].	- 150 Kcal
**5. Learn the labels**		
Be careful about food claims. Check the fat and sugar content on food labels when shopping and preparing food.	Food labels detailing the caloric and nutritional content of foods provide a basis for making healthy food choices [4]. Inadequate labeling can have a negative impact on nutrition [4]. Providing individuals with simple methods to understand labels will facilitate informed choices [33].	This tip helps people to make informed choices.
**6. Caution with your portions**		
Don't heap food on your plate (except vegetables). Think twice before having second helpings.	Portion sizes have increased in the past 30 years [34,35]. Larger portions contain more calories and can contribute to excess energy intake and weight gain. Eating satisfying portions of low-energy-dense foods can help enhance satiety and control hunger while restricting energy intake for weight management [35].	- 100 Kcal
**7. Up on your feet**		
Break up your sitting time. Stand up for ten minutes out of every hour.	Inactive people are more likely to be obese than active people [29]. Time spent in sedentary behaviors is related to overweight and obesity, independent of physical activity level [36,37]Decreasing sedentary time and increasing light–to-moderate activity may bring substantial health benefit [29,36].	- 100 Kcal
**8. Think about your drinks**		
Choose water or sugar-free squashes. Unsweetened fruit juice contains natural sugar so limit to one glass	Intake of sugar-sweetened soft drinks has increased over the last 30 years; up by 135 % (278 kcal) in 5 years [38].	- 150 Kcal

For peer review only

Beeken et al. BMC Public Health 2012, 12:667
http://www.biomedcentral.com/1471-2458/12/667

Table 1 Scientific justification for the '10 Top Tips' *(Continued)*

a day (200 ml/1/3 pint). Alcohol is high in calories; limit to one unit a day for women and two for men.	Higher consumption of sugar-sweetened beverages is associated with greater weight gain [39]. Intake of calorific drinks may lead to excess energy intake that is not compensated for elsewhere in the daily diet [39].	
**9. Focus on your food**		
Slow down. Don't eat on the go or while watching TV. Eat at a table if possible.	More TV viewing tends to be associated with a higher calorie intake. Internal cues regulating food intake may not be as effective while distracted by the TV [40].	This tip helps place the focus on current habits and to avoid unconscious slips in behavior.
**10. Don't forget your 5 a day**		
Eat at least 5 portions of fruit and vegetables a day.	The UK Department of Health recommends 400 g of fruit and vegetables a day. Fruits and vegetables have high nutritional quality and low energy density. Eating the recommended amount produces health benefits including reduction in the risk of cancer and coronary heart disease [41].	This tip is important for health.
**Total Calorie Deficit**		- 800 to 900 Kcal

additional attempts to contact participants may have a positive effect on retention.

Measurement of outcomes at follow up

Table 2 summarises the measurement of outcomes at baseline and follow-up. Weight and waist circumference will be assessed at all time points, as will questionnaire-based measures. Clinical measures will be taken only at 3 months.

Qualitative interviews

We will conduct semi-structured individual telephone interviews with a purposive sample from the intervention group (n ~ 20) at 3 and 6 month follow-up to explore self-reported barriers to, and facilitators of, compliance with each of the 'tips'.

Compliance

The intervention requires little supervision or contact with health professionals, and the instructions are simple. In the pilot study, the ease of the intervention was well received by the participants, even those with fewer years of education, who compared it favourably with other weight loss programmes. Patients randomised to the intervention will be given logbooks with a simple system of recording daily tip adherence, and will be asked to return completed books to the researchers via post. This data will provide some idea of the level of compliance and will be presented descriptively.

Methods for protecting against sources of bias

It is not possible to blind participants, but follow-up at the end of the active treatment period (3 months) will be with a health professional blind to the intervention allocation where possible. Practical reasons (e.g. unavailability of the appropriate health professional) may prohibit blinded follow ups for all patients at 3 months. Given that the primary outcome is at 3 months, and the use of pre-dominantly objective measures, it was felt that blinding was un-necessary after the 3 month follow up. A detailed analysis plan will be developed prior to the analysis and the data-analyst will remain blind to allocation until completion of the final analyses. We will limit non-response bias through maximising follow-up using reminder phone calls prior to their follow-up appointments. Number of contact attempts and reason for withdrawing from follow-up will be recorded to facilitate statistical analyses of non-response bias. Non-attenders will also be asked to provide a self-report of their weight over the phone. Selection bias arising from recruitment of patients with recorded BMI data is largely unavoidable but comparison of sample characteristics

Table 2 Summary of baseline and follow-up assessments

Measure		Month of treatment				
	Baseline	3 (End of active treatment phase)	6	12	18	24
Weight	X	X	X	X	X	X
Waist circumference	X	X	X	X	X	X
Questionnaire-based measures	X	X	X	X	X	X
Blood pressure	X	X				
Blood cholesterol/LDL/glucose levels	X	X				

For peer review only

with those of the wider (obese) population obtained from Health Survey for England data will allow us to assess the extent of this bias.

Statistics and data analysis

Sample size and assumptions

The exploratory trial of the intervention [15] showed a difference of weight change between baseline and 8 weeks of 1.41 kg (SD = 1.9) between the intervention and control groups. As the trial population may be more heterogeneous, and the outcome is measured 4 weeks later, we powered the study to detect a mean difference in weight change of 1.0 kg, with a standard deviation of 2.5. Based on a two sample test, 132 evaluable participants per arm are required to detect such a difference with 90% power, and a significance level of 5%. This sample size has been calculated using the statistical software STATA. However, a clustering effect may occur in the intervention arm due to different health professionals delivering the intervention in each practice. Power calculation formulae for a partially clustered design have been reported [42]. Based on an average cluster size of 13 evaluable participants (those completing 3 months follow-up), and an ICC of 0.05 [43] in the intervention arm, a total of 364 evaluable patients at 3 months (26 in each of the 14 practices) would provide 92% power to detect such a difference. This should insure the power stays above 90% even with a small imbalance in cluster size between practices [44]. Allowing for 30% attrition (26% observed in the pilot study at 8 weeks), 260 participants would need to be recruited by arm, or 520 in total.

Data analysis

Baseline characteristics will be reported by each arm using descriptive statistics. A random effects linear regression model accounting for clustering by health professionals delivering the intervention in the intervention arm will be used to compare the weight change at 3 months from baseline between the two groups. This analysis will be adjusted for baseline weight which should make the statistical analysis more efficient. Random effects linear regression model will also be used to investigate the clustering effects due to practice.

Bias due to missing data will be investigated. If required, sensitivity of the results to missing data will be investigated using different approaches for handling missing data, including adjusting for predictors of missingness that are related to the outcome, multiple imputation and baseline observation carried forward. The possibility that data may not be missing not at random (MNAR) will also be considered and if necessary approaches that can handle MNAR data will be used as part of the sensitivity analysis [45]. Additionally, the scope of incorporating information on reasons for drop-out in the analyses will also be explored.

Secondary outcomes will be compared between the intervention groups using appropriate regression models taking account of clustering. Subgroup analyses will investigate intervention effects by gender, age (in tertiles), baseline BMI (<35 vs. ≥35), ethnic origin (white vs. other) and deprivation (tertiles of postcode-based Index of Multiple Deprivation) as part of secondary analyses. Results from all secondary analyses will be considered as exploratory.

The primary aim of the trial is to evaluate the effectiveness of offering the intervention; therefore all analyses will be performed on an intention to treat basis. A detailed analysis plan will be prepared nearer the data analysis stage.

Economic analyses

Economic analyses will conform to NICE's preferred methodology [46]. The costs of the intervention will be calculated using a bottom-up method. Within-trial quality adjusted life years (QALYs) gained during the follow-up period will be derived from EQ-5D scores measured throughout the trial until two years. Predicted lifetime QALYs gained will be estimated using decision-analytic modelling to predict long-term quality adjusted survival and costs. Cost-effectiveness will be measured in terms of incremental cost per unit change in body weight and per QALY gained (using both within-trial and lifetime QALYs). Results will be subjected to simple and probabilistic sensitivity analysis.

Analysis of qualitative interviews

All interviews will be audio recorded and transcribed. Thematic analysis will be used to identify the main themes that will form the basis of our results.

Data storage and retention

Data management will be handled by PRIMENT Clinical Trials Unit with data being held according to Good Clinical Practice (GCP) requirements. As per GCP requirements, data will be held for a minimum of ten years from completion of the project.

Ethical approval, research governance and data access

Ethical approval was obtained from the South East London Research Ethics Committee 2 via IRAS, (Ref No. 10/H0802/59, Approval granted 9th July 2010) with other participating centres providing site-specific approval as per normal IRAS procedures. NHS Research and Development (R&D) approval was obtained from all participating NHS Boards prior to the start of the trial. Further advice and support on governance and GCP

issues will be provided by PRIMENT Clinical Trials Unit, who provide Standard Operating Procedures for tasks such as obtaining consent, managing and archiving data, access to trial data, training and how to handle breaches of GCP. The trial has been submitted to the International Standard Randomised Controlled Trials (ISRCT) and allocated the number ISRCTN16347068.

Study/trial sponsorship

University College London is the sponsor of this trial.

Discussion

The 10TT intervention is consistent with Foresight's recommendations which include *promoting healthier food choices, building physical activity into our lives,* and *personalised advice and support* [2]; complementing preventive care with treatment for those who already have weight problems.

Obese patients have regular contact with primary care even before experiencing any specific obesity-related co-morbidities [47]; providing an opportunity for early intervention. However, weight management activities in primary care are limited and inconsistent [48], with some GPs reluctant to raise the subject of weight [49], and many lacking confidence in existing treatments [50]. The 10TT intervention addresses these issues, offering a simple, low-cost, treatment that can be delivered by primary care staff without special expertise.

Existing reviews identify a need for novel, practical, empirically supported trials with adequate follow-up [8,48,51,52]. Many ongoing or recently completed behavioural clinical trials registered with the metaregister of controlled trials (mRCT) focus on interventions with high levels of therapeutic contact (e.g. counselling, tailored interventions, variable content over time). These interventions show promise for use with patients who are willing to engage in a therapeutic programme but may not be as acceptable to those with more limited interest in weight control. The habit-formation approach could complement these by providing a low-contact alternative. Evidence of better weight loss maintenance for self-guided than professionally-led programmes [53] is also supportive of this approach.

The results from this study will assess the impact of the 10TT intervention on body weight over 3 months and will provide information on whether the weight loss is maintained up to 24 months. It will also increase understanding of whether the focus on habit formation leads to improvements in, and increased automaticity of, diet and physical activity behaviours, and if it is cost-effective in the NHS context. If proven to be effective when delivered through primary care, 10TT could make a highly cost-effective contribution to improvements in population health.

Abbreviations

10TT: 10 Top Tips; EQ-5D: EuroQol −5 domains; GCP: Good clinical practice; GPRF: General Practice Research Framework; ISRCT: International Standard Randomised Controlled Trials; LDL: Low-density lipoprotein; LOCF: Last-observation-carried-forward; mRCT: metaregister of controlled trials; MNAR: Missing not at random; NHS: National Health Service; NICE: National Institute for Health and Clinical Excellence; QALYs: Quality adjusted life years; R&D: Research and Development.

Competing interests

The authors declare that they have no competing interests.

Authors' contributions

JW, IN, HC and SM conceived and wrote the funding application. JW is the PI and grant holder. RJB, BL and RO have contributed to subsequent refinements of the design and analysis plan. RJB drafted the final protocol and is the trial manager. BL and RO are the trial statisticians. SM is the health economist for the trial. JW and HC provide expertise on obesity and weight management interventions. IN provides expertise on trials and primary care. All authors revised the manuscript, and read and approved the final version.

Acknowledgements

Weight Concern and Cancer Research UK developed the 10 Top Tips intervention and materials, and Weight Concern is providing training on how to deliver the intervention. The General Practice Research Framework provided advice on the study design and is involved in both recruiting sites and training the health professionals at each site on the trial procedures, along with providing quality control visits.

The trial is funded by the Medical Research Council – National Prevention Research Initiative. RJB's post is funded through this grant. HC and JW are funded by Cancer Research UK. SM and IN are HEFCE-funded. RO and BL are funded by the National Institute for Health Research.

Author details

[1]Department of Epidemiology & Public Health, University College London, Gower Street, London WC1E 6BT, UK. [2]Department of Primary Care & Population Health, University College London, Gower Street, London WC1E 6BT, UK. [3]Department of Statistical Science, University College London, Gower Street, London WC1E 6BT, UK.

Received: 20 July 2012 Accepted: 9 August 2012
Published: 16 August 2012

References

1. Allender S, Rayner M: **The burden of overweight and obesity-related ill health in the UK.** *Obes Rev* 2007, **8**(5):467–473.
2. *Tackling Obesities.*: Future Choices; www.foresight.gov.uk/Obesity/17.pdf.
3. Visscher TL, Seidell JC: **The public health impact of obesity.** *Annu Rev Public Health* 2001, **22**:355–375.
4. *Obesity.* [www.publications.parliament.uk/pa/cm200304/cmselect/cmhealth/23/23.pdf]
5. Counterweight Project Team: **Influence of body mass index on prescribing costs and potential cost savings of a weight management programme in primary care.** *J Health Serv Res Policy* 2008, **13**(3):158–166.
6. *Healthy Weight, Healthy Lives: A Cross Government Strategy for England.* [www.dh.gov.uk/en/Publicationsandstatistics/Publications/PublicationsPolicyAndGuidance/DH_082378].
7. *Lightening the load: Tackling overweight and obesity.* [www.heartforum.org.uk/downloads/Overweight_ObesityToolkit_Full.pdf]
8. Shaw KA, O'Rourke P, Del Mar C, Kenardy J: **Psychological interventions for overweight or obesity.** *Cochrane Database of Systematic Reviews* 2005, **Issue 2**:Art. No.: CD003818. doi:10.1002/14651858.CD003818.pub2.
9. Rossner S, Hammarstrand M, Hemmingsson E, Neovius M, Johansson K: **Long-term weight loss and weight-loss maintenance strategies.** *Obes Rev* 2008, **9**(6):624–630.
10. Wood W, Neal DT: **A new look at habits and the habit-goal interface.** *Psychol Rev* 2007, **114**(4):843–863.
11. Lally P, Van Jaarsveld CHM, Potts HWW, Wardle J: **How are habits formed: Modelling habit formation in the real world.** *Eur J Soc Psychol* 2010, **40** (6):998–1009.

For peer review only

12. de Bruijn GJ, Kroeze W, Oenema A, Brug J: **Saturated fat consumption and the Theory of Planned Behaviour: exploring additive and interactive effects of habit strength.** *Appetite* 2008, **51**:318–323.
13. Kremers SP, Brug J: **Habit strength of physical activity and sedentary behavior among children and adolescents.** *Pediatr Exerc Sci* 2008, **20** (1):5–14.
14. de Bruijn GJ, Kremers SP, Singh A, van den Putte B, van Mechelen W: **Adult active transportation: adding habit strength to the theory of planned behavior.** *Am J Prev Med* 2009, **36**(3):189–194.
15. Lally P, Chipperfield A, Wardle J: **Healthy habits: efficacy of simple advice on weight control based on a habit-formation model.** *Int J Obes* 2008, **32**(4):700–707.
16. Osborn DP, Levy G, Nazareth I, Petersen I, Islam A, King MB: **Relative risk of cardiovascular and cancer mortality in people with severe mental illness from the United Kingdom's General Practice Rsearch Database.** *Arch Gen Psychiatry* 2007, **64(2)**:242–249.
17. Roe L, Strong C, Whiteside C, Neil A, Mant D: **Dietary intervention in primary care: validity of the DINE method for diet assessment.** *Fam Pract* 1994, **11**(4):375–381.
18. Besson H, Brage S, Jakes RW, Ekelund U, Wareham NJ: **Estimating physical activity energy expenditure, sedentary time, and physical activity intensity by self-report in adults.** *Am J Clin Nutr* 2010, **91** (1):106–114.
19. Verplanken B, Orbell S: **Reflections on past behavior: A self-report index of habit strength.** *J Appl Soc Psychol* 2003, **33**(6):1313–1330.
20. Kind P: ***Quality of life and PharmacoEconomics in clinical trials***. In *The EuroQol instrument: An index of health related quality of life*. 2nd edition. Edited by Spiker B. Philadelphia: Lippincott-Raven Publishers; 1996.
21. Clark MM, Abrams DB, Niaura RS, Eaton CA, Rossi JS: **Self-efficacy in weight management.** *J Consult Clin Psychol* 1991, **59**(5):739–744.
22. van Strien T, Frijters JER, Bergers GPA, Defares PB: **The Dutch Eating Behavior Questionnaire (Debq) for Assessment of Restrained, Emotional, and External Eating Behavior.** *Int J Eat Disorder* 1986, **5**(2):295–315.
23. Carey KB, Neal DJ, Collins SE: **A psychometric analysis of the self-regulation questionnaire.** *Addict Behav* 2004, **29**(2):253–260.
24. Carlson JA, Sallis JF, Wagner N, Calfas KJ, Patrick K, Groesz LM: **Brief physical activity-related psychosocial measures: reliability and construct validity.** *J Phys Activ Health* 2012, **9**(8): Online first.
25. Westenhoefer J, von Falck B, Stellfeldt A, Fintelmann S: **Behavioural correlates of successful weight reduction over 3y. Results from the Lean Habits Study.** *Int J Obes* 2004, **28**(2):334–335.
26. Gorin AA, Phelan S, Wing RR, Hill JO: **Promoting long-term weight control: does dieting consistency matter?** *Int J Obes* 2004, **28**(2):278–281.
27. Mulvihill C, Quigley R: *The management of obesity and overweight: An analysis of reviews of diet, physical activity and behavioural approaches: Evidence briefing*. London: Health Development Agency; 2003.
28. Wing RR, Hill JO: **Successful weight loss maintenance.** *Annu Rev Nutr* 2001, **21**:323–341.
29. Department of Health: *At least five a week. Evidence on the impact of physical activity and its relationship to health. A report from the chief medical officer*. London: Department of Health; 2004.
30. Saris WH, Blair SN, van Baak MA, Eaton SB, Davies PS, Di Pietro L, Fogelholm M, Rissanen A, Schoeller D, Swinburn B, *et al*: **How much physical activity is enough to prevent unhealthy weight gain? Outcome of the IASO 1st Stock Conference and consensus statement.** *Obes Rev* 2003, **4**(2):101–114.
31. Tudor-Locke C, Bassett DR Jr: **How many steps/day are enough? Preliminary pedometer indices for public health.** *Sports Med* 2004, **34**(1):1–8.
32. Berteus Forslund H, Torgerson JS, Sjostrom L, Lindroos AK: **Snacking frequency in relation to energy intake and food choices in obese men and women compared to a reference population.** *Int J Obes* 2005, **29** (6):711–719.
33. Food Standards Agency: *Concept Testing of Alternative Labelling of Healthy/ Less Healthy Foods*. London: Food Standards Agency; 2004.
34. Young LR, Nestle M: **Expanding portion sizes in the US marketplace: implications for nutrition counseling.** *J Am Diet Assoc* 2003, **103**(2): 231–234.
35. Ello-Martin JA, Ledikwe JH, Rolls BJ: **The influence of food portion size and energy density on energy intake: implications for weight management.** *Am J Clin Nutr* 2005, **82**(1 Suppl):236S–241S.
36. Hu FB, Li TY, Colditz GA, Willett WC, Manson JE: **Television watching and other sedentary behaviors in relation to risk of obesity and type 2 diabetes mellitus in women.** *J Am Med Assoc* 2003, **289**(14):1785–1791.
37. Mummery WK, Schofield GM, Steele R, Eakin EG, Brown WJ: **Occupational sitting time and overweight and obesity in Australian workers.** *Am J Prev Med* 2005, **29**(2):91–97.
38. Nielsen SJ, Popkin BM: **Changes in beverage intake between 1977 and 2001.** *Am J Prev Med* 2004, **27**(3):205–210.
39. Schulze MB, Manson JE, Ludwig DS, Colditz GA, Stampfer MJ, Willett WC, Hu FB: **Sugar-sweetened beverages, weight gain, and incidence of type 2 diabetes in young and middle-aged women.** *J Am Med Assoc* 2004, **292**(8):927–934.
40. Stroebele N, de Castro JM: **Television viewing is associated with an increase in meal frequency in humans.** *Appetite* 2004, **42**(1):111–113.
41. NHS: *5 a day*. [http://www.5aday.nhs.uk/professionals/default.aspx]
42. Roberts C, Roberts SA: **Design and analysis of clinical trials with clustering effects due to treatment.** *Clin trials* 2005, **2**(2):152–162.
43. Campbell M, Grimshaw J, Steen N: **Sample size calculations for cluster randomised trials. Changing Professional Practice in Europe Group (EU BIOMED II Concerted Action).** *J Health Serv Res Policy* 2000, 5(1):12–16.
44. Eldridge SM, Ashby D, Kerry S: **Sample size for cluster randomized trials: effect of coefficient of variation of cluster size and analysis method.** *Int J Epidemiol* 2006, **35(5)**:1292–1300.
45. Molenberghs G: *Kenward MG: Missing Data in Clinical Studies*. Chichester: John Wiley and Sons Ltd; 2007.
46. NICE: *Guide to the methods of technology appraisal*. [http://nice.org.uk/ niceMedia/ pdf/TAP_Methods.pdf]
47. Frost GS, Lyons GF: **Obesity impacts on general practice appointments.** *Obes Res* 2005, **13**(8):1442–1449.
48. NICE: *Obesity guideline*. [http://www.nice.org.uk/CG043fullguideline]
49. Michie S: **Talking to primary care patients about weight: a study of GPs and practice nurses in the UK.** *Psychol Health Med* 2007, **12**(5):521–525.
50. Epstein L, Ogden J: **A qualitative study of GPs' views of treating obesity.** *Br J Gen Pract* 2005, **55**(519):750–754.
51. Avenell A, Broom J, Brown TJ, Poobalan A, Aucott L, Stearns SC, Smith WC, Jung RT, Campbell MK, Grant AM: **Systematic review of the long-term effects and economic consequences of treatments for obesity and implications for health improvement.** *Health Technol Assess* 2004, **8**(21):1–182.
52. Douketis JD, Macie C, Thabane L, Williamson DF: **Systematic review of long-term weight loss studies in obese adults: clinical significance and applicability to clinical practice.** *Int J Obes* 2005, **29**(10):1153–1167.
53. Marinilli Pinto A, Gorin AA, Raynor HA, Tate DF, Fava JL, Wing RR: **Successful weight-loss maintenance in relation to method of weight loss.** *Obesity* 2008, **16**(11):2456–2461.

doi:10.1186/1471-2458-12-667
Cite this article as: Beeken *et al.*: Study protocol for the 10 Top Tips (10TT) Trial: Randomised controlled trial of habit-based advice for weight control in general practice. *BMC Public Health* 2012 **12**:667.

Submit your next manuscript to BioMed Central and take full advantage of:

- **Convenient online submission**
- **Thorough peer review**
- **No space constraints or color figure charges**
- **Immediate publication on acceptance**
- **Inclusion in PubMed, CAS, Scopus and Google Scholar**
- **Research which is freely available for redistribution**

Submit your manuscript at
www.biomedcentral.com/submit

BioMed Central

For peer review only

VALUE IN HEALTH 16 (2013) e1–e5

Available online at www.sciencedirect.com

SciVerse ScienceDirect

ELSEVIER

journal homepage: www.elsevier.com/locate/jval

VALUE IN HEALTH ONLINE

Consolidated Health Economic Evaluation Reporting Standards (CHEERS) Statement

Don Husereau, BScPharm, MSc[1,2,3,], Michael Drummond, PhD[4], Stavros Petrou, MPhil, PhD[5], Chris Carswell, MSc, MRPharmS[6], David Moher, PhD[7], Dan Greenberg, PhD[8,9], Federico Augustovski, MD, MSc, PhD[10,11], Andrew H. Briggs, MSc (York), MSc (Oxon), DPhil (Oxon)[12], Josephine Mauskopf, PhD[13], Elizabeth Loder, MD, MPH[14], on behalf of the CHEERS Task Force*

[1]*Institute of Health Economics, Edmonton, Canada;* [2]*Department of Epidemiology and Community Medicine. University of Ottawa, Ottawa, ON, Canada;* [3]*University for Health Sciences, Medical Informatics and Technology, Hall in Tirol, Austria;* [4]*Centre for Health Economics, University of York, Heslington, York, UK;* [5]*Warwick Medical School, University of Warwick, Coventry, UK;* [6]*Pharmacoeconomics, Adis International, Auckland, New Zealand;* [7]*Clinical Epidemiology, Program, Ottawa Hospital Research Institute, Ottawa, ON, Canada;* [8]*Faculty of Health Sciences, Department of Health Systems Management, Ben-Gurion University of the Negev, Beer-Sheva, Israel;* [9]*Center for the Evaluation of Value and Risk in Health, Tufts Medical Center, Boston, MA, USA;* [10]*Health Economic Evaluation and Technology Assessment, Institute for Clinical Effectiveness and Health Policy (IECS), Buenos Aires, Argentina;* [11]*Universidad de Buenos Aires, Buenos Aires, Argentina;* [12]*Institute of Health & Wellbeing, University of Glasgow, Glasgow, Scotland;* [13]*RTI Health Solutions, Research Triangle Park, NC, USA;* [14]*Brigham and Women's/Faulkner Neurology, Faulkner Hospital, Boston, MA, USA*

ABSTRACT

Economic evaluations of health interventions pose a particular challenge for reporting. There is also a need to consolidate and update existing guidelines and promote their use in a user friendly manner. The Consolidated Health Economic Evaluation Reporting Standards (CHEERS) statement is an attempt to consolidate and update previous health economic evaluation guidelines efforts into one current, useful reporting guidance. The primary audiences for the CHEERS statement are researchers reporting economic evaluations and the editors and peer reviewers assessing them for publication.

The need for new reporting guidance was identified by a survey of medical editors. A list of possible items based on a systematic review was created. A two round, modified Delphi panel consisting of representatives from academia, clinical practice, industry, government, and the editorial community was conducted. Out of 44 candidate items, 24 items and accompanying recommendations were developed. The recommendations are contained in a user friendly, 24 item checklist. A copy of the statement, accompanying checklist, and this report can be found on the ISPOR Health Economic Evaluations Publication Guidelines Task Force website: (www.ispor.org/TaskForces/EconomicPubGuidelines.asp).

We hope CHEERS will lead to better reporting, and ultimately, better health decisions. To facilitate dissemination and uptake, the CHEERS statement is being co-published across 10 health economics and medical journals. We encourage other journals and groups, to endorse CHEERS. The author team plans to review the checklist for an update in five years.
Keywords: biomedical research/methods, biomedical research/standards, costs and cost analysis, guidelines as topic/standards, humans, publishing/standards.

© 2013, International Society for Pharmacoeconomics and Outcomes Research (ISPOR). Published by Elsevier HS Journals, Inc.

Health economic evaluations are conducted to inform resource allocation decisions. Economic evaluation has been defined as "the comparative analysis of alternative courses of action in terms of both their costs and their consequences." [1] All economic evaluations assess costs, but approaches to measuring and valuing the consequences of health interventions may differ (see Box 1).

Economic evaluations have been widely applied in health policy, including the assessment of prevention programmes (such as vaccination, screening, and health promotion), diagnostics, treatment interventions (such as drugs and surgical procedures), organisation of care, and rehabilitation. Economic evaluations are increasingly being used for decision making and are an important component of programmes for health technology assessment internationally [2].

Reporting Challenges and Shortcomings in Health Economic Evaluations

Compared with clinical studies, which report the consequences of an intervention only, economic evaluations require more reporting space for additional items, such as resource use, costs, preference

This article is a joint publication by Value in Health, *BJOG: An International Journal of Obstetrics and Gynaecology, BMC Medicine, BMJ, Clinical Therapeutics, Cost Effectiveness and Resource Allocation, The European Journal of Health Economics, International Journal of Technology Assessment in Health Care, Journal of Medical Economics,* and *Pharmacoeconomics*. Each publisher holds its own copyright.

* Address correspondence to: Don Husereau, 879 Winnington Avenue, Ottawa, ON, Canada K2B 5C4.
E-mail: donh@donhusereau.com.

1098-3015/$36.00 – see front matter Copyright © 2013, International Society for Pharmacoeconomics and Outcomes Research (ISPOR). Published by Elsevier HS Journals, Inc.
http://dx.doi.org/10.1016/j.jval.2013.02.010

Box 1 – Forms of economic evaluation.

Specific forms of analysis reflect different approaches to evaluating the consequences of health interventions. Health consequences may be estimated from a single analytical (experimental or non-experimental) study, a synthesis of studies, mathematical modelling, or a combination of modelling and study information.

Cost consequences analysis examines costs and consequences without attempting to isolate a single consequence or aggregate consequences into a single measure

Cost minimisation analysis (CMA)—The consequences of compared interventions are required to be equivalent, and only relative costs are compared

Cost effectiveness analysis (CEA) measures consequences in natural units, such as life years gained, disability days avoided, or cases detected. In a variant of CEA, often called cost utility analysis, consequences are measured in terms of preference-based measures of health, such as quality adjusted life years or disability adjusted life years.

Cost benefit analysis—Consequences are valued in monetary units.

Readers should be aware that an economic evaluation might be referred to as a "cost effectiveness analysis" or "cost benefit analysis" even if it does not strictly adhere to the definitions above. Multiple forms may also exist within a single evaluation. Different forms of analysis provide unique advantages or disadvantages for decision making. The Consolidated Health Economic Evaluation Reporting Standards (CHEERS) statement can be used with any form of economic evaluation.

related information, and cost effectiveness results. This creates challenges for editors, reviewers, and those who wish to scrutinise a study's findings [3]. There is evidence that the quality of reporting of economic evaluations varies widely and could potentially benefit from improved quality assurance mechanisms [4,5].

With the increasing number of publications available, and opportunity costs from decisions based on misleading study findings, transparency and clarity in reporting are important. In addition, outside of economic evaluations conducted alongside clinical trials, there are no widespread mechanisms for warehousing economic evaluation data to allow for independent interrogation, such as ethics review proceedings, regulator dossiers, or study registries. Instead, independent analysis may rely on the record keeping of individual investigators.

Even if measures to promote transparency exist, such as registries, biomedical journal editors have increasingly promoted and endorsed the use of reporting guidelines. Endorsement of guidelines by journals for randomised controlled trials has been shown to improve reporting [6]. The combination of the risk of making costly decisions due to poor reporting with the lack of mechanisms that promote accountability makes transparency in reporting economic evaluations especially important and a primary concern among journal editors and decision makers [3,7].

Aim and Scope

The aim of the Consolidated Health Economic Evaluation Reporting Standards (CHEERS) statement is to provide recommendations, in the form of a checklist, to optimise reporting of health economic evaluations. The need for a contemporary reporting guidance for economic evaluations was recently identified by researchers and biomedical journal editors [8]. The CHEERS statement attempts to consolidate and update previous efforts [9–20] into a single useful reporting guidance.

The primary audiences for the CHEERS statement are researchers reporting economic evaluations and the editors and peer reviewers evaluating their publication potential. We hope the statement (which consists of a 24 item checklist and accompanying recommendations on the minimum amount of information to be included when reporting economic evaluations) is a useful and practical tool for these audiences and will improve reporting and, in turn, health and healthcare decisions. To best understand and apply the recommendations contained within the statement, we encourage readers to access the Explanation and Elaboration Report [21].

Development of the CHEERS Statement

The statement was developed by a task force supported by the International Society for Pharmacoeconomics and Outcomes Research (ISPOR), as part of a broader initiative to facilitate and encourage the interchange of expert knowledge and develop best practices. The CHEERS Task Force members were chosen by the chair of the task force primarily based on their longstanding academic expertise and contribution to the multidisciplinary field of health economic evaluation. In addition to four members of the task force with doctorates in economics and its subdiscipline of health economics (AHB, MD, JM, SP), members included experts in health technology assessment and decision making (FA, AHB, DH, MD, JM) and in clinical epidemiology and biostatistics (AHB, EL, DM), those in active clinical practice (EL, FA), and those with previous experience in reporting guideline development (MD, DM). All members are researchers in applied health and health policy, with five members currently serving as editors for journals in the field (AHB, CC, MD, DG, EL).

The CHEERS Task Force followed current recommendations for developing reporting guidelines [22]. Briefly, the need for new guidance was first identified through a survey of members of the World Association of Medical Editors. Of the 6% (55/965) who responded, 91% (n=50) indicated they would use a standard if one were widely available [8]. Next, published checklists or guidance documents related to reporting economic evaluations were identified from a systematic review and survey of task force members [23]. Both of these activities were used to create a preliminary list of items to include when reporting economic evaluations. Recommendations of the minimum set of reporting items were then developed through a modified Delphi panel process. Forty eight individuals identified by the task force with broad geographical representation and representing academia, biomedical journal editors, the pharmaceutical industry, government decision makers, and those in clinical practice were invited to participate. Thirty seven agreed to participate. Participants were asked to score importance on a Likert scale and the average scores, weighted by each individual's confidence in ability to score, were then used to rank items. A cut-off point was applied to the ranked list to determine the minimum number of items important for reporting.

The CHEERS statement recommendations have been independently reviewed and subsequently revised by task force members. The recommendations are entirely those of the task force—the sponsors of the study had no role in study design, data analysis, data interpretation, or writing of the final recommendations. A more complete description of the methods and findings of the Delphi panel are found in the larger explanation and elaboration document [21].

Checklist Items

The final recommendations are subdivided into six main categories: (1) title and abstract; (2) introduction; (3) methods; (4) results;

VALUE IN HEALTH 16 (2013) e1–e5

e3

Table 1 – CHEERS checklist—Items to include when reporting economic evaluations of health interventions.

Section/item	Item No	Recommendation	Reported on page No/ ~~line No~~
**Title and abstract**			
Title	1	Identify the study as an economic evaluation or use more specific terms such as "cost-effectiveness analysis", and describe the interventions compared.	1
Abstract	2	Provide a structured summary of objectives, perspective, setting, methods (including study design and inputs), results (including base case and uncertainty analyses), and conclusions.	2
**Introduction**			
Background and objectives	3	Provide an explicit statement of the broader context for the study. Present the study question and its relevance for health policy or practice decisions.	4-6
**Methods**			
Target population and subgroups	4	Describe characteristics of the base case population and subgroups analysed, including why they were chosen.	7
Setting and location	5	State relevant aspects of the system(s) in which the decision(s) need(s) to be made.	7
Study perspective	6	Describe the perspective of the study and relate this to the costs being evaluated.	8
Comparators	7	Describe the interventions or strategies being compared and state why they were chosen.	7
Time horizon	8	State the time horizon(s) over which costs and consequences are being evaluated and say why appropriate.	8
Discount rate	9	Report the choice of discount rate(s) used for costs and outcomes and say why appropriate.	8
Choice of health outcomes	10	Describe what outcomes were used as the measure(s) of benefit in the evaluation and their relevance for the type of analysis performed.	7
Measurement of effectiveness	11a	*Single study-based estimates:* Describe fully the design features of the single effectiveness study and why the single study was a sufficient source of clinical effectiveness data.	10
	11b	*Synthesis-based estimates:* Describe fully the methods used for identification of included studies and synthesis of clinical effectiveness data.	N/A
Measurement and valuation of preference based outcomes	12	If applicable, describe the population and methods used to elicit preferences for outcomes.	N/A
Estimating resources and costs	13a	*Single study-based economic evaluation:* Describe approaches used to estimate resource use associated with the alternative interventions. Describe primary or secondary research methods for valuing each resource item in terms of its unit cost. Describe any adjustments made to approximate to opportunity costs.	10
	13b	*Model-based economic evaluation:* Describe approaches and data sources used to estimate resource use associated with model health states. Describe primary or secondary research methods for valuing each resource item in terms of its unit cost. Describe any adjustments made to approximate to opportunity costs.	N/A
Currency, price date, and conversion	14	Report the dates of the estimated resource quantities and unit costs. Describe methods for adjusting estimated unit costs to the year of reported costs if necessary. Describe methods for converting costs into a common currency base and the exchange rate.	8-10
Choice of model	15	Describe and give reasons for the specific type of decision-analytical model used. Providing a figure to show model structure is strongly recommended.	N/A
Assumptions	16	Describe all structural or other assumptions underpinning the decision-analytical model.	N/A
Analytical methods	17	Describe all analytical methods supporting the evaluation. This could include methods for dealing with skewed, missing, or censored data; extrapolation methods; methods for pooling data; approaches to validate or make adjustments (such as half cycle corrections) to a model; and methods for handling population heterogeneity and uncertainty.	10

Table 1 – *continued*

Section/item	Item No	Recommendation	Reported on page No/~~line~~ No
**Results**			
Study parameters	18	Report the values, ranges, references, and, if used, probability distributions for all parameters. Report reasons or sources for distributions used to represent uncertainty where appropriate. Providing a table to show the input values is strongly recommended.	11-12
Incremental costs and outcomes	19	For each intervention, report mean values for the main categories of estimated costs and outcomes of interest, as well as mean differences between the comparator groups. If applicable, report incremental cost-effectiveness ratios.	13
Characterising uncertainty	20a	*Single study-based economic evaluation:* Describe the effects of sampling uncertainty for the estimated incremental cost and incremental effectiveness parameters, together with the impact of methodological assumptions (such as discount rate, study perspective).	14-15
	20b	*Model-based economic evaluation:* Describe the effects on the results of uncertainty for all input parameters, and uncertainty related to the structure of the model and assumptions.	N/A
Characterising heterogeneity	21	If applicable, report differences in costs, outcomes, or cost-effectiveness that can be explained by variations between subgroups of patients with different baseline characteristics or other observed variability in effects that are not reducible by more information.	N/A
**Discussion**			
Study findings, limitations, generalisability, and current knowledge	22	Summarise key study findings and describe how they support the conclusions reached. Discuss limitations and the generalisability of the findings and how the findings fit with current knowledge.	15-17
**Other**			
Source of funding	23	Describe how the study was funded and the role of the funder in the identification, design, conduct, and reporting of the analysis. Describe other non-monetary sources of support.	1
Conflicts of interest	24	Describe any potential for conflict of interest of study contributors in accordance with journal policy. In the absence of a journal policy, we recommend authors comply with International Committee of Medical Journal Editors recommendations.	17.

For consistency, the CHEERS statement checklist format is based on the format of the CONSORT statement checklist.

(5) discussion; and (6) other. The recommendations are contained in a user friendly, 24 item checklist (Table 1) to aid users who wish to follow them. A copy of the checklist can also be found on the CHEERS Task Force website. (www.ispor.org/TaskForces/EconomicPubGuidelines.asp). In order to encourage dissemination and use of a single international standard for reporting, the task force approached 14 journals identified as either the largest publishers of economic evaluations or widely read by the medical and research community. Thirteen journals responded, and 10 expressed their ability and interest in endorsing this guidance. The CHEERS statement is being simultaneously published in BMC Medicine, BMJ, *BJOG: An International Journal of Obstetrics and Gynaecology, Clinical Therapeutics, Cost Effectiveness and Resource Allocation, The European Journal of Health Economics, International Journal of Technology Assessment in Health Care, Journal of Medical Economics, Pharmacoeconomics*, and *Value in Health*. To facilitate wider dissemination and uptake of this reporting guidance, we encourage other journals and groups to consider endorsing CHEERS.

Concluding Remarks

As the number of published health economic evaluations continues to grow, we believe more transparent and complete reporting of methods and findings will be increasingly important to facilitate interpretation and comparison of studies. We hope the CHEERS statement, consisting of recommendations in a 24 item checklist, will be viewed as an effective consolidation and update of previous efforts and serve as a starting point for standard reporting going forward.

We believe the CHEERS statement represents a considerable expansion over previous efforts. The strength of our approach is that it was developed in accordance with current recommendations for the development of reporting guidelines, using an international and multidisciplinary team of editors and content experts in economic evaluation and reporting [22]. Similar to the approach taken with other widely accepted guidelines, we have defined a minimum set of criteria though a modified Delphi technique and have translated these into recommendations, an explanatory document with explanations, and a checklist. Unlike some previous reporting guidance for economic evaluation, we have also made every effort to be neutral about the conduct of economic evaluation, allowing analysts the freedom to choose different methods.

There may be several limitations to our approach. A larger Delphi panel with a different composition could have led to a different final set of recommendations [24]. Some less common

approaches and contexts (such as public health, developing countries, and system dynamic models) for conducting health economic evaluation may not be well represented by our sample of experts. Additionally, like many Delphi panel processes, we based decisions to reject or accept criteria on arbitrary levels of importance. However, we feel the group recruited to create the statement is sufficiently knowledgeable of the more common applications of economic evaluation, and the rules used to select criteria were created a priori and are consistent with previous efforts.

We believe it will be important to evaluate the effects of implementation of this statement and checklist on reporting in future economic evaluations. As methods for the conduct of economic evaluation continue to evolve, it will also be important to revisit or extend the guidance. The CHEERS Task Force feels that this statement should be reviewed for updating five years from its release.

Acknowledgements

Elizabeth Loder—*BMJ* clinical epidemiology editor—played no part in the peer review or decision making of this paper at the editorial level, and contributed solely as an author.

The International Society for Pharmacoeconomics and Outcomes Research (ISPOR) Health Economic Evaluation Publication Guidelines—CHEERS Task Force acknowledge the support of Elizabeth Molsen; Donna Rindress, who provided the initial leadership for this effort; and the reviewers and Delphi panel participants, who are named in the larger explanation and elaboration document and on the CHEERS Task Force website.

Contributors: All authors provided a substantial contribution to the design and interpretation of the protocol and guidance, as well as writing sections of drafts, revising based on comments received, and approving the final version. DH conducted the analysis of Delphi panel responses, drafted and revised the protocol and the drafts of this paper, and is the guarantor for the study.

Competing interests: All authors have completed the ICMJE uniform disclosure form at www.icmje.org/coi_disclosure.pdf and declare: FA served as board member for the study funder; FA, AHB, CC, MD, DG, DH, EL, JM, and SP were provided support for travel to a face-to-face meeting to discuss the contents of the report; FA and MD have received payment from the study sponsor for serving as co-editors for *Value in Health*; no other relationships or activities that could appear to have influenced the submitted work.

Source of financial support: All CHEERS Task Force members are volunteers. Support for this initiative was provided by the International Society for Pharmacoeconomics and Outcomes Research.

REFERENCES

[1] Drummond MF, Sculpher MJ, Torrance G, O'Brien J, Stoddart GL. Methods for the economic evaluation of health care programmes. 3rd ed. Oxford University Press, 2005.

[2] Drummond MF, Schwartz JS, Jönsson B, et al. Key principles for the improved conduct of health technology assessments for resource allocation decisions. Int J Technol Assess Health Care 2008;24:244–58.

[3] Rennie D, Luft HS. Pharmacoeconomic analyses. JAMA 2000;283:2158–60.

[4] Neumann PJ, Stone PW, Chapman RH, et al. The quality of reporting in published cost-utility analyses, 1976-1997. Ann Intern Med 2000;132:964.

[5] Rosen AB, Greenberg D, Stone PW, et al. Quality of abstracts of papers reporting original cost-effectiveness analyses. Med Decis Making 2005;25:424–8.

[6] Turner L, Shamseer L, Altman DG, et al. Does use of the CONSORT Statement impact the completeness of reporting of randomised controlled trials published in medical journals? A Cochrane review. Syst Rev 2012;1:60.

[7] Drummond MF. A reappraisal of economic evaluation of pharmaceuticals. Science or marketing? Pharmacoeconomics 1998;14:1–9.

[8] McGhan WF, Al M, Doshi JA, Kamae I, et al. The ISPOR Good Practices for Quality Improvement of Cost-Effectiveness Research Task Force report. Value Health 2009;12:1086–99.

[9] Task Force on Principles for Economic Analysis of Health Care Technology. Economic analysis of health care technology. A report on principles. Ann Intern Med 1995;123:61–70.

[10] Drummond MF, Jefferson TO. Guidelines for authors and peer reviewers of economic submissions to the BMJ. BMJ 1996;313:275–83.

[11] Gold MR. Cost-effectiveness in health and medicine. Oxford University Press, 1996.

[12] Siegel JE, Weinstein MC, Russell LB, Gold MR. Recommendations for reporting cost-effectiveness analyses. Panel on Cost-Effectiveness in Health and Medicine. JAMA 1996;276:1339–41.

[13] Nuijten MJ, Pronk MH, Brorens MJ, et al. Reporting format for economic evaluation: Part II: Focus on modelling studies. Pharmacoeconomics 1998;14:259–68.

[14] Vintzileos AM, Beazoglou T. Design, execution, interpretation, and reporting of economic evaluation studies in obstetrics. Am J Obstet Gynecol 2004;191:1070–6.

[15] Drummond M, Manca A, Sculpher M. Increasing the generalizability of economic evaluations: recommendations for the design, analysis, and reporting of studies. Int J Technol Assess Health Care 2005;21:165–71.

[16] Ramsey S, Willke R, Briggs A, et al. Good research practices for cost-effectiveness analysis alongside clinical trials: the ISPOR RCT-CEA Task Force report. Value Health 2005;8:521–33.

[17] Goetghebeur MM, Wagner M, Khoury H, et al. Evidence and value: impact on decisionmaking—the EVIDEM framework and potential applications. BMC Health Serv Rev 2008;8:270.

[18] Davis JC, Robertson MC, Comans T, Scuffham PA. Guidelines for conducting and reporting economic evaluation of fall prevention strategies. Osteoporos Int 2010;22:2449–59.

[19] Petrou S, Gray A. Economic evaluation using decision analytical modelling: design, conduct, analysis, and reporting. BMJ 2011;342:d1766.

[20] Petrou S, Gray A. Economic evaluation alongside randomised controlled trials: design, conduct, analysis, and reporting. BMJ 2011;342:d1548.

[21] Husereau D, Drummond M, Petrou S, et al. Consolidated Health Economic Evaluation Reporting Standards (CHEERS)—explanation and elaboration: a report of the ISPOR Health Economic Evaluations Publication Guidelines Task Force. Value Health 2013;16:231–50.

[22] Moher D, Schulz KF, Simera I, Altman DG. Guidance for developers of health research reporting guidelines. PLoS Med 2010;7:e1000217.

[23] Moher D, Weeks L, Ocampo M, et al. Describing reporting guidelines for health research: a systematic review. J Clin Epi 2011;64:718–42.

[24] Campbell SM, Hann M, Roland MO, et al. The effect of panel membership and feedback on ratings in a two-round Delphi survey: results of a randomized controlled trial. Med Care 1999;37:964–8.

BMJ Open

Cost-effectiveness of habit-based advice for weight control versus usual care in general practice in the Ten Top Tips (10TT) Trial: economic evaluation based on a randomised controlled trial

Journal:	*BMJ Open*
Manuscript ID	bmjopen-2017-017511.R1
Article Type:	Research
Date Submitted by the Author:	02-Aug-2017
Complete List of Authors:	Patel, Nishma; UCL, Department of Applied Health Research<br>Beeken, Rebecca; University College London, Dept. of Epidemiol<br>Leurent, Baptiste; London School of Hygiene and Tropical Medicine, Department of Infectious Disease Epidemiology<br>Omar, Rumana; University College London,<br>Nazareth, Irwin; UCL, Primary Care and Population Health<br>Morris, Stephen; University College London, Department of Applied Health Research
**Primary Subject Heading**:	Health economics
Secondary Subject Heading:	Public health, General practice / Family practice
Keywords:	HEALTH ECONOMICS, PRIMARY CARE, PUBLIC HEALTH

SCHOLARONE™
Manuscripts

For peer review only

Cost-effectiveness of habit-based advice for weight control versus usual care in general practice in the Ten Top Tips (10TT) Trial: economic evaluation based on a randomised controlled trial

Nishma Patel[1], Rebecca J. Beeken[2,3], Baptiste Leurent[4], Rumana Z. Omar[5], Irwin Nazareth[6] and Stephen Morris[1]

[1] Department of Applied Health Research, University College London, Gower Street, London, WC1E 6BT, UK

[2] Department of Behavioural Science & Health, University College London, Gower Street, London, WC1E 6BT, UK

[3]Leeds Institute of Health Sciences, University of Leeds, Clarendon Way, Leeds, LS2 9NL

[4] London School of Hygiene and Tropical Medicine, Keppel Street, WC1E 7HT

[5] Department of Statistical Science, University College London, Gower Street, London, WC1E 6BT, UK

[6]Deparment of Primary Care & Population Health, UCL, Gower Street, London, WC1E 6BT

Correspondence to: Nishma Patel, Department of Applied Health Research, University College London WC1E 6BT, UK. Telephone: +44 2031 083027 (email: nishma.patel@ucl.ac.uk)

For peer review only - http://bmjopen.bmj.com/site/about/guidelines.xhtml

Abstract

Objective: Ten Top Tips (10TT) is a primary care led behavioural intervention, which aims to help adults reduce and manage their weight by following ten weight loss tips. The intervention promotes habit formation to encourage long-term behavioural changes. The aim of this study was to estimate the cost-effectiveness of 10TT in general practice from the perspective of the UK National Health Service.

Design: An economic evaluation was conducted alongside an individually randomised controlled trial.

Setting: 14 General Practitioner (GP) practices in England.

Participants: All patients were aged ≥18 years, with BMI ≥30kg/m^2. A total of 537 patients were recruited; 270 received the usual care offered by their practices and 267 received the 10TT intervention.

Outcomes measures: Health service use and quality-adjusted life years (QALY) were measured over two years. Analysis was conducted in terms of incremental Net Monetary Benefit (NMB), using non-parametric bootstrapping and multiple imputation.

Results: Over a two-year time horizon, mean costs and QALYs per patient in the 10TT group were £1889 (95% CI: £1522 to £2566) and 1.51 (95% CI: 1.44 to 1.58). The mean costs and QALYs for usual care were £1925 (95% CI: £1599 to £2251) and 1.51 (95% CI: 1.45 to 1.57), respectively. This generated a mean cost difference of -£36 (95%CI: -£511 to £440) and a mean QALY difference of 0.0007 (95%CI: -0.080 to 0.082). The incremental NMB for 10TT versus usual care was £49 (95%CI: -£1709 to £1800) at a maximum willingness to pay for a QALY of £20,000. 10TT had a 52% probability of being cost-effective at this threshold.

For peer review only - http://bmjopen.bmj.com/site/about/guidelines.xhtml

For peer review only

Conclusions: Costs and QALYs for 10TT were not significantly different from usual care and therefore 10TT is as cost-effective as usual care. There was no evidence to recommend nor advice against offering 10TT to obese patients in general practices based on cost-effectiveness considerations.

Trial Registration: ISRCTN registry ISRCTN16347068

Article summary

Strengths and limitations of this study

- The analysis is based on a large multicentre randomised trial with detailed information on resource use and utility values for a median follow-up period of two years.
- Individual data on standard weight-loss interventions received by the participant were not recorded.
- The analysis took a UK NHS/PSS perspective but a wider perspective (e.g., societal) could have been taken, including costs to patients and families.
- The time horizon was two years but could have taken a longer time horizon.
- Missing NHS resource use and utility data was accounted for using multiple imputation.

For peer review only

For peer review only - http://bmjopen.bmj.com/site/about/guidelines.xhtml

66 **Introduction**

67 The worldwide prevalence of obesity nearly doubled between 1980 and 2008. Based on
68 recent estimates in the European Union, overweight affects 30-70% and obesity affects 10-
69 30% of adults(1). For both women and men aged 18 years and over, the lowest proportions
70 of the population considered to be obese in 2008 were observed in Romania (8.0 % for
71 women and 7.6 % for men), Italy (9.3 % and 11.3 %), Bulgaria (11.3 % and 11.6 %) and
72 France (12.7 % and 11.7 %). The highest proportions of women who were obese were
73 recorded in the UK (23.9 %), Malta (21.1 %), Latvia (20.9 %) and Estonia (20.5 %); and of
74 men in Malta (24.7 %), the UK (22.1 %) and Hungary (21.4 %)(2).

75 Health risks associated with obesity range from heart disease, stroke and type 2 diabetes to
76 all cancers, gall bladder disease and mortality(3). It is estimated that health problems
77 associated with being overweight or obese cost the National Health Service (NHS) in England
78 more than £5 billion every year(4).

79 Non-surgical interventions for weight loss are available and include dietary advice, physical
80 activity and behaviour modification. These can be accessed mainly through primary care.
81 Despite considerable investment in such interventions, there is limited evidence about their
82 cost-effectiveness(5-9).

83 Ten Top Tips (10TT) was a simple leaflet-based intervention for patients in primary care
84 designed to help with weight loss. The underpinning theory behind 10TT was habit
85 formation. Psychological research shows that repetition of a simple action in a consistent
86 context leads, through associative learning, to the action being activated upon subsequent
87 exposure to those contextual cues(10). A small pilot trial consisting of 100 people showed

For peer review only - http://bmjopen.bmj.com/site/about/guidelines.xhtml

For peer review only

88 that those who received the Ten Top Tips leaflet lost significantly more weight than those who did not receive it(11). Although the absolute weight loss was modest, the intervention had the potential to be disseminated at minimal cost to large numbers simply by making the leaflet available. In response to this a large randomised trial was undertaken. Participants were given a leaflet, which provided weight loss tips based on scientific evidence on how to turn the tips into healthy habits, during a 30-minute appointment with a practice nurse. The ten healthy tips included: keeping to meal routines; reduced fat intake; walking off weight; packing a healthy snack; looking at food labels; taking caution with portion size; standing up; thinking about drinks; focusing on food; and, not forgetting 'five a day'. The 'five a day campaign is based on recommendation from the World Health Organisation (WHO) encouraging consumption of at least 400g of fruit and vegetables a day to lower the risk of serious health problems, such as heart disease, stroke and some cancers(12). Participants were asked to record their progress in a logbook. Using routinely collected data from primary care practices, the aim of the present study was to analyse the cost and cost-effectiveness of 10TT.

Methods

Trial background

The 10TT trial was a two-arm, individually randomised, controlled trial in which 537 obese men and women were enrolled. Practices across England (n=14) were recruited through the MRC General Practice Research Framework (GPRF). The majority were located in Southern England (n=9), with three located in the Midland and two in the North Recruitment occurred between August 2010 and October 2011. Participants were randomly assigned to 10TT (n=267) or usual care (n=270), and followed for up to two years. The primary outcome was

For peer review only - http://bmjopen.bmj.com/site/about/guidelines.xhtml

weight loss at three months. Secondary outcomes included BMI, waist circumference, the number of people achieving a 5% reduction in weight, clinical markers for potential co-morbidities (blood pressure, total cholesterol/low-density lipoprotein and blood glucose), and maintenance of weight loss over 24 months. At three months participants in the 10TT group lost significantly more weight than those receiving usual care (mean difference in weight change=-0·87kg, 95%CI -1·47 to -0·27, p=0·004). But this effect was not maintained over time (difference at 24 months = +0·75kg (95% CI: -0.73 to 2.24)).

; while weight loss in the usual care group was slower in the first 6 months, it continued until 18 months, whereas the 10TT group experienced a greater weight loss in the first 6 months, but did not lose any additional weight after this point.

Overview of economic evaluation

We undertook a cost-utility analysis to compare the costs and outcomes associated with 10TT and usual care. The outcome measure was quality-adjusted life years (QALYs), which combines length of life and quality of life, and is consistent with The National Institute for Health and Care Excellence (NICE) recommendations(13).The primary outcomes were the incremental costs and effects and the incremental net monetary benefit (NMB) of 10TT versus usual care. Costs were measured from a NHS and personal social services (PSS) perspective; since PSS costs were negligible, we focused on NHS costs. These were calculated in 2013/14 UK£. The time horizon for costs and effects was two years, reflecting the trial follow-up period. Extrapolation beyond this time period was not undertaken because the within-trial analysis found no evidence of significant differences in costs (-£36 (95%CI -£511 to £440, p-value 0.88) or outcomes between groups (0 (95% CI -0.080 to 0.082, p-value 0.93) at 24 months; two years was considered long enough to reflect all

For peer review only - http://bmjopen.bmj.com/site/about/guidelines.xhtml

important differences in costs or outcomes between treatments. Costs and outcomes in the second year were discounted at 3.5%(13).

Resource use and costs

Resource use was extracted for two years prior to randomisation and post randomisation on: the number and type of contacts with the GP (surgery visit, home visit, phone call); practice nurse contacts (practice visit, home visit, phone call); dietician visits; hospital outpatient visits; hospital inpatient stay; accident and emergency (A&E) visits; and outpatient services. These data were retrieved from GP records. Data on use of drugs included dose, type and frequency of administration. All resource use data was extracted by the practice nurse at participating centres from patient records.

Unit costs were attached to each resource item. The cost per visit to primary care (GP surgery visit, GP home visit, GP phone call, practice nurse visit, nurse home visit, practice nurse phone call and dietician visits) were taken from Personal Social Services Research Unit (PSSRU) estimates(14). The cost of inpatient episodes, outpatient visits and A&E visits were taken from the PSSRU and based on NHS Reference Costs(15). They included cost of medical staff, equipment, consumables, and diagnostics. The weighted average cost of inpatient stays was calculated combining the cost of inpatient long and short stay. Drug costs were obtained from British National Formulary(16).

The cost of the 10TT intervention included the following, which were valued using market prices: a logbook (£2.80); time patients spent with the practice nurse to introduce the programme (£20 for a 30-minute visit); wallet sized food label guidance (£0.09), and a 10TT leaflet (£0.05). The total cost of 10TT was £22.94 per participant. The cost of the food label

For peer review only - http://bmjopen.bmj.com/site/about/guidelines.xhtml

For peer review only

guidance was based on the total cost of printing (£1,392) divided by the number of labels printed (15,000). Similarly, the cost of 10TT leaflets was based on a total cost of printing (£1,540) divided by the number of leaflets printed (30,000).

All practices offered standard weight-loss interventions as part of usual care. Standard weight loss interventions consisted of a range of interventions including referrals to community programmes, gym prescription and referral to a dietician and/or psychologist, among others. While data were provided by practices on interventions typically offered to their patient, the uptake of these interventions was not recorded. In our base case, we made the conservative assumption (possibly biased against 10TT) that no patients received these interventions. In a sensitivity analysis, we included the costs of these interventions assuming each patient in the control group received the standard weight-loss intervention offered by the practice. Where single interventions were offered by a practice these were costed accordingly, based on the average cost of participating in Weight Watchers (assumed to cost £78.22 per patient) or Slimming World (£72.62 per patient). Where multiple interventions were offered by a practice these were costed based on the average cost of participating in the Size Down Programme at a cost of £93.48 per patient(17).

The unit costs for each cost component are shown in Table 1. Unit costs were multiplied by resource volume and summed across all cost components to obtain a total cost per patient.

Utilities and QALYs

Health utilities were based on the EuroQol standardised measure of health status, EQ-5D-3L descriptive system(18, 19). This is a five dimensional questionnaire (mobility, self-care, usual activities, pain and discomfort), with three levels in each dimension (severe problems, some

For peer review only - http://bmjopen.bmj.com/site/about/guidelines.xhtml

problems, no problems). Each EQ-5D state was converted into a single utility score based on valuations from the UK general population. Utility values of one represent full health, values of zero are equivalent to death, negative values represent states worse than death. The EQ-5D questionnaire was completed at baseline, three months, six months, 12 months, 18 months and 24 months. A utility profile was constructed for participants assuming a straight-line relation between their utility values at each measurement point. QALYs for every patient from baseline to two years were calculated as the area under the utility profile.

Dealing with Missing Data

There were missing data for NHS resource use and utility scores. Multiple imputation by multivariate normal regression was used to impute missing data(20, 21). The imputation method used an iterative Markov Chain Monte Carlo (MCMC) technique to simulate from the posterior predictive distribution of missing data, generating 20 imputed datasets(22). We imputed missing data for the following variables: weight (0.2%); BMI (0.2%); waist circumference (0.6%); EQ-5D at baseline (5%), 3 months (26%), 6 months (40%), 12 months (46%), 24 months (46%), NHS visits-; GP practice (28%), GP home (31%), GP phone calls (30%), practice nurse (27%), nurse home (31%), nurse phone calls (31%), extra nurse (30%), dietician (31%), hospital inpatient stay (31%), outpatient clinic (30%), A&E visits (30%)and other outpatient service visits (69%) (Table 1). Age, sex and study centre were included in the imputation model as additional explanatory variables. Imputations were undertaken using the –mi impute mvn(23)– command in Stata SE version 14(24).

Statistical analysis

For peer review only - http://bmjopen.bmj.com/site/about/guidelines.xhtml

Two sample t-tests were carried out for complete data. A linear regression model for imputed data was used to test for differences in mean resource use, costs, EQ-5D scores at each time point, and QALYs(25, 26). Probabilistic uncertainty using Monte Carlo Simulations (27) were used to resample each estimated (non-sampled) variable to account for uncertainty around point estimates; the difference in mean costs, QALYs and incremental NMBs with confidence intervals between groups. The incremental NMBs were calculated as the difference in mean QALYs per participant (*Q*) with 10TT(*T*) versus usual care(*U*) multiplied by the maximum willingness to pay for a QALY (*R*) minus the difference in mean cost per participant (*C*), i.e., incremental NMB = $(Q_T – Q_U)*R – (C_T– C_U)$. We used the cost-effectiveness threshold range recommended by NICE (£20 000 to £30 000(13) as the lower and upper limits of the maximum willingness to pay for a QALY (*R*). Negative incremental NMBs indicate that usual care is preferred on cost-effectiveness grounds and positive incremental NMBs indicate that 10TT is preferred(28).The ordinary least squares (OLS) method, which assumes a normal distribution and minimises residual error was applied to estimate the unknown difference between observed QALYs gained and the dependant variables; age, gender, practice and baseline utility values using regression analysis. Incremental costs were initially adjusted for age, gender, practice and prior costs in the previous two years (generalised linear model with gamma family and log link to account for skewness). Adjusting for these factors did not make significant difference to the results (Table 2) and were therefore not included in the final model.

For each of the 20 imputed datasets we ran 1000 bootstrap replications using non-parametric bootstrapping, resampling observations with replacement. The bootstrapped results were combined using the formula described by Briggs et al(21), to calculate standard

For peer review only - http://bmjopen.bmj.com/site/about/guidelines.xhtml

errors around mean values allowing for uncertainty around the imputed values, skewness of cost and utility data, and sampling variation. Standard errors were on based a normal distribution and used to calculate 95% CIs around point estimates.

The cost-effectiveness plane (figure 1) were used to illustrate the difference in costs and outcomes between the two arms on a graph. The probability that 10TT was cost-effective in comparison to usual care at different values of the cost-effectiveness threshold was presented in form of a cost-effectiveness acceptability curve (CEAC) (figure 2)(29). The CEAC was based on the proportion of bootstrap replications across all twenty imputed datasets that were below the cost-effectiveness threshold, which was varied from £0 to £50,000.

Several deterministic sensitivity analyses were carried out to assess the uncertainty around key components of the analysis. An analysis was undertaken based on complete cases without any imputation. Cost components varied were primary and secondary care costs and drug costs. The total costs of each component were amended by ±10%. We also conducted analyses including the cost of standard weight-loss interventions for usual care only.

Results

Baseline description

Table 3 illustrates the mean costs, two years prior to the trial. The mean cost for the usual care group was £1848 (95% CI: 1615, 2082), and £2052 (95% CI: 1756, 2349) in the 10TT group. Baseline characteristics- age, gender, weight, BMI blood pressure and cholesterol did not differ between groups(30).

Base case

For peer review only - http://bmjopen.bmj.com/site/about/guidelines.xhtml

For peer review only

Table 1 shows mean imputed resource use by treatment group. The intervention and control groups had the same mean GP practice visits per participant over the two-year period (9.4 versus 9.4), as well as similar GP home visits (0 versus 0.1), GP phone contacts (1.3 versus 1.3), nurse practice visits (6.6 versus 6.9), nurse home visits (0 versus 0.1), inpatient episodes (0.2 versus 0.2), outpatient clinic visits (2.0 versus 2.1) and accident and emergency visits (0.4 versus 0.4). There was no statistically significant difference between groups for any category of health service resource use.

Table 3 shows the total cost per group. The non-parametric bootstrapping including imputed resource use produced mean total costs per participant in the intervention group of £1889 (95% CI: £1522 to £2566), compared to £1925 (95% CI: £1599 to £2251) in the control group. The mean difference in cost between 10TT and control group was-£36 (95%CI: -£511 to £440), which was not statistically significant (p=0.88) (Table 3).

Undertaking non-parametric bootstrapping after multiple imputation produced 1.51 (95% CI: 1.44 to 1.57) QALYs in the intervention group and 1.51 (95% CI: 1.45 to 1.57) in the control group. Generating a mean difference in QALYs of 0.0007 (95%CI: -0.080 to 0.082) (p=0.85) (Table 4 and 5) which was not statistically significant. Hence, patients receiving 10TT accrued non-significantly lower costs and the difference in QALYs between the groups was very small.

The incremental net monetary benefit for 10TT versus usual care was £49 (95% CI: -£1709 to £1800) at a maximum willingness to pay for a QALY of £20,000 and £55 (95% CI: -2489, 2583) at a maximum willingness to pay for a QALY of £30,000 (Table 4).

For peer review only - http://bmjopen.bmj.com/site/about/guidelines.xhtml

Of the 20,000 bootstrap replications produced under base case assumptions, 24% fell into south-west quadrant of the cost-effectiveness plane (10TT was less costly and less effective than usual care); 20% fell into the north-east quadrant (10TT was more costly and more effective than usual care); 25% fell into the north-west quadrant (10TT was more costly and less effective than usual care); and 31% fell into the south-east quadrant (10TT was less costly and more effective than usual care) (Figure 1). The CEAC derived from Figure 1 is illustrated in Figure 2. Under base case assumptions, 10TT has a 52% probability of being cost-effective at a willingness to pay threshold of £20,000 and 52% at a willingness to pay threshold of £30,000 (Table 4).

Sensitivity analyses

Including the cost of the standard weight-loss interventions (£87) in the usual care group the mean costs per patient in the control group increased to £2012, which was higher than the mean cost in the intervention group (£1889). The mean incremental cost difference between groups was -£122 (95% CI: -£598 to £353). The incremental net monetary benefit for 10TT versus usual care was £140 (95% CI: (-£1666 to £1902) at a maximum willingness to pay for a QALY of £20,000 and £148 (95% CI: -£2463 to £2693) at a maximum willingness to pay of £30,000. Increasing and decreasing costs by 10% did not affect the findings appreciably (Table 4).

A complete case analysis was undertaken using 163 of 537 participants with complete utility and cost data. The incremental NMB was -£877 (95% CI: -£2338 to £3404) at a maximum willingness to pay for a QALY of £20,000 and -£1349 (95% CI: -£3269 to £4972) at a maximum willingness to pay for a QALY of £30,000.

Discussion

For peer review only - http://bmjopen.bmj.com/site/about/guidelines.xhtml

Our economic analysis of 10TT showed that this intervention had similar costs and QALYs as usual care. Sensitivity analyses showed little uncertainty in this finding. On the one hand the findings mean there is no reason to prefer 10TT or usual care on the basis of differences in quality of life or cost, or on cost-effectiveness grounds. On the other hand, this means that 10TT is as cost-effective as usual care.

Previous studies have reported the cost-effectiveness of primary care led programmes such as lifestyle counselling(8), commercial weight loss programme(6, 7), patient education and the combination of diet and exercise over 12 months to 24 month period, compared with usual care.

Fuller et al(7) evaluated the cost-effectiveness of standard care versus a commercial weight loss programme (Weight Watchers (CP)) in a population of 772 participants from Australia, UK and Germany, over a timeframe of one year. The cost per kilogram of weight loss was US$122 (Australia), US$90 (UK) and US$180 (Germany) for the commercial provider and US$138 (Australia), US$151 (UK) and US$133 (Germany) for standard care. The incremental cost per QALY gained of the commercial programme versus standard care was US$18,266 (Australia), US$12,100 (UK) and US$40,933 (Germany). A further collection of follow-up data at two-years was used to develop a decision analytic model to estimate the lifetime costs and outcomes in the Australian population. Over a lifetime of the patient, the commercial weight loss programme resulted in an incremental cost saving of US$47 per patient(6, 31), suggesting that the commercial programme may be cost effective. When analysing completed Quality of Life questionnaires (IWQOL-Lite) there was a significantly greater change in utility for commercial weight loss programme (Weight Watchers (CP)) for Australia and Germany but not in the UK. However, this may have been due to the small

For peer review only

sample size. When an intention to treat analysis was undertaken, CP was the preferred approach and the difference in utility scores between CP and standard care (SC) was reported statistically significant for Australia (0.0006) and the UK (0.033). A long-term analysis of the trial suggested over a lifetime of the patient resulted in a cost saving of US$47 per patient and an incremental 0.03 QALY gained per patient(6), however these differences were not statistically significant.

An evaluation of obesity treatment in Pennsylvania (USA), with 390 participants looked at the cost-effectiveness of usual care (quarterly visits to the primary care provider), lifestyle counselling (quarterly visits to the primary care provider plus monthly weight loss counselling) and enhanced lifestyle counselling (all the above plus meal replacements and weight loss medication). The study showed the incremental cost per kilogram lost was US$292 for the enhanced lifestyle counselling compared to usual care. Long-term estimates using published data and trial data suggested Brief Lifestyle Counselling would fall somewhere between US$7000 and US$12600 cost per QALY; Enhanced Brief lifestyle counselling the cost per kilogram could be as high as US$22000(8).

The cost-effectiveness of the Counterweight intervention in the UK (a nurse-delivered patient education programme) showed that the intervention was dominant (less costly and more effective) compared with no active intervention; it produced a gain in QALYs (0.06 per participant) and cost savings of £27 per participant (9).

For overweight and obese women, a three component intervention of diet, exercise and behaviour change in the USA produced a cost per QALY gained of US$12,600 compared with usual care, exercise, and diet alone(5). The authors concluded the three-component programme was good value for money.

For peer review only - http://bmjopen.bmj.com/site/about/guidelines.xhtml

A recent evaluation of a primary care led behavioural programme(32) looking at a brief advice and self-help materials, weight management programme over 12 weeks and the same weight management programme over 52 weeks concluded that a weight loss programme over 12 weeks is more effective (-4.75kg) than brief advice and self-help material (-3.26kg) and a 52 weeks programme produces greater weight loss (-6.76kg) and was £159 per kg lost, producing additional clinical benefits. The ICER for the 52- week programme when compared to the brief intervention was £2394 per QALY.

The difference in the results of these studies may be explained by different settings in which the intervention is executed, the type of interventions being evaluated and finally, the practice of usual care, which may differ across countries and healthcare practitioners. Given the evidence on primary care led programmes, there is a possibility that 10TT alongside other programmes would be effective at a low-cost.

The main strength of our analysis is that it is based on a large multicentre randomised trial with detailed information on resource use and utility values for a median follow-up period of two years. There were several limitations to our study. While data were recorded in the trial on standard weight-loss interventions for obese patients at each practice, individual data on the uptake and prescribing of standard weight-loss interventions received by the participant were not recorded. This trial was also unblinded, which introduced the potential bias of GPs over prescribing standard weight-loss interventions producing a healthy user bias effect. Under base case assumptions, excluding standard weight-loss interventions, the incremental NMB of 10TT versus usual care was not significantly different from zero, and this did not change when intervention costs in the control group were included. Hence, these costs did not make an appreciable difference to our conclusions. Secondly, the

For peer review only - http://bmjopen.bmj.com/site/about/guidelines.xhtml

analysis took a UK NHS/PSS perspective. A wider perspective (e.g., societal) could have been taken, including costs to patients and families. Third, the time horizon was limited to two years, due to the lack of difference in costs (-£36 (95%CI -£511 to £440, p-value 0.88) and benefits (0.0007 (95% CI -0.080 to 0.082, p-value 0.93) at 24 months between groups. The trial did not capture major effects of weight loss post 24 months. A longer follow-up period may have allowed for the detection of long-term health benefits produced as a consequence of avoiding obesity related health conditions, such as diabetes and cardiovascular disease. Fourth, while there are various instruments are available to measure Health related Quality of Life (HRQoL) associated with weight loss. We administered the EQ-5D(33). We acknowledge that there may have been a potential value in using more than one measure in the trial, such as the SF-36 and Impact of Weight on Quality of Life –Lite (IWQOL-Lite) to measure differences in the positive short-term psychological effects(34). However, given the small difference between the two arms it is highly unlikely alternative measures would've produced significant differences. Finally, we were unable to access Hospital Episode Statistics (HES) data containing secondary care resource use of NHS services by patients. Obtaining HES data would been problematic due to the short timeframe of this trial and linking primary care data to HES data by patient ID(35). As a consequence, data for secondary care resource use were extracted from GP practices with a degree of missingness in our analysis. We accounted for this using multiple imputation, assuming data to be missing at random.

In conclusion, the 10TT trial found that after three months' participants receiving 10TT lost significantly more weight than those receiving usual care, but there was no difference in weight change between the two groups at 24 months. Consistent with these findings, the

For peer review only - http://bmjopen.bmj.com/site/about/guidelines.xhtml

For peer review only

present study found that differences in costs and QALYs between 10TT and usual care were not significantly different over the two-year period. There is no evidence to either recommend or advise against 10TT, based on cost-effectiveness considerations.

Acknowledgements

Weight Concern and Cancer Research UK developed the 10 Top Tips intervention and materials, and Weight Concern provided training on how to deliver the intervention. The General Practice Research Framework provided advice on the study design and was involved in both recruiting sites and training the health professionals at each site on the trial procedures, along with providing quality control visits. Practices were located in: Wellingborough, Southampton, Bradford-on-Avon, Bromsgrove, Frome, Guisborough, Glastonbury, Ivybridge, Dunstable, Liskeard, Ledbury, New Mills, and London. The authors would like to acknowledge the substantial intellectual contribution made by Professor Jane Wardle who sadly passed away prior to publication, and is deeply missed by all of her co-authors, colleagues and students.

For peer review only

Contributors

NP undertook data analysis, interpreted the results and drafted and wrote the manuscript. RJB helped design the study, undertook data collection and critically reviewed the manuscript. BL helped design the study, critically reviewed the data and manuscript. RZO helped design the study and critically reviewed the manuscript. IN helped design the study and critically reviewed the manuscript. SM undertook the data analysis, devised the analysis plan, interpreted the results and critically reviewed the manuscript.

Funding

For peer review only - http://bmjopen.bmj.com/site/about/guidelines.xhtml

This work was funded by the Medical Research Council (MRC) – National Prevention Research Initiative (NPRI) (UK) grant number G080202. RJB's post was funded through this grant and she is now supported by Yorkshire Cancer Research Academic Fellowship funding. SM, NP and IN are HEFCE-funded. RZO was funded by the National Institute for Health Research.

Disclaimer

The views expressed are those of the authors and not necessarily those of the funding bodies.

Competing interests

All authors have completed the ICMJE uniform disclosure form at http://www.icmje.org/coi_disclosure.pdf (available on request from the corresponding author) and declare that: NP, RJB, BL, RZO, IN and SM have nothing to disclose.

Ethics approval

This study has been approved by the South East London REC 2, 23/08/2010 (Reference number 10/H0802/59). All participants gave informed consent before taking part.

Provenance and peer review

Not commissioned; externally peer reviewed.

Data sharing statement

No additional data are available.

For peer review only

For peer review only - http://bmjopen.bmj.com/site/about/guidelines.xhtml

Table 1. Resource use, unit cost, and mean cost per participant for primary and secondary care services post randomisation

		Usual Care					10TT					Usual Care	10TT	Incremental difference	
	N	Mean	(SD)	Median	(IQR)	N	Mean	(SD)	Median	(IQR)	Unit cost (£)	Mean cost (£)	Mean cost (£)	Mean (£)	(95% CI)
**No imputation for missing values**															
GP surgery visit	204	9.4	(8.4)	7	(7.00)	182	9.7	(9.5)	7	(9.00)	45	417	425	-8	(-89, 72)
GP home visit	196	0.1	(0.8)	0	(0.00)	176	0.0	(0.1)	0	(0.00)	292	37	9	-28	(-7, 61)
GP phone	199	1.3	(3.0)	0	(1.00)	179	1.3	(3.0)	0	(1.00)	27	34	34	0	(-16, 16)
GP practice nurse	206	6.9	(8.4)	4	(6.00)	184	6.8	(9.5)	4	(6.50)	40	274	268	-6	(-64, 77)
Nurse home visit	195	0.1	(1.1)	0	(0.00)	175	0.0	(0.1)	0	(0.00)	70	5	1	-4	(-7, 16)
GP practice nurse phone	197	0.5	(2.3)	0	(0.00)	176	0.4	(1.2)	0	(0.00)	10	5	4	-1	(-2, 5)
Dietician	195	0.1	(0.5)	0	(0.00)	175	0.1	(0.8)	0	(0.00)	35	4	4	0	(-4, 4)
Additional nurse visit	197	1.1	(3.1)	0	(1.00)	179	1.6	(2.6)	0	(1.00)	4	45	44	-1	(-22, 23)
Inpatient admission	196	0.2	(0.7)	0	(0.00)	175	0.2	(0.7)	0	(0.00)	1713	372	387	-15	(-249, 220)
Outpatient clinic	200	2.2	(3.5)	1	(3.00)	178	2.1	(3.3)	1	(3.00)	135	288	269	-19	(-70, 110)
Accident & Emergency visit	199	0.4	(1.2)	0	(0.00)	175	0.4	(0.9)	0	(1.00)	177	65	67	-2	(-39, 36)
Other outpatient services	194	1.0	(1.8)	0	(1.00)	179	1.0	(2.3)	0	(1.00)	135	124	129	-5	(-58, 48)
**Missing values imputed***			SE					SE							
GP surgery visit	270	9.4	(0.6)	9		267	9.4	(0.7)	9		45	419	420	-1	(-79,81)
GP home visit	270	0.1	(0.5)	0		267	0.0	(0.2)	0		292	36	13	-23	(-58, 12)
GP phone	270	1.3	(0.2)	1		267	1.3	(0.2)	1		27	35	33	-2	(-19, 14)
GP practice nurse	270	6.9	(0.6)	6		267	6.6	(0.7)	6		40	274	266	-8	(-75, 57)
Nurse home visit	270	0.1	(1.0)	0		267	0.0	(0.1)	0		70	6	2	-4	(-16, 7)
GP practice nurse phone	270	0.5	(0.6)	0		267	0.4	(0.1)	0		10	5	4	-1	(-5, 2)
Dietician	270	0.1	(0.0)	0		267	0.1	(0.6)	0		35	4	4	0	(-4, 4)

For peer review only

For peer review only - http://bmjopen.bmj.com/site/about/guidelines.xhtml

Additional nurse visit	270	1.2 (0.2)	1	267	1.8 (0.2)	0	4	46	47	-1	(-23, 23)
Inpatient admission	270	0.2 (0.1)	0	267	0.2 (0.1)	0	1713	373	379	-6	(-238, 251)
Outpatient clinic	270	2.1 (0.3)	2	267	2.0 (0.2)	0	135	287	266	-21	(-106, 63)
Accident & Emergency visit	270	0.4 (0.9)	0	267	0.4 (0.7)	0	177	67	69	-2	(-38, 42)
Other outpatient services	270	1 (0.1)	1	267	1 (0.2)	0	135	122	129	-7	(-52, 65)
**Weight loss interventions**											
10TT		- -	- -		- -	- -	23	-	-		
Weight Watchers		- -	- -		- -	- -	78	-	-		
Slimming World		- -	- -		- -	- -	93	-	-		
Size Down Programme		- -	- -		- -	- -	73	-	-		

GP = general practitioner. SD = standard deviation for non-imputed data. SE = standard error for imputed data to capture how confident we are about the mean value. CI = confidence interval. IQR = inter-quartile range. Costs are in 2013/14 UK£. *Missing values imputed using multiple imputation with 20 imputed datasets (see text).

Table 2. Difference between groups in age, sex, costs and health-related quality of life at baseline

	Usual care				10TT				
	N	Mean	SD	95% CI	N	Mean	SD	95% CI	P-value≠
Age	270	58	12.61	(56.77, 59.79)	267	57	12.88	(55.93, 59.79)	0.5
Sex*									
Male	95	35.2	-	-	89	33.3	-	-	-
Female	175	64.8	-	-	178	66.7	-	--	-
Prior costs	270	1848	1948	(1615, 2082)	267	2052	2461	(1756, 2349)	0.3
Baseline EQ-5D	257	0.76	0.24	(0.73, 0.79)	255	0.74	0.27	(0.71, 0.76)	0.41

SD = standard deviation. CI = confidence interval. ≠From independent t-test, comparing mean cost between groups. *Based on mean proportion (%) of in each group

For peer review only - http://bmjopen.bmj.com/site/about/guidelines.xhtml

Table 3. Cost description two years prior and post randomisation

	Usual care			10TT			Incremental difference	
	**N**	**Mean**	**SD**	**N**	**Mean**	**SD**	**Mean (£)**	**(95% CI)**
**Cost, two years prior**								
Cost of primary and secondary care contacts	270	1516	1694	267	1735	2186	-219	(-550, 112)
Drugs costs	270	332	748	267	317	624	-15	(-102, 132)
Total cost	270	1848	1948	267	2052	2461	204	(-580, 172)
**Costs after randomisation (imputed)***			**SE**			**SE**		
Cost of primary and secondary care contacts	270	1675	2204	267	1631	2084	-44	(-477, 387)
Drugs costs	270	249	715	267	236	1114	-13	(-172, 146)
Intervention costs	-	-	-	267	23	-	-	-
Total cost	270	1925	2477	267	1889	2427	-36	(-511, 440)

SD = standard deviation for non-imputed data. SE = standard error for imputed data to capture how confident we are about the mean value. CI = confidence interval

For peer review only - http://bmjopen.bmj.com/site/about/guidelines.xhtml

For peer review only

Table 4. Incremental cost-effectiveness of 10TT versus usual care

	Incremental cost		QALYs gained		Incremental net monetary benefit (NMB)				Probability cost-effective	
					£20,000		£30,000			
	Mean (£)	(95% CI)	Mean	(95% CI)	Mean (£)	(95% CI)	Mean (£)	(95% CI)	£20,000	£30,000
Base case*	-36	(-511, 440)	0.0007	(-0.080, 0.816)	49	(-1709, 1800)	55	(-2489, 2583)	52%	52%
Base case with control group intervention cost~	-122	(-598, 353)	0.0007	(-0.080, 0.082)	140	(-1666, 1902)	148	(-2463, 2693)	56%	54%
Complete case analysis§	-66	(-907, 774)	-0.0472	(-0.180, 0.086)	-877	(-2338, 3404)	-1349	(-3269, 4972)	66%	67%
Primary and secondary care cost increased #	-40	(-556, 475)	0.0007	(-0.080, 0.081)	48	(-1743, 1851)	53	(-2520, 2623)	52%	51%
Drug cost increased #	23	(-396, 442)	0.0007	(-0.080, 0.077)	-14	(-1734, 1716	-9	(-2511, 2510)	49%	50%
All costs increased #	-39	(-554, 476)	0.0007	(-0.080, 0.082)	43	(-1756, 1851)	46	(-2537, 2641)	52%	51%
Primary and secondary care cost decreased ¥	-31	(-463, 401)	0.0007	(-0.080, 0.081)	54	(-1683, 1804)	64	(-2453, 2587)	52%	52%
Drug costs decreased ¥	26	(-387, 438)	0.0007	(-0.080, 0.081)	0	(-1741, 1727)	11	(-2530, 2520)	50%	51%
All costs decreased ¥	-32	(-460, 395)	0.0007	(-0.080, 0.081)	50	(-1682, 1811)	59	(-2458, 2599)	52%	52%

*N= 537. Data include values imputed using multiple imputation (see text). The 95% confidence intervals were derived from 1000 bootstrap replications of each of the 20 imputed datasets (see text). The incremental net monetary benefit and the probability 10TT is cost-effective are based on the QALYs gained and incremental costs and calculated at a maximum willingness to pay for a QALY of £20,000 and £30,000.
~ As for the base case analysis except standard weight-loss interventions (Weight Watchers, Slimming World, Size Down programme) are included in the control group.
§As for the base case analysis except there is no multiple imputation of missing values and the 95% confidence intervals were derived from 1000 bootstrap replications of a single dataset containing the n=72 participants in the 10TT group and n=91 participants in the usual care group with no missing values. The costs of interventions in the control group (Weight Watchers, Slimming World, Size Down programme) were excluded.
# As for the base case analysis except costs are increased by 10% for 10TT.
¥As for the base case analysis except costs are decreased by 10% for 10TT.

For peer review only - http://bmjopen.bmj.com/site/about/guidelines.xhtml

For peer review only

Table 5. Utility values and QALYs per patient

	Usual Care				10TT				Incremental difference	
	N	Mean	SD	95% CI	N	Mean	SD	95% CI	Mean	(95% CI)
**No imputation for missing values**										
3 months	206	0.77	0.23	(0.74, 0.80)	190	0.77	0.26	(0.73, 0.80)	0	(-0.5, 0.05)
6 months	163	0.76	0.24	(0.72, 0.80)	159	0.78	0.24	(0.74, 0.82)	-0.02	(-0.07, 0.03)
12 months	151	0.77	0.24	(0.73, 0.81)	138	0.74	0.27	(0.70, 0.79)	0.03	(-0.03, 0.09)
18 months	138	0.75	0.25	(0.71, 0.79)	124	0.74	0.27	(0.69, 0.79)	0.01	(-0.05, 0.07)
24 months	157	0.76	0.25	(0.73, 0.80)	131	0.76	0.26	(0.72, 0.81)	0	(-0.06, 0.06)
Discounted QALYs	92	1.52	0.40	(1.44 1.61)	73	1.47	0.47	(1.36, 1.58)	0.05	(-0.08, 0.18)
**Missing values imputed***			**SE**				**SE**			
3 months	270	0.77	0.016	(0.73, 0.80)	267	0.77	0.017	(0.71, 0.78)	-0.01	(-0.04, 0.05)
6 months	270	0.76	0.016	(0.73, 0.79)	267	0.78	0.018	(0.73, 0.81)	-0.04	(-0.03, 0.06)
12 months	270	0.76	0.019	(0.73, 0.79)	267	0.75	0.020	(0.71, 0.80)	-0.01	(-0.06, 0.04)
18 months	270	0.76	0.019	(0.72, 0.79)	267	0.75	0.020	(0.71, 0.79)	-0.01	(-0.07, 0.05)
24 months	270	0.76	0.019	(0.72, 0.79)	267	0.76	0.018	(0.72, 0.79)	0	(-0.04, 0.05)
Discounted QALYs	270	1.51	0.030	(1.45, 1.57)	267	1.51	0.033	(1.44, 1.58)	0.0007	(-0.080, 0.082)

QALY = quality-adjusted life year. SD = standard deviation for non-imputed data. SE = standard error for imputed data to capture how confident we are about the mean value. CI = confidence interval. Costs are in 2013/14 UK£. CI were based on non-parametric bootstrapping for observed data and imputed data.
* Missing values imputed using multiple imputation with 20 imputed datasets (see text).

For peer review only - http://bmjopen.bmj.com/site/about/guidelines.xhtml

References

1. http://www.euro.who.int/en/health-topics/noncommunicable-diseases/obesity/data-and-statistics. Accessed 07.03.15.
2. http://ec.europa.eu/eurostat/statistics-explained/index.php/Overweight_and_obesity_-_BMI_statistics. Accessed 07.03.15.
3. Guh DP, Zhang W, Bansback N, Amarsi Z, Birmingham CL, Anis AH. The incidence of co-morbidities related to obesity and overweight: a systematic review and meta-analysis. BMC public health. 2009;9:88.
4. Reducing obesity and improving diet. https://www.gov.uk/government/policies/reducing-obesity-and-improving-diet. Accessed 07.03.15.
5. Roux L, Kuntz KM, Donaldson C, Goldie SJ. Economic evaluation of weight loss interventions in overweight and obese women. Obesity (Silver Spring, Md). 2006;14(6):1093-106.
6. Fuller NR, Carter H, Schofield D, Hauner H, Jebb SA, Colagiuri S, et al. Cost effectiveness of primary care referral to a commercial provider for weight loss treatment, relative to standard care: a modelled lifetime analysis. Int J Obes. 2014;38(8):1104-9.
7. Fuller NR, Colagiuri S, Schofield D, Olson AD, Shrestha R, Holzapfel C, et al. A within-trial cost-effectiveness analysis of primary care referral to a commercial provider for weight loss treatment, relative to standard care--an international randomised controlled trial. International journal of obesity (2005). 2013;37.
8. Tsai AG, Wadden TA, Volger S, Sarwer DB, Vetter M, Kumanyika S, et al. Cost-effectiveness of a primary care intervention to treat obesity. International journal of obesity (2005). 2013;37 Suppl 1:S31-7.
9. Trueman P, Haynes SM, Felicity Lyons G, Louise McCombie E, McQuigg MS, Mongia S, et al. Long-term cost-effectiveness of weight management in primary care. International journal of clinical practice. 2010;64(6):775-83.
10. Gardner B, Lally P, Wardle J. Making health habitual: the psychology of 'habit-formation' and general practice. The British journal of general practice : the journal of the Royal College of General Practitioners. 2012;62(605):664-6.
11. Lally P, Chipperfield A, Wardle J. Healthy habits: efficacy of simple advice on weight control based on a habit-formation model. Int J Obes. 2007;32(4):700-7.
12. http://www.nhs.uk/Livewell/5ADAY/Pages/Why5ADAY.aspx. Accessed 18.07.17.
13. National Institute for Health and Care Excellence (NICE). Guide to the methods of technology appraisal. London: NICE; 2013.
14. Curtis L. Unit costs of health and social care 2013. Personal Social Services Research Unit Kent; 2013.
15. Department of Health. National Schedule of Reference Costs- 2012-2013. NHS trusts and NHS foundation trusts. London Department of Health; 2012.
16. Joint Formulary Committee . British National Formulary, 68th edition. London: BMJ Group and Pharmaceutical Press; 2013 [Available from: https://www.medicinescomplete.com/mc/bnf/current/. Accessed December 2014.
17. Jolly K, Lewis A, Beach J, Denley J, Adab P, Deeks JJ, et al. Comparison of range of commercial or primary care led weight reduction programmes with minimal intervention control for weight loss in obesity: lighten Up randomised controlled trial. BMJ (Clinical research ed). 2011;343:d6500.
18. Rabin R, de Charro F. EQ-5D: a measure of health status from the EuroQol Group. Annals of medicine. 2001;33(5):337-43.
19. Brooks R. EuroQol: the current state of play. Health policy (Amsterdam, Netherlands). 1996;37(1):53-72.

For peer review only - http://bmjopen.bmj.com/site/about/guidelines.xhtml

20. Petrou S, Dakin H, Abangma G, Benge S, Williamson I. Cost-utility analysis of topical intranasal steroids for otitis media with effusion based on evidence from the GNOME trial. Value in health : the journal of the International Society for Pharmacoeconomics and Outcomes Research. 2010;13(5):543-51.
21. Briggs A, Clark T, Wolstenholme J, Clarke P. Missing... presumed at random: cost-analysis of incomplete data. Health economics. 2003;12(5):377-92.
22. Royston P Multiple imputation of missing values: update of ice. Stata J. 2005: 527-36.
23. Morris S, Patel NV, Dobson J, Featherstone RL, Richards T, Luengo-Fernandez R, et al. Cost-utility analysis of stenting versus endarterectomy in the International Carotid Stenting Study. International journal of stroke : official journal of the International Stroke Society. 2016;11(4):446-53.
24. StataCorp. 2015. Stata Statistical Software: Release 14. College Station, TX: StataCorp LP. .
25. Sinharay S, Stern HS, Russell D. The use of multiple imputation for the analysis of missing data. Psychological methods. 2001;6(4):317-29.
26. Yang X, Belin TR, Boscardin WJ. Imputation and variable selection in linear regression models with missing covariates. Biometrics. 2005;61(2):498-506.
27. Pasta DJ, Taylor JL, Henning JM. Probabilistic sensitivity analysis incorporating the bootstrap: an example comparing treatments for the eradication of Helicobacter pylori. Medical decision making : an international journal of the Society for Medical Decision Making. 1999;19(3):353-63.
28. Zethraeus N, Johannesson M, Jonsson B, Lothgren M, Tambour M. Advantages of using the net-benefit approach for analysing uncertainty in economic evaluation studies. PharmacoEconomics. 2003;21(1):39-48.
29. Briggs AH, Gray AM. Handling uncertainty when performing economic evaluation of healthcare interventions. Health technology assessment (Winchester, England). 1999;3(2):1-134.
30. Beeken RJ, Leurent B, Vickerstaff V, Wilson R, Croker H, Morris S, et al. A brief intervention for weight control based on habit-formation theory delivered through primary care: results from a randomised controlled trial. International journal of obesity (2005). 2017.
31. http://data.worldbank.org/indicator/pa.nus.pppc.rf. Accessed 07.03.15.
32. Ahern AL, Wheeler GM, Aveyard P, Boyland EJ, Halford JCG, Mander AP, et al. Extended and standard duration weight-loss programme referrals for adults in primary care (WRAP): a randomised controlled trial. Lancet. 2017;389(10085):2214-25.
33. Kolotkin RL, Norquist JM, Crosby RD, Suryawanshi S, Teixeira PJ, Heymsfield SB, et al. One-year health-related quality of life outcomes in weight loss trial participants: comparison of three measures. Health and quality of life outcomes. 2009;7:53.
34. Peckmezian T, Hay P. A systematic review and narrative synthesis of interventions for uncomplicated obesity: weight loss, well-being and impact on eating disorders. Journal of eating disorders. 2017;5:15.
35. Herrett E, Shah AD, Boggon R, Denaxas S, Smeeth L, van Staa T, et al. Completeness and diagnostic validity of recording acute myocardial infarction events in primary care, hospital care, disease registry, and national mortality records: cohort study. BMJ (Clinical research ed). 2013;346:f2350.

For peer review only - http://bmjopen.bmj.com/site/about/guidelines.xhtml

Figure 1. Cost-Effectiveness plane, base case analysis assumptions. Based on 20,000 bootstrap replications, from 20 imputed datasets.

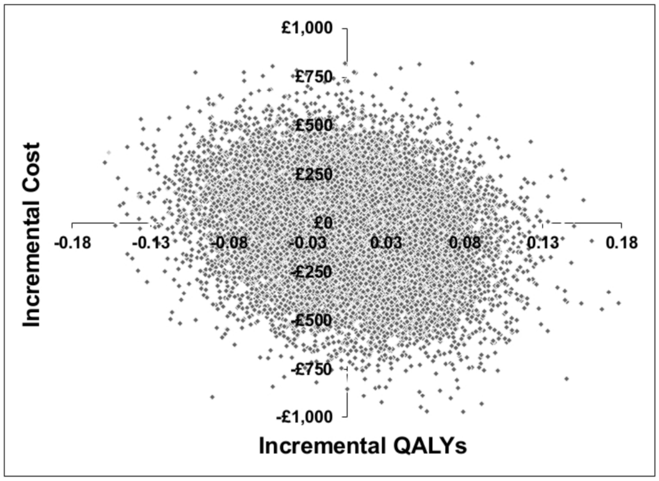

1

Figure 1. Cost-Effectiveness plane, base case analysis assumptions. Based on 20,000 bootstrap replications, from 20 imputed datasets.

296x209mm (300 x 300 DPI)

Figure 2. Cost-effectiveness acceptability curve showing the probability that 10TT is cost-effective vs usual care at different values of the maximum willingness to pay for a QALY (base-case assumptions)

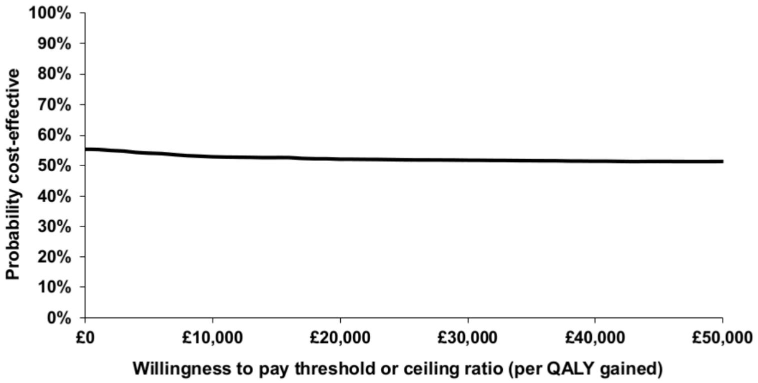

QALY = quality adjusted life year

Figure 2. Cost-effectiveness acceptability curve showing the probability that 10TT is cost-effective vs usual care at different values of the maximum willingness to pay for a QALY (base-case assumptions)

296x209mm (300 x 300 DPI)

ew only

For peer review only - http://bmjopen.bmj.com/site/about/guidelines.xhtml

BMJ

BMJ 2013;346:f1049 doi: 10.1136/bmj.f1049 (Published 25 March 2013) Page 1 of 6

RESEARCH METHODS & REPORTING

Consolidated Health Economic Evaluation Reporting Standards (CHEERS) statement

OPEN ACCESS

Don Husereau *senior associate; adjunct professor of medicine; senior scientist*[1 2 3], Michael Drummond *co-editor-in-chief, Value in Health; professor of health economics*[4], Stavros Petrou *professor of health economics*[5], Chris Carswell *editor*[6], David Moher *senior scientist*[7], Dan Greenberg *associate professor and chairman; visiting assistant professor*[8 9], Federico Augustovski *director; professor of public health*[10 11], Andrew H Briggs *William R Lindsay chair of health economics, health economics and health technology assessment*[12], Josephine Mauskopf *vice president of health economics*[13], Elizabeth Loder *chief of division; clinical epidemiology editor, BMJ*[14], on behalf of the CHEERS Task Force

[1]Institute of Health Economics, Edmonton, Canada; [2]Department of Epidemiology and Community Medicine. University of Ottawa, Ottawa, Canada; [3]University for Health Sciences, Medical Informatics and Technology, Hall in Tirol, Austria; [4]Centre for Health Economics, University of York, York, UK; [5]Warwick Medical School, University of Warwick, Coventry, UK; [6]Pharmacoeconomics, Adis International, Auckland, New Zealand; [7]Clinical Epidemiology Program, Ottawa Hospital Research Institute, Ottawa, Canada; [8]Department of Health Systems Management, Faculty of Health Sciences, Ben-Gurion University of the Negev, Beer-Sheva, Israel; [9]Center for the Evaluation of Value and Risk in Health, Tufts Medical Center, Boston MA, USA; [10]Health Economic Evaluation and Technology Assessment, Institute for Clinical Effectiveness and Health Policy (IECS), Buenos Aires, Argentina; [11]Universidad de Buenos Aires, Buenos Aires, Argentina; [12]Institute of Health and Wellbeing, University of Glasgow, Glasgow, Scotland; [13]RTI Health Solutions, Research Triangle Park NC, USA; [14]Division of Headache and Pain, Brigham and Women's/Faulkner Neurology, Faulkner Hospital, Boston MA, USA

Abstract

Economic evaluations of health interventions pose a particular challenge for reporting. There is also a need to consolidate and update existing guidelines and promote their use in a user friendly manner. The Consolidated Health Economic Evaluation Reporting Standards (CHEERS) statement is an attempt to consolidate and update previous health economic evaluation guidelines efforts into one current, useful reporting guidance. The primary audiences for the CHEERS statement are researchers reporting economic evaluations and the editors and peer reviewers assessing them for publication.

The need for new reporting guidance was identified by a survey of medical editors. A list of possible items based on a systematic review was created. A two round, modified Delphi panel consisting of representatives from academia, clinical practice, industry, government, and the editorial community was conducted. Out of 44 candidate items, 24 items and accompanying recommendations were developed. The recommendations are contained in a user friendly, 24 item checklist. A copy of the statement, accompanying checklist, and this report can be found on the ISPOR Health Economic Evaluations Publication Guidelines Task Force website (www.ispor.org/TaskForces/EconomicPubGuidelines.asp).

We hope CHEERS will lead to better reporting, and ultimately, better health decisions. To facilitate dissemination and uptake, the CHEERS statement is being co-published across 10 health economics and medical journals. We encourage other journals and groups, to endorse CHEERS. The author team plans to review the checklist for an update in five years.

Health economic evaluations are conducted to inform resource allocation decisions. Economic evaluation has been defined as "the comparative analysis of alternative courses of action in terms of both their costs and their consequences."[1] All economic evaluations assess costs, but approaches to measuring and valuing the consequences of health interventions may differ (see box).

Economic evaluations have been widely applied in health policy, including the assessment of prevention programmes (such as vaccination, screening, and health promotion), diagnostics, treatment interventions (such as drugs and surgical procedures), organisation of care, and rehabilitation. Economic evaluations are increasingly being used for decision making and are an important component of programmes for health technology assessment internationally.[2]

Correspondence to: D Husereau, 879 Winnington Ave, Ottawa, ON K2B 5C4, Canada donh@donhusereau.com

No commercial reuse: See rights and reprints [illegible] Subscribe: http://www.bmj.com/subscribe

For peer review only - http://bmjopen.bmj.com/site/about/guidelines.xhtml

BMJ 2013;346:f1049 doi: 10.1136/bmj.f1049 (Published 25 March 2013)

Forms of economic evaluation[1]

Specific forms of analysis reflect different approaches to evaluating the consequences of health interventions. Health consequences may be estimated from a single analytical (experimental or non-experimental) study, a synthesis of studies, mathematical modelling, or a combination of modelling and study information.

Cost consequences analysis examines costs and consequences without attempting to isolate a single consequence or aggregate consequences into a single measure

Cost minimisation analysis (CMA)—The consequences of compared interventions are required to be equivalent, and only relative costs are compared

Cost effectiveness analysis (CEA) measures consequences in natural units, such as life years gained, disability days avoided, or cases detected. In a variant of CEA, often called cost utility analysis, consequences are measured in terms of preference-based measures of health, such as quality adjusted life years or disability adjusted life years.

Cost benefit analysis—Consequences are valued in monetary units.

Readers should be aware that an economic evaluation might be referred to as a "cost effectiveness analysis" or "cost benefit analysis" even if it does not strictly adhere to the definitions above. Multiple forms may also exist within a single evaluation. Different forms of analysis provide unique advantages or disadvantages for decision making. The Consolidated Health Economic Evaluation Reporting Standards (CHEERS) statement can be used with any form of economic evaluation.

Reporting challenges and shortcomings in health economic evaluations

Compared with clinical studies, which report the consequences of an intervention only, economic evaluations require more reporting space for additional items, such as resource use, costs, preference related information, and cost effectiveness results. This creates challenges for editors, reviewers, and those who wish to scrutinise a study's findings.[3] There is evidence that the quality of reporting of economic evaluations varies widely and could potentially benefit from improved quality assurance mechanisms.[4 5]

With the increasing number of publications available, and opportunity costs from decisions based on misleading study findings, transparency and clarity in reporting are important. In addition, outside of economic evaluations conducted alongside clinical trials, there are no widespread mechanisms for warehousing economic evaluation data to allow for independent interrogation, such as ethics review proceedings, regulator dossiers, or study registries. Instead, independent analysis may rely on the record keeping of individual investigators.

Even if measures to promote transparency exist, such as registries, biomedical journal editors have increasingly promoted and endorsed the use of reporting guidelines. Endorsement of guidelines by journals for randomised controlled trials has been shown to improve reporting.[6] The combination of the risk of making costly decisions due to poor reporting with the lack of mechanisms that promote accountability makes transparency in reporting economic evaluations especially important and a primary concern among journal editors and decision makers.[3 7]

Aim and scope

The aim of the Consolidated Health Economic Evaluation Reporting Standards (CHEERS) statement is to provide recommendations, in the form of a checklist, to optimise reporting of health economic evaluations. The need for a contemporary reporting guidance for economic evaluations was recently identified by researchers and biomedical journal editors.[8] The CHEERS statement attempts to consolidate and update previous efforts [9-20] into a single useful reporting guidance.

The primary audiences for the CHEERS statement are researchers reporting economic evaluations and the editors and peer reviewers evaluating their publication potential. We hope the statement (which consists of a 24 item checklist and accompanying recommendations on the minimum amount of information to be included when reporting economic evaluations) is a useful and practical tool for these audiences and will improve reporting and, in turn, health and healthcare decisions. To best understand and apply the recommendations contained within the statement, we encourage readers to access the Explanation and Elaboration Report.[21]

Development of the CHEERS statement

The statement was developed by a task force supported by the International Society for Pharmacoeconomics and Outcomes Research (ISPOR), as part of a broader initiative to facilitate and encourage the interchange of expert knowledge and develop best practices. The CHEERS Task Force members were chosen by the chair of the task force primarily based on their longstanding academic expertise and contribution to the multidisciplinary field of health economic evaluation. In addition to four members of the task force with doctorates in economics and its sub-discipline of health economics (AHB, MD, JM, SP), members included experts in health technology assessment and decision making (FA, AHB, DH, MD, JM) and in clinical epidemiology and biostatistics (AHB, EL, DM), those in active clinical practice (EL, FA), and those with previous experience in reporting guideline development (MD, DM). All members are researchers in applied health and health policy, with five members currently serving as editors for journals in the field (AHB, CC, MD, DG, EL).

The CHEERS Task Force followed current recommendations for developing reporting guidelines.[22] Briefly, the need for new guidance was first identified through a survey of members of the World Association of Medical Editors. Of the 6% (55/965) who responded, 91% (n=50) indicated they would use a standard if one were widely available.[8] Next, published checklists or guidance documents related to reporting economic evaluations were identified from a systematic review and survey of task force members.[23] Both of these activities were used to create a preliminary list of items to include when reporting economic evaluations. Recommendations of the minimum set of reporting items were then developed through a modified Delphi panel process. Forty eight individuals identified by the task force with broad geographical representation and representing academia, biomedical journal editors, the pharmaceutical industry, government decision makers, and those in clinical practice were invited to participate. Thirty eight agreed to participate. Participants were asked to score importance on a Likert scale and the average scores, weighted by each individual's confidence in ability to score, were then used to rank items. A cut-off point was applied to the ranked list to determine the minimum number of items important for reporting.

The CHEERS statement recommendations have been independently reviewed and subsequently revised by task force members. The recommendations are entirely those of the task

For peer review only - http://bmjopen.bmj.com/site/about/guidelines.xhtml

No commercial reuse: See rights and reprints http://www.bmj.com/permissions Subscribe: http://www.bmj.com/subscribe

BMJ 2013;346:f1049 doi: 10.1136/bmj.f1049 (Published 25 March 2013)

force—the sponsors of the study had no role in study design, data analysis, data interpretation, or writing of the final recommendations. A more complete description of the methods and findings of the Delphi panel are found in the larger explanation and elaboration document.[21]

Checklist items

The final recommendations are subdivided into six main categories: (1) title and abstract; (2) introduction; (3) methods; (4) results; (5) discussion; and (6) other. The recommendations are contained in a user friendly, 24 item checklist (table⇓) to aid users who wish to follow them. A copy of the checklist can also be found on the CHEERS Task Force website. (www.ispor.org/TaskForces/EconomicPubGuidelines.asp). In order to encourage dissemination and use of a single international standard for reporting, the task force approached 14 journals identified as either the largest publishers of economic evaluations or widely read by the medical and research community. Thirteen journals responded, and 10 expressed their ability and interest in endorsing this guidance. The CHEERS statement is being simultaneously published in *BMC Medicine, BMJ, BJOG: An International Journal of Obstetrics and Gynaecology, Clinical Therapeutics, Cost Effectiveness and Resource Allocation, The European Journal of Health Economics, International Journal of Technology Assessment in Health Care, Journal of Medical Economics, Pharmacoeconomics*, and *Value in Health*. To facilitate wider dissemination and uptake of this reporting guidance, we encourage other journals and groups to consider endorsing CHEERS.

Concluding remarks

As the number of published health economic evaluations continues to grow, we believe more transparent and complete reporting of methods and findings will be increasingly important to facilitate interpretation and comparison of studies. We hope the CHEERS statement, consisting of recommendations in a 24 item checklist, will be viewed as an effective consolidation and update of previous efforts and serve as a starting point for standard reporting going forward.

We believe the CHEERS statement represents a considerable expansion over previous efforts. The strength of our approach is that it was developed in accordance with current recommendations for the development of reporting guidelines, using an international and multidisciplinary team of editors and content experts in economic evaluation and reporting.[22] Similar to the approach taken with other widely accepted guidelines, we have defined a minimum set of criteria though a modified Delphi technique and have translated these into recommendations, an explanatory document with explanations, and a checklist. Unlike some previous reporting guidance for economic evaluation, we have also made every effort to be neutral about the conduct of economic evaluation, allowing analysts the freedom to choose different methods.

There may be several limitations to our approach. A larger Delphi panel with a different composition could have led to a different final set of recommendations.[24] Some less common approaches and contexts (such as public health, developing countries, and system dynamic models) for conducting health economic evaluation may not be well represented by our sample of experts. Additionally, like many Delphi panel processes, we based decisions to reject or accept criteria on arbitrary levels of importance. However, we feel the group recruited to create the statement is sufficiently knowledgeable of the more common applications of economic evaluation, and the rules used to select criteria were created a priori and are consistent with previous efforts.

We believe it will be important to evaluate the effects of implementation of this statement and checklist on reporting in future economic evaluations. As methods for the conduct of economic evaluation continue to evolve, it will also be important to revisit or extend the guidance. The CHEERS Task Force feels that this statement should be reviewed for updating five years from its release.

Competing interests: All authors have completed the ICMJE uniform disclosure form at www.icmje.org/coi_disclosure.pdf and declare: FA served as board member for the study funder; FA, AHB, CC, MD, DG, DH, EL, JM, and SP were provided support for travel to a face-to-face meeting to discuss the contents of the report; FA and MD have received payment from the study sponsor for serving as co-editors for *Value in Health*; no other relationships or activities that could appear to have influenced the submitted work.

Elizabeth Loder—*BMJ* clinical epidemiology editor—played no part in the peer review or decision making of this paper at the editorial level, and contributed solely as an author.

The International Society for Pharmacoeconomics and Outcomes Research (ISPOR) Health Economic Evaluation Publication Guidelines—CHEERS Task Force acknowledge the support of Elizabeth Molsen; Donna Rindress, who provided the initial leadership for this effort; and the reviewers and Delphi panel participants, who are named in the larger explanation and elaboration document and on the CHEERS Task Force website.

Contributors: All authors provided a substantial contribution to the design and interpretation of the protocol and guidance, as well as writing sections of drafts, revising based on comments received, and approving the final version. DH conducted the analysis of Delphi panel responses, drafted and revised the protocol and the drafts of this paper, and is the guarantor for the study.

Funding: All CHEERS Task Force members are volunteers. Support for this initiative was provided by the International Society for Pharmacoeconomics and Outcomes Research.

1 Drummond MF, Sculpher MJ, Torrance G, O'Brien J, Stoddart GL. *Methods for the economic evaluation of health care programmes* . 3rd ed. Oxford University Press, 2005.
2 Drummond MF, Schwartz JS, Jönsson B, Luce BR, Neumann PJ, Siebert U, et al. Key principles for the improved conduct of health technology assessments for resource allocation decisions. *Int J Technol Assess Health Care* 2008;24:244-58.
3 Rennie D, Luft HS. Pharmacoeconomic analyses. *JAMA* 2000;283:2158-60.
4 Neumann PJ, Stone PW, Chapman RH, Sandberg EA, Bell CM. The quality of reporting in published cost-utility analyses, 1976-1997. *Ann Intern Med* 2000;132:964.
5 Rosen AB, Greenberg D, Stone PW, Olchanski NV, Neumann PJ. Quality of abstracts of papers reporting original cost-effectiveness analyses. *Med Decis Making* 2005;25:424-8.
6 Turner L, Shamseer L, Altman DG, Schulz KF, Moher D. Does use of the CONSORT Statement impact the completeness of reporting of randomised controlled trials published in medical journals? A Cochrane review. *Syst Rev* 2012;1:60.
7 Drummond MF. A reappraisal of economic evaluation of pharmaceuticals. Science or marketing? *Pharmacoeconomics* 1998;14:1-9.
8 McGhan WF, Al M, Doshi JA, Kamae I, Marx SE, Rindress D. The ISPOR Good Practices for Quality Improvement of Cost-Effectiveness Research Task Force report. *Value Health* 2009;12:1086-99.
9 Task Force on Principles for Economic Analysis of Health Care Technology. Economic analysis of health care technology. A report on principles. *Ann Intern Med* 1995;123:61-70.
10 Drummond MF, Jefferson TO. Guidelines for authors and peer reviewers of economic submissions to the BMJ. *BMJ* 1996;313:275-83.
11 Gold MR. *Cost-effectiveness in health and medicine* . Oxford University Press, 1996.
12 Siegel JE, Weinstein MC, Russell LB, Gold MR. Recommendations for reporting cost-effectiveness analyses. Panel on Cost-Effectiveness in Health and Medicine. *JAMA* 1996;276:1339-41.
13 Nuijten MJ, Pronk MH, Brorens MJ, Hekster YA, Lockefeer JH, de Smet PA, et al. Reporting format for economic evaluation: Part II: Focus on modelling studies. *Pharmacoeconomics* 1998;14:259-68.
14 Vintzileos AM, Beazoglou T. Design, execution, interpretation, and reporting of economic evaluation studies in obstetrics. *Am J Obstet Gynecol* 2004;191:1070-6.
15 Drummond M, Manca A, Sculpher M. Increasing the generalizability of economic evaluations: recommendations for the design, analysis, and reporting of studies. *Int J Technol Assess Health Care* 2005;21:165-71.

No commercial reuse: See rights and reprints http://www.bmj.com/permissions Subscribe: http://www.bmj.com/subscribe

For peer review only - http://bmjopen.bmj.com/site/about/guidelines.xhtml

BMJ 2013;346:f1049 doi: 10.1136/bmj.f1049 (Published 25 March 2013)

16 Ramsey S, Willke R, Briggs A, Brown R, Buxton M, Chawla A, et al. Good research practices for cost-effectiveness analysis alongside clinical trials: the ISPOR RCT-CEA Task Force report. *Value Health* 2005;8:521-33.

17 Goetghebeur MM, Wagner M, Khoury H, Levitt RJ, Erickson LJ, Rindress D. Evidence and value: impact on decisionmaking—the EVIDEM framework and potential applications. *BMC Health Serv Res* 2008;8:270.

18 Davis JC, Robertson MC, Comans T, Scuffham PA. Guidelines for conducting and reporting economic evaluation of fall prevention strategies. *Osteoporos Int* 2010;22:2449-59.

19 Petrou S, Gray A. Economic evaluation using decision analytical modelling: design, conduct, analysis, and reporting. *BMJ* 2011;342:d1766.

20 Petrou S, Gray A. Economic evaluation alongside randomised controlled trials: design, conduct, analysis, and reporting. *BMJ* 2011;342:d1548.

21 Husereau D, Drummond M, Petrou S, Carswell C, Moher D, Greenberg D, et al. Consolidated Health Economic Evaluation Reporting Standards (CHEERS)—explanation and elaboration: a report of the ISPOR Health Economic Evaluations Publication Guidelines Task Force. *Value in Health* (forthcoming).

22 Moher D, Schulz KF, Simera I, Altman DG. Guidance for developers of health research reporting guidelines. *PLoS Med* 2010;7:e1000217.

23 Moher D, Simera I, Schulz K, Miller D, Grimshaw J, Hoey J, et al. Reporting guidelines for clinical research: a systematic review. Vancouver, BC, 2009. Available at: www.ama-assn.org/public/peer/abstracts-0912.pdf.

24 Campbell SM, Hann M, Roland MO, Quayle JA, Shekelle PG. The effect of panel membership and feedback on ratings in a two-round Delphi survey: results of a randomized controlled trial. *Med Care* 1999;37:964-8

Cite this as: *BMJ* 2013;346:f1049

This is an open-access article distributed under the terms of the Creative Commons Attribution Non-commercial License, which permits use, distribution, and reproduction in any medium, provided the original work is properly cited, the use is non commercial and is otherwise in compliance with the license. See: http://creativecommons.org/licenses/by-nc/2.0/ and http://creativecommons.org/licenses/by-nc/2.0/legalcode.

No commercial reuse: See rights and reprints http://www.bmj.com/permissions Subscribe: http://www.bmj.com/subscribe

BMJ 2013;346:f1049 doi: 10.1136/bmj.f1049 (Published 25 March 2013)

Table

Table 1| **CHEERS checklist—Items to include when reporting economic evaluations of health interventions**

Section/item	Item No	Recommendation	Reported on page No/ line No
**Title and abstract**			
Title	1	Identify the study as an economic evaluation or use more specific terms such as "cost-effectiveness analysis", and describe the interventions compared.	P1 L1
Abstract	2	Provide a structured summary of objectives, perspective, setting, methods (including study design and inputs), results (including base case and uncertainty analyses), and conclusions.	P2 L24
**Introduction**			
Background and objectives	3	Provide an explicit statement of the broader context for the study.	P4 L81
		Present the study question and its relevance for health policy or practice decisions.	P6 L113
**Methods**			
Target population and subgroups	4	Describe characteristics of the base case population and subgroups analysed, including why they were chosen.	P7 L135
Setting and location	5	State relevant aspects of the system(s) in which the decision(s) need(s) to be made.	P7 L137
Study perspective	6	Describe the perspective of the study and relate this to the costs being evaluated.	P8 L165
Comparators	7	Describe the interventions or strategies being compared and state why they were chosen.	P8 L160
Time horizon	8	State the time horizon(s) over which costs and consequences are being evaluated and say why appropriate.	P8 L167
Discount rate	9	Report the choice of discount rate(s) used for costs and outcomes and say why appropriate.	P8 L173
Choice of health outcomes	10	Describe what outcomes were used as the measure(s) of benefit in the evaluation and their relevance for the type of analysis performed.	P8 L164
Measurement of effectiveness	11a	*Single study-based estimates:* Describe fully the design features of the single effectiveness study and why the single study was a sufficient source of clinical effectiveness data.	P6 L118
	11b	*Synthesis-based estimates:* Describe fully the methods used for identification of included studies and synthesis of clinical effectiveness data.	N/A
Measurement and valuation of preference based outcomes	12	If applicable, describe the population and methods used to elicit preferences for outcomes.	P10 L213
Estimating resources and costs	13a	*Single study-based economic evaluation:* Describe approaches used to estimate resource use associated with the alternative interventions. Describe primary or secondary research methods for valuing each resource item in terms of its unit cost. Describe any adjustments made to approximate to opportunity costs.	P8 L175
	13b	*Model-based economic evaluation:* Describe approaches and data sources used to estimate resource use associated with model health states. Describe primary or secondary research methods for valuing each resource item in terms of its unit cost. Describe any adjustments made to approximate to opportunity costs.	N/A
Currency, price date, and conversion	14	Report the dates of the estimated resource quantities and unit costs. Describe methods for adjusting estimated unit costs to the year of reported costs if necessary. Describe methods for converting costs into a common currency base and the exchange rate.	P9 L182
Choice of model	15	Describe and give reasons for the specific type of decision-analytical model used. Providing a figure to show model structure is strongly recommended.	N/A
Assumptions	16	Describe all structural or other assumptions underpinning the decision-analytical model.	N/A
Analytical methods	17	Describe all analytical methods supporting the evaluation. This could include methods for dealing with skewed, missing, or censored data; extrapolation methods; methods for pooling data; approaches to validate or make adjustments (such as half cycle corrections) to a model; and methods for handling population heterogeneity and uncertainty.	P11 L216
**Results**			
Study parameters	18	Report the values, ranges, references, and, if used, probability distributions for all parameters. Report reasons or sources for distributions used to represent uncertainty where appropriate. Providing a table to show the input values is strongly recommended.	P13 L282
Incremental costs and outcomes	19	For each intervention, report mean values for the main categories of estimated costs and outcomes of interest, as well as mean differences between the comparator groups. If applicable, report incremental cost-effectiveness ratios.	P14 L305
Characterising uncertainty	20a	*Single study-based economic evaluation:* Describe the effects of sampling uncertainty for the estimated incremental cost and incremental effectiveness parameters, together with the impact of methodological assumptions (such as discount rate, study perspective).	P15 L317

No commercial reuse: See rights and reprints http://www.bmj.com/permissions Subscribe: http://www.bmj.com/subscribe

For peer review only - http://bmjopen.bmj.com/site/about/guidelines.xhtml

BMJ 2013;346:f1049 doi: 10.1136/bmj.f1049 (Published 25 March 2013)

(continued)

Section/item	Item No	Recommendation	Reported on page No/ line No
	20b	*Model-based economic evaluation:* Describe the effects on the results of uncertainty for all input parameters, and uncertainty related to the structure of the model and assumptions.	N/A
Characterising heterogeneity	21	If applicable, report differences in costs, outcomes, or cost-effectiveness that can be explained by variations between subgroups of patients with different baseline characteristics or other observed variability in effects that are not reducible by more information.	P14-P15
**Discussion**			
Study findings, limitations, generalisability, and current knowledge	22	Summarise key study findings and describe how they support the conclusions reached. Discuss limitations and the generalisability of the findings and how the findings fit with current knowledge.	P15L331
**Other**			
Source of funding	23	Describe how the study was funded and the role of the funder in the identification, design, conduct, and reporting of the analysis. Describe other non-monetary sources of support.	P21L451
Conflicts of interest	24	Describe any potential for conflict of interest of study contributors in accordance with journal policy. In the absence of a journal policy, we recommend authors comply with International Committee of Medical Journal Editors recommendations.	P21L458

For consistency, the CHEERS statement checklist format is based on the format of the CONSORT statement checklist

No commercial reuse: See rights and reprints http://www.bmj.com/permissions Subscribe: http://www.bmj.com/subscribe

Cost-effectiveness of habit-based advice for weight control versus usual care in general practice in the Ten Top Tips (10TT) Trial: economic evaluation based on a randomised controlled trial

Journal:	*BMJ Open*
Manuscript ID	bmjopen-2017-017511.R2
Article Type:	Research
Date Submitted by the Author:	22-Nov-2017
Complete List of Authors:	Patel, Nishma; UCL, Department of Applied Health Research<br>Beeken, Rebecca; University of Leeds, Leeds Institute of Health Sciences Clarendon Way LS2 9NL<br>Leurent, Baptiste; London School of Hygiene and Tropical Medicine, Department of Infectious Disease Epidemiology<br>Omar, Rumana; University College London,<br>Nazareth, Irwin; UCL, Primary Care and Population Health<br>Morris, Stephen; University College London, Department of Applied Health Research
**Primary Subject Heading**:	Health economics
Secondary Subject Heading:	Public health, General practice / Family practice
Keywords:	HEALTH ECONOMICS, PRIMARY CARE, PUBLIC HEALTH

Cost-effectiveness of habit-based advice for weight control versus usual care in general practice in the Ten Top Tips (10TT) Trial: economic evaluation based on a randomised controlled trial

Nishma Patel[1], Rebecca J. Beeken[2,3], Baptiste Leurent[4], Rumana Z. Omar[5], Irwin Nazareth[6] and Stephen Morris[1]

[1] Department of Applied Health Research, University College London, Gower Street, London, WC1E 6BT, UK

[2] Department of Behavioural Science & Health, University College London, Gower Street, London, WC1E 6BT, UK

[3]Leeds Institute of Health Sciences, University of Leeds, Clarendon Way, Leeds, LS2 9NL

[4] London School of Hygiene and Tropical Medicine, Keppel Street, WC1E 7HT

[5] Department of Statistical Science, University College London, Gower Street, London, WC1E 6BT, UK

[6]Deparment of Primary Care & Population Health, UCL, Gower Street, London, WC1E 6BT

Correspondence to: Nishma Patel, Department of Applied Health Research, University College London WC1E 6BT, UK. Telephone: +44 2031 083027 (email: nishma.patel@ucl.ac.uk)

For peer review only

For peer review only - http://bmjopen.bmj.com/site/about/guidelines.xhtml

Abstract

Objective: Ten Top Tips (10TT) is a primary care led behavioural intervention, which aims to help adults reduce and manage their weight by following ten weight loss tips. The intervention promotes habit formation to encourage long-term behavioural changes. The aim of this study was to estimate the cost-effectiveness of 10TT in general practice from the perspective of the UK National Health Service.

Design: An economic evaluation was conducted alongside an individually randomised controlled trial.

Setting: 14 General Practitioner (GP) practices in England.

Participants: All patients were aged ≥18 years, with BMI ≥30kg/m^2. A total of 537 patients were recruited; 270 received the usual care offered by their practices and 267 received the 10TT intervention.

Outcomes measures: Health service use and quality-adjusted life years (QALY) were measured over two years. Analysis was conducted in terms of incremental Net Monetary Benefit (NMB), using non-parametric bootstrapping and multiple imputation.

Results: Over a two-year time horizon, mean costs and QALYs per patient in the 10TT group were £1889 (95% CI: £1522 to £2566) and 1.51 (95% CI: 1.44 to 1.58). The mean costs and QALYs for usual care were £1925 (95% CI: £1599 to £2251) and 1.51 (95% CI: 1.45 to 1.57), respectively. This generated a mean cost difference of -£36 (95%CI: -£511 to £440) and a mean QALY difference of 0.000 (95%CI: -0.080 to 0.082). The incremental NMB for 10TT versus usual care was £49 (95%CI: -£1709 to £1800) at a maximum willingness to pay for a QALY of £20,000. 10TT had a 52% probability of being cost-effective at this threshold.

For peer review only

For peer review only - http://bmjopen.bmj.com/site/about/guidelines.xhtml

Conclusions: Costs and QALYs for 10TT were not significantly different from usual care and therefore 10TT is as cost-effective as usual care. There was no evidence to recommend nor advice against offering 10TT to obese patients in general practices based on cost-effectiveness considerations.

Trial Registration: ISRCTN registry ISRCTN16347068

Article summary

Strengths and limitations of this study

- The analysis is based on a large multicentre randomised trial with detailed information on resource use and utility values for a median follow-up period of two years.
- Individual data on standard weight-loss interventions received by the participant were not recorded.
- The analysis took a UK NHS/PSS perspective but a wider perspective (e.g., societal) could have been taken, including costs to patients and families.
- The time horizon was two years but could have taken a longer time horizon.
- Missing NHS resource use and utility data was accounted for using multiple imputation.

For peer review only - http://bmjopen.bmj.com/site/about/guidelines.xhtml

For peer review only

Introduction

The worldwide prevalence of obesity nearly doubled between 1980 and 2008. Based on recent estimates in the European Union, overweight affects 30-70% and obesity affects 10-30% of adults(1). In the UK 23.9 % of women aged 18 years and over are obese- the highest proportion in Europe. The proportion of UK men who are obese is 22.1%, the second highest proportion in Europe behind Malta (24.7 %)(2).

Health risks associated with obesity range from heart disease, stroke and type 2 diabetes to all cancers, gall bladder disease and mortality(3). It is estimated that health problems associated with being overweight or obese cost the National Health Service (NHS) in England more than £5 billion every year(4).

Non-surgical interventions for weight loss are available and include dietary advice, physical activity and behaviour modification. These can be accessed mainly through primary care. Despite considerable investment in such interventions, there is limited evidence about their cost-effectiveness(5-9).

Ten Top Tips (10TT) was a simple leaflet-based intervention for patients in primary care designed to help with weight loss. The underpinning theory behind 10TT was habit formation. Psychological research shows that repetition of a simple action in a consistent context leads, through associative learning, to the action being activated upon subsequent exposure to those contextual cues(10). A small pilot trial consisting of 100 people showed that those who received the Ten Top Tips leaflet lost significantly more weight than those who did not receive it(11). Although the absolute weight loss was modest, the intervention had the potential to be disseminated at minimal cost to large numbers simply by making the

For peer review only - http://bmjopen.bmj.com/site/about/guidelines.xhtml

leaflet available. In response to this a large randomised trial was undertaken. Participants were given a leaflet, which provided weight loss tips based on scientific evidence on how to turn the tips into healthy habits, during a 30-minute appointment with a practice nurse. The ten healthy tips included: keeping to meal routines; reduced fat intake; walking off weight; packing a healthy snack; looking at food labels; taking caution with portion size; standing up; thinking about drinks; focusing on food; and, not forgetting 'five a day'. The 'five a day campaign is based on recommendation from the World Health Organisation (WHO) encouraging consumption of at least 400g of fruit and vegetables a day to lower the risk of serious health problems, such as heart disease, stroke and some cancers(12). Participants were asked to record their progress in a logbook. Using routinely collected data from primary care practices, the aim of the present study was to analyse the cost and cost-effectiveness of 10TT.

Methods

Trial background

The 10TT trial was a two-arm, individually randomised, controlled trial in which 537 obese men and women were enrolled. Practices across England (n=14) were recruited through the MRC General Practice Research Framework (GPRF). The majority were located in Southern England (n=9), with three located in the Midland and two in the North. Recruitment occurred between August 2010 and October 2011. Participants were randomly assigned to 10TT (n=267) or usual care (n=270), and followed for up to two years. The primary outcome was weight loss at three months. Secondary outcomes included BMI, waist circumference, the number of people achieving a 5% reduction in weight, clinical markers for potential co-morbidities (blood pressure, total cholesterol/low-density lipoprotein and blood glucose),

For peer review only - http://bmjopen.bmj.com/site/about/guidelines.xhtml

and maintenance of weight loss over 24 months. At three months participants in the 10TT group lost significantly more weight than those receiving usual care (mean difference in weight change=-0·87kg, 95% CI -1·47 to -0·27, p=0·004). But this effect was not maintained at 24 months (mean difference in weight change= +0·75kg (95% CI: -0.73 to 2.24)). Weight loss in the usual care group was slow in the first 6 months, but it continued until 18 months, whereas the 10TT group experienced a greater weight loss in the first 6 months, but did not lose any additional weight after this point.

Overview of economic evaluation

We undertook a cost-utility analysis to compare the costs and outcomes associated with 10TT and usual care. The outcome measure was quality-adjusted life years (QALYs), which combines length of life and quality of life, and is consistent with The National Institute for Health and Care Excellence (NICE) recommendations(13).The primary outcomes were the incremental costs and effects and the incremental net monetary benefit (NMB) of 10TT versus usual care. Costs were measured from a NHS and personal social services (PSS) perspective; since PSS costs were negligible, we focused on NHS costs. These were calculated in 2013/14 UK£. The time horizon for costs and effects was two years, reflecting the trial follow-up period. Extrapolation beyond this time period was not undertaken because the within-trial analysis found no evidence of significant differences in costs (-£36 (95% CI: -£511 to £440, p-value 0.88)) or outcomes between groups (0.001 (95% CI: -0.080 to 0.082, p-value 0.93)) at 24 months; two years was considered long enough to reflect all important differences in costs or outcomes between treatments. Costs and outcomes in the second year were discounted at 3.5%(13).

Resource use and costs

For peer review only - http://bmjopen.bmj.com/site/about/guidelines.xhtml

Resource use were extracted from GP records for two years prior to randomisation and two-years post randomisation on: the number and type of contacts with the GP (surgery visit, home visit, phone call); practice nurse contacts (practice visit, home visit, phone call); dietician visits; hospital outpatient visits; hospital inpatient stay; accident and emergency (A&E) visits; and outpatient services. Data on use of drugs included dose, type and frequency of administration. All resource use data was extracted by the practice nurse at participating centres from patient records.

Unit costs were attached to each resource item. The cost per visit to primary care (GP surgery visit, GP home visit, GP phone call, practice nurse visit, nurse home visit, practice nurse phone call and dietician visits) were taken from Personal Social Services Research Unit (PSSRU) estimates(14). The cost of inpatient episodes, outpatient visits and A&E visits were taken from the PSSRU and based on NHS Reference Costs(15). They included cost of medical staff, equipment, consumables, and diagnostics. The weighted average cost of inpatient stays was calculated combining the cost of inpatient long and short stay. Drug costs were obtained from British National Formulary(16).

The cost of the 10TT intervention included the following, which were valued using market prices: a logbook (£2.80 per participant); time patients spent with the practice nurse to introduce the programme (£20 for a 30-minute visit); wallet sized food label guidance (£0.09), and a 10TT leaflet (£0.05). The total cost of 10TT was £22.94 per participant. The cost of the food label guidance was based on the total cost of printing (£1,392) divided by the number of labels printed (15,000). Similarly, the cost of 10TT leaflets was based on a total cost of printing (£1,540) divided by the number of leaflets printed (30,000).

For peer review only

All practices offered standard weight-loss interventions as part of usual care. Standard weight loss interventions consisted of a range of interventions including referrals to community programmes, gym prescription and referral to a dietician and/or psychologist, among others. While data were provided by practices on interventions typically offered to their patient, the uptake of these interventions was not recorded. In our base case, we made the conservative assumption (possibly biased against 10TT) that no patients received these interventions. In a sensitivity analysis, we included the costs of these interventions assuming each patient in the control group received the standard weight-loss intervention offered by the practice. Where single interventions were offered by a practice these were costed accordingly, based on the average cost of participating in Weight Watchers (assumed to cost £78.22 per patient(17)) or Slimming World (£72.62 per patient). Where multiple interventions were offered by a practice these were costed based on the average cost of participating in the Size Down Programme at a cost of £93.48 per patient(17).

The unit cost (total expediture incurred by the NHS for one visit) for each cost component are shown in Table 1. Unit costs were multiplied by resource volume and summed across all cost components to obtain a total cost per patient.

Utilities and QALYs

Health utilities were based on the EuroQol standardised measure of health status, EQ-5D-3L descriptive system (18, 19). This is a five dimensional questionnaire (mobility, self-care, usual activities, pain and discomfort), with three levels in each dimension (severe problems, some problems, no problems). Each EQ-5D state was converted into a single utility score based on valuations from the UK general population(20). Utility values of one represent full health, values of zero are equivalent to death, negative values represent states worse than

For peer review only - http://bmjopen.bmj.com/site/about/guidelines.xhtml

death. The EQ-5D questionnaire was completed at baseline, three months, six months, 12 months, 18 months and 24 months. A utility profile was constructed for participants assuming a straight-line relation between their utility values at each measurement point. QALYs for every patient from baseline to two years were calculated as the area under the utility profile.

Dealing with Missing Data

There were missing data for NHS resource use and utility scores. ~~Multivariate imputation~~Multivariate imputation by chained equations was used to impute missing data (21, 22). The imputation method used an iterative Markov Chain Monte Carlo (MCMC) technique to simulate from the posterior predictive distribution of missing data ((23). We generated 20 imputed datasets. We imputed missing data for the following variables: weight (0.2%); BMI (0.2%); waist circumference (0.6%); EQ-5D at baseline (5%), 3 months (26%), 6 months (40%), 12 months (46%) and 24 months (46%); NHS visits; GP practice (28%); GP home (31%); GP phone calls (30%); practice nurse (27%); nurse home (31%); nurse phone calls (31%); extra nurse (30%); dietician (31%); hospital inpatient stay (31%); outpatient clinic (30%); A&E visits (30%) and other outpatient service visits (69%) (Table 1). Age, sex and study centre were included in the imputation model as additional explanatory variables. Imputations were undertaken using the –mi impute mvn– command in Stata SE version 14 (Stata Corp, College Station, TX)(24).

Statistical analysis

Two sample t-tests to test for differences in cost and QALYs between the two groups were carried out for complete data. A linear regression model was used to test for differences in

For peer review only - http://bmjopen.bmj.com/site/about/guidelines.xhtml

mean resource use, costs, EQ-5D scores at each time point, and QALYs (25, 26) using the imputed data. Probabilistic uncertainty using Monte Carlo Simulations (27) were used to resample each estimated (non-sampled) variable to account for uncertainty around point estimates: the difference in mean costs, QALYs and NMBs. The incremental NMBs were calculated as the difference in mean QALYs per participant (*Q*) with 10TT(*T*) versus usual care(*U*) multiplied by the maximum willingness to pay for a QALY (*R*) minus the difference in mean cost per participant (*C*), i.e., incremental NMB = $(Q_T - Q_U)*R - (C_T - C_U)$. We used the cost-effectiveness threshold range recommended by NICE (£20 000 to £30 000 (13) as the lower and upper limits of the maximum willingness to pay for a QALY (*R*). Negative incremental NMBs indicate that usual care is preferred on cost-effectiveness grounds and positive incremental NMBs indicate that 10TT is preferred (28). A linear regression using the ordinary least squares (OLS) regression was used to estimate the mean difference in observed QALYs   between the two trial; age, gender, practice and baseline utility values. Incremental costs were initially adjusted for age, gender, practice and prior costs in the previous two years (generalised linear model with gamma family and log link to account for skewness). Adjusting for these factors did not make significant difference to the results (Table 2) and they were therefore not included in the final model.

For each of the 20 imputed datasets we ran 1000 bootstrap replications using non-parametric bootstrapping, resampling observations with replacement. The bootstrapped results were combined using the formula described by Briggs et al (22), to calculate standard errors around mean values allowing for uncertainty around the imputed values, skewness of cost and utility data, and sampling variation. Standard errors were on based a normal distribution and used to calculate 95% CIs around point estimates.

For peer review only - http://bmjopen.bmj.com/site/about/guidelines.xhtml

The cost-effectiveness plane (figure 1) was used to illustrate the difference in costs and outcomes between the two groups on a graph. The probability that 10TT was cost-effective in comparison to usual care at different values of the cost-effectiveness threshold was presented in form of a cost-effectiveness acceptability curve (CEAC) (figure 2)(29). The CEAC was based on the proportion of bootstrap replications across all twenty imputed datasets that were below the cost-effectiveness threshold, which was varied from £0 to £50,000.

Several deterministic sensitivity analyses were carried out to assess the uncertainty around key components of the analysis. An analysis was undertaken based on complete cases without any imputation. We varied cost components for primary and secondary care costs and drug costs. The total costs of each component were amended by ±10%. We also conducted an analysis including the cost of standard weight-loss interventions for the usual care group.

Results

Baseline description

Table 3 illustrates the mean costs, two years prior to the trial. The mean cost for the usual care group was £1848 (95% CI: 1615, 2082), and £2052 (95% CI: 1756, 2349) in the 10TT group. Baseline characteristics- age, gender, weight, BMI blood pressure and cholesterol did not differ between groups(30).

Base case

Table 1 shows mean imputed resource use by treatment group. The intervention and control groups had the same mean GP practice visits per participant over the two-year period (9.4 versus 9.4), as well as similar GP home visits (0.0 versus 0.1), GP phone contacts

For peer review only - http://bmjopen.bmj.com/site/about/guidelines.xhtml

(1.3 versus 1.3), nurse practice visits (6.6 versus 6.9), nurse home visits (0.0 versus 0.1), inpatient episodes (0.2 versus 0.2), outpatient clinic visits (2.1 versus 2.2) and accident and emergency visits (0.4 versus 0.4). There was no statistically significant difference between groups for any category of health service resource use.

Table 3 shows the total cost per participant in each group. The non-parametric bootstrapping including imputed resource use produced mean total costs per participant in the intervention group of £1889 (95% CI: £1522 to £2566), compared to £1925 (95% CI: £1599 to £2251) in the control group. The mean difference in cost between 10TT and control group was-£36 (95%CI: -£511 to £440), which was not statistically significant (p=0.88) .

Undertaking non-parametric bootstrapping after multiple imputation produced 1.51 (95% CI: 1.44 to 1.58) QALYs in the intervention group and 1.51 (95% CI: 1.45 to 1.57) in the control group. Generating a mean difference in QALYs of 0.000 (95%CI: -0.080 to 0.082) (p=0.93) (Table 4 and 5) which was not statistically significant. Hence, patients receiving 10TT accrued non-significantly lower costs and the difference in QALYs between the groups was very small.

The incremental net monetary benefit for 10TT versus usual care was £49 (95% CI: -£1709 to £1800) at a maximum willingness to pay for a QALY of £20,000 and £55 (95% CI: -£2489 to £2583) at a maximum willingness to pay for a QALY of £30,000 (Table 4).

Of the 20,000 bootstrap replications produced under base case assumptions, 24% fell into south-west quadrant of the cost-effectiveness plane (10TT was less costly and less effective than usual care); 20% fell into the north-east quadrant (10TT was more costly and more

For peer review only - http://bmjopen.bmj.com/site/about/guidelines.xhtml

effective than usual care); 25% fell into the north-west quadrant (10TT was more costly and less effective than usual care); and 31% fell into the south-east quadrant (10TT was less costly and more effective than usual care) (Figure 1). The CEAC derived from Figure 1 is illustrated in Figure 2. Under base case assumptions, 10TT has a 52% probability of being cost-effective at a willingness to pay threshold of £20,000 and 52% at a willingness to pay threshold of £30,000 (Table 4).

Sensitivity analyses

Including the cost of the standard weight-loss interventions (£87) in the usual care group the mean costs per patient in the control group increased to £2012, which was higher than the mean cost in the intervention group (£1889). The mean incremental cost difference between groups was -£122 (95% CI: -£598 to £353). The incremental net monetary benefit for 10TT versus usual care was £140 (95% CI: (-£1666 to £1902) at a maximum willingness to pay for a QALY of £20,000 and £148 (95% CI: -£2463 to £2693) at a maximum willingness to pay of £30,000. Increasing and decreasing costs by 10% did not affect the findings appreciably (Table 4).

A complete case analysis was undertaken using 163 of 537 participants with complete utility and cost data. The incremental NMB was -£877 (95% CI: -£2338 to £3404) at a maximum willingness to pay for a QALY of £20,000 and -£1349 (95% CI: -£3269 to £4972) at a maximum willingness to pay for a QALY of £30,000.

Discussion

Our economic analysis of 10TT showed that this intervention had similar costs and QALYs as usual care. Sensitivity analyses showed little uncertainty in this finding. On the one hand the

For peer review only - http://bmjopen.bmj.com/site/about/guidelines.xhtml

findings mean there is no reason to prefer 10TT or usual care on the basis of differences in quality of life or cost, or on cost-effectiveness grounds. On the other hand, this means that 10TT is as cost-effective as usual care.

We undertook a rapid review to assess the comparability of our results to similar weight loss programs. We identified six studies, which ranged from primary care led programmes such as lifestyle counselling (8), commercial weight loss programme (6, 7), patient education and the combination of diet and exercise over 12 months to 24 month period, compared with usual care. 10TT compliments the existing studies by illustrating the implications of such programs in both the short-term and long-term. Our findings showed that weight loss programmes such as Weight Watchers lost more weight than education programmes and resulted in the possibility of cost savings, which we outline below. Fuller et al(7) reports the cost-effectiveness of standard care versus a commercial weight loss programme (Weight Watchers (CP)) in a population of 772 participants from Australia, UK and Germany, over a timeframe of one year. The cost per kilogram of weight loss was US$122 (Australia), US$90 (UK) and US$180 (Germany) for the commercial provider and US$138 (Australia), US$151 (UK) and US$133 (Germany) for standard care. The incremental cost per QALY gained of the commercial programme versus standard care was US$18,266 (Australia), US$12,100 (UK) and US$40,933 (Germany). A further collection of follow-up data at two-years was used to develop a decision analytic model to estimate the lifetime costs and outcomes in the Australian population. Over a lifetime of the patient, the commercial weight loss programme resulted in an incremental cost saving of US$47 per patient(6, 31), suggesting that the commercial programme may be cost effective. When analysing completed Quality of Life questionnaires (IWQOL-Lite) there was a significantly greater change in utility for

For peer review only

commercial weight loss programme (Weight Watchers (CP)) for Australia and Germany but not in the UK. However, this may have been due to the small sample size. When an intention to treat analysis was undertaken, CP was the preferred approach and the difference in utility scores between CP and standard care (SC) was reported statistically significant for Australia (0.0006) and the UK (0.033). A long-term analysis of the trial suggested over a lifetime of the patient resulted in a cost saving of US$47 per patient and an incremental 0.03 QALY gained per patient(6), however these differences were not statistically significant.

Upon examination of two self-directed programs, an evaluation of obesity treatment in Pennsylvania (USA), with 390 participants looked at the cost-effectiveness of usual care (quarterly visits to the primary care provider), lifestyle counselling (quarterly visits to the primary care provider plus monthly weight loss counselling) and enhanced lifestyle counselling (all the above plus meal replacements and weight loss medication). The study showed the incremental cost per kilogram lost was US$292 for the enhanced lifestyle counselling compared to usual care. Long-term estimates using published data and trial data suggested Brief Lifestyle Counselling would fall somewhere between US$7000 and US$12600 cost per QALY; Enhanced Brief lifestyle counselling the cost per kilogram could be as high as US$22000(8).

For overweight and obese women, a three component intervention of diet, exercise and behaviour change in the USA produced a cost per QALY gained of US$12,600 compared with usual care, exercise, and diet alone (5). The authors concluded the three-component programme was good value for money.

For peer review only - http://bmjopen.bmj.com/site/about/guidelines.xhtml

The primary care led programCounterweight (a nurse-delivered patient education programme) showed that the intervention was dominant (less costly and more effective) compared with no active intervention; it produced a gain in QALYs (0.06 per participant) and cost savings of £27 per participant (9).

Finally, in the UK a recent evaluation of a primary care led behavioural programme(32) looking at a brief advice and self-help materials, weight management programme over 12 weeks and the same weight management programme over 52 weeks concluded that a weight loss programme over 12 weeks is more effective (-4.75kg) than brief advice and self-help material (-3.26kg) and a 52 weeks programme produces greater weight loss (-6.76kg) and was £159 per kg lost, producing additional clinical benefits. The ICER for the 52- week programme when compared to the brief intervention was £2394 per QALY(32).

The differences in the results of the studies above may be explained by different settings in which the intervention is executed, the type of interventions being evaluated and finally, the practice of usual care, which may differ across countries and healthcare practitioners.

Overall, existing literature for weight loss programmes has grown, prompting clinicians to consider referring patients to primary care led weight loss programmes that show cost-effectiveness. evidence,tThere is a possibility that 10TT alongside other programmes would be effective at a low-cost.

The main strength of our analysis is that it is based on a large multicentre randomised trial with detailed information on resource use and utility values for a median follow-up period of two years. There were several limitations to our study. While data were recorded in the

For peer review only - http://bmjopen.bmj.com/site/about/guidelines.xhtml

trial on standard weight-loss interventions for obese patients at each practice, individual data on the uptake and prescribing of standard weight-loss interventions received by the participant were not recorded. This trial was also unblinded, which introduced the potential bias of GPs over prescribing standard weight-loss interventions producing a healthy user bias effect. Under base case assumptions, excluding standard weight-loss interventions, the incremental NMB of 10TT versus usual care was not significantly different from zero, and this did not change when intervention costs in the control group were included. Hence, these costs did not make an appreciable difference to our conclusions. Secondly, the analysis took a UK NHS/PSS perspective. A wider perspective (e.g., societal) could have been taken, including costs to patients and families. Third, the time horizon was limited to two years, due to the lack of difference in costs (-£36 (95%CI -£511 to £440, p-value 0.88) and benefits (0.000 (95% CI -0.080 to 0.082, p-value 0.93) at 24 months between groups. The trial did not capture QALYs from weight loss post 24 months. A longer follow-up period may have allowed for the detection of long-term health benefits produced as a consequence of avoiding obesity related health conditions, such as diabetes and cardiovascular disease. However, at the time of this study little was known about the association between habit-formation and weight loss. This study has identified the importance of longer-term strategies for continued adherence of weight loss. With add-on approaches such as counselling and education on how to maintain weight loss for participants alongside 10TT, it may be possible to maintain weight loss post two-years.

Fourth, while there are various instruments are available to measure Health related Quality of Life (HRQoL) associated with weight loss. We administered the EQ-5D(33). We acknowledge that there may have been a potential value in using more than one measure in

For peer review only - http://bmjopen.bmj.com/site/about/guidelines.xhtml

the trial, such as the SF-36 and Impact of Weight on Quality of Life –Lite (IWQOL-Lite) to measure differences in the positive short-term psychological effects(34). However, given the small difference between the two groups it is highly unlikely alternative measures would've produced significant differences. Finally, we were unable to access Hospital Episode Statistics (HES) data containing secondary care resource use of NHS services by patients. Obtaining HES data would been problematic due to the short timeframe of this trial and linking primary care data to HES data by patient ID(35). As a consequence, data for secondary care resource use were extracted from GP practices with a degree of missingness in our analysis. We accounted for this using multiple imputation, assuming data to be missing at random. It is important to note that when using such methods there is uncertainty around the non-observed value across the imputations. To account for the uncertainty around the values, we employed the non-parametric bootstrap approach to estimate the variance (a representation of uncertainty) around the true values (22). We acknowledge that though multiple imputation is able to statistically test for error, this method can produce bias. The bias arises from the assumption that missing data in the study were 'missing at random'. For example, the missing at random assumption may be reasonable if a variable that is predictive of missing data is included in the imputation model, but not if the variable is omitted from the model(36). However, multiple imputation offers an advantage over complete case analysis, by filling in missing values and enabling participants to be included in the analysis, where there were auxiliary variables that are strong predictors of missingness(37). Guidelines were followed to ensure the model predicted the best possible estimates; investigation of the proportion of missing data; selection of specific variables and; identification of the predictors of missingness(38). We acknowledge that as the percentage of missingness increases (in some cases more than

For peer review only - http://bmjopen.bmj.com/site/about/guidelines.xhtml

50%) there will be greater inefficiency in the imputed data and more chance of bias. However, in general the application of regression imputation with large missing data may be acceptable if adjustments are made for the predictions, as we have done(22).

In conclusion, the 10TT trial found that after three months' participants receiving 10TT lost significantly more weight than those receiving usual care, but there was no difference in weight change between the two groups at 24 months. Consistent with these findings, the present study found that differences in costs and QALYs between 10TT and usual care were not significantly different over the two-year period. There is no evidence to either recommend or advise against 10TT, based on cost-effectiveness considerations.

For peer review only

Acknowledgements

Weight Concern and Cancer Research UK developed the 10 Top Tips intervention and materials, and Weight Concern provided training on how to deliver the intervention. The General Practice Research Framework provided advice on the study design and was involved in both recruiting sites and training the health professionals at each site on the trial procedures, along with providing quality control visits. Practices were located in: Wellingborough, Southampton, Bradford-on-Avon, Bromsgrove, Frome, Guisborough, Glastonbury, Ivybridge, Dunstable, Liskeard, Ledbury, New Mills, and London. The authors would like to acknowledge the substantial intellectual contribution made by Professor Jane Wardle who sadly passed away prior to publication, and is deeply missed by all of her co-authors, colleagues and students.

Contributors

NP undertook data analysis, interpreted the results, drafted, and wrote the manuscript. RJB helped design the study, undertook data collection and critically reviewed the manuscript.

For peer review only - http://bmjopen.bmj.com/site/about/guidelines.xhtml

BL helped design the study, critically reviewed the data and manuscript. RZO helped design the study and critically reviewed the manuscript. IN helped design the study and critically reviewed the manuscript. SM undertook the data analysis, devised the analysis plan, interpreted the results and critically reviewed the manuscript.

Funding

This work was funded by the Medical Research Council (MRC) – National Prevention Research Initiative (NPRI) (UK) grant number G080202. RJB's post was funded through this grant and she is now supported by Yorkshire Cancer Research Academic Fellowship funding. SM, NP and IN are HEFCE-funded. RZO was funded by the National Institute for Health Research.

Disclaimer

The views expressed are those of the authors and not necessarily those of the funding bodies.

Competing interests

All authors have completed the ICMJE uniform disclosure form at http://www.icmje.org/coi_disclosure.pdf (available on request from the corresponding author) and declare that: NP, RJB, BL, RZO, IN and SM have nothing to disclose.

Ethics approval

This study has been approved by the South East London REC 2, 23/08/2010 (Reference number 10/H0802/59). All participants gave informed consent before taking part.

Provenance and peer review

Not commissioned; externally peer reviewed.

For peer review only

450 **Data sharing statement**

451 No additional data are available.

For peer review only

For peer review only - http://bmjopen.bmj.com/site/about/guidelines.xhtml

Table 1. Resource use, unit cost, and mean cost per participant for primary and secondary care services post randomisation

		Usual Care					10TT					Usual Care	10TT	Incremental difference	
	N	Mean	(SD)	Median	(IQR)	N	Mean	(SD)	Median	(IQR)	Unit cost (£)	Mean cost (£)	Mean cost (£)	Mean (£)	(95% CI)
**No imputation for missing values**															
GP surgery visit	204	9.4	(8.4)	7	(7.00)	182	9.7	(9.5)	7	(9.00)	45	417	425	-8	(-89, 72)
GP home visit	196	0.1	(0.8)	0	(0.00)	176	0.0	(0.1)	0	(0.00)	292	37	9	-28	(-7, 61)
GP phone	199	1.3	(3.0)	0	(1.00)	179	1.3	(3.0)	0	(1.00)	27	34	34	0	(-16, 16)
GP practice nurse	206	6.9	(8.4)	4	(6.00)	184	6.8	(9.5)	4	(6.50)	40	274	268	-6	(-64, 77)
Nurse home visit	195	0.1	(1.1)	0	(0.00)	175	0.0	(0.1)	0	(0.00)	70	5	1	-4	(-7, 16)
GP practice nurse phone	197	0.5	(2.3)	0	(0.00)	176	0.4	(1.2)	0	(0.00)	10	5	4	-1	(-2, 5)
Dietician	195	0.1	(0.5)	0	(0.00)	175	0.1	(0.8)	0	(0.00)	35	4	4	0	(-4, 4)
Additional nurse visit	197	1.1	(3.1)	0	(1.00)	179	1.6	(2.6)	0	(1.00)	4	45	44	-1	(-22, 23)
Inpatient admission	196	0.2	(0.7)	0	(0.00)	175	0.2	(0.7)	0	(0.00)	1713	372	387	-15	(-249, 220)
Outpatient clinic	200	2.2	(3.5)	1	(3.00)	178	2.1	(3.3)	1	(3.00)	135	288	269	-19	(-70, 110)
Accident & Emergency visit	199	0.4	(1.2)	0	(0.00)	175	0.4	(0.9)	0	(1.00)	177	65	67	-2	(-39, 36)
Other outpatient services	194	1.0	(1.8)	0	(1.00)	179	1.0	(2.3)	0	(1.00)	135	124	129	-5	(-58, 48)
**Missing values imputed***			SE					SE							
GP surgery visit	270	9.4	(0.6)	9		267	9.4	(0.7)	9		45	419	420	-1	(-79,81)
GP home visit	270	0.1	(0.5)	0		267	0.0	(0.2)	0		292	36	13	-23	(-58, 12)
GP phone	270	1.3	(0.2)	1		267	1.3	(0.2)	1		27	35	33	-2	(-19, 14)
GP practice nurse	270	6.9	(0.6)	6		267	6.6	(0.7)	6		40	274	266	-8	(-75, 57)
Nurse home visit	270	0.1	(1.0)	0		267	0.0	(0.1)	0		70	6	2	-4	(-16, 7)
GP practice nurse phone	270	0.5	(0.6)	0		267	0.4	(0.1)	0		10	5	4	-1	(-5, 2)
Dietician	270	0.1	(0.0)	0		267	0.1	(0.6)	0		35	4	4	0	(-4, 4)
Additional nurse visit	270	1.2	(0.2)	1		267	1.8	(0.2)	0		4	46	47	-1	(-23, 23)

For peer review only - http://bmjopen.bmj.com/site/about/guidelines.xhtml

Inpatient admission	270	0.2	(0.1)	0		267	0.2	(0.1)	0		1713	373	379	-6	(-238, 251)
Outpatient clinic	270	2.1	(0.3)	2		267	2.0	(0.2)	0		135	287	266	-21	(-106, 63)
Accident & Emergency visit	270	0.4	(0.9)	0		267	0.4	(0.7)	0		177	67	69	-2	(-38, 42)
Other outpatient services	270	1	(0.1)	1		267	1	(0.2)	0		135	122	129	-7	(-52, 65)
**Weight loss interventions**															
10TT		-	-	-	-		-	-	-	-	23	-	-		
Weight Watchers		-	-	-	-		-	-	-	-	78	-	-		
Slimming World		-	-	-	-		-	-	-	-	93	-	-		
Size Down Programme		-	-	-	-		-	-	-	-	73	-	-		

GP = general practitioner. SD = standard deviation for non-imputed data. SE = standard error for imputed data. CI = confidence interval. IQR = inter-quartile range. Costs are in 2013/14 UK£. *Missing values imputed using multiple imputation with 20 imputed datasets (see text).

Table 2. Demographics at baseline

	Usual care			10TT		
	N	Mean*	SD	N	Mean	SD
Age	270	58	12.61	267	57	12.88
Male	95	35.2	-	89	33.3	-
Female	175	64.8	-	178	66.7	-
Prior costs	270	1848	1948	267	2052	2461
Baseline EQ-5D	257	0.76	0.24	255	0.74	0.27

SD = standard deviation. * Figures sex based on the mean proportion (%) in each group.

For peer review only

For peer review only - http://bmjopen.bmj.com/site/about/guidelines.xhtml

Table 3. Cost description two years prior and post randomisation

	Usual care			10TT			Incremental difference	
	**N**	**Mean**	**SD**	**N**	**Mean**	**SD**	**Mean (£)**	**(95% CI)**
**Cost, two years prior**								
Cost of primary and secondary care contacts	270	1516	1694	267	1735	2186	-219	(-550, 112)
Drugs costs	270	332	748	267	317	624	-15	(-102, 132)
Total cost	270	1848	1948	267	2052	2461	204	(-580, 172)
**Costs after randomisation (imputed)***			**SE**			**SE**		
Cost of primary and secondary care contacts	270	1675	2204	267	1631	2084	-44	(-477, 387)
Drugs costs	270	249	715	267	236	1114	-13	(-172, 146)
Intervention costs	-	-	-	267	23	-	-	-
Total cost	270	1925	2477	267	1889	2427	-36	(-511, 440)

SD = standard deviation for non-imputed data. SE = standard error for imputed data. CI = confidence interval

For peer review only - http://bmjopen.bmj.com/site/about/guidelines.xhtml

Table 4. Incremental cost-effectiveness of 10TT versus usual care

	Incremental cost		Incremental QALYs		Incremental net monetary benefit (NMB)			
					£20,000		£30,000	
	Mean (£)	(95% CI)	Mean	(95% CI)	Mean (£)	(95% CI)	Mean (£)	
Base case*	-36	(-511, 440)	0.000	(-0.080, 0.082	49	(-1709, 1800)	55	(-2
Base case with control group intervention cost~	-122	(-598, 353)	0.000	(-0.080, 0.082)	140	(-1666, 1902)	148	(-2
Complete case analysis§	-66	(-907, 774)	-0.047	(-0.180, 0.086)	-877	(-2338, 3404)	-1349	(-3
Primary and secondary care cost increased #	-40	(-556, 475)	0.000	(-0.080, 0.081)	48	(-1743, 1851)	53	(-2
Drug cost increased #	23	(-396, 442)	0.000	(-0.080, 0.077)	-14	(-1734, 1716)	-9	(-2
All costs increased #	-39	(-554, 476)	0.000	(-0.080, 0.082)	43	(-1756, 1851)	46	(-2
Primary and secondary care cost decreased ¥	-31	(-463, 401)	0.000	(-0.080, 0.081)	54	(-1683, 1804)	64	(-2
Drug costs decreased ¥	26	(-387, 438)	0.000	(-0.080, 0.081)	0	(-1741, 1727)	11	(-2
All costs decreased ¥	-32	(-460, 395)	0.000	(-0.080, 0.081)	50	(-1682, 1811)	59	(-2

*N= 537. Data include values imputed using multiple imputation (see text). The 95% confidence intervals were derived from 1000 bootstrap replications of each of the 20 imputed datasets (see text). The incremental net monetary benefit and the probability 10TT is cost-effective are based on the QALYs gained and incremental costs and calculated at a maximum willingness to pay for a QALY of £20,000 and £30,000.

~ As for the base case analysis except standard weight-loss interventions (Weight Watchers, Slimming World, Size Down programme) are included in the control group.

§As for the base case analysis except there is no multiple imputation of missing values and the 95% confidence intervals were derived from 1000 bootstrap replications of a single dataset containing the n=72 participants in the 10TT group and n=91 participants in the usual care group with no missing values. The costs of interventions in the control group (Weight Watchers, Slimming World, Size Down programme) were excluded.

# As for the base case analysis except costs are increased by 10% for 10TT.

¥As for the base case analysis except costs are decreased by 10% for 10TT.

For peer review only - http://bmjopen.bmj.com/site/about/guidelines.xhtml

For peer review only

Table 5. Quality Adjusted Life Years (QALYs) per patient

	Usual Care				10TT				Incremental difference	
	**N**	**Mean**	**SD**	**95% CI**	**N**	**Mean**	**SD**	**95% CI**	**Mean**	**(95% CI)**
**No imputation for missing values**										
Utility at 3 months	206	0.77	0.23	(0.74, 0.80)	190	0.77	0.26	(0.73, 0.80)	0.00	(0.05, 0.05)
Utility at 6 months	163	0.76	0.24	(0.72, 0.80)	159	0.78	0.24	(0.74, 0.82)	0.02	(-0.03, 0.07)
Utility at 12 months	151	0.77	0.24	(0.73, 0.81)	138	0.74	0.27	(0.70, 0.79)	0.03	(, 0.09,0.03)
Utility at 18 months	138	0.75	0.25	(0.71, 0.79)	124	0.74	0.27	(0.69, 0.79)	0.01	(0.07, 0.05)
Utility at 24 months	157	0.76	0.25	(0.73, 0.80)	131	0.76	0.26	(0.72, 0.81)	0.00	(0.06, 0.06)
**Discounted QALYs**	92	1.52	0.40	(1.44 1.61)	73	1.47	0.47	(1.36, 1.58)	0.05	(0.18, 0.08)
**Missing values imputed***			**SE**				**SE**			
Utility at 3 months	270	0.77	0.016	(0.73, 0.80)	267	0.77	0.017	(0.71, 0.78)	-0.01	(0.05, 0.04)
Utility at 6 months	270	0.76	0.016	(0.73, 0.79)	267	0.78	0.018	(0.73, 0.81)	-0.01	(0.06, 0.03)
Utility at 12 months	270	0.76	0.019	(0.73, 0.79)	267	0.75	0.020	(0.71, 0.80)	-0.01	(0.04, 0.06)
Utility at 18 months	270	0.76	0.019	(0.72, 0.79)	267	0.75	0.020	(0.71, 0.79)	-0.01	(0.05, 0.07)
Utility at 24 months	270	0.76	0.019	(0.72, 0.79)	267	0.76	0.018	(0.72, 0.79)	0.00	(0.05, 0.04)
**Discounted QALYs**	270	1.51	0.030	(1.45, 1.57)	267	1.51	0.033	(1.44, 1.58)	-0.00	(0.082, 0.080)

QALY = quality-adjusted life year. SD = standard deviation for non-imputed data. SE = standard error for imputed data. CI = confidence interval. Costs are in 2013/14 UK£. CI were based on non-parametric bootstrapping for observed data and imputed data.
* Missing values imputed using multiple imputation with 20 imputed datasets (see text).

For peer review only - http://bmjopen.bmj.com/site/about/guidelines.xhtml

Figure 1. Cost-Effectiveness plane, base case analysis assumptions. Based on 20,000 bootstrap replications, from 20 imputed datasets.

Figure 2. Cost-effectiveness acceptability curve showing the probability that 10TT is cost-effective vs usual care at different values of the maximum willingness to pay for a QALY (base-case assumptions)

QALY = quality adjusted life year

For peer review only

References

1. http://www.euro.who.int/en/health-topics/noncommunicable-diseases/obesity/data-and-statistics.
2. http://ec.europa.eu/eurostat/statistics-explained/index.php/Overweight_and_obesity_-_BMI_statistics.
3. Guh DP, Zhang W, Bansback N, Amarsi Z, Birmingham CL, Anis AH. The incidence of co-morbidities related to obesity and overweight: a systematic review and meta-analysis. BMC public health. 2009;9:88.
4. Reducing obesity and improving diet. https://www.gov.uk/government/policies/reducing-obesity-and-improving-diet. Accessed 07.03.15.
5. Roux L, Kuntz KM, Donaldson C, Goldie SJ. Economic evaluation of weight loss interventions in overweight and obese women. Obesity (Silver Spring, Md). 2006;14(6):1093-106.
6. Fuller NR, Carter H, Schofield D, Hauner H, Jebb SA, Colagiuri S, et al. Cost effectiveness of primary care referral to a commercial provider for weight loss treatment, relative to standard care: a modelled lifetime analysis. Int J Obes. 2014;38(8):1104-9.
7. Fuller NR, Colagiuri S, Schofield D, Olson AD, Shrestha R, Holzapfel C, et al. A within-trial cost-effectiveness analysis of primary care referral to a commercial provider for weight loss treatment, relative to standard care--an international randomised controlled trial. International journal of obesity (2005). 2013;37.
8. Tsai AG, Wadden TA, Volger S, Sarwer DB, Vetter M, Kumanyika S, et al. Cost-effectiveness of a primary care intervention to treat obesity. International journal of obesity (2005). 2013;37 Suppl 1:S31-7.
9. Trueman P, Haynes SM, Felicity Lyons G, Louise McCombie E, McQuigg MS, Mongia S, et al. Long-term cost-effectiveness of weight management in primary care. International journal of clinical practice. 2010;64(6):775-83.
10. Gardner B, Lally P, Wardle J. Making health habitual: the psychology of 'habit-formation' and general practice. The British journal of general practice : the journal of the Royal College of General Practitioners. 2012;62(605):664-6.
11. Lally P, Chipperfield A, Wardle J. Healthy habits: efficacy of simple advice on weight control based on a habit-formation model. Int J Obes. 2007;32(4):700-7.
12. http://www.nhs.uk/Livewell/5ADAY/Pages/Why5ADAY.aspx.
13. National Institute for Health and Care Excellence (NICE). Guide to the methods of technology appraisal. London: NICE; 2013.
14. Curtis L. Unit costs of health and social care 2013. Kent: Personal Social Services Research Unit; 2013.
15. Department of Health. National Schedule of Reference Costs- 2012-2013. NHS trusts and NHS foundation trusts. London Department of Health; 2012.

For peer review only - http://bmjopen.bmj.com/site/about/guidelines.xhtml

16. Joint Formulary Committee . British National Formulary, 68th edition. London: BMJ Group and Pharmaceutical Press; 2013 [Available from: https://www.medicinescomplete.com/mc/bnf/current/.
17. Jolly K, Lewis A, Beach J, Denley J, Adab P, Deeks JJ, et al. Comparison of range of commercial or primary care led weight reduction programmes with minimal intervention control for weight loss in obesity: lighten Up randomised controlled trial. BMJ (Clinical research ed). 2011;343:d6500.
18. Rabin R, de Charro F. EQ-5D: a measure of health status from the EuroQol Group. Annals of medicine. 2001;33(5):337-43.
19. Brooks R. EuroQol: the current state of play. Health policy (Amsterdam, Netherlands). 1996;37(1):53-72.
20. Dolan P. Modeling valuations for EuroQol health states. Medical care. 1997;35(11):1095-108.
21. Petrou S, Dakin H, Abangma G, Benge S, Williamson I. Cost-utility analysis of topical intranasal steroids for otitis media with effusion based on evidence from the GNOME trial. Value in health : the journal of the International Society for Pharmacoeconomics and Outcomes Research. 2010;13(5):543-51.
22. Briggs A, Clark T, Wolstenholme J, Clarke P. Missing... presumed at random: cost-analysis of incomplete data. Health economics. 2003;12(5):377-92.
23. Royston P Multiple imputation of missing values: update of ice. Stata J. 2005: 527-36.
24. StataCorp. 2015. Stata Statistical Software: Release 14. College Station, TX: StataCorp LP. .
25. Sinharay S, Stern HS, Russell D. The use of multiple imputation for the analysis of missing data. Psychological methods. 2001;6(4):317-29.
26. Yang X, Belin TR, Boscardin WJ. Imputation and variable selection in linear regression models with missing covariates. Biometrics. 2005;61(2):498-506.
27. Pasta DJ, Taylor JL, Henning JM. Probabilistic sensitivity analysis incorporating the bootstrap: an example comparing treatments for the eradication of Helicobacter pylori. Medical decision making : an international journal of the Society for Medical Decision Making. 1999;19(3):353-63.
28. Zethraeus N, Johannesson M, Jonsson B, Lothgren M, Tambour M. Advantages of using the net-benefit approach for analysing uncertainty in economic evaluation studies. PharmacoEconomics. 2003;21(1):39-48.
29. Briggs AH, Gray AM. Handling uncertainty when performing economic evaluation of healthcare interventions. Health technology assessment (Winchester, England). 1999;3(2):1-134.
30. Beeken RJ, Leurent B, Vickerstaff V, Wilson R, Croker H, Morris S, et al. A brief intervention for weight control based on habit-formation theory delivered through primary care: results from a randomised controlled trial. International journal of obesity (2005). 2017.
31. http://data.worldbank.org/indicator/pa.nus.pppc.rf.
32. Ahern AL, Wheeler GM, Aveyard P, Boyland EJ, Halford JCG, Mander AP, et al. Extended and standard duration weight-loss programme referrals for adults in primary care (WRAP): a randomised controlled trial. Lancet. 2017;389(10085):2214-25.
33. Kolotkin RL, Norquist JM, Crosby RD, Suryawanshi S, Teixeira PJ, Heymsfield SB, et al. One-year health-related quality of life outcomes in weight loss trial participants: comparison of three measures. Health and quality of life outcomes. 2009;7:53.
34. Peckmezian T, Hay P. A systematic review and narrative synthesis of interventions for uncomplicated obesity: weight loss, well-being and impact on eating disorders. Journal of eating disorders. 2017;5:15.
35. Herrett E, Shah AD, Boggon R, Denaxas S, Smeeth L, van Staa T, et al. Completeness and diagnostic validity of recording acute myocardial infarction events in primary care, hospital care, disease registry, and national mortality records: cohort study. BMJ (Clinical research ed). 2013;346:f2350.

For peer review only - http://bmjopen.bmj.com/site/about/guidelines.xhtml

36. Sterne JA, White IR, Carlin JB, Spratt M, Royston P, Kenward MG, et al. Multiple imputation for missing data in epidemiological and clinical research: potential and pitfalls. BMJ (Clinical research ed). 2009;338:b2393.

37. Lee KJ, Simpson JA. Introduction to multiple imputation for dealing with missing data. Respirology (Carlton, Vic). 2014;19(2):162-7.

38. Hayati Rezvan P, Lee KJ, Simpson JA. The rise of multiple imputation: a review of the reporting and implementation of the method in medical research. BMC medical research methodology. 2015;15:30.

For peer review only

For peer review only - http://bmjopen.bmj.com/site/about/guidelines.xhtml

Figure 1. Cost-Effectiveness plane, base case analysis assumptions. Based on 20,000 bootstrap replications, from 20 imputed datasets.

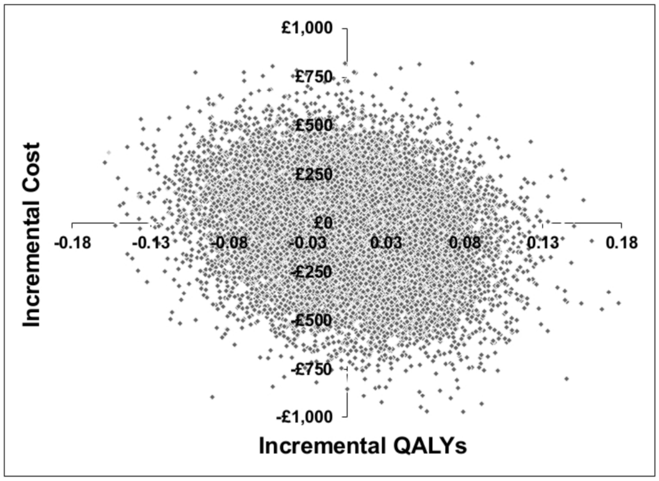

1

Figure 1. Cost-Effectiveness plane, base case analysis assumptions. Based on 20,000 bootstrap replications, from 20 imputed datasets.

296x209mm (300 x 300 DPI)

For peer review only - http://bmjopen.bmj.com/site/about/guidelines.xhtml

Figure 2. Cost-effectiveness acceptability curve showing the probability that 10TT is cost-effective vs usual care at different values of the maximum willingness to pay for a QALY (base-case assumptions)

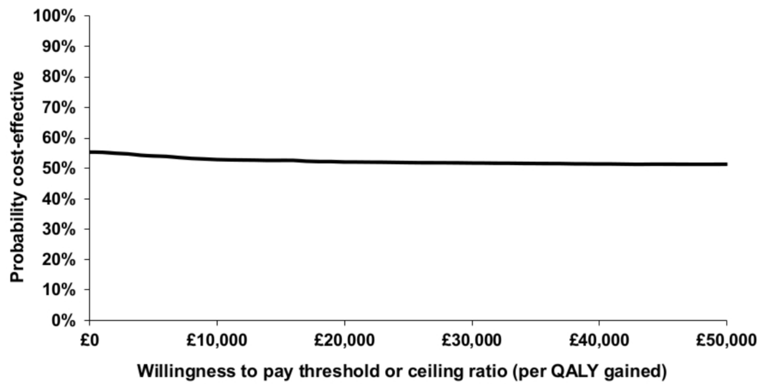

QALY = quality adjusted life year

Figure 2. Cost-effectiveness acceptability curve showing the probability that 10TT is cost-effective vs usual care at different values of the maximum willingness to pay for a QALY (base-case assumptions)

296x209mm (300 x 300 DPI)

BMJ

BMJ 2013;346:f1049 doi: 10.1136/bmj.f1049 (Published 25 March 2013) Page 1 of 6

RESEARCH METHODS & REPORTING

Consolidated Health Economic Evaluation Reporting Standards (CHEERS) statement

OPEN ACCESS

Don Husereau *senior associate; adjunct professor of medicine; senior scientist*[1 2 3], Michael Drummond *co-editor-in-chief, Value in Health; professor of health economics*[4], Stavros Petrou *professor of health economics*[5], Chris Carswell *editor*[6], David Moher *senior scientist*[7], Dan Greenberg *associate professor and chairman; visiting assistant professor* [8 9], Federico Augustovski *director; professor of public health*[10 11], Andrew H Briggs *William R Lindsay chair of health economics, health economics and health technology assessment* [12], Josephine Mauskopf *vice president of health economics* [13], Elizabeth Loder *chief of division; clinical epidemiology editor, BMJ*[14], on behalf of the CHEERS Task Force

[1]Institute of Health Economics, Edmonton, Canada; [2]Department of Epidemiology and Community Medicine, University of Ottawa, Ottawa, Canada; [3]University for Health Sciences, Medical Informatics and Technology, Hall in Tirol, Austria; [4]Centre for Health Economics, University of York, York, UK; [5]Warwick Medical School, University of Warwick, Coventry, UK; [6]Pharmacoeconomics, Adis International, Auckland, New Zealand; [7]Clinical Epidemiology Program, Ottawa Hospital Research Institute, Ottawa, Canada; [8]Department of Health Systems Management, Faculty of Health Sciences, Ben-Gurion University of the Negev, Beer-Sheva, Israel; [9]Center for the Evaluation of Value and Risk in Health, Tufts Medical Center, Boston MA, USA; [10]Health Economic Evaluation and Technology Assessment, Institute for Clinical Effectiveness and Health Policy (IECS), Buenos Aires, Argentina; [11]Universidad de Buenos Aires, Buenos Aires, Argentina; [12]Institute of Health and Wellbeing, University of Glasgow, Glasgow, Scotland; [13]RTI Health Solutions, Research Triangle Park NC, USA; [14]Division of Headache and Pain, Brigham and Women's/Faulkner Neurology, Faulkner Hospital, Boston MA, USA

Abstract

Economic evaluations of health interventions pose a particular challenge for reporting. There is also a need to consolidate and update existing guidelines and promote their use in a user friendly manner. The Consolidated Health Economic Evaluation Reporting Standards (CHEERS) statement is an attempt to consolidate and update previous health economic evaluation guidelines efforts into one current, useful reporting guidance. The primary audiences for the CHEERS statement are researchers reporting economic evaluations and the editors and peer reviewers assessing them for publication.

The need for new reporting guidance was identified by a survey of medical editors. A list of possible items based on a systematic review was created. A two round, modified Delphi panel consisting of representatives from academia, clinical practice, industry, government, and the editorial community was conducted. Out of 44 candidate items, 24 items and accompanying recommendations were developed. The recommendations are contained in a user friendly, 24 item checklist. A copy of the statement, accompanying checklist, and this report can be found on the ISPOR Health Economic Evaluations Publication Guidelines Task Force website (www.ispor.org/TaskForces/EconomicPubGuidelines.asp).

We hope CHEERS will lead to better reporting, and ultimately, better health decisions. To facilitate dissemination and uptake, the CHEERS statement is being co-published across 10 health economics and medical journals. We encourage other journals and groups, to endorse CHEERS. The author team plans to review the checklist for an update in five years.

Health economic evaluations are conducted to inform resource allocation decisions. Economic evaluation has been defined as "the comparative analysis of alternative courses of action in terms of both their costs and their consequences."[1] All economic evaluations assess costs, but approaches to measuring and valuing the consequences of health interventions may differ (see box).

Economic evaluations have been widely applied in health policy, including the assessment of prevention programmes (such as vaccination, screening, and health promotion), diagnostics, treatment interventions (such as drugs and surgical procedures), organisation of care, and rehabilitation. Economic evaluations are increasingly being used for decision making and are an important component of programmes for health technology assessment internationally.[2]

Correspondence to: D Husereau, 879 Winnington Ave, Ottawa, ON K2B 5C4, Canada donh@donhusereau.com

No commercial reuse: See rights and reprints http://www.bmj.com/permissions Subscribe: http://www.bmj.com/subscribe

For peer review only - http://bmjopen.bmj.com/site/about/guidelines.xhtml

BMJ 2013;346:f1049 doi: 10.1136/bmj.f1049 (Published 25 March 2013)

Forms of economic evaluation[1]

Specific forms of analysis reflect different approaches to evaluating the consequences of health interventions. Health consequences may be estimated from a single analytical (experimental or non-experimental) study, a synthesis of studies, mathematical modelling, or a combination of modelling and study information.

Cost consequences analysis examines costs and consequences without attempting to isolate a single consequence or aggregate consequences into a single measure

Cost minimisation analysis (CMA)—The consequences of compared interventions are required to be equivalent, and only relative costs are compared

Cost effectiveness analysis (CEA) measures consequences in natural units, such as life years gained, disability days avoided, or cases detected. In a variant of CEA, often called cost utility analysis, consequences are measured in terms of preference-based measures of health, such as quality adjusted life years or disability adjusted life years.

Cost benefit analysis—Consequences are valued in monetary units.

Readers should be aware that an economic evaluation might be referred to as a "cost effectiveness analysis" or "cost benefit analysis" even if it does not strictly adhere to the definitions above. Multiple forms may also exist within a single evaluation. Different forms of analysis provide unique advantages or disadvantages for decision making. The Consolidated Health Economic Evaluation Reporting Standards (CHEERS) statement can be used with any form of economic evaluation.

Reporting challenges and shortcomings in health economic evaluations

Compared with clinical studies, which report the consequences of an intervention only, economic evaluations require more reporting space for additional items, such as resource use, costs, preference related information, and cost effectiveness results. This creates challenges for editors, reviewers, and those who wish to scrutinise a study's findings.[3] There is evidence that the quality of reporting of economic evaluations varies widely and could potentially benefit from improved quality assurance mechanisms.[4 5]

With the increasing number of publications available, and opportunity costs from decisions based on misleading study findings, transparency and clarity in reporting are important. In addition, outside of economic evaluations conducted alongside clinical trials, there are no widespread mechanisms for warehousing economic evaluation data to allow for independent interrogation, such as ethics review proceedings, regulator dossiers, or study registries. Instead, independent analysis may rely on the record keeping of individual investigators.

Even if measures to promote transparency exist, such as registries, biomedical journal editors have increasingly promoted and endorsed the use of reporting guidelines. Endorsement of guidelines by journals for randomised controlled trials has been shown to improve reporting.[6] The combination of the risk of making costly decisions due to poor reporting with the lack of mechanisms that promote accountability makes transparency in reporting economic evaluations especially important and a primary concern among journal editors and decision makers.[3 7]

Aim and scope

The aim of the Consolidated Health Economic Evaluation Reporting Standards (CHEERS) statement is to provide recommendations, in the form of a checklist, to optimise reporting of health economic evaluations. The need for a contemporary reporting guidance for economic evaluations was recently identified by researchers and biomedical journal editors.[8] The CHEERS statement attempts to consolidate and update previous efforts [9-20] into a single useful reporting guidance.

The primary audiences for the CHEERS statement are researchers reporting economic evaluations and the editors and peer reviewers evaluating their publication potential. We hope the statement (which consists of a 24 item checklist and accompanying recommendations on the minimum amount of information to be included when reporting economic evaluations) is a useful and practical tool for these audiences and will improve reporting and, in turn, health and healthcare decisions. To best understand and apply the recommendations contained within the statement, we encourage readers to access the Explanation and Elaboration Report.[21]

Development of the CHEERS statement

The statement was developed by a task force supported by the International Society for Pharmacoeconomics and Outcomes Research (ISPOR), as part of a broader initiative to facilitate and encourage the interchange of expert knowledge and develop best practices. The CHEERS Task Force members were chosen by the chair of the task force primarily based on their longstanding academic expertise and contribution to the multidisciplinary field of health economic evaluation. In addition to four members of the task force with doctorates in economics and its sub-discipline of health economics (AHB, MD, JM, SP), members included experts in health technology assessment and decision making (FA, AHB, DH, MD, JM) and in clinical epidemiology and biostatistics (AHB, EL, DM), those in active clinical practice (EL, FA), and those with previous experience in reporting guideline development (MD, DM). All members are researchers in applied health and health policy, with five members currently serving as editors for journals in the field (AHB, CC, MD, DG, EL).

The CHEERS Task Force followed current recommendations for developing reporting guidelines.[22] Briefly, the need for new guidance was first identified through a survey of members of the World Association of Medical Editors. Of the 6% (55/965) who responded, 91% (n=50) indicated they would use a standard if one were widely available.[8] Next, published checklists or guidance documents related to reporting economic evaluations were identified from a systematic review and survey of task force members.[23] Both of these activities were used to create a preliminary list of items to include when reporting economic evaluations. Recommendations of the minimum set of reporting items were then developed through a modified Delphi panel process. Forty eight individuals identified by the task force with broad geographical representation and representing academia, biomedical journal editors, the pharmaceutical industry, government decision makers, and those in clinical practice were invited to participate. Thirty eight agreed to participate. Participants were asked to score importance on a Likert scale and the average scores, weighted by each individual's confidence in ability to score, were then used to rank items. A cut-off point was applied to the ranked list to determine the minimum number of items important for reporting.

The CHEERS statement recommendations have been independently reviewed and subsequently revised by task force members. The recommendations are entirely those of the task

For peer review only - http://bmjopen.bmj.com/site/about/guidelines.xhtml

No commercial reuse: See rights and reprints http://www.bmj.com/permissions Subscribe: http://www.bmj.com/subscribe

BMJ 2013;346:f1049 doi: 10.1136/bmj.f1049 (Published 25 March 2013)

force—the sponsors of the study had no role in study design, data analysis, data interpretation, or writing of the final recommendations. A more complete description of the methods and findings of the Delphi panel are found in the larger explanation and elaboration document.[21]

Checklist items

The final recommendations are subdivided into six main categories: (1) title and abstract; (2) introduction; (3) methods; (4) results; (5) discussion; and (6) other. The recommendations are contained in a user friendly, 24 item checklist (table⇓) to aid users who wish to follow them. A copy of the checklist can also be found on the CHEERS Task Force website. (www.ispor.org/TaskForces/EconomicPubGuidelines.asp). In order to encourage dissemination and use of a single international standard for reporting, the task force approached 14 journals identified as either the largest publishers of economic evaluations or widely read by the medical and research community. Thirteen journals responded, and 10 expressed their ability and interest in endorsing this guidance. The CHEERS statement is being simultaneously published in *BMC Medicine, BMJ, BJOG: An International Journal of Obstetrics and Gynaecology, Clinical Therapeutics, Cost Effectiveness and Resource Allocation, The European Journal of Health Economics, International Journal of Technology Assessment in Health Care, Journal of Medical Economics, Pharmacoeconomics,* and *Value in Health.* To facilitate wider dissemination and uptake of this reporting guidance, we encourage other journals and groups to consider endorsing CHEERS.

Concluding remarks

As the number of published health economic evaluations continues to grow, we believe more transparent and complete reporting of methods and findings will be increasingly important to facilitate interpretation and comparison of studies. We hope the CHEERS statement, consisting of recommendations in a 24 item checklist, will be viewed as an effective consolidation and update of previous efforts and serve as a starting point for standard reporting going forward.

We believe the CHEERS statement represents a considerable expansion over previous efforts. The strength of our approach is that it was developed in accordance with current recommendations for the development of reporting guidelines, using an international and multidisciplinary team of editors and content experts in economic evaluation and reporting.[22] Similar to the approach taken with other widely accepted guidelines, we have defined a minimum set of criteria though a modified Delphi technique and have translated these into recommendations, an explanatory document with explanations, and a checklist. Unlike some previous reporting guidance for economic evaluation, we have also made every effort to be neutral about the conduct of economic evaluation, allowing analysts the freedom to choose different methods.

There may be several limitations to our approach. A larger Delphi panel with a different composition could have led to a different final set of recommendations.[24] Some less common approaches and contexts (such as public health, developing countries, and system dynamic models) for conducting health economic evaluation may not be well represented by our sample of experts. Additionally, like many Delphi panel processes, we based decisions to reject or accept criteria on arbitrary levels of importance. However, we feel the group recruited to create the statement is sufficiently knowledgeable of the more common applications of economic evaluation, and the rules used to select criteria were created a priori and are consistent with previous efforts.

We believe it will be important to evaluate the effects of implementation of this statement and checklist on reporting in future economic evaluations. As methods for the conduct of economic evaluation continue to evolve, it will also be important to revisit or extend the guidance. The CHEERS Task Force feels that this statement should be reviewed for updating five years from its release.

Competing interests: All authors have completed the ICMJE uniform disclosure form at www.icmje.org/coi_disclosure.pdf and declare: FA served as board member for the study funder; FA, AHB, CC, MD, DG, DH, EL, JM, and SP were provided support for travel to a face-to-face meeting to discuss the contents of the report; FA and MD have received payment from the study sponsor for serving as co-editors for *Value in Health*; no other relationships or activities that could appear to have influenced the submitted work.

Elizabeth Loder—*BMJ* clinical epidemiology editor—played no part in the peer review or decision making of this paper at the editorial level, and contributed solely as an author.

The International Society for Pharmacoeconomics and Outcomes Research (ISPOR) Health Economic Evaluation Publication Guidelines—CHEERS Task Force acknowledge the support of Elizabeth Molsen; Donna Rindress, who provided the initial leadership for this effort; and the reviewers and Delphi panel participants, who are named in the larger explanation and elaboration document and on the CHEERS Task Force website.

Contributors: All authors provided a substantial contribution to the design and interpretation of the protocol and guidance, as well as writing sections of drafts, revising based on comments received, and approving the final version. DH conducted the analysis of Delphi panel responses, drafted and revised the protocol and the drafts of this paper, and is the guarantor for the study.

Funding: All CHEERS Task Force members are volunteers. Support for this initiative was provided by the International Society for Pharmacoeconomics and Outcomes Research.

1 Drummond MF, Sculpher MJ, Torrance G, O'Brien J, Stoddart GL. *Methods for the economic evaluation of health care programmes* . 3rd ed. Oxford University Press, 2005.
2 Drummond MF, Schwartz JS, Jönsson B, Luce BR, Neumann PJ, Siebert U, et al. Key principles for the improved conduct of health technology assessments for resource allocation decisions. *Int J Technol Assess Health Care* 2008;24:244-58.
3 Rennie D, Luft HS. Pharmacoeconomic analyses. *JAMA* 2000;283:2158-60.
4 Neumann PJ, Stone PW, Chapman RH, Sandberg EA, Bell CM. The quality of reporting in published cost-utility analyses, 1976-1997. *Ann Intern Med* 2000;132:964.
5 Rosen AB, Greenberg D, Stone PW, Olchanski NV, Neumann PJ. Quality of abstracts of papers reporting original cost-effectiveness analyses. *Med Decis Making* 2005;25:424-8.
6 Turner L, Shamseer L, Altman DG, Schulz KF, Moher D. Does use of the CONSORT Statement impact the completeness of reporting of randomised controlled trials published in medical journals? A Cochrane review. *Syst Rev* 2012;1:60.
7 Drummond MF. A reappraisal of economic evaluation of pharmaceuticals. Science or marketing? *Pharmacoeconomics* 1998;14:1-9.
8 McGhan WF, Al M, Doshi JA, Kamae I, Marx SE, Rindress D. The ISPOR Good Practices for Quality Improvement of Cost-Effectiveness Research Task Force report. *Value Health* 2009;12:1086-99.
9 Task Force on Principles for Economic Analysis of Health Care Technology. Economic analysis of health care technology. A report on principles. *Ann Intern Med* 1995;123:61-70.
10 Drummond MF, Jefferson TO. Guidelines for authors and peer reviewers of economic submissions to the BMJ. *BMJ* 1996;313:275-83.
11 Gold MR. *Cost-effectiveness in health and medicine* . Oxford University Press, 1996.
12 Siegel JE, Weinstein MC, Russell LB, Gold MR. Recommendations for reporting cost-effectiveness analyses. Panel on Cost-Effectiveness in Health and Medicine. *JAMA* 1996;276:1339-41.
13 Nuijten MJ, Pronk MH, Brorens MJ, Hekster YA, Lockefeer JH, de Smet PA, et al. Reporting format for economic evaluation: Part II: Focus on modelling studies. *Pharmacoeconomics* 1998;14:259-68.
14 Vintzileos AM, Beazoglou T. Design, execution, interpretation, and reporting of economic evaluation studies in obstetrics. *Am J Obstet Gynecol* 2004;191:1070-6.
15 Drummond M, Manca A, Sculpher M. Increasing the generalizability of economic evaluations: recommendations for the design, analysis, and reporting of studies. *Int J Technol Assess Health Care* 2005;21:165-71.

No commercial reuse: See rights and reprints http://www.bmj.com/permissions

Subscribe: http://www.bmj.com/subscribe

For peer review only - http://bmjopen.bmj.com/site/about/guidelines.xhtml

BMJ 2013;346:f1049 doi: 10.1136/bmj.f1049 (Published 25 March 2013)

16 Ramsey S, Willke R, Briggs A, Brown R, Buxton M, Chawla A, et al. Good research practices for cost-effectiveness analysis alongside clinical trials: the ISPOR RCT-CEA Task Force report. *Value Health* 2005;8:521-33.

17 Goetghebeur MM, Wagner M, Khoury H, Levitt RJ, Erickson LJ, Rindress D. Evidence and value: impact on decisionmaking—the EVIDEM framework and potential applications. *BMC Health Serv Rev* 2008;8:270.

18 Davis JC, Robertson MC, Comans T, Scuffham PA. Guidelines for conducting and reporting economic evaluation of fall prevention strategies. *Osteoporos Int* 2010;22:2449-59.

19 Petrou S, Gray A. Economic evaluation using decision analytical modelling: design, conduct, analysis, and reporting. *BMJ* 2011;342:d1766.

20 Petrou S, Gray A. Economic evaluation alongside randomised controlled trials: design, conduct, analysis, and reporting. *BMJ* 2011;342:d1548.

21 Husereau D, Drummond M, Petrou S, Carswell C, Moher D, Greenberg D, et al. Consolidated Health Economic Evaluation Reporting Standards (CHEERS)—explanation and elaboration: a report of the ISPOR Health Economic Evaluations Publication Guidelines Task Force. *Value in Health* (forthcoming).

22 Moher D, Schulz KF, Simera I, Altman DG. Guidance for developers of health research reporting guidelines. *PLoS Med* 2010;7:e1000217.

23 Moher D, Simera I, Schulz K, Miller D, Grimshaw J, Hoey J, et al. Reporting guidelines for clinical research: a systematic review. Vancouver, BC, 2009. Available at: www.ama-assn.org/public/peer/abstracts-0912.pdf.

24 Campbell SM, Hann M, Roland MO, Quayle JA, Shekelle PG. The effect of panel membership and feedback on ratings in a two-round Delphi survey: results of a randomized controlled trial. *Med Care* 1999;37:964-8

Cite this as: *BMJ* 2013;346:f1049

This is an open-access article distributed under the terms of the Creative Commons Attribution Non-commercial License, which permits use, distribution, and reproduction in any medium, provided the original work is properly cited, the use is non commercial and is otherwise in compliance with the license. See: http://creativecommons.org/licenses/by-nc/2.0/ and http://creativecommons.org/licenses/by-nc/2.0/legalcode.

No commercial reuse: See rights and reprints http://www.bmj.com/permissions Subscribe: http://www.bmj.com/subscribe

BMJ 2013;346:f1049 doi: 10.1136/bmj.f1049 (Published 25 March 2013)

Table

Table 1| **CHEERS checklist—Items to include when reporting economic evaluations of health interventions**

Section/item	Item No	Recommendation	Reported on page No/ line No
**Title and abstract**			
Title	1	Identify the study as an economic evaluation or use more specific terms such as "cost-effectiveness analysis", and describe the interventions compared.	1
Abstract	2	Provide a structured summary of objectives, perspective, setting, methods (including study design and inputs), results (including base case and uncertainty analyses), and conclusions.	2
**Introduction**			4-5
Background and objectives	3	Provide an explicit statement of the broader context for the study.	5-6
		Present the study question and its relevance for health policy or practice decisions.	6
**Methods**			
Target population and subgroups	4	Describe characteristics of the base case population and subgroups analysed, including why they were chosen.	5
Setting and location	5	State relevant aspects of the system(s) in which the decision(s) need(s) to be made.	5-6
Study perspective	6	Describe the perspective of the study and relate this to the costs being evaluated.	5
Comparators	7	Describe the interventions or strategies being compared and state why they were chosen.	5
Time horizon	8	State the time horizon(s) over which costs and consequences are being evaluated and say why appropriate.	5
Discount rate	9	Report the choice of discount rate(s) used for costs and outcomes and say why appropriate.	6
Choice of health outcomes	10	Describe what outcomes were used as the measure(s) of benefit in the evaluation and their relevance for the type of analysis performed.	6
Measurement of effectiveness	11a	*Single study-based estimates:* Describe fully the design features of the single effectiveness study and why the single study was a sufficient source of clinical effectiveness data.	N/A
	11b	*Synthesis-based estimates:* Describe fully the methods used for identification of included studies and synthesis of clinical effectiveness data.	N/A
Measurement and valuation of preference based outcomes	12	If applicable, describe the population and methods used to elicit preferences for outcomes.	8
Estimating resources and costs	13a	*Single study-based economic evaluation:* Describe approaches used to estimate resource use associated with the alternative interventions. Describe primary or secondary research methods for valuing each resource item in terms of its unit cost. Describe any adjustments made to approximate to opportunity costs.	6-7
	13b	*Model-based economic evaluation:* Describe approaches and data sources used to estimate resource use associated with model health states. Describe primary or secondary research methods for valuing each resource item in terms of its unit cost. Describe any adjustments made to approximate to opportunity costs.	N/A.
Currency, price date, and conversion	14	Report the dates of the estimated resource quantities and unit costs. Describe methods for adjusting estimated unit costs to the year of reported costs if necessary. Describe methods for converting costs into a common currency base and the exchange rate.	6
Choice of model	15	Describe and give reasons for the specific type of decision-analytical model used. Providing a figure to show model structure is strongly recommended.	6
Assumptions	16	Describe all structural or other assumptions underpinning the decision-analytical model.	N/A
Analytical methods	17	Describe all analytical methods supporting the evaluation. This could include methods for dealing with skewed, missing, or censored data; extrapolation methods; methods for pooling data; approaches to validate or make adjustments (such as half cycle corrections) to a model; and methods for handling population heterogeneity and uncertainty.	9-10
**Results**			
Study parameters	18	Report the values, ranges, references, and, if used, probability distributions for all parameters. Report reasons or sources for distributions used to represent uncertainty where appropriate. Providing a table to show the input values is strongly recommended.	N/A.
Incremental costs and outcomes	19	For each intervention, report mean values for the main categories of estimated costs and outcomes of interest, as well as mean differences between the comparator groups. If applicable, report incremental cost-effectiveness ratios.	11-12. 27
Characterising uncertainty	20a	*Single study-based economic evaluation:* Describe the effects of sampling uncertainty for the estimated incremental cost and incremental effectiveness parameters, together with the impact of methodological assumptions (such as discount rate, study perspective).	13, 27

No commercial reuse: See rights and reprints http://www.bmj.com/permissions Subscribe: http://www.bmj.com/subscribe

For peer review only - http://bmjopen.bmj.com/site/about/guidelines.xhtml

BMJ 2013;346:f1049 doi: 10.1136/bmj.f1049 (Published 25 March 2013) Page 6 of 6

(continued)

Section/Item	Item No	Recommendation	Reported on page No/ line No
	20b	*Model-based economic evaluation:* Describe the effects on the results of uncertainty for all input parameters, and uncertainty related to the structure of the model and assumptions.	N/A
Characterising heterogeneity	21	If applicable, report differences in costs, outcomes, or cost-effectiveness that can be explained by variations between subgroups of patients with different baseline characteristics or other observed variability in effects that are not reducible by more information.	N/A
**Discussion**			
Study findings, limitations, generalisability, and current knowledge	22	Summarise key study findings and describe how they support the conclusions reached. Discuss limitations and the generalisability of the findings and how the findings fit with current knowledge.	14-16.
**Other**			
Source of funding	23	Describe how the study was funded and the role of the funder in the identification, design, conduct, and reporting of the analysis. Describe other non-monetary sources of support.	19-20
Conflicts of interest	24	Describe any potential for conflict of interest of study contributors in accordance with journal policy. In the absence of a journal policy, we recommend authors comply with International Committee of Medical Journal Editors recommendations.	19-20

For consistency, the CHEERS statement checklist format is based on the format of the CONSORT statement checklist

No commercial reuse: See rights and reprints http://www.bmj.com/permissions Subscribe: http://www.bmj.com/subscribe

Cost-effectiveness of habit-based advice for weight control versus usual care in general practice in the Ten Top Tips (10TT) Trial: economic evaluation based on a randomised controlled trial

Journal:	*BMJ Open*
Manuscript ID	bmjopen-2017-017511.R3
Article Type:	Research
Date Submitted by the Author:	23-Mar-2018
Complete List of Authors:	Patel, Nishma; UCL, Department of Applied Health Research<br>Beeken, Rebecca; University of Leeds, Leeds Institute of Health Sciences Clarendon Way LS2 9NL<br>Leurent, Baptiste; London School of Hygiene and Tropical Medicine, Department of Infectious Disease Epidemiology<br>Omar, Rumana; University College London,<br>Nazareth, Irwin; UCL, Primary Care and Population Health<br>Morris, Stephen; University College London, Department of Applied Health Research
**Primary Subject Heading**:	Health economics
Secondary Subject Heading:	Public health, General practice / Family practice
Keywords:	Weight Loss programme, PRIMARY CARE, Cost-effectiveness

SCHOLARONE™
Manuscripts

Cost-effectiveness of habit-based advice for weight control versus usual care in general practice in the Ten Top Tips (10TT) Trial: economic evaluation based on a randomised controlled trial

Nishma Patel[1], Rebecca J. Beeken[2,3], Baptiste Leurent[4], Rumana Z. Omar[5], Irwin Nazareth[6] and Stephen Morris[1]

[1] Department of Applied Health Research, University College London, Gower Street, London, WC1E 6BT, UK

[2] Department of Behavioural Science & Health, University College London, Gower Street, London, WC1E 6BT, UK

[3]Leeds Institute of Health Sciences, University of Leeds, Clarendon Way, Leeds, LS2 9NL

[4] London School of Hygiene and Tropical Medicine, Keppel Street, WC1E 7HT

[5] Department of Statistical Science, University College London, Gower Street, London, WC1E 6BT, UK

[6]Deparment of Primary Care & Population Health, UCL, Gower Street, London, WC1E 6BT

Correspondence to: Nishma Patel, Department of Applied Health Research, University College London WC1E 6BT, UK. Telephone: +44 2031 083027 (email: nishma.patel@ucl.ac.uk)

For peer review only - http://bmjopen.bmj.com/site/about/guidelines.xhtml

Abstract

Objective: Ten Top Tips (10TT) is a primary care led behavioural intervention, which aims to help adults reduce and manage their weight by following ten weight loss tips. The intervention promotes habit formation to encourage long-term behavioural changes. The aim of this study was to estimate the cost-effectiveness of 10TT in general practice from the perspective of the UK National Health Service.

Design: An economic evaluation was conducted alongside an individually randomised controlled trial.

Setting: 14 General Practitioner (GP) practices in England.

Participants: All patients were aged ≥18 years, with BMI ≥30kg/m^2. A total of 537 patients were recruited; 270 received the usual care offered by their practices and 267 received the 10TT intervention.

Outcomes measures: Health service use and quality-adjusted life years (QALY) were measured over two years. Analysis was conducted in terms of incremental net monetary benefits (NMBs), using non-parametric bootstrapping and multiple imputation.

Results: Over a two-year time horizon, mean costs and QALYs per patient in the 10TT group were £1889 (95% CI: £1522 to £2566) and 1.51 (95% CI: 1.44 to 1.58). The mean costs and QALYs for usual care were £1925 (95% CI: £1599 to £2251) and 1.51 (95% CI: 1.45 to 1.57), respectively. This generated a mean cost difference of -£36 (95%CI: -£512 to £441) and a mean QALY difference of 0.001 (95%CI: -0.080 to 0.082). The incremental NMB for 10TT versus usual care was £49 (95%CI: -£1709 to £1800) at a maximum willingness to pay for a QALY of £20,000. 10TT had a 52% probability of being cost-effective at this threshold.

For peer review only

For peer review only - http://bmjopen.bmj.com/site/about/guidelines.xhtml

Conclusions: Costs and QALYs for 10TT were not significantly different from usual care and therefore 10TT is as cost-effective as usual care. There was no evidence to recommend nor advice against offering 10TT to obese patients in general practices based on cost-effectiveness considerations.

Trial Registration: ISRCTN registry ISRCTN16347068

Article summary

Strengths and limitations of this study

- The analysis is based on a large multicentre randomised trial with detailed information on resource use and utility values for a median follow-up period of two years.
- Individual data on standard weight-loss interventions received by the participant were not recorded.
- The analysis took a UK NHS/PSS perspective but a wider perspective (e.g., societal) could have been taken, including costs to patients and families.
- The time horizon was two years
- Missing NHS resource use and utility data was accounted for using multiple imputation.

For peer review only - http://bmjopen.bmj.com/site/about/guidelines.xhtml

Introduction

The worldwide prevalence of obesity nearly doubled between 1980 and 2008. Based on recent estimates in the European Union, overweight affects 30-70% and obesity affects 10-30% of adults(1). In the UK 23.9 % of women aged 18 years and over are obese- the highest proportion in Europe. The proportion of UK men who are obese is 22.1%, the second highest proportion in Europe behind Malta (24.7 %)(2).

Health risks associated with obesity range from heart disease, stroke and type 2 diabetes to all cancers, gall bladder disease and mortality(3). It is estimated that health problems associated with being overweight or obese cost the National Health Service (NHS) in England more than £5 billion every year(4).

Non-surgical interventions for weight loss are available and include dietary advice, physical activity and behaviour modification. These can be accessed mainly through primary care. Despite considerable investment in such interventions, there is limited evidence about their cost-effectiveness(5-9).

Ten Top Tips (10TT) was a simple leaflet-based intervention for patients in primary care designed to help with weight loss. The underpinning theory behind 10TT was habit formation. Psychological research shows that repetition of a simple action in a consistent context leads, through associative learning, to the action being activated upon subsequent exposure to those contextual cues(10). A small pilot trial consisting of 100 people showed that those who received the Ten Top Tips leaflet lost significantly more weight than those who did not receive it(11). Although the absolute weight loss was modest, the intervention had the potential to be disseminated at minimal cost to large numbers simply by making the

For peer review only - http://bmjopen.bmj.com/site/about/guidelines.xhtml

leaflet available. In response to this a large randomised trial was undertaken. Participants were given a leaflet, which provided weight loss tips based on scientific evidence on how to turn the tips into healthy habits, during a 30-minute appointment with a practice nurse. The ten healthy tips included: keeping to meal routines; reduced fat intake; walking off weight; packing a healthy snack; looking at food labels; taking caution with portion size; standing up; thinking about drinks; focusing on food; and, not forgetting 'five a day'. The 'five a day campaign is based on recommendation from the World Health Organisation (WHO) encouraging consumption of at least 400g of fruit and vegetables a day to lower the risk of serious health problems, such as heart disease, stroke and some cancers(12). Participants were asked to record their progress in a logbook. Using routinely collected data from primary care practices, the aim of the present study was to analyse the cost and cost-effectiveness of 10TT.

Methods

Trial background

The 10TT trial was a two-arm, individually randomised, controlled trial in which 537 obese men and women were enrolled(13). Practices across England (n=14) were recruited through the MRC General Practice Research Framework (GPRF). They were located in: Wellingborough, Southampton, Bradford-on-Avon, Bromsgrove, Frome, Guisborough, Glastonbury, Ivybridge, Dunstable, Liskeard, Ledbury, New Mills, and London. The majority were located in Southern England (n=9), with three located in the Midlands and two in the North. Recruitment occurred between August 2010 and October 2011. Participants were randomly assigned to 10TT (n=267) or usual care (n=270), and followed for up to two years. The primary outcome was weight loss at three months. Secondary outcomes included BMI,

For peer review only - http://bmjopen.bmj.com/site/about/guidelines.xhtml

waist circumference, the number of people achieving a 5% reduction in weight, clinical markers for potential co-morbidities (blood pressure, total cholesterol/low-density lipoprotein and blood glucose), and maintenance of weight loss over 24 months. At three months participants in the 10TT group lost significantly more weight than those receiving usual care (mean difference in weight change=-0·87kg, 95% CI -1·47 to -0·27, p=0·004). But this effect was not maintained at 24 months (mean difference in weight change= +0·75kg (95% CI: -0.73 to 2.24)). Weight loss in the usual care group was slow in the first 6 months, but it continued until 18 months, whereas the 10TT group experienced a greater weight loss in the first 6 months, but did not lose any additional weight after this point.

Patient and Public Involvement

There was patient representation on the trial steering group for 10TT, however neither patients nor the public were involved in the economic analyses presented in this paper.

Overview of economic evaluation

We undertook a cost-utility analysis to compare the costs and outcomes associated with 10TT and usual care. The outcome measure was quality-adjusted life years (QALYs), which combines length of life and quality of life, and is consistent with National Institute for Health and Care Excellence (NICE) recommendations(14).The primary outcomes were incremental costs and effects and the incremental net monetary benefit (NMB) of 10TT versus usual care. Costs were measured from a NHS and personal social services (PSS) perspective; since PSS costs were negligible, we focused on NHS costs. These were calculated in 2013/14 UK£. The time horizon for costs and effects was two years, reflecting the trial follow-up period. Extrapolation beyond this time period was not undertaken because the within-trial analysis

For peer review only - http://bmjopen.bmj.com/site/about/guidelines.xhtml

found no evidence of a significant difference in costs or outcomes at 24 months; two years was therefore considered long enough to reflect all important differences in costs or outcomes between treatments. Costs and outcomes in the second year were discounted at 3.5%(14).

Resource use and costs

Resource use data were extracted from GP records for two years prior to randomisation and two-years post randomisation on: the number and type of contacts with the GP (surgery visit, home visit, phone call); practice nurse contacts (practice visit, home visit, phone call); dietician visits; hospital outpatient visits; hospital inpatient stay; accident and emergency (A&E) visits; and outpatient services. Data on use of drugs included dose, type and frequency of administration. All resource use data were extracted by practice nurses at participating centres from patient records.

Unit costs were attached to each resource item. The cost per visit to primary care (GP surgery visit, GP home visit, GP phone call, practice nurse visit, nurse home visit, practice nurse phone call and dietician visits) were taken from Personal Social Services Research Unit (PSSRU) estimates(15). The cost of inpatient episodes, outpatient visits and A&E visits were taken from the PSSRU and based on NHS Reference Costs(16). They included cost of medical staff, equipment, consumables, and diagnostics. The weighted average cost of inpatient stays was calculated combining the cost of inpatient long and short stay. Drug costs were obtained from the British National Formulary(17).

The cost of the 10TT intervention included the following, valued using market prices: a logbook (£2.80 per participant); time patients spent with the practice nurse to introduce the

For peer review only - http://bmjopen.bmj.com/site/about/guidelines.xhtml

programme (£20 for a 30-minute visit); wallet sized food label guidance (£0.09), and a 10TT leaflet (£0.05). The total cost of 10TT was £22.94 per participant. The cost of the food label guidance was based on the total cost of printing (£1,392) divided by the number of labels printed (15,000). Similarly, the cost of 10TT leaflets was based on a total cost of printing (£1,540) divided by the number of leaflets printed (30,000).

All practices offered standard weight-loss interventions as part of usual care. These consisted of a range of interventions including referrals to community programmes, gym prescription and referral to a dietician and/or psychologist, among others. While data were provided by practices on interventions typically offered to their patients, the uptake of these interventions was not recorded. In our base case, we made the conservative assumption (possibly biased against 10TT) that no patients received these interventions. In a sensitivity analysis, we included the costs of these interventions assuming each patient in the control group received the standard weight-loss intervention offered by the practice. Where single interventions were offered by a practice these were costed accordingly, based on the average cost of participating in Weight Watchers (assumed to cost £78.22 per patient(18)) or Slimming World (£72.62 per patient). Where multiple interventions were offered by a practice these were costed based on the average cost of participating in the Size Down Programme at a cost of £93.48 per patient(18).

Unit costs (total expenditure incurred by the NHS for one visit) for each cost component are shown in Table 1. These were multiplied by resource volume and summed across all cost components to obtain a total cost per patient.

Utilities and QALYs

For peer review only - http://bmjopen.bmj.com/site/about/guidelines.xhtml

Health utilities were based on the EuroQol EQ-5D-3L descriptive system (19, 20). This is a five dimensional questionnaire (mobility, self-care, usual activities, pain and discomfort), with three levels in each dimension (severe problems, some problems, no problems). Each EQ-5D state was converted into a single utility score based on valuations from the UK general population(21). Utility values of one represent full health, values of zero are equivalent to death, negative values represent states worse than death. The EQ-5D questionnaire was completed at baseline, three months, six months, 12 months, 18 months and 24 months. A utility profile was constructed for participants assuming a straight-line relation between their utility values at each measurement point. QALYs for every patient from baseline to two years were calculated as the area under the utility profile.

Dealing with Missing Data

There were missing data for NHS resource use and utility scores. Multivariate imputation by chained equations was used to impute missing data (22, 23). The imputation method used an iterative Markov Chain Monte Carlo (MCMC) technique to simulate from the posterior predictive distribution of missing data (24). We generated 20 imputed datasets. We imputed missing data (% of missing data) for the following variables: weight (0.2%); BMI (0.2%); waist circumference (0.6%); EQ-5D at baseline (5%), 3 months (26%), 6 months (40%), 12 months (46%) and 24 months (46%); NHS visits (GP practice (28%); GP home (31%); GP phone calls (30%); practice nurse (27%); nurse home (31%); nurse phone calls (31%); extra nurse (30%); dietician (31%); hospital inpatient stay (31%); outpatient clinic (30%); A&E visits (30%)) and other outpatient service visits (30%) (Table 1). Age, sex and study centre were included in the imputation model as additional explanatory variables. Imputations were undertaken

For peer review only - http://bmjopen.bmj.com/site/about/guidelines.xhtml

For peer review only

using the –mi impute mvn– command in Stata SE version 14 (Stata Corp, College Station, TX)(25).

Statistical analysis

Two sample t-tests to test for differences in cost and QALYs between the two groups were carried out for complete data. A linear regression model was used to test for differences in mean resource use, costs, EQ-5D scores at each time point, and QALYs (26, 27) using the imputed data. The incremental NMBs were calculated as the difference in mean QALYs per participant (*Q*) with 10TT(*T*) versus usual care(*U*) multiplied by the maximum willingness to pay for a QALY (*R*) minus the difference in mean cost per participant (*C*), i.e., incremental NMB = $(Q_T - Q_U)*R - (C_T - C_U)$. We used the cost-effectiveness threshold range recommended by NICE (£20 000 to £30 000 (14)) as the lower and upper limits of the maximum willingness to pay for a QALY (*R*). Negative incremental NMBs indicate that usual care is preferred on cost-effectiveness grounds and positive incremental NMBs indicate that 10TT is preferred (28).

Incremental costs were initially adjusted for; age, gender, practice and costs two years prior to implementation of 10TT. Adjusting for these factors did not make a significant difference to the results. Therefore, these were not included in the final model.

A linear regression model using ordinary least squares (OLS) regression was used in the final analyses to estimate the mean difference in observed QALYs between the two trial arms. The independent variables used to perform the analysis were age, gender, practice and baseline utility values. There were no differences between the two groups in terms of age,

For peer review only - http://bmjopen.bmj.com/site/about/guidelines.xhtml

gender, prior costs and baseline EQ-5D scores (P= 0.47, 0.65, 0.29 and 0.41) (Table 2). Therefore, these were not included in the final model.

For each of the 20 imputed datasets we ran 1000 bootstrap replications using non-parametric bootstrapping, resampling observations with replacement(29). The bootstrapped results were combined using the formula described by Briggs et al (23), to calculate the mean values for costs and utilities and the standard errors around the imputed values. Standard errors were on based a normal distribution and used to calculate 95% CIs around point estimates.

The cost-effectiveness plane was used to illustrate the difference in costs and outcomes between the two groups. The probability that 10TT was cost-effective in comparison to usual care at different values of the cost-effectiveness threshold was presented in form of a cost-effectiveness acceptability curve (CEAC)(30). The CEAC was based on the proportion of bootstrap replications across all twenty imputed datasets that were below the cost-effectiveness threshold, which was varied from £0 to £50,000.

Several deterministic sensitivity analyses were carried out to assess the uncertainty around key components of the analysis. An analysis was undertaken based on complete cases without any imputation. We varied cost components for primary and secondary care costs and drug costs. The total costs of each component were amended by ±10%. We also conducted an analysis including the cost of standard weight-loss interventions for the usual care group.

Results

For peer review only - http://bmjopen.bmj.com/site/about/guidelines.xhtml

Baseline description

Table 3 illustrates the mean costs, two years prior to the trial. The mean cost for the usual care group was £1848 (95% CI: 1615, 2082), and £2052 (95% CI: 1756, 2349) in the 10TT group. Baseline characteristics- age, gender, weight, BMI, blood pressure and cholesterol did not differ between groups(13).

Base case

Table 1 shows mean imputed resource use by treatment group. The intervention and control groups had the same mean GP practice visits per participant over the two-year period (9.4 versus 9.4), as well as similar GP home visits (0.0 versus 0.1), GP phone contacts (1.3 versus 1.3), nurse practice visits (6.6 versus 6.9), nurse home visits (0.0 versus 0.1), inpatient episodes (0.2 versus 0.2), outpatient clinic visits (2.0 versus 2.1) and accident and emergency visits (0.4 versus 0.4). There was no statistically significant difference between groups for any category of health service resource use.

Table 3 shows the total cost per participant in each group. The non-parametric bootstrapping including imputed resource use produced mean total costs per participant in the intervention group of £1889 (95% CI: £1522 to £2566), compared to £1925 (95% CI: £1599 to £2251) in the control group. The mean difference in cost between 10TT and control group was -£36 (95%CI: -£512 to £441), which was not statistically significant (p=0.88).

Undertaking non-parametric bootstrapping after multiple imputation produced 1.51 (95% CI: 1.44 to 1.58) QALYs in the intervention group and 1.51 (95% CI: 1.45 to 1.57) in the

For peer review only - http://bmjopen.bmj.com/site/about/guidelines.xhtml

control group. Generating a mean difference in QALYs of 0.001 (95%CI: -0.080 to 0.082) (p=0.93) (Table 4 and 5) which was not statistically significant. Hence, patients receiving 10TT accrued non-significantly lower costs and the difference in QALYs between the groups was very small and non-significant.

The incremental net monetary benefit for 10TT versus usual care was £49 (95% CI: -£1709 to £1800) at a maximum willingness to pay for a QALY of £20,000 and £55 (95% CI: -£2489 to £2583) at a maximum willingness to pay for a QALY of £30,000 (Table 4).

Of the 20,000 bootstrap replications produced under base case assumptions, 24% fell into south-west quadrant of the cost-effectiveness plane (10TT was less costly and less effective than usual care); 20% fell into the north-east quadrant (10TT was more costly and more effective than usual care); 25% fell into the north-west quadrant (10TT was more costly and less effective than usual care); and 31% fell into the south-east quadrant (10TT was less costly and more effective than usual care) (Figure 1). The CEAC derived from Figure 1 is illustrated in Figure 2. Under base case assumptions, 10TT has a 52% probability of being cost-effective at a willingness to pay threshold of £20,000 and 52% at a willingness to pay threshold of £30,000 (Table 4).

Sensitivity analyses

Including the cost of the standard weight-loss interventions (£87) in the usual care group the mean costs per patient in the control group increased to £2012, which was higher than the mean cost in the intervention group (£1889). The mean incremental cost difference between groups was -£122 (95% CI: -£598 to £353). The incremental net monetary benefit for 10TT versus usual care was £140 (95% CI: (-£1666 to £1902) at a maximum willingness to pay for a QALY of £20,000 and £148 (95% CI: -£2463 to £2693) at a maximum willingness to

For peer review only - http://bmjopen.bmj.com/site/about/guidelines.xhtml

For peer review only

pay of £30,000. Increasing and decreasing costs by 10% did not affect the findings appreciably (Table 4).

A complete case analysis was undertaken using 163 of 537 participants with complete utility and cost data. The incremental NMB was -£889 (95% CI: -£3993 to £2253) at a maximum willingness to pay for a QALY of £20,000 and -£1361 (95% CI: -£5772 to £3052) at a maximum willingness to pay for a QALY of £30,000.

Discussion

Our economic analysis of 10TT showed that this intervention had similar costs and QALYs as usual care. Sensitivity analyses showed little uncertainty in this finding. On the one hand the findings mean there is no reason to prefer 10TT or usual care on the basis of differences in quality of life or cost, or on cost-effectiveness grounds. On the other hand, this means that 10TT is as cost-effective as usual care.

We undertook a rapid review to compare our results to similar weight loss programs. We found that that commercial weight loss programmes were highly prescribed amongst primary care providers and these participants lost more weight than those in self-led education programmes alone. Fuller et al(7) reported the long-term analysis of a 20 minute GP consultation versus Weight Watchers and found Weight Watchers produced a cost saving of US$47 per patient and an incremental 0.03 QALY gained per patient. Similarly, a recent evaluation in the UK of a primary care led behavioural programme(31) looked at a brief advice and self-help materials (primary care led programme) versus Weight Watchers over 12 weeks and over 52 weeks. The authors concluded that Weight Watchers was more effective over 12 weeks (-4.75kg) and 52 weeks (-6.76kg) than brief advice and self-help

For peer review only - http://bmjopen.bmj.com/site/about/guidelines.xhtml

material (-3.26kg), at a cost of £159 per kg lost. Additionally, a primary care led program Counterweight (a nurse-delivered patient education programme) showed that nurse delivered education was less costly and more effective compared with no active intervention, producing a gain in QALYs (0.06 per participant) and cost savings of £27 per participant (9).

It is evident from existing literature that GPs play a crucial role in obesity prevention and weight management and are gate keepers to lifestyle weight management programmes(32). While there is evidence to suggest GP prescribed commercial programmes and/or weight-loss education is effective, further research is needed to explore the relationship between habit-formation programmes such as 10TT with commercial programmes, with the aim to determine what the long-term cost savings and QALYs produced potentially would be over a long-time horizon.

The main strength of our analysis is that it is based on a large multicentre randomised trial with detailed information on resource use and utility values for a median follow-up period of two years. There were several limitations to our study. While data were recorded in the trial on standard weight-loss interventions for obese patients at each practice, individual data on the uptake and prescribing of standard weight-loss interventions received by the participant were not recorded. This trial was also unblinded, which introduced the potential bias of GPs over prescribing standard weight-loss interventions producing a healthy user bias effect. Under base case assumptions, excluding standard weight-loss interventions, the incremental NMB of 10TT versus usual care was not significantly different from zero, and this did not change when intervention costs in the control group were included. Hence, these costs did not make an appreciable difference to our conclusions. Secondly, the

For peer review only

For peer review only - http://bmjopen.bmj.com/site/about/guidelines.xhtml

analysis took a UK NHS/PSS perspective. A wider perspective (e.g., societal) could have been taken, including costs to patients and families. Third, the time horizon was limited to two years, due to the lack of difference in costs (-£36 (95%CI -£512 to £441, p-value 0.88) and benefits (0.001 (95% CI -0.080 to 0.082, p-value 0.93) at 24 months between groups. The trial did not capture QALYs from weight loss post 24 months. A longer follow-up period may have allowed for the detection of long-term health benefits produced as a consequence of avoiding obesity related health conditions, such as diabetes and cardiovascular disease. However, at the time of this study little was known about the association between habit-formation and weight loss. This study has identified the importance of longer-term strategies for continued adherence of weight loss. With add-on approaches such as counselling and education on how to maintain weight loss for participants alongside 10TT, it may be possible to maintain weight loss post two-years.

Fourth, while there are various instruments available to measure health related quality of life, we administered the EQ-5D(33). We acknowledge that there may have been a potential value in using more than one measure in the trial, such as the SF-36 and Impact of Weight on Quality of Life –Lite (IWQOL-Lite) to measure differences in the positive short-term psychological effects(34). However, given the small difference between the two groups it is highly unlikely alternative measures would've produced significant differences. Finally, data for secondary care resource use were extracted from GP practices with a degree of missingness in our analysis. We were unable to access, e.g., Hospital Episode Statistics (HES) data containing detailed secondary care resource use of NHS services by patients. Obtaining HES data would been problematic due to the short timeframe of this trial and linking primary care data to HES data by patient ID(35). We accounted for missing secondary care

For peer review only

For peer review only - http://bmjopen.bmj.com/site/about/guidelines.xhtml

data using multiple imputation, assuming these data to be missing at random. It is important to note that when using such methods there is uncertainty around the non-observed value across the imputations. To account for the uncertainty around the values, we employed the non-parametric bootstrap approach to estimate the variance (a representation of uncertainty) around the true values(23). We acknowledge that though multiple imputation is able to statistically test for error, this method can produce bias. The bias arises from the assumption that missing data in the study were 'missing at random'. For example, the missing at random assumption may be reasonable if a variable that is predictive of missing data is included in the imputation model, but not if the variable is omitted from the model(36). However, multiple imputation offers an advantage over complete case analysis, by filling in missing values and enabling participants to be included in the analysis, where there were auxiliary variables that are strong predictors of missingness(37). Guidelines were followed to ensure the model predicted the best possible estimates; investigation of the proportion of missing data; selection of specific variables and; identification of the predictors of missingness(38). We acknowledge that as the percentage of missingness increases (in some cases more than 50%) there will be greater inefficiency in the imputed data and more chance of bias. However, in general the application of regression imputation with large missing data may be acceptable if adjustments are made for the predictions, as we have done(23).

In conclusion, the 10TT trial found that after three months' participants receiving 10TT lost significantly more weight than those receiving usual care, but there was no difference in weight change between the two groups at 24 months. Consistent with these findings, the present study found that differences in costs and QALYs between 10TT and usual care were

For peer review only - http://bmjopen.bmj.com/site/about/guidelines.xhtml

not significantly different over the two-year period. There is no evidence to either recommend or advise against 10TT, based on cost-effectiveness considerations.

Acknowledgements

Weight Concern and Cancer Research UK developed the 10 Top Tips intervention and materials, and Weight Concern provided training on how to deliver the intervention. The General Practice Research Framework provided advice on the study design and was involved in both recruiting sites and training the health professionals at each site on the trial procedures, along with providing quality control visits. Practices were located in: Wellingborough, Southampton, Bradford-on-Avon, Bromsgrove, Frome, Guisborough, Glastonbury, Ivybridge, Dunstable, Liskeard, Ledbury, New Mills, and London. The authors would like to acknowledge the substantial intellectual contribution made by Professor Jane Wardle who sadly passed away prior to publication, and is deeply missed by all of her co-authors, colleagues and students.

Contributors

NP undertook data analysis, interpreted the results, drafted, and wrote the manuscript. RJB helped design the study, undertook data collection and critically reviewed the manuscript. BL helped design the study, critically reviewed the data and manuscript. RZO helped design the study and critically reviewed the manuscript. IN helped design the study and critically reviewed the manuscript. SM undertook the data analysis, devised the analysis plan, interpreted the results and critically reviewed the manuscript.

Funding

For peer review only - http://bmjopen.bmj.com/site/about/guidelines.xhtml

This work was funded by the Medical Research Council (MRC) – National Prevention Research Initiative (NPRI) (UK) grant number G080202. RJB's post was funded through this grant and she is now supported by Yorkshire Cancer Research Academic Fellowship funding. SM, NP and IN are HEFCE-funded. RZO was funded by the National Institute for Health Research.

Disclaimer

The views expressed are those of the authors and not necessarily those of the funding bodies.

Competing interests

All authors have completed the ICMJE uniform disclosure form at http://www.icmje.org/coi_disclosure.pdf (available on request from the corresponding author) and declare that: NP, RJB, BL, RZO, IN and SM have nothing to disclose.

Ethics approval

This study has been approved by the South East London REC 2, 23/08/2010 (Reference number 10/H0802/59). All participants gave informed consent before taking part.

Provenance and peer review

Not commissioned; externally peer reviewed.

Data sharing statement

No additional data are available.

For peer review only

Table 1. Resource use, unit cost, and mean cost per participant for primary and secondary care services post randomisation

		Usual Care					10TT					Usual Care	10TT	Incremental difference	
	n	Mean	(SD)	Median	(IQR)	n	Mean	(SD)	Median	(IQR)	Unit cost (£)	Mean cost (£)	Mean cost (£)	Mean (£)	(95% CI)
**No imputation for missing values**															
GP surgery visit	204	9.4	(8.4)	7	(7.00)	182	9.7	(9.5)	7	(9.00)	45	417	425	8	(-72, 89)
GP home visit	196	0.1	(0.8)	0	(0.00)	176	0.0	(0.1)	0	(0.00)	292	37	9	-27	(-61, 7)
GP phone	199	1.3	(3.0)	0	(1.00)	179	1.3	(3.0)	0	(1.00)	27	34	34	0	(-16, 16)
GP practice nurse	206	6.9	(8.4)	4	(6.00)	184	6.8	(9.5)	4	(6.50)	40	274	268	-6	(-77, 64)
Nurse home visit	195	0.1	(1.1)	0	(0.00)	175	0.0	(0.1)	0	(0.00)	70	5	1	-4	(-16, 7)
GP practice nurse phone	197	0.5	(2.3)	0	(0.00)	176	0.4	(1.2)	0	(0.00)	10	5	4	-1	(-5, 2)
Dietician	195	0.1	(0.5)	0	(0.00)	175	0.1	(0.8)	0	(0.00)	35	4	4	0	(-4, 4)
Additional nurse visit	197	1.1	(3.1)	0	(1.00)	179	1.6	(2.6)	0	(1.00)	4	45	44	0	(-23, 22)
Inpatient admission	196	0.2	(0.7)	0	(0.00)	175	0.2	(0.7)	0	(0.00)	1713	372	387	14	(-220, 249)
Outpatient clinic	200	2.2	(3.5)	1	(3.00)	178	2.1	(3.3)	1	(3.00)	135	288	269	-20	(-110, 70)
Accident & Emergency visit	199	0.4	(1.2)	0	(0.00)	175	0.4	(0.9)	0	(1.00)	177	65	67	2	(-36, 39)
Other outpatient services	194	1.0	(1.8)	0	(1.00)	179	1.0	(2.3)	0	(1.00)	135	124	129	5	(-48, 58)
**Missing values imputed***			SE					SE							
GP surgery visit	270	9.4	(0.6)	9		267	9.4	(0.7)	9		45	419	420	1	(-79,81)
GP home visit	270	0.1	(0.5)	0		267	0.0	(0.2)	0		292	36	13	-23	(-58, 12)
GP phone	270	1.3	(0.2)	1		267	1.3	(0.2)	1		27	35	33	-2	(-19, 14)
GP practice nurse	270	6.9	(0.6)	6		267	6.6	(0.7)	6		40	274	266	-9	(-75, 57)
Nurse home visit	270	0.1	(1.0)	0		267	0.0	(0.1)	0		70	6	2	-4	(-16, 7)
GP practice nurse phone	270	0.5	(0.6)	0		267	0.4	(0.1)	0		10	5	4	-1	(-5, 2)
Dietician	270	0.1	(0.0)	0		267	0.1	(0.6)	0		35	4	4	0	(-4, 4)

For peer review only

For peer review only - http://bmjopen.bmj.com/site/about/guidelines.xhtml

Additional nurse visit	270	1.2	(0.2)	1		267	1.8	(0.2)	0		4	46	47	0	(-23, 23)
Inpatient admission	270	0.2	(0.1)	0		267	0.2	(0.1)	0		1713	373	379	6	(-238, 251)
Outpatient clinic	270	2.1	(0.3)	2		267	2.0	(0.2)	0		135	287	266	-22	(-106, 63)
Accident & Emergency visit	270	0.4	(0.9)	0		267	0.4	(0.7)	0		177	67	69	2	(-38, 42)
Other outpatient services	270	1	(0.1)	1		267	1	(0.2)	0		135	122	129	6	(-52, 65)
**Weight loss interventions**															
10TT		-	-	-	-		-	-	-	-	23	-	-		
Weight Watchers		-	-	-	-		-	-	-	-	78	-	-		
Slimming World		-	-	-	-		-	-	-	-	93	-	-		
Size Down Programme		-	-	-	-		-	-	-	-	73	-	-		

GP = general practitioner. SD = standard deviation for non-imputed data. SE = standard error for imputed data. CI = confidence interval. IQR = inter-quartile range. Costs are in 2013/14 UK£. *Missing values imputed using multiple imputation with 20 imputed datasets (see text).

Table 2. Demographics at baseline

	Usual care			10TT		
	n	Mean*	SD	n	Mean	SD
Age	270	58	12.61	267	57	12.88
Male	95	35.2	-	89	33.3	-
Female	175	64.8	-	178	66.7	-
Prior costs	270	1848	1948	267	2052	2461
Weight (kg)	269	101	17.46	267	100	16.98
Body Mass Index (BMI)	269	36.59	5.72	267	36.18	4.71
Baseline EQ-5D	257	0.76	0.24	255	0.74	0.27

SD = standard deviation. * Figures sex based on the mean proportion (%) in each group.

For peer review only - http://bmjopen.bmj.com/site/about/guidelines.xhtml

Table 3. Cost description two years prior and post randomisation

	Usual care			10TT			Incremental difference	
	n	Mean	SD	n	Mean	SD	Mean (£)	(95% CI)
**Cost, two years prior**								
Cost of primary and secondary care contacts	270	1516	1694	267	1735	2186	219	(-112, 550)
Drugs costs	270	332	748	267	317	624	-15	(-132, 102)
Total cost	270	1848	1948	267	2052	2461	204	(-172, 580)
**Costs after randomisation (imputed)***			**SE**			**SE**		
Cost of primary and secondary care contacts	270	1675	150	267	1631	169	-45	(-477, 387)
Drugs costs	270	249	44	267	236	68	-13	(-172, 146)
Intervention costs	-	-	-	267	23	-	-	-
Total cost	270	1925	165	267	1889	186	-36	(-512, 441)

SD = standard deviation for non-imputed data. SE = standard error for imputed data. CI = confidence interval

For peer review only - http://bmjopen.bmj.com/site/about/guidelines.xhtml

For peer review only

Table 4. Incremental cost-effectiveness of 10TT versus usual care

	Incremental cost		Incremental QALYs		Incremental net monetary benefit (NMB)				Probability cost-effective	
					£20,000		£30,000			
	Mean (£)	(95% CI)	Mean	(95% CI)	Mean (£)	(95% CI)	Mean (£)	(95% CI)	£20,000	£30,000
Base case*	-36	(-512, 441)	0.001	(-0.080, 0.082	49	(-1709, 1800)	55	(-2489, 2583)	52%	52%
Base case with control group intervention cost~	-122	(-598, 353)	0.001	(-0.080, 0.082)	140	(-1666, 1902)	148	(-2463, 2693)	56%	54%
Complete case analysis§	-66	(-907, 774)	-0.047	(-0.180, 0.086)	-889	(-3993, 2253)	-1361	(-5772, 3052)	29%	28%
Primary and secondary care cost increased #	-40	(-556, 475)	0.001	(-0.080, 0.081)	48	(-1743, 1851)	53	(-2520, 2623)	52%	51%
Drug cost increased #	23	(-396, 442)	0.001	(-0.080, 0.077)	-14	(-1734, 1716)	-9	(-2511, 2510)	49%	50%
All costs increased #	-39	(-554, 476)	0.001	(-0.080, 0.082)	43	(-1756, 1851)	46	(-2537, 2641)	52%	51%
Primary and secondary care cost decreased ¥	-31	(-463, 401)	0.001	(-0.080, 0.081)	54	(-1683, 1804)	64	(-2453, 2587)	52%	52%
Drug costs decreased ¥	26	(-387, 438)	0.001	(-0.080, 0.081)	0	(-1741, 1727)	11	(-2530, 2520)	50%	51%
All costs decreased ¥	-32	(-460, 395)	0.001	(-0.080, 0.081)	50	(-1682, 1811)	59	(-2458, 2599)	52%	52%

*N= 537. Data include values imputed using multiple imputation (see text). The 95% confidence intervals were derived from 1000 bootstrap replications of each of the 20 imputed datasets (see text). The incremental net monetary benefit and the probability 10TT is cost-effective are based on the QALYs gained and incremental costs and calculated at a maximum willingness to pay for a QALY of £20,000 and £30,000.

~ As for the base case analysis. Standard weight-loss interventions (Weight Watchers, Slimming World, Size Down programme) are included in the control group.

§As for the base case analysis. No multiple imputation of missing values and the 95% confidence intervals were derived from 1000 bootstrap replications of a single dataset containing the n=72 participants in the 10TT group and n=91 participants in the usual care group with no missing values. The costs of interventions in the control group (Weight Watchers, Slimming World, Size Down programme) were excluded.

# As for the base case analysis. Costs were increased by 10% for 10TT.

¥ As for the base case analysis. Costs were decreased by 10% for 10TT.

For peer review only - http://bmjopen.bmj.com/site/about/guidelines.xhtml

Table 5. Quality Adjusted Life Years (QALYs) per patient

	Usual Care				10TT				Incremental difference	
	n	Mean	SD	95% CI	n	Mean	SD	95% CI	Mean	(95% CI)
**No imputation for missing values**										
Utility at 3 months	206	0.77	0.23	(0.74, 0.80)	190	0.77	0.26	(0.73, 0.80)	0.00	(-0.05, 0.05)
Utility at 6 months	163	0.76	0.24	(0.72, 0.80)	159	0.78	0.24	(0.74, 0.82)	0.02	(-0.03, 0.07)
Utility at 12 months	151	0.77	0.24	(0.73, 0.81)	138	0.74	0.27	(0.70, 0.79)	-0.03	(-0.09, 0.03)
Utility at 18 months	138	0.75	0.25	(0.71, 0.79)	124	0.74	0.27	(0.69, 0.79)	-0.01	(-0.07, 0.05)
Utility at 24 months	157	0.76	0.25	(0.73, 0.80)	131	0.76	0.26	(0.72, 0.81)	0.00	(-0.06, 0.06)
**Discounted QALYs (24 months)**	92	1.52	0.40	(1.44 1.61)	73	1.47	0.47	(1.36, 1.58)	-0.05	(-0.18, 0.08)
**Missing values imputed***			**SE**				**SE**			
Utility at 3 months	270	0.77	0.016	(0.73, 0.80)	267	0.77	0.017	(0.73, 0.80)	0.00	(-0.05, 0.05)
Utility at 6 months	270	0.76	0.016	(0.73, 0.79)	267	0.78	0.018	(0.73, 0.81)	0.01	(-0.03, 0.06)
Utility at 12 months	270	0.76	0.019	(0.73, 0.80)	267	0.75	0.020	(0.71, 0.80)	-0.01	(-0.06, 0.04)
Utility at 18 months	270	0.76	0.019	(0.72, 0.79)	267	0.75	0.020	(0.71, 0.79)	-0.01	(-0.05, 0.04)
Utility at 24 months	270	0.76	0.019	(0.72, 0.79)	267	0.76	0.018	(0.72, 0.79)	0.00	(-0.05, 0.05)
**Discounted QALYs (24 months)**	270	1.51	0.030	(1.45, 1.57)	267	1.51	0.033	(1.44, 1.58)	0.00	(-0.08, 0.08)

QALY = quality-adjusted life year. SD = standard deviation for non-imputed data. SE = standard error for imputed data. CI = confidence interval. Costs are in 2013/14 UK£. CI were based on non-parametric bootstrapping for observed data and imputed data. * Missing values imputed using multiple imputation with 20 imputed datasets (see text).

For peer review only - http://bmjopen.bmj.com/site/about/guidelines.xhtml

Figure 1. Cost-Effectiveness plane, base case analysis assumptions. Based on 20,000 bootstrap replications, from 20 imputed datasets.

Figure 2. Cost-effectiveness acceptability curve showing the probability that 10TT is cost-effective vs usual care at different values of the maximum willingness to pay for a QALY (base-case assumptions)

QALY = quality adjusted life year

For peer review only

For peer review only - http://bmjopen.bmj.com/site/about/guidelines.xhtml

References

1. http://www.euro.who.int/en/health-topics/noncommunicable-diseases/obesity/data-and-statistics.
2. http://ec.europa.eu/eurostat/statistics-explained/index.php/Overweight_and_obesity_-_BMI_statistics.
3. Guh DP, Zhang W, Bansback N, Amarsi Z, Birmingham CL, Anis AH. The incidence of co-morbidities related to obesity and overweight: a systematic review and meta-analysis. BMC public health. 2009;9:88.
4. Reducing obesity and improving diet. https://www.gov.uk/government/policies/reducing-obesity-and-improving-diet. Accessed 07.03.15.
5. Roux L, Kuntz KM, Donaldson C, Goldie SJ. Economic evaluation of weight loss interventions in overweight and obese women. Obesity (Silver Spring, Md). 2006;14(6):1093-106.
6. Fuller NR, Carter H, Schofield D, Hauner H, Jebb SA, Colagiuri S, et al. Cost effectiveness of primary care referral to a commercial provider for weight loss treatment, relative to standard care: a modelled lifetime analysis. Int J Obes. 2014;38(8):1104-9.
7. Fuller NR, Colagiuri S, Schofield D, Olson AD, Shrestha R, Holzapfel C, et al. A within-trial cost-effectiveness analysis of primary care referral to a commercial provider for weight loss treatment, relative to standard care--an international randomised controlled trial. International journal of obesity (2005). 2013;37.
8. Tsai AG, Wadden TA, Volger S, Sarwer DB, Vetter M, Kumanyika S, et al. Cost-effectiveness of a primary care intervention to treat obesity. International journal of obesity (2005). 2013;37 Suppl 1:S31-7.
9. Trueman P, Haynes SM, Felicity Lyons G, Louise McCombie E, McQuigg MS, Mongia S, et al. Long-term cost-effectiveness of weight management in primary care. International journal of clinical practice. 2010;64(6):775-83.
10. Gardner B, Lally P, Wardle J. Making health habitual: the psychology of 'habit-formation' and general practice. The British journal of general practice : the journal of the Royal College of General Practitioners. 2012;62(605):664-6.
11. Lally P, Chipperfield A, Wardle J. Healthy habits: efficacy of simple advice on weight control based on a habit-formation model. Int J Obes. 2007;32(4):700-7.
12. http://www.nhs.uk/Livewell/5ADAY/Pages/Why5ADAY.aspx.
13. Beeken RJ, Leurent B, Vickerstaff V, Wilson R, Croker H, Morris S, et al. A brief intervention for weight control based on habit-formation theory delivered through primary care: results from a randomised controlled trial. International journal of obesity (2005). 2017.
14. National Institute for Health and Care Excellence (NICE). Guide to the methods of technology appraisal. London: NICE; 2013.
15. Curtis L. Unit costs of health and social care 2013. Kent: Personal Social Services Research Unit; 2013.
16. Department of Health. National Schedule of Reference Costs- 2012-2013. NHS trusts and NHS foundation trusts. London Department of Health; 2012.
17. Joint Formulary Committee . British National Formulary, 68th edition. London: BMJ Group and Pharmaceutical Press; 2013 [Available from: https://www.medicinescomplete.com/mc/bnf/current/.
18. Jolly K, Lewis A, Beach J, Denley J, Adab P, Deeks JJ, et al. Comparison of range of commercial or primary care led weight reduction programmes with minimal intervention control for weight loss in obesity: lighten Up randomised controlled trial. BMJ (Clinical research ed). 2011;343:d6500.

For peer review only - http://bmjopen.bmj.com/site/about/guidelines.xhtml

19. Rabin R, de Charro F. EQ-5D: a measure of health status from the EuroQol Group. Annals of medicine. 2001;33(5):337-43.
20. Brooks R. EuroQol: the current state of play. Health policy (Amsterdam, Netherlands). 1996;37(1):53-72.
21. Dolan P. Modeling valuations for EuroQol health states. Medical care. 1997;35(11):1095-108.
22. Petrou S, Dakin H, Abangma G, Benge S, Williamson I. Cost-utility analysis of topical intranasal steroids for otitis media with effusion based on evidence from the GNOME trial. Value in health : the journal of the International Society for Pharmacoeconomics and Outcomes Research. 2010;13(5):543-51.
23. Briggs A, Clark T, Wolstenholme J, Clarke P. Missing... presumed at random: cost-analysis of incomplete data. Health economics. 2003;12(5):377-92.
24. Royston P Multiple imputation of missing values: update of ice. Stata J. 2005: 527-36.
25. StataCorp. 2015. Stata Statistical Software: Release 14. College Station, TX: StataCorp LP. .
26. Sinharay S, Stern HS, Russell D. The use of multiple imputation for the analysis of missing data. Psychological methods. 2001;6(4):317-29.
27. Yang X, Belin TR, Boscardin WJ. Imputation and variable selection in linear regression models with missing covariates. Biometrics. 2005;61(2):498-506.
28. Zethraeus N, Johannesson M, Jonsson B, Lothgren M, Tambour M. Advantages of using the net-benefit approach for analysing uncertainty in economic evaluation studies. PharmacoEconomics. 2003;21(1):39-48.
29. Pasta DJ, Taylor JL, Henning JM. Probabilistic sensitivity analysis incorporating the bootstrap: an example comparing treatments for the eradication of Helicobacter pylori. Medical decision making : an international journal of the Society for Medical Decision Making. 1999;19(3):353-63.
30. Briggs AH, Gray AM. Handling uncertainty when performing economic evaluation of healthcare interventions. Health technology assessment (Winchester, England). 1999;3(2):1-134.
31. Ahern AL, Wheeler GM, Aveyard P, Boyland EJ, Halford JCG, Mander AP, et al. Extended and standard duration weight-loss programme referrals for adults in primary care (WRAP): a randomised controlled trial. Lancet. 2017;389(10085):2214-25.
32. National Institute for Health and Care Excellence. Weight management: lifetsyle services for overweight or obesity adults, NICE guideline. London: NICE, 2014.
33. Kolotkin RL, Norquist JM, Crosby RD, Suryawanshi S, Teixeira PJ, Heymsfield SB, et al. One-year health-related quality of life outcomes in weight loss trial participants: comparison of three measures. Health and quality of life outcomes. 2009;7:53.
34. Peckmezian T, Hay P. A systematic review and narrative synthesis of interventions for uncomplicated obesity: weight loss, well-being and impact on eating disorders. Journal of eating disorders. 2017;5:15.
35. Herrett E, Shah AD, Boggon R, Denaxas S, Smeeth L, van Staa T, et al. Completeness and diagnostic validity of recording acute myocardial infarction events in primary care, hospital care, disease registry, and national mortality records: cohort study. BMJ (Clinical research ed). 2013;346:f2350.
36. Sterne JA, White IR, Carlin JB, Spratt M, Royston P, Kenward MG, et al. Multiple imputation for missing data in epidemiological and clinical research: potential and pitfalls. BMJ (Clinical research ed). 2009;338:b2393.
37. Lee KJ, Simpson JA. Introduction to multiple imputation for dealing with missing data. Respirology (Carlton, Vic). 2014;19(2):162-7.
38. Hayati Rezvan P, Lee KJ, Simpson JA. The rise of multiple imputation: a review of the reporting and implementation of the method in medical research. BMC medical research methodology. 2015;15:30.

For peer review only

For peer review only - http://bmjopen.bmj.com/site/about/guidelines.xhtml

For peer review only

For peer review only - http://bmjopen.bmj.com/site/about/guidelines.xhtml

Figure 1. Cost-Effectiveness plane, base case analysis assumptions. Based on 20,000 bootstrap replications, from 20 imputed datasets.

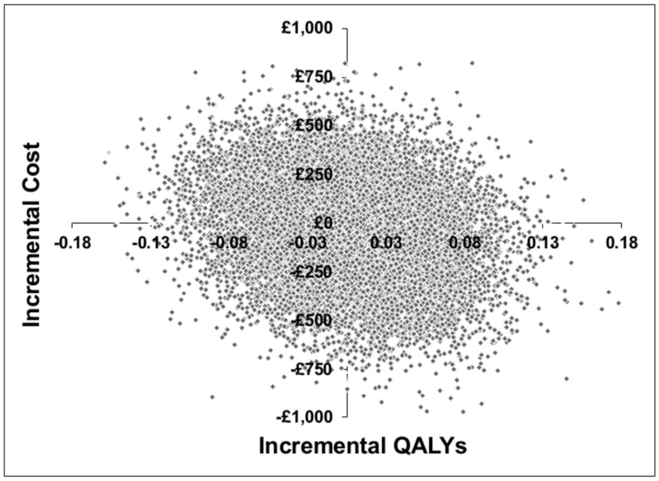

1

Figure 1. Cost-Effectiveness plane, base case analysis assumptions. Based on 20,000 bootstrap replications, from 20 imputed datasets.

296x209mm (300 x 300 DPI)

Figure 2. Cost-effectiveness acceptability curve showing the probability that 10TT is cost-effective vs usual care at different values of the maximum willingness to pay for a QALY (base-case assumptions)

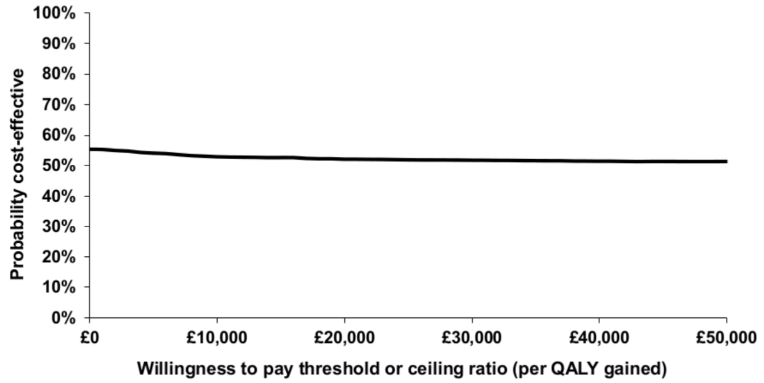

QALY = quality adjusted life year

Figure 2. Cost-effectiveness acceptability curve showing the probability that 10TT is cost-effective vs usual care at different values of the maximum willingness to pay for a QALY (base-case assumptions)

296x209mm (300 x 300 DPI)

BMJ

BMJ 2013;346:f1049 doi: 10.1136/bmj.f1049 (Published 25 March 2013) Page 1 of 6

RESEARCH METHODS & REPORTING

Consolidated Health Economic Evaluation Reporting Standards (CHEERS) statement

OPEN ACCESS

Don Husereau *senior associate; adjunct professor of medicine; senior scientist*[1 2 3], Michael Drummond *co-editor-in-chief, Value in Health; professor of health economics*[4], Stavros Petrou *professor of health economics*[5], Chris Carswell *editor*[6], David Moher *senior scientist*[7], Dan Greenberg *associate professor and chairman; visiting assistant professor*[8 9], Federico Augustovski *director; professor of public health*[10 11], Andrew H Briggs *William R Lindsay chair of health economics, health economics and health technology assessment*[12], Josephine Mauskopf *vice president of health economics*[13], Elizabeth Loder *chief of division; clinical epidemiology editor, BMJ*[14], on behalf of the CHEERS Task Force

[1]Institute of Health Economics, Edmonton, Canada; [2]Department of Epidemiology and Community Medicine, University of Ottawa, Ottawa, Canada; [3]University for Health Sciences, Medical Informatics and Technology, Hall in Tirol, Austria; [4]Centre for Health Economics, University of York, York, UK; [5]Warwick Medical School, University of Warwick, Coventry, UK; [6]Pharmacoeconomics, Adis International, Auckland, New Zealand; [7]Clinical Epidemiology Program, Ottawa Hospital Research Institute, Ottawa, Canada; [8]Department of Health Systems Management, Faculty of Health Sciences, Ben-Gurion University of the Negev, Beer-Sheva, Israel; [9]Center for the Evaluation of Value and Risk in Health, Tufts Medical Center, Boston MA, USA; [10]Health Economic Evaluation and Technology Assessment, Institute for Clinical Effectiveness and Health Policy (IECS), Buenos Aires, Argentina; [11]Universidad de Buenos Aires, Buenos Aires, Argentina; [12]Institute of Health and Wellbeing, University of Glasgow, Glasgow, Scotland; [13]RTI Health Solutions, Research Triangle Park NC, USA; [14]Division of Headache and Pain, Brigham and Women's/Faulkner Neurology, Faulkner Hospital, Boston MA, USA

Abstract

Economic evaluations of health interventions pose a particular challenge for reporting. There is also a need to consolidate and update existing guidelines and promote their use in a user friendly manner. The Consolidated Health Economic Evaluation Reporting Standards (CHEERS) statement is an attempt to consolidate and update previous health economic evaluation guidelines efforts into one current, useful reporting guidance. The primary audiences for the CHEERS statement are researchers reporting economic evaluations and the editors and peer reviewers assessing them for publication.

The need for new reporting guidance was identified by a survey of medical editors. A list of possible items based on a systematic review was created. A two round, modified Delphi panel consisting of representatives from academia, clinical practice, industry, government, and the editorial community was conducted. Out of 44 candidate items, 24 items and accompanying recommendations were developed. The recommendations are contained in a user friendly, 24 item checklist. A copy of the statement, accompanying checklist, and this report can be found on the ISPOR Health Economic Evaluations Publication Guidelines Task Force website (www.ispor.org/TaskForces/EconomicPubGuidelines.asp).

We hope CHEERS will lead to better reporting, and ultimately, better health decisions. To facilitate dissemination and uptake, the CHEERS statement is being co-published across 10 health economics and medical journals. We encourage other journals and groups, to endorse CHEERS. The author team plans to review the checklist for an update in five years.

Health economic evaluations are conducted to inform resource allocation decisions. Economic evaluation has been defined as "the comparative analysis of alternative courses of action in terms of both their costs and their consequences."[1] All economic evaluations assess costs, but approaches to measuring and valuing the consequences of health interventions may differ (see box).

Economic evaluations have been widely applied in health policy, including the assessment of prevention programmes (such as vaccination, screening, and health promotion), diagnostics, treatment interventions (such as drugs and surgical procedures), organisation of care, and rehabilitation. Economic evaluations are increasingly being used for decision making and are an important component of programmes for health technology assessment internationally.[2]

Correspondence to: D Husereau, 879 Winnington Ave, Ottawa, ON K2B 5C4, Canada donh@donhusereau.com

For peer review only - http://bmjopen.bmj.com/site/about/guidelines.xhtml

No commercial reuse: See rights and reprints http://www.bmj.com/permissions Subscribe: http://www.bmj.com/subscribe

Forms of economic evaluation

Specific forms of analysis reflect different approaches to evaluating the consequences of health interventions. Health consequences may be estimated from a single analytical (experimental or non-experimental) study, a synthesis of studies, mathematical modelling, or a combination of modelling and study information.

Cost consequences analysis examines costs and consequences without attempting to isolate a single consequence or aggregate consequences into a single measure

Cost minimisation analysis (CMA)—The consequences of compared interventions are required to be equivalent, and only relative costs are compared

Cost effectiveness analysis (CEA) measures consequences in natural units, such as life years gained, disability days avoided, or cases detected. In a variant of CEA, often called cost utility analysis, consequences are measured in terms of preference-based measures of health, such as quality adjusted life years or disability adjusted life years.

Cost benefit analysis—Consequences are valued in monetary units.

Readers should be aware that an economic evaluation might be referred to as a "cost effectiveness analysis" or "cost benefit analysis" even if it does not strictly adhere to the definitions above. Multiple forms may also exist within a single evaluation. Different forms of analysis provide unique advantages or disadvantages for decision making. The Consolidated Health Economic Evaluation Reporting Standards (CHEERS) statement can be used with any form of economic evaluation.

Reporting challenges and shortcomings in health economic evaluations

Compared with clinical studies, which report the consequences of an intervention only, economic evaluations require more reporting space for additional items, such as resource use, costs, preference related information, and cost effectiveness results. This creates challenges for editors, reviewers, and those who wish to scrutinise a study's findings.[3] There is evidence that the quality of reporting of economic evaluations varies widely and could potentially benefit from improved quality assurance mechanisms.[4 5]

With the increasing number of publications available, and opportunity costs from decisions based on misleading study findings, transparency and clarity in reporting are important. In addition, outside of economic evaluations conducted alongside clinical trials, there are no widespread mechanisms for warehousing economic evaluation data to allow for independent interrogation, such as ethics review proceedings, regulator dossiers, or study registries. Instead, independent analysis may rely on the record keeping of individual investigators.

Even if measures to promote transparency exist, such as registries, biomedical journal editors have increasingly promoted and endorsed the use of reporting guidelines. Endorsement of guidelines by journals for randomised controlled trials has been shown to improve reporting.[6] The combination of the risk of making costly decisions due to poor reporting with the lack of mechanisms that promote accountability makes transparency in reporting economic evaluations especially important and a primary concern among journal editors and decision makers.[3 7]

Aim and scope

The aim of the Consolidated Health Economic Evaluation Reporting Standards (CHEERS) statement is to provide recommendations, in the form of a checklist, to optimise reporting of health economic evaluations. The need for a contemporary reporting guidance for economic evaluations was recently identified by researchers and biomedical journal editors.[8] The CHEERS statement attempts to consolidate and update previous efforts [9-20] into a single useful reporting guidance.

The primary audiences for the CHEERS statement are researchers reporting economic evaluations and the editors and peer reviewers evaluating their publication potential. We hope the statement (which consists of a 24 item checklist and accompanying recommendations on the minimum amount of information to be included when reporting economic evaluations) is a useful and practical tool for these audiences and will improve reporting and, in turn, health and healthcare decisions. To best understand and apply the recommendations contained within the statement, we encourage readers to access the Explanation and Elaboration Report.[21]

Development of the CHEERS statement

The statement was developed by a task force supported by the International Society for Pharmacoeconomics and Outcomes Research (ISPOR), as part of a broader initiative to facilitate and encourage the interchange of expert knowledge and develop best practices. The CHEERS Task Force members were chosen by the chair of the task force primarily based on their longstanding academic expertise and contribution to the multidisciplinary field of health economic evaluation. In addition to four members of the task force with doctorates in economics and its sub-discipline of health economics (AHB, MD, JM, SP), members included experts in health technology assessment and decision making (FA, AHB, DH, MD, JM) and in clinical epidemiology and biostatistics (AHB, EL, DM), those in active clinical practice (EL, FA), and those with previous experience in reporting guideline development (MD, DM). All members are researchers in applied health and health policy, with five members currently serving as editors for journals in the field (AHB, CC, MD, DG, EL).

The CHEERS Task Force followed current recommendations for developing reporting guidelines.[22] Briefly, the need for new guidance was first identified through a survey of members of the World Association of Medical Editors. Of the 6% (55/965) who responded, 91% (n=50) indicated they would use a standard if one were widely available.[8] Next, published checklists or guidance documents related to reporting economic evaluations were identified from a systematic review and survey of task force members.[23] Both of these activities were used to create a preliminary list of items to include when reporting economic evaluations. Recommendations of the minimum set of reporting items were then developed through a modified Delphi panel process. Forty eight individuals identified by the task force with broad geographical representation and representing academia, biomedical journal editors, the pharmaceutical industry, government decision makers, and those in clinical practice were invited to participate. Thirty eight agreed to participate. Participants were asked to score importance on a Likert scale and the average scores, weighted by each individual's confidence in ability to score, were then used to rank items. A cut-off point was applied to the ranked list to determine the minimum number of items important for reporting.

The CHEERS statement recommendations have been independently reviewed and subsequently revised by task force members. The recommendations are entirely those of the task

For peer review only - http://bmjopen.bmj.com/site/about/guidelines.xhtml

No commercial reuse: See rights and reprints http://www.bmj.com/permissions Subscribe: http://www.bmj.com/subscribe

BMJ 2013;346:f1049 doi: 10.1136/bmj.f1049 (Published 25 March 2013)

force—the sponsors of the study had no role in study design, data analysis, data interpretation, or writing of the final recommendations. A more complete description of the methods and findings of the Delphi panel are found in the larger explanation and elaboration document.[21]

Checklist items

The final recommendations are subdivided into six main categories: (1) title and abstract; (2) introduction; (3) methods; (4) results; (5) discussion; and (6) other. The recommendations are contained in a user friendly, 24 item checklist (table⇓) to aid users who wish to follow them. A copy of the checklist can also be found on the CHEERS Task Force website. (www.ispor.org/TaskForces/EconomicPubGuidelines.asp). In order to encourage dissemination and use of a single international standard for reporting, the task force approached 14 journals identified as either the largest publishers of economic evaluations or widely read by the medical and research community. Thirteen journals responded, and 10 expressed their ability and interest in endorsing this guidance. The CHEERS statement is being simultaneously published in *BMC Medicine*, *BMJ*, *BJOG: An International Journal of Obstetrics and Gynaecology*, *Clinical Therapeutics*, *Cost Effectiveness and Resource Allocation*, *The European Journal of Health Economics*, *International Journal of Technology Assessment in Health Care*, *Journal of Medical Economics*, *Pharmacoeconomics*, and *Value in Health*. To facilitate wider dissemination and uptake of this reporting guidance, we encourage other journals and groups to consider endorsing CHEERS.

Concluding remarks

As the number of published health economic evaluations continues to grow, we believe more transparent and complete reporting of methods and findings will be increasingly important to facilitate interpretation and comparison of studies. We hope the CHEERS statement, consisting of recommendations in a 24 item checklist, will be viewed as an effective consolidation and update of previous efforts and serve as a starting point for standard reporting going forward.

We believe the CHEERS statement represents a considerable expansion over previous efforts. The strength of our approach is that it was developed in accordance with current recommendations for the development of reporting guidelines, using an international and multidisciplinary team of editors and content experts in economic evaluation and reporting.[22] Similar to the approach taken with other widely accepted guidelines, we have defined a minimum set of criteria though a modified Delphi technique and have translated these into recommendations, an explanatory document with explanations, and a checklist. Unlike some previous reporting guidance for economic evaluation, we have also made every effort to be neutral about the conduct of economic evaluation, allowing analysts the freedom to choose different methods.

There may be several limitations to our approach. A larger Delphi panel with a different composition could have led to a different final set of recommendations.[24] Some less common approaches and contexts (such as public health, developing countries, and system dynamic models) for conducting health economic evaluation may not be well represented by our sample of experts. Additionally, like many Delphi panel processes, we based decisions to reject or accept criteria on arbitrary levels of importance. However, we feel the group recruited to create the statement is sufficiently knowledgeable of the more common applications of economic evaluation, and the rules used to select criteria were created a priori and are consistent with previous efforts.

We believe it will be important to evaluate the effects of implementation of this statement and checklist on reporting in future economic evaluations. As methods for the conduct of economic evaluation continue to evolve, it will also be important to revisit or extend the guidance. The CHEERS Task Force feels that this statement should be reviewed for updating five years from its release.

Competing interests: All authors have completed the ICMJE uniform disclosure form at www.icmje.org/coi_disclosure.pdf and declare: FA served as board member for the study funder; FA, AHB, CC, MD, DG, DH, EL, JM, and SP were provided support for travel to a face-to-face meeting to discuss the contents of the report; FA and MD have received payment from the study sponsor for serving as co-editors for *Value in Health*; no other relationships or activities that could appear to have influenced the submitted work.

Elizabeth Loder—*BMJ* clinical epidemiology editor—played no part in the peer review or decision making of this paper at the editorial level, and contributed solely as an author.

The International Society for Pharmacoeconomics and Outcomes Research (ISPOR) Health Economic Evaluation Publication Guidelines—CHEERS Task Force acknowledge the support of Elizabeth Molsen; Donna Rindress, who provided the initial leadership for this effort; and the reviewers and Delphi panel participants, who are named in the larger explanation and elaboration document and on the CHEERS Task Force website.

Contributors: All authors provided a substantial contribution to the design and interpretation of the protocol and guidance, as well as writing sections of drafts, revising based on comments received, and approving the final version. DH conducted the analysis of Delphi panel responses, drafted and revised the protocol and the drafts of this paper, and is the guarantor for the study.

Funding: All CHEERS Task Force members are volunteers. Support for this initiative was provided by the International Society for Pharmacoeconomics and Outcomes Research.

1 Drummond MF, Sculpher MJ, Torrance G, O'Brien J, Stoddart GL. *Methods for the economic evaluation of health care programmes*. 3rd ed. Oxford University Press, 2005.
2 Drummond MF, Schwartz JS, Jönsson B, Luce BR, Neumann PJ, Siebert U, et al. Key principles for the improved conduct of health technology assessments for resource allocation decisions. *Int J Technol Assess Health Care* 2008;24:244-58.
3 Rennie D, Luft HS. Pharmacoeconomic analyses. *JAMA* 2000;283:2158-60.
4 Neumann PJ, Stone PW, Chapman RH, Sandberg EA, Bell CM. The quality of reporting in published cost-utility analyses, 1976-1997. *Ann Intern Med* 2000;132:964.
5 Rosen AB, Greenberg D, Stone PW, Olchanski NV, Neumann PJ. Quality of abstracts of papers reporting original cost-effectiveness analyses. *Med Decis Making* 2005;25:424-8.
6 Turner L, Shamseer L, Altman DG, Schulz KF, Moher D. Does use of the CONSORT Statement impact the completeness of reporting of randomised controlled trials published in medical journals? A Cochrane review. *Syst Rev* 2012;1:60.
7 Drummond MF. A reappraisal of economic evaluation of pharmaceuticals. Science or marketing? *Pharmacoeconomics* 1998;14:1-9.
8 McGhan WF, Al M, Doshi JA, Kamae I, Marx SE, Rindress D. The ISPOR Good Practices for Quality Improvement of Cost-Effectiveness Research Task Force report. *Value Health* 2009;12:1086-99.
9 Task Force on Principles for Economic Analysis of Health Care Technology. Economic analysis of health care technology. A report on principles. *Ann Intern Med* 1995;123:61-70.
10 Drummond MF, Jefferson TO. Guidelines for authors and peer reviewers of economic submissions to the BMJ. *BMJ* 1996;313:275-83.
11 Gold MR. *Cost-effectiveness in health and medicine*. Oxford University Press, 1996.
12 Siegel JE, Weinstein MC, Russell LB, Gold MR. Recommendations for reporting cost-effectiveness analyses. Panel on Cost-Effectiveness in Health and Medicine. *JAMA* 1996;276:1339-41.
13 Nuijten MJ, Pronk MH, Brorens MJ, Hekster YA, Lockefeer JH, de Smet PA, et al. Reporting format for economic evaluation: Part II: Focus on modelling studies. *Pharmacoeconomics* 1998;14:259-68.
14 Vintzileos AM, Beazoglou T. Design, execution, interpretation, and reporting of economic evaluation studies in obstetrics. *Am J Obstet Gynecol* 2004;191:1070-6.
15 Drummond M, Manca A, Sculpher M. Increasing the generalizability of economic evaluations: recommendations for the design, analysis, and reporting of studies. *Int J Technol Assess Health Care* 2005;21:165-71.

For peer review only - http://bmjopen.bmj.com/site/about/guidelines.xhtml

No commercial reuse: See rights and reprints http://www.bmj.com/permissions Subscribe: http://www.bmj.com/subscribe

BMJ 2013;346:f1049 doi: 10.1136/bmj.f1049 (Published 25 March 2013)

16 Ramsey S, Willke R, Briggs A, Brown R, Buxton M, Chawla A, et al. Good research practices for cost-effectiveness analysis alongside clinical trials: the ISPOR RCT-CEA Task Force report. *Value Health* 2005;8:521-33.

17 Goetghebeur MM, Wagner M, Khoury H, Levitt RJ, Erickson LJ, Rindress D. Evidence and value: impact on decisionmaking—the EVIDEM framework and potential applications. *BMC Health Serv Res* 2008;8:270.

18 Davis JC, Robertson MC, Comans T, Scuffham PA. Guidelines for conducting and reporting economic evaluation of fall prevention strategies. *Osteoporos Int* 2010;22:2449-59.

19 Petrou S, Gray A. Economic evaluation using decision analytical modelling: design, conduct, analysis, and reporting. *BMJ* 2011;342:d1766.

20 Petrou S, Gray A. Economic evaluation alongside randomised controlled trials: design, conduct, analysis, and reporting. *BMJ* 2011;342:d1548.

21 Husereau D, Drummond M, Petrou S, Carswell C, Moher D, Greenberg D, et al. Consolidated Health Economic Evaluation Reporting Standards (CHEERS)—explanation and elaboration: a report of the ISPOR Health Economic Evaluations Publication Guidelines Task Force. *Value in Health* (forthcoming).

22 Moher D, Schulz KF, Simera I, Altman DG. Guidance for developers of health research reporting guidelines. *PLoS Med* 2010;7:e1000217.

23 Moher D, Simera I, Schulz K, Miller D, Grimshaw J, Hoey J, et al. Reporting guidelines for clinical research: a systematic review. Vancouver, BC, 2009. Available at: www.ama-assn.org/public/peer/abstracts-0912.pdf.

24 Campbell SM, Hann M, Roland MO, Quayle JA, Shekelle PG. The effect of panel membership and feedback on ratings in a two round Delphi survey: results of a randomized controlled trial. *Med Care* 1999;37:964-8.

Cite this as: *BMJ* 2013;346:f1049

This is an open-access article distributed under the terms of the Creative Commons Attribution Non-commercial License, which permits use, distribution, and reproduction in any medium, provided the original work is properly cited, the use is non commercial and is otherwise in compliance with the license. See: http://creativecommons.org/licenses/by-nc/2.0/ and http://creativecommons.org/licenses/by-nc/2.0/legalcode

For peer review only

No commercial reuse: See rights and reprints http://www.bmj.com/permissions Subscribe: http://www.bmj.com/subscribe

BMJ 2013;346:f1049 doi: 10.1136/bmj.f1049 (Published 25 March 2013)

Table

Table 1| CHEERS checklist—Items to include when reporting economic evaluations of health interventions

Section/item	Item No	Recommendation	Reported on page No/ line No
**Title and abstract**			
Title	1	Identify the study as an economic evaluation or use more specific terms such as "cost-effectiveness analysis", and describe the interventions compared.	1
Abstract	2	Provide a structured summary of objectives, perspective, setting, methods (including study design and inputs), results (including base case and uncertainty analyses), and conclusions.	23
**Introduction**			
Background and objectives	3	Provide an explicit statement of the broader context for the study.	102 – 133
		Present the study question and its relevance for health policy or practice decisions.	121 – 133
**Methods**			
Target population and subgroups	4	Describe characteristics of the base case population and subgroups analysed, including why they were chosen.	102 – 109
Setting and location	5	State relevant aspects of the system(s) in which the decision(s) need(s) to be made.	104 – 107
Study perspective	6	Describe the perspective of the study and relate this to the costs being evaluated.	126
Comparators	7	Describe the interventions or strategies being compared and state why they were chosen.	121 – 122
Time horizon	8	State the time horizon(s) over which costs and consequences are being evaluated and say why appropriate.	129 – 130
Discount rate	9	Report the choice of discount rate(s) used for costs and outcomes and say why appropriate.	132 – 133
Choice of health outcomes	10	Describe what outcomes were used as the measure(s) of benefit in the evaluation and their relevance for the type of analysis performed.	174
Measurement of effectiveness	11a	*Single study-based estimates:* Describe fully the design features of the single effectiveness study and why the single study was a sufficient source of clinical effectiveness data.	174 – 183
	11b	*Synthesis-based estimates:* Describe fully the methods used for identification of included studies and synthesis of clinical effectiveness data.	N/A
Measurement and valuation of preference based outcomes	12	If applicable, describe the population and methods used to elicit preferences for outcomes.	N/A 174.
Estimating resources and costs	13a	*Single study-based economic evaluation:* Describe approaches used to estimate resource use associated with the alternative interventions. Describe primary or secondary research methods for valuing each resource item in terms of its unit cost. Describe any adjustments made to approximate to opportunity costs.	135 – 149
	13b	*Model-based economic evaluation:* Describe approaches and data sources used to estimate resource use associated with model health states. Describe primary or secondary research methods for valuing each resource item in terms of its unit cost. Describe any adjustments made to approximate to opportunity costs.	N/A
Currency, price date, and conversion	14	Report the dates of the estimated resource quantities and unit costs. Describe methods for adjusting estimated unit costs to the year of reported costs if necessary. Describe methods for converting costs into a common currency base and the exchange rate.	142.
Choice of model	15	Describe and give reasons for the specific type of decision-analytical model used. Providing a figure to show model structure is strongly recommended.	N/A
Assumptions	16	Describe all structural or other assumptions underpinning the decision-analytical model.	
Analytical methods	17	Describe all analytical methods supporting the evaluation. This could include methods for dealing with skewed, missing, or censored data; extrapolation methods; methods for pooling data; approaches to validate or make adjustments (such as half cycle corrections) to a model; and methods for handling population heterogeneity and uncertainty.	186 – 221
**Results**			
Study parameters	18	Report the values, ranges, references, and, if used, probability distributions for all parameters. Report reasons or sources for distributions used to represent uncertainty where appropriate. Providing a table to show the input values is strongly recommended.	Table 4
Incremental costs and outcomes	19	For each intervention, report mean values for the main categories of estimated costs and outcomes of interest, as well as mean differences between the comparator groups. If applicable, report incremental cost-effectiveness ratios.	254 – 259 241 – 244
Characterising uncertainty	20a	*Single study-based economic evaluation:* Describe the effects of sampling uncertainty for the estimated incremental cost and incremental effectiveness parameters, together with the impact of methodological assumptions (such as discount rate, study perspective).	278 – 290

For peer review only

No commercial reuse: See rights and reprints http://www.bmj.com/permissions Subscribe: http://www.bmj.com/subscribe

BMJ 2013;346:f1049 doi: 10.1136/bmj.f1049 (Published 25 March 2013)

(continued)

Section/item	Item No	Recommendation	Reported on page No/ line No
	20b	*Model-based economic evaluation:* Describe the effects on the results of uncertainty for all input parameters, and uncertainty related to the structure of the model and assumptions.	N/A
Characterising heterogeneity	21	If applicable, report differences in costs, outcomes, or cost-effectiveness that can be explained by variations between subgroups of patients with different baseline characteristics or other observed variability in effects that are not reducible by more information.	217-219
**Discussion**			
Study findings, limitations, generalisability, and current knowledge	22	Summarise key study findings and describe how they support the conclusions reached. Discuss limitations and the generalisability of the findings and how the findings fit with current knowledge.	292-296 321-377
**Other**			
Source of funding	23	Describe how the study was funded and the role of the funder in the identification, design, conduct, and reporting of the analysis. Describe other non-monetary sources of support.	397-401
Conflicts of interest	24	Describe any potential for conflict of interest of study contributors in accordance with journal policy. In the absence of a journal policy, we recommend authors comply with International Committee of Medical Journal Editors recommendations.	406-408

For consistency, the CHEERS statement checklist format is based on the format of the CONSORT statement checklist

For peer review only

No commercial reuse: See rights and reprints http://www.bmj.com/permissions Subscribe: http://www.bmj.com/subscribe

BMJ Open

Cost-effectiveness of habit-based advice for weight control versus usual care in general practice in the Ten Top Tips (10TT) Trial: economic evaluation based on a randomised controlled trial

Journal:	*BMJ Open*
Manuscript ID	bmjopen-2017-017511.R4
Article Type:	Research
Date Submitted by the Author:	10-May-2018
Complete List of Authors:	Patel, Nishma; UCL, Department of Applied Health Research<br>Beeken, Rebecca; University of Leeds, Leeds Institute of Health Sciences Clarendon Way LS2 9NL<br>Leurent, Baptiste; London School of Hygiene and Tropical Medicine, Department of Infectious Disease Epidemiology<br>Omar, Rumana; University College London,<br>Nazareth, Irwin; UCL, Primary Care and Population Health<br>Morris, Stephen; University College London, Department of Applied Health Research
**Primary Subject Heading**:	Health economics
Secondary Subject Heading:	Public health, General practice / Family practice
Keywords:	Weight Loss programme, PRIMARY CARE, Cost-effectiveness

SCHOLARONE™ Manuscripts

For peer review only

Cost-effectiveness of habit-based advice for weight control versus usual care in general practice in the Ten Top Tips (10TT) Trial: economic evaluation based on a randomised controlled trial

Nishma Patel[1], Rebecca J. Beeken[2,3], Baptiste Leurent[4], Rumana Z. Omar[5], Irwin Nazareth[6] and Stephen Morris[1]

[1] Department of Applied Health Research, University College London, Gower Street, London, WC1E 6BT, UK

[2] Department of Behavioural Science & Health, University College London, Gower Street, London, WC1E 6BT, UK

[3]Leeds Institute of Health Sciences, University of Leeds, Clarendon Way, Leeds, LS2 9NL

[4] London School of Hygiene and Tropical Medicine, Keppel Street, WC1E 7HT

[5] Department of Statistical Science, University College London, Gower Street, London, WC1E 6BT, UK

[6]Deparment of Primary Care & Population Health, UCL, Gower Street, London, WC1E 6BT

Correspondence to: Nishma Patel, Department of Applied Health Research, University College London WC1E 6BT, UK. Telephone: +44 2031 083027 (email: nishma.patel@ucl.ac.uk)

For peer review only

Abstract

Objective: Ten Top Tips (10TT) is a primary care led behavioural intervention, which aims to help adults reduce and manage their weight by following ten weight loss tips. The intervention promotes habit formation to encourage long-term behavioural changes. The aim of this study was to estimate the cost-effectiveness of 10TT in general practice from the perspective of the UK National Health Service.

Design: An economic evaluation was conducted alongside an individually randomised controlled trial.

Setting: 14 General Practitioner (GP) practices in England.

Participants: All patients were aged ≥18 years, with BMI ≥30kg/m^2. A total of 537 patients were recruited; 270 received the usual care offered by their practices and 267 received the 10TT intervention.

Outcomes measures: Health service use and quality-adjusted life years (QALY) were measured over two years. Analysis was conducted in terms of incremental net monetary benefits (NMBs), using non-parametric bootstrapping and multiple imputation.

Results: Over a two-year time horizon, mean costs and QALYs per patient in the 10TT group were £1889 (95% CI: £1522 to £2566) and 1.51 (95% CI: 1.44 to 1.58). The mean costs and QALYs for usual care were £1925 (95% CI: £1599 to £2251) and 1.51 (95% CI: 1.45 to 1.57), respectively. This generated a mean cost difference of -£36 (95%CI: -£512 to £441) and a mean QALY difference of 0.001 (95%CI: -0.080 to 0.082). The incremental NMB for 10TT versus usual care was £49 (95%CI: -£1709 to £1800) at a maximum willingness to pay for a QALY of £20,000. 10TT had a 52% probability of being cost-effective at this threshold.

For peer review only - http://bmjopen.bmj.com/site/about/guidelines.xhtml

Conclusions: Costs and QALYs for 10TT were not significantly different from usual care and therefore 10TT is as cost-effective as usual care. There was no evidence to recommend nor advice against offering 10TT to obese patients in general practices based on cost-effectiveness considerations.

Trial Registration: ISRCTN registry ISRCTN16347068

Article summary

Strengths and limitations of this study

- The analysis is based on a large multicentre randomised trial with detailed information on resource use and utility values for a median follow-up period of two years.
- Individual data on standard weight-loss interventions received by the participant were not recorded.
- The analysis took a UK NHS/PSS perspective but a wider perspective (e.g., societal) could have been taken, including costs to patients and families.
- The time horizon was two years
- Missing NHS resource use and utility data was accounted for using multiple imputation.

For peer review only - http://bmjopen.bmj.com/site/about/guidelines.xhtml

Introduction

The worldwide prevalence of obesity nearly doubled between 1980 and 2008. Based on recent estimates in the European Union, overweight affects 30-70% and obesity affects 10-30% of adults(1). In the UK 23.9 % of women aged 18 years and over are obese- the highest proportion in Europe. The proportion of UK men who are obese is 22.1%, the second highest proportion in Europe behind Malta (24.7 %)(2).

Health risks associated with obesity range from heart disease, stroke and type 2 diabetes to all cancers, gall bladder disease and mortality(3). It is estimated that health problems associated with being overweight or obese cost the National Health Service (NHS) in England more than £5 billion every year(4).

Non-surgical interventions for weight loss are available and include dietary advice, physical activity and behaviour modification. These can be accessed mainly through primary care. Despite considerable investment in such interventions, there is limited evidence about their cost-effectiveness(5-9).

Ten Top Tips (10TT) was a simple leaflet-based intervention for patients in primary care designed to help with weight loss. The underpinning theory behind 10TT was habit formation. Psychological research shows that repetition of a simple action in a consistent context leads, through associative learning, to the action being activated upon subsequent exposure to those contextual cues(10). A small pilot trial consisting of 100 people showed that those who received the Ten Top Tips leaflet lost significantly more weight than those who did not receive it(11). Although the absolute weight loss was modest, the intervention had the potential to be disseminated at minimal cost to large numbers simply by making the

For peer review only

leaflet available. In response to this a large randomised trial was undertaken. Participants were given a leaflet, which provided weight loss tips based on scientific evidence on how to turn the tips into healthy habits, during a 30-minute appointment with a practice nurse. The ten healthy tips included: keeping to meal routines; reduced fat intake; walking off weight; packing a healthy snack; looking at food labels; taking caution with portion size; standing up; thinking about drinks; focusing on food; and, not forgetting 'five a day'. The 'five a day campaign is based on recommendation from the World Health Organisation (WHO) encouraging consumption of at least 400g of fruit and vegetables a day to lower the risk of serious health problems, such as heart disease, stroke and some cancers(12). Participants were asked to record their progress in a logbook. Using routinely collected data from primary care practices, the aim of the present study was to analyse the cost and cost-effectiveness of 10TT.

Methods

Trial background

The 10TT trial was a two-arm, individually randomised, controlled trial in which 537 obese men and women were enrolled(13). Practices across England (n=14) were recruited through the MRC General Practice Research Framework (GPRF). They were located in: Wellingborough, Southampton, Bradford-on-Avon, Bromsgrove, Frome, Guisborough, Glastonbury, Ivybridge, Dunstable, Liskeard, Ledbury, New Mills, and London. The majority were located in Southern England (n=9), with three located in the Midlands and two in the North. Recruitment occurred between August 2010 and October 2011. Participants were randomly assigned to 10TT (n=267) or usual care (n=270), and followed for up to two years. The primary outcome was weight loss at three months. Secondary outcomes included BMI,

For peer review only - http://bmjopen.bmj.com/site/about/guidelines.xhtml

waist circumference, the number of people achieving a 5% reduction in weight, clinical markers for potential co-morbidities (blood pressure, total cholesterol/low-density lipoprotein and blood glucose), and maintenance of weight loss over 24 months. At three months participants in the 10TT group lost significantly more weight than those receiving usual care (mean difference in weight change=-0·87kg, 95% CI -1·47 to -0·27, p=0·004). But this effect was not maintained at 24 months (mean difference in weight change= +0·75kg (95% CI: -0.73 to 2.24)). Weight loss in the usual care group was slow in the first 6 months, but it continued until 18 months, whereas the 10TT group experienced a greater weight loss in the first 6 months, but did not lose any additional weight after this point.

Patient and Public Involvement

There was patient representation on the trial steering group for 10TT, however neither patients nor the public were involved in the economic analyses presented in this paper.

Overview of economic evaluation

We undertook a cost-utility analysis to compare the costs and outcomes associated with 10TT and usual care. The outcome measure was quality-adjusted life years (QALYs), which combines length of life and quality of life, and is consistent with National Institute for Health and Care Excellence (NICE) recommendations(14).The primary outcomes were incremental costs and effects and the incremental net monetary benefit (NMB) of 10TT versus usual care. Costs were measured from a NHS and personal social services (PSS) perspective; since PSS costs were negligible, we focused on NHS costs. These were calculated in 2013/14 UK£. The time horizon for costs and effects was two years, reflecting the trial follow-up period. Extrapolation beyond this time period was not undertaken because the within-trial analysis

For peer review only - http://bmjopen.bmj.com/site/about/guidelines.xhtml

found no evidence of a significant difference in costs or outcomes at 24 months; two years was therefore considered long enough to reflect all important differences in costs or outcomes between treatments. Costs and outcomes in the second year were discounted at 3.5%(14).

Resource use and costs

Resource use data were extracted from GP records for two years prior to randomisation and two-years post randomisation on: the number and type of contacts with the GP (surgery visit, home visit, phone call); practice nurse contacts (practice visit, home visit, phone call); dietician visits; hospital outpatient visits; hospital inpatient stay; accident and emergency (A&E) visits; and outpatient services. Data on use of drugs included dose, type and frequency of administration. All resource use data were extracted by practice nurses at participating centres from patient records.

Unit costs were attached to each resource item. The cost per visit to primary care (GP surgery visit, GP home visit, GP phone call, practice nurse visit, nurse home visit, practice nurse phone call and dietician visits) were taken from Personal Social Services Research Unit (PSSRU) estimates(15). The cost of inpatient episodes, outpatient visits and A&E visits were taken from the PSSRU and based on NHS Reference Costs(16). They included cost of medical staff, equipment, consumables, and diagnostics. The weighted average cost of inpatient stays was calculated combining the cost of inpatient long and short stay. Drug costs were obtained from the British National Formulary(17).

The cost of the 10TT intervention included the following, valued using market prices: a logbook (£2.80 per participant); time patients spent with the practice nurse to introduce the

For peer review only - http://bmjopen.bmj.com/site/about/guidelines.xhtml

programme (£20 for a 30-minute visit); wallet sized food label guidance (£0.09), and a 10TT leaflet (£0.05). The total cost of 10TT was £22.94 per participant. The cost of the food label guidance was based on the total cost of printing (£1,392) divided by the number of labels printed (15,000). Similarly, the cost of 10TT leaflets was based on a total cost of printing (£1,540) divided by the number of leaflets printed (30,000).

All practices offered standard weight-loss interventions as part of usual care. These consisted of a range of interventions including referrals to community programmes, gym prescription and referral to a dietician and/or psychologist, among others. While data were provided by practices on interventions typically offered to their patients, the uptake of these interventions was not recorded. In our base case, we made the conservative assumption (possibly biased against 10TT) that no patients received these interventions. In a sensitivity analysis, we included the costs of these interventions assuming each patient in the control group received the standard weight-loss intervention offered by the practice. Where single interventions were offered by a practice these were costed accordingly, based on the average cost of participating in Weight Watchers (assumed to cost £78.22 per patient(18)) or Slimming World (£72.62 per patient). Where multiple interventions were offered by a practice these were costed based on the average cost of participating in the Size Down Programme at a cost of £93.48 per patient(18).

Unit costs (total expenditure incurred by the NHS for one visit) for each cost component are shown in Table 1. These were multiplied by resource volume and summed across all cost components to obtain a total cost per patient.

Utilities and QALYs

For peer review only - http://bmjopen.bmj.com/site/about/guidelines.xhtml

Health utilities were based on the EuroQol EQ-5D-3L descriptive system (19, 20). This is a five dimensional questionnaire (mobility, self-care, usual activities, pain and discomfort), with three levels in each dimension (severe problems, some problems, no problems). Each EQ-5D state was converted into a single utility score based on valuations from the UK general population(21). Utility values of one represent full health, values of zero are equivalent to death, negative values represent states worse than death. The EQ-5D questionnaire was completed at baseline, three months, six months, 12 months, 18 months and 24 months. A utility profile was constructed for participants assuming a straight-line relation between their utility values at each measurement point. QALYs for every patient from baseline to two years were calculated as the area under the utility profile.

Dealing with Missing Data

There were missing data for NHS resource use and utility scores. Multivariate imputation by chained equations was used to impute missing data (22, 23). The imputation method used an iterative Markov Chain Monte Carlo (MCMC) technique to simulate from the posterior predictive distribution of missing data (24). We generated 20 imputed datasets. We imputed missing data (% of missing data) for the following variables: weight (0.2%); BMI (0.2%); waist circumference (0.6%); EQ-5D at baseline (5%), 3 months (26%), 6 months (40%), 12 months (46%) and 24 months (46%); NHS visits (GP practice (28%); GP home (31%); GP phone calls (30%); practice nurse (27%); nurse home (31%); nurse phone calls (31%); extra nurse (30%); dietician (31%); hospital inpatient stay (31%); outpatient clinic (30%); A&E visits (30%)) and other outpatient service visits (30%) (Table 1). Age, sex and study centre were included in the imputation model as additional explanatory variables. Imputations were undertaken

For peer review only - http://bmjopen.bmj.com/site/about/guidelines.xhtml

using the –mi impute mvn– command in Stata SE version 14 (Stata Corp, College Station, TX)(25).

Statistical analysis

Two sample t-tests to test for differences in cost and QALYs between the two groups were carried out for complete data. A linear regression model was used to test for differences in mean resource use, costs, EQ-5D scores at each time point, and QALYs (26, 27) using the imputed data. The incremental NMBs were calculated as the difference in mean QALYs per participant (Q) with 10TT(T) versus usual care(U) multiplied by the maximum willingness to pay for a QALY (R) minus the difference in mean cost per participant (C), i.e., incremental NMB = $(Q_T - Q_U)*R - (C_T - C_U)$. We used the cost-effectiveness threshold range recommended by NICE (£20 000 to £30 000 (14)) as the lower and upper limits of the maximum willingness to pay for a QALY (R). Negative incremental NMBs indicate that usual care is preferred on cost-effectiveness grounds and positive incremental NMBs indicate that 10TT is preferred (28).

We had initially adjusted for age, gender, practice and costs two years prior in the analysis for incremental costs. Similarly, we adjusted for age, gender, practice and baseline utility values in the analysis for incremental QALYs. There were no differences between the two groups in terms of these factors (Table 2) and therefore an unadjusted model was used.

For each of the 20 imputed datasets we ran 1000 bootstrap replications using non-parametric bootstrapping, resampling observations with replacement(29). The bootstrapped results were combined using the formula described by Briggs et al (23), to calculate the mean values for costs and utilities and the standard errors around the imputed

For peer review only - http://bmjopen.bmj.com/site/about/guidelines.xhtml

223 values. Standard errors were on based a normal distribution and used to calculate 95% CIs around point estimates.

225 The cost-effectiveness plane was used to illustrate the difference in costs and outcomes between the two groups. The probability that 10TT was cost-effective in comparison to usual care at different values of the cost-effectiveness threshold was presented in form of a cost-effectiveness acceptability curve (CEAC)(30). The CEAC was based on the proportion of bootstrap replications across all twenty imputed datasets that were below the cost-effectiveness threshold, which was varied from £0 to £50,000.

231 Several deterministic sensitivity analyses were carried out to assess the uncertainty around key components of the analysis. An analysis was undertaken based on complete cases without any imputation. We varied cost components for primary and secondary care costs and drug costs. The total costs of each component were amended by ±10%. We also conducted an analysis including the cost of standard weight-loss interventions for the usual care group.

Results

Baseline description

239 Table 3 illustrates the mean costs, two years prior to the trial. The mean cost for the usual care group was £1848 (95% CI: 1615, 2082), and £2052 (95% CI: 1756, 2349) in the 10TT group. Baseline characteristics- age, gender, weight, BMI, blood pressure and cholesterol did not differ between groups(13).

For peer review only

For peer review only - http://bmjopen.bmj.com/site/about/guidelines.xhtml

Base case

Table 1 shows mean imputed resource use by treatment group. The intervention and control groups had the same mean GP practice visits per participant over the two-year period (9.4 versus 9.4), as well as similar GP home visits (0.0 versus 0.1), GP phone contacts (1.3 versus 1.3), nurse practice visits (6.6 versus 6.9), nurse home visits (0.0 versus 0.1), inpatient episodes (0.2 versus 0.2), outpatient clinic visits (2.0 versus 2.1) and accident and emergency visits (0.4 versus 0.4). There was no statistically significant difference between groups for any category of health service resource use.

Table 3 shows the total cost per participant in each group. The non-parametric bootstrapping including imputed resource use produced mean total costs per participant in the intervention group of £1889 (95% CI: £1522 to £2566), compared to £1925 (95% CI: £1599 to £2251) in the control group. The mean difference in cost between 10TT and control group was -£36 (95%CI: -£512 to £441), which was not statistically significant (p=0.88).

Undertaking non-parametric bootstrapping after multiple imputation produced 1.51 (95% CI: 1.44 to 1.58) QALYs in the intervention group and 1.51 (95% CI: 1.45 to 1.57) in the control group. Generating a mean difference in QALYs of 0.001 (95%CI: -0.080 to 0.082) (p=0.93) (Table 4 and 5) which was not statistically significant. Hence, patients receiving 10TT accrued non-significantly lower costs and the difference in QALYs between the groups was very small and non-significant.

For peer review only - http://bmjopen.bmj.com/site/about/guidelines.xhtml

The incremental net monetary benefit for 10TT versus usual care was £49 (95% CI: -£1709 to £1800) at a maximum willingness to pay for a QALY of £20,000 and £55 (95% CI: -£2489 to £2583) at a maximum willingness to pay for a QALY of £30,000 (Table 4).

Of the 20,000 bootstrap replications produced under base case assumptions, 24% fell into south-west quadrant of the cost-effectiveness plane (10TT was less costly and less effective than usual care); 20% fell into the north-east quadrant (10TT was more costly and more effective than usual care); 25% fell into the north-west quadrant (10TT was more costly and less effective than usual care); and 31% fell into the south-east quadrant (10TT was less costly and more effective than usual care) (Figure 1). The CEAC derived from Figure 1 is illustrated in Figure 2. Under base case assumptions, 10TT has a 52% probability of being cost-effective at a willingness to pay threshold of £20,000 and 52% at a willingness to pay threshold of £30,000 (Table 4).

Sensitivity analyses

Including the cost of the standard weight-loss interventions (£87) in the usual care group the mean costs per patient in the control group increased to £2012, which was higher than the mean cost in the intervention group (£1889). The mean incremental cost difference between groups was -£122 (95% CI: -£598 to £353). The incremental net monetary benefit for 10TT versus usual care was £140 (95% CI: (-£1666 to £1902) at a maximum willingness to pay for a QALY of £20,000 and £148 (95% CI: -£2463 to £2693) at a maximum willingness to pay of £30,000. Increasing and decreasing costs by 10% did not affect the findings appreciably (Table 4).

A complete case analysis was undertaken using 163 of 537 participants with complete utility and cost data. The incremental NMB was -£889 (95% CI: -£3993 to £2253) at a maximum

For peer review only - http://bmjopen.bmj.com/site/about/guidelines.xhtml

willingness to pay for a QALY of £20,000 and -£1361 (95% CI: -£5772 to £3052) at a maximum willingness to pay for a QALY of £30,000.

Discussion

Our economic analysis of 10TT showed that this intervention had similar costs and QALYs as usual care. Sensitivity analyses showed little uncertainty in this finding. On the one hand the findings mean there is no reason to prefer 10TT or usual care on the basis of differences in quality of life or cost, or on cost-effectiveness grounds. On the other hand, this means that 10TT is as cost-effective as usual care.

We undertook a rapid review to compare our results to similar weight loss programs. We found that commercial weight loss programmes were highly prescribed amongst primary care providers and these participants lost more weight than those in self-led education programmes alone. Fuller et al(7) reported the long-term analysis of a 20 minute GP consultation versus Weight Watchers and found Weight Watchers produced a cost saving of US$47 per patient and an incremental 0.03 QALY gained per patient. Similarly, a recent evaluation in the UK of a primary care led behavioural programme(31) looked at a brief advice and self-help materials (primary care led programme) versus Weight Watchers over 12 weeks and over 52 weeks. The authors concluded that Weight Watchers was more effective over 12 weeks (-4.75kg) and 52 weeks (-6.76kg) than brief advice and self-help material (-3.26kg), at a cost of £159 per kg lost. Additionally, a primary care led program Counterweight (a nurse-delivered patient education programme) showed that nurse delivered education was less costly and more effective compared with no active intervention, producing a gain in QALYs (0.06 per participant) and cost savings of £27 per participant (9).

For peer review only

It is evident from existing literature that GPs play a crucial role in obesity prevention and weight management and are gate keepers to lifestyle weight management programmes(32). While there is evidence to suggest GP prescribed commercial programmes and/or weight-loss education is effective, further research is needed to explore the relationship between habit-formation programmes such as 10TT with commercial programmes, with the aim to determine what the long-term cost savings and QALYs produced potentially would be over a long-time horizon.

The main strength of our analysis is that it is based on a large multicentre randomised trial with detailed information on resource use and utility values for a median follow-up period of two years. There were several limitations to our study. While data were recorded in the trial on standard weight-loss interventions for obese patients at each practice, individual data on the uptake and prescribing of standard weight-loss interventions received by the participant were not recorded. This trial was also unblinded, which introduced the potential bias of GPs over prescribing standard weight-loss interventions producing a healthy user bias effect. Under base case assumptions, excluding standard weight-loss interventions, the incremental NMB of 10TT versus usual care was not significantly different from zero, and this did not change when intervention costs in the control group were included. Hence, these costs did not make an appreciable difference to our conclusions. Secondly, the analysis took a UK NHS/PSS perspective. A wider perspective (e.g., societal) could have been taken, including costs to patients and families. Third, the time horizon was limited to two years, due to the lack of difference in costs (-£36 (95%CI -£512 to £441, p-value 0.88) and benefits (0.001 (95% CI -0.080 to 0.082, p-value 0.93) at 24 months between groups. The trial did not capture QALYs from weight loss post 24 months. A longer follow-up period may

For peer review only - http://bmjopen.bmj.com/site/about/guidelines.xhtml

have allowed for the detection of long-term health benefits produced as a consequence of avoiding obesity related health conditions, such as diabetes and cardiovascular disease. However, at the time of this study little was known about the association between habit-formation and weight loss. This study has identified the importance of longer-term strategies for continued adherence of weight loss. With add-on approaches such as counselling and education on how to maintain weight loss for participants alongside 10TT, it may be possible to maintain weight loss post two-years.

Fourth, while there are various instruments available to measure health related quality of life, we administered the EQ-5D(33). We acknowledge that there may have been a potential value in using more than one measure in the trial, such as the SF-36 and Impact of Weight on Quality of Life –Lite (IWQOL-Lite) to measure differences in the positive short-term psychological effects(34). However, given the small difference between the two groups it is highly unlikely alternative measures would've produced significant differences. Finally, we were unable to access Hospital Episode Statistics (HES) data containing detailed secondary care resource use of NHS services by patients. Obtaining HES data would been problematic as this data would need to be linked to HES data by patient ID. Given the short timeframe of this trial, this was not feasible(35). Where data were available for secondary care and missing (inpatient admissions, A&E visits, outpatient clinic and other outpatient services), this was accounted for using multiple imputation, assuming these data were missing at random. It is important to note that when using such methods there is uncertainty around the non-observed value across the imputations. To account for the uncertainty around the values, we employed the non-parametric bootstrap approach to estimate the variance (a representation of uncertainty) around the true values(23). We acknowledge that though

For peer review only

For peer review only - http://bmjopen.bmj.com/site/about/guidelines.xhtml

multiple imputation is able to statistically test for error, this method can produce bias. The bias arises from the assumption that missing data in the study were ‘missing at random’. For example, the missing at random assumption may be reasonable if a variable that is predictive of missing data is included in the imputation model, but not if the variable is omitted from the model(36). However, multiple imputation offers an advantage over complete case analysis, by filling in missing values and enabling participants to be included in the analysis, where there were auxiliary variables that are strong predictors of missingness(37). Guidelines were followed to ensure the model predicted the best possible estimates; investigation of the proportion of missing data; selection of specific variables and; identification of the predictors of missingness(38). We acknowledge that as the percentage of missingness increases (in some cases more than 50%) there will be greater inefficiency in the imputed data and more chance of bias. However, in general the application of regression imputation with large missing data may be acceptable if adjustments are made for the predictions, as we have done(23).

In conclusion, the 10TT trial found that after three months’ participants receiving 10TT lost significantly more weight than those receiving usual care, but there was no difference in weight change between the two groups at 24 months. Similarly, there was no significant difference in costs and QALYs between the two arms at 24 months. There is no evidence to either recommend or advise against 10TT, based on cost-effectiveness considerations.

Acknowledgements

Weight Concern and Cancer Research UK developed the 10 Top Tips intervention and materials, and Weight Concern provided training on how to deliver the intervention. The General Practice Research Framework provided advice on the study design and was involved

For peer review only

For peer review only - http://bmjopen.bmj.com/site/about/guidelines.xhtml

in both recruiting sites and training the health professionals at each site on the trial procedures, along with providing quality control visits. Practices were located in: Wellingborough, Southampton, Bradford-on-Avon, Bromsgrove, Frome, Guisborough, Glastonbury, Ivybridge, Dunstable, Liskeard, Ledbury, New Mills, and London. The authors would like to acknowledge the substantial intellectual contribution made by Professor Jane Wardle who sadly passed away prior to publication, and is deeply missed by all of her co-authors, colleagues and students.

Contributors

NP undertook data analysis, interpreted the results, drafted, and wrote the manuscript. RJB helped design the study, undertook data collection and critically reviewed the manuscript. BL helped design the study, critically reviewed the data and manuscript. RZO helped design the study and critically reviewed the manuscript. IN helped design the study and critically reviewed the manuscript. SM undertook the data analysis, devised the analysis plan, interpreted the results and critically reviewed the manuscript.

Funding

This work was funded by the Medical Research Council (MRC) – National Prevention Research Initiative (NPRI) (UK) grant number G080202. RJB's post was funded through this grant and she is now supported by Yorkshire Cancer Research Academic Fellowship funding. SM, NP and IN are HEFCE-funded. RZO was funded by the National Institute for Health Research.

Disclaimer

The views expressed are those of the authors and not necessarily those of the funding bodies.

For peer review only

For peer review only - http://bmjopen.bmj.com/site/about/guidelines.xhtml

Competing interests

All authors have completed the ICMJE uniform disclosure form at http://www.icmje.org/coi_disclosure.pdf (available on request from the corresponding author) and declare that: NP, RJB, BL, RZO, IN and SM have nothing to disclose.

Ethics approval

This study has been approved by the South East London REC 2, 23/08/2010 (Reference number 10/H0802/59). All participants gave informed consent before taking part.

Provenance and peer review

Not commissioned; externally peer reviewed.

Data sharing statement

No additional data are available.

For peer review only

For peer review only - http://bmjopen.bmj.com/site/about/guidelines.xhtml

Table 1. Resource use, unit cost, and mean cost per participant for primary and secondary care services post randomisation

		Usual Care					10TT					Usual Care	10TT	Incremental difference	
	n	Mean	(SD)	Median	(IQR)	n	Mean	(SD)	Median	(IQR)	Unit cost (£)	Mean cost (£)	Mean cost (£)	Mean (£)	(95% CI)
**No imputation for missing values**															
GP surgery visit	204	9.4	(8.4)	7	(7.00)	182	9.7	(9.5)	7	(9.00)	45	417	425	8	(-72, 89)
GP home visit	196	0.1	(0.8)	0	(0.00)	176	0.0	(0.1)	0	(0.00)	292	37	9	-27	(-61, 7)
GP phone	199	1.3	(3.0)	0	(1.00)	179	1.3	(3.0)	0	(1.00)	27	34	34	0	(-16, 16)
GP practice nurse	206	6.9	(8.4)	4	(6.00)	184	6.8	(9.5)	4	(6.50)	40	274	268	-6	(-77, 64)
Nurse home visit	195	0.1	(1.1)	0	(0.00)	175	0.0	(0.1)	0	(0.00)	70	5	1	-4	(-16, 7)
GP practice nurse phone	197	0.5	(2.3)	0	(0.00)	176	0.4	(1.2)	0	(0.00)	10	5	4	-1	(-5, 2)
Dietician	195	0.1	(0.5)	0	(0.00)	175	0.1	(0.8)	0	(0.00)	35	4	4	0	(-4, 4)
Additional nurse visit	197	1.1	(3.1)	0	(1.00)	179	1.6	(2.6)	0	(1.00)	4	45	44	0	(-23, 22)
Inpatient admission	196	0.2	(0.7)	0	(0.00)	175	0.2	(0.7)	0	(0.00)	1713	372	387	14	(-220, 249)
Outpatient clinic	200	2.2	(3.5)	1	(3.00)	178	2.1	(3.3)	1	(3.00)	135	288	269	-20	(-110, 70)
Accident & Emergency visit	199	0.4	(1.2)	0	(0.00)	175	0.4	(0.9)	0	(1.00)	177	65	67	2	(-36, 39)
Other outpatient services	194	1.0	(1.8)	0	(1.00)	179	1.0	(2.3)	0	(1.00)	135	124	129	5	(-48, 58)
**Missing values imputed***			**SE**					**SE**							
GP surgery visit	270	9.4	(0.6)	9		267	9.4	(0.7)	9		45	419	420	1	(-79,81)
GP home visit	270	0.1	(0.5)	0		267	0.0	(0.2)	0		292	36	13	-23	(-58, 12)
GP phone	270	1.3	(0.2)	1		267	1.3	(0.2)	1		27	35	33	-2	(-19, 14)
GP practice nurse	270	6.9	(0.6)	6		267	6.6	(0.7)	6		40	274	266	-9	(-75, 57)
Nurse home visit	270	0.1	(1.0)	0		267	0.0	(0.1)	0		70	6	2	-4	(-16, 7)
GP practice nurse phone	270	0.5	(0.6)	0		267	0.4	(0.1)	0		10	5	4	-1	(-5, 2)
Dietician	270	0.1	(0.0)	0		267	0.1	(0.6)	0		35	4	4	0	(-4, 4)

For peer review only - http://bmjopen.bmj.com/site/about/guidelines.xhtml

Additional nurse visit	270	1.2	(0.2)	1		267	1.8	(0.2)	0		4	46	47	0	(-23, 23)
Inpatient admission	270	0.2	(0.1)	0		267	0.2	(0.1)	0		1713	373	379	6	(-238, 251)
Outpatient clinic	270	2.1	(0.3)	2		267	2.0	(0.2)	0		135	287	266	-22	(-106, 63)
Accident & Emergency visit	270	0.4	(0.9)	0		267	0.4	(0.7)	0		177	67	69	2	(-38, 42)
Other outpatient services	270	1	(0.1)	1		267	1	(0.2)	0		135	122	129	6	(-52, 65)
**Weight loss interventions**															
10TT		-	-	-	-		-	-	-	-	23	-	-		
Weight Watchers		-	-	-	-		-	-	-	-	78	-	-		
Slimming World		-	-	-	-		-	-	-	-	93	-	-		
Size Down Programme		-	-	-	-		-	-	-	-	73	-	-		

GP = general practitioner. SD = standard deviation for non-imputed data. SE = standard error for imputed data. CI = confidence interval. IQR = inter-quartile range. Costs are in 2013/14 UK£. *Missing values imputed using multiple imputation with 20 imputed datasets (see text).

Table 2. Demographics at baseline

	Usual care			10TT			
	n	Mean*	SD	n	Mean	SD	P-value≠
Age	270	58	12.61	267	57	12.88	0.47
Male	95	35.2	-	89	33.3	-	-
Female	175	64.8	-	178	66.7	-	-
Prior costs	270	1848	1948	267	2052	2461	0.29
Weight (kg)	269	101	17.46	267	100	16.98	0.59
Body Mass Index (BMI)	269	36.59	5.72	267	36.18	4.71	0.36
Baseline EQ-5D	257	0.76	0.24	255	0.74	0.27	0.41

SD = standard deviation. * Figures sex based on the mean proportion (%) in each group. ≠From independent t-test

For peer review only - http://bmjopen.bmj.com/site/about/guidelines.xhtml

Table 3. Cost description two years prior and post randomisation

	Usual care			10TT			Incremental difference	
	n	Mean	SD	n	Mean	SD	Mean (£)	(95% CI)
**Cost, two years prior**								
Cost of primary and secondary care contacts	270	1516	1694	267	1735	2186	219	(-112, 550)
Drugs costs	270	332	748	267	317	624	-15	(-132, 102)
Total cost	270	1848	1948	267	2052	2461	204	(-172, 580)
**Costs after randomisation (imputed)***			**SE**			**SE**		
Cost of primary and secondary care contacts	270	1675	150	267	1631	169	-45	(-477, 387)
Drugs costs	270	249	44	267	236	68	-13	(-172, 146)
Intervention costs	-	-	-	267	23	-	-	-
Total cost	270	1925	165	267	1889	186	-36	(-512, 441)

SD = standard deviation for non-imputed data. SE = standard error for imputed data. CI = confidence interval

For peer review only

Table 4. Incremental cost-effectiveness of 10TT versus usual care

	Incremental cost		Incremental QALYs		Incremental net monetary benefit (NMB)				Probability cost-effective	
					£20,000		£30,000			
	Mean (£)	(95% CI)	Mean	(95% CI)	Mean (£)	(95% CI)	Mean (£)	(95% CI)	£20,000	£30,000
Base case*	-36	(-512, 441)	0.001	(-0.080, 0.082)	49	(-1709, 1800)	55	(-2489, 2583)	52%	52%
Base case with control group intervention cost~	-122	(-598, 353)	0.001	(-0.080, 0.082)	140	(-1666, 1902)	148	(-2463, 2693)	56%	54%
Complete case analysis§	-66	(-907, 774)	-0.047	(-0.180, 0.086)	-889	(-3993, 2253)	-1361	(-5772, 3052)	29%	28%
Primary and secondary care cost increased #	-40	(-556, 475)	0.001	(-0.080, 0.081)	48	(-1743, 1851)	53	(-2520, 2623)	52%	51%
Drug cost increased #	23	(-396, 442)	0.001	(-0.080, 0.077)	-14	(-1734, 1716)	-9	(-2511, 2510)	49%	50%
All costs increased #	-39	(-554, 476)	0.001	(-0.080, 0.082)	43	(-1756, 1851)	46	(-2537, 2641)	52%	51%
Primary and secondary care cost decreased ¥	-31	(-463, 401)	0.001	(-0.080, 0.081)	54	(-1683, 1804)	64	(-2453, 2587)	52%	52%
Drug costs decreased ¥	26	(-387, 438)	0.001	(-0.080, 0.081)	0	(-1741, 1727)	11	(-2530, 2520)	50%	51%
All costs decreased ¥	-32	(-460, 395)	0.001	(-0.080, 0.081)	50	(-1682, 1811)	59	(-2458, 2599)	52%	52%

*N= 537. Data include values imputed using multiple imputation (see text). The 95% confidence intervals were derived from 1000 bootstrap replications of each of the 20 imputed datasets (see text). The incremental net monetary benefit and the probability 10TT is cost-effective are based on the QALYs gained and incremental costs and calculated at a maximum willingness to pay for a QALY of £20,000 and £30,000.

~ As for the base case analysis. Standard weight-loss interventions (Weight Watchers, Slimming World, Size Down programme) are included in the control group.

§As for the base case analysis. No multiple imputation of missing values and the 95% confidence intervals were derived from 1000 bootstrap replications of a single dataset containing the n=72 participants in the 10TT group and n=91 participants in the usual care group with no missing values. The costs of interventions in the control group (Weight Watchers, Slimming World, Size Down programme) were excluded.

# As for the base case analysis. Costs were increased by 10% for 10TT.

¥ As for the base case analysis. Costs were decreased by 10% for 10TT.

For peer review only - http://bmjopen.bmj.com/site/about/guidelines.xhtml

For peer review only

Table 5. Quality Adjusted Life Years (QALYs) per patient

	Usual Care				10TT				Incremental difference	
	**n**	**Mean**	**SD**	**95% CI**	**n**	**Mean**	**SD**	**95% CI**	**Mean**	**(95% CI)**
**No imputation for missing values**										
Utility at 3 months	206	0.77	0.23	(0.74, 0.80)	190	0.77	0.26	(0.73, 0.80)	0.00	(-0.05, 0.05)
Utility at 6 months	163	0.76	0.24	(0.72, 0.80)	159	0.78	0.24	(0.74, 0.82)	0.02	(-0.03, 0.07)
Utility at 12 months	151	0.77	0.24	(0.73, 0.81)	138	0.74	0.27	(0.70, 0.79)	-0.03	(-0.09, 0.03)
Utility at 18 months	138	0.75	0.25	(0.71, 0.79)	124	0.74	0.27	(0.69, 0.79)	-0.01	(-0.07, 0.05)
Utility at 24 months	157	0.76	0.25	(0.73, 0.80)	131	0.76	0.26	(0.72, 0.81)	0.00	(-0.06, 0.06)
**Discounted QALYs (24 months)**	92	1.52	0.40	(1.44, 1.61)	73	1.47	0.47	(1.36, 1.58)	-0.05	(-0.18, 0.08)
**Missing values imputed***			**SE**				**SE**			
Utility at 3 months	270	0.77	0.016	(0.73, 0.80)	267	0.77	0.017	(0.73, 0.80)	0.00	(-0.05, 0.05)
Utility at 6 months	270	0.76	0.016	(0.73, 0.79)	267	0.78	0.018	(0.73, 0.81)	0.01	(-0.03, 0.06)
Utility at 12 months	270	0.76	0.019	(0.73, 0.80)	267	0.75	0.020	(0.71, 0.80)	-0.01	(-0.06, 0.04)
Utility at 18 months	270	0.76	0.019	(0.72, 0.79)	267	0.75	0.020	(0.71, 0.79)	-0.01	(-0.05, 0.04)
Utility at 24 months	270	0.76	0.019	(0.72, 0.79)	267	0.76	0.018	(0.72, 0.79)	0.00	(-0.05, 0.05)
**Discounted QALYs (24 months)**	270	1.51	0.030	(1.45, 1.57)	267	1.51	0.033	(1.44, 1.58)	0.00	(-0.08, 0.08)

QALY = quality-adjusted life year. SD = standard deviation for non-imputed data. SE = standard error for imputed data. CI = confidence interval. Costs are in 2013/14 UK£. CI were based on non-parametric bootstrapping for observed data and imputed data. * Missing values imputed using multiple imputation with 20 imputed datasets (see text).

For peer review only

For peer review only - http://bmjopen.bmj.com/site/about/guidelines.xhtml

Figure 1. Cost-Effectiveness plane, base case analysis assumptions. Based on 20,000 bootstrap replications, from 20 imputed datasets.

Figure 2. Cost-effectiveness acceptability curve showing the probability that 10TT is cost-effective vs usual care at different values of the maximum willingness to pay for a QALY (base-case assumptions)

QALY = quality adjusted life year

For peer review only

For peer review only - http://bmjopen.bmj.com/site/about/guidelines.xhtml

References

1. http://www.euro.who.int/en/health-topics/noncommunicable-diseases/obesity/data-and-statistics.

2. http://ec.europa.eu/eurostat/statistics-explained/index.php/Overweight_and_obesity_-_BMI_statistics.

3. Guh DP, Zhang W, Bansback N, Amarsi Z, Birmingham CL, Anis AH. The incidence of co-morbidities related to obesity and overweight: a systematic review and meta-analysis. BMC public health. 2009;9:88.

4. Reducing obesity and improving diet. https://www.gov.uk/government/policies/reducing-obesity-and-improving-diet. Accessed 07.03.15.

5. Roux L, Kuntz KM, Donaldson C, Goldie SJ. Economic evaluation of weight loss interventions in overweight and obese women. Obesity (Silver Spring, Md). 2006;14(6):1093-106.

6. Fuller NR, Carter H, Schofield D, Hauner H, Jebb SA, Colagiuri S, et al. Cost effectiveness of primary care referral to a commercial provider for weight loss treatment, relative to standard care: a modelled lifetime analysis. Int J Obes. 2014;38(8):1104-9.

7. Fuller NR, Colagiuri S, Schofield D, Olson AD, Shrestha R, Holzapfel C, et al. A within-trial cost-effectiveness analysis of primary care referral to a commercial provider for weight loss treatment, relative to standard care--an international randomised controlled trial. International journal of obesity (2005). 2013;37.

8. Tsai AG, Wadden TA, Volger S, Sarwer DB, Vetter M, Kumanyika S, et al. Cost-effectiveness of a primary care intervention to treat obesity. International journal of obesity (2005). 2013;37 Suppl 1:S31-7.

9. Trueman P, Haynes SM, Felicity Lyons G, Louise McCombie E, McQuigg MS, Mongia S, et al. Long-term cost-effectiveness of weight management in primary care. International journal of clinical practice. 2010;64(6):775-83.

10. Gardner B, Lally P, Wardle J. Making health habitual: the psychology of 'habit-formation' and general practice. The British journal of general practice : the journal of the Royal College of General Practitioners. 2012;62(605):664-6.

11. Lally P, Chipperfield A, Wardle J. Healthy habits: efficacy of simple advice on weight control based on a habit-formation model. Int J Obes. 2007;32(4):700-7.

12. http://www.nhs.uk/Livewell/5ADAY/Pages/Why5ADAY.aspx.

13. Beeken RJ, Leurent B, Vickerstaff V, Wilson R, Croker H, Morris S, et al. A brief intervention for weight control based on habit-formation theory delivered through primary care: results from a randomised controlled trial. International journal of obesity (2005). 2017.

14. National Institute for Health and Care Excellence (NICE). Guide to the methods of technology appraisal. London: NICE; 2013.

15. Curtis L. Unit costs of health and social care 2013. Kent: Personal Social Services Research Unit; 2013.

16. Department of Health. National Schedule of Reference Costs- 2012-2013. NHS trusts and NHS foundation trusts. London Department of Health; 2012.

17. Joint Formulary Committee . British National Formulary, 68th edition. London: BMJ Group and Pharmaceutical Press; 2013 [Available from: https://www.medicinescomplete.com/mc/bnf/current/.

18. Jolly K, Lewis A, Beach J, Denley J, Adab P, Deeks JJ, et al. Comparison of range of commercial or primary care led weight reduction programmes with minimal intervention control for weight loss in obesity: lighten Up randomised controlled trial. BMJ (Clinical research ed). 2011;343:d6500.

For peer review only

For peer review only - http://bmjopen.bmj.com/site/about/guidelines.xhtml

19. Rabin R, de Charro F. EQ-5D: a measure of health status from the EuroQol Group. Annals of medicine. 2001;33(5):337-43.
20. Brooks R. EuroQol: the current state of play. Health policy (Amsterdam, Netherlands). 1996;37(1):53-72.
21. Dolan P. Modeling valuations for EuroQol health states. Medical care. 1997;35(11):1095-108.
22. Petrou S, Dakin H, Abangma G, Benge S, Williamson I. Cost-utility analysis of topical intranasal steroids for otitis media with effusion based on evidence from the GNOME trial. Value in health : the journal of the International Society for Pharmacoeconomics and Outcomes Research. 2010;13(5):543-51.
23. Briggs A, Clark T, Wolstenholme J, Clarke P. Missing... presumed at random: cost-analysis of incomplete data. Health economics. 2003;12(5):377-92.
24. Royston P Multiple imputation of missing values: update of ice. Stata J. 2005: 527-36.
25. StataCorp. 2015. Stata Statistical Software: Release 14. College Station, TX: StataCorp LP. .
26. Sinharay S, Stern HS, Russell D. The use of multiple imputation for the analysis of missing data. Psychological methods. 2001;6(4):317-29.
27. Yang X, Belin TR, Boscardin WJ. Imputation and variable selection in linear regression models with missing covariates. Biometrics. 2005;61(2):498-506.
28. Zethraeus N, Johannesson M, Jonsson B, Lothgren M, Tambour M. Advantages of using the net-benefit approach for analysing uncertainty in economic evaluation studies. PharmacoEconomics. 2003;21(1):39-48.
29. Pasta DJ, Taylor JL, Henning JM. Probabilistic sensitivity analysis incorporating the bootstrap: an example comparing treatments for the eradication of Helicobacter pylori. Medical decision making : an international journal of the Society for Medical Decision Making. 1999;19(3):353-63.
30. Briggs AH, Gray AM. Handling uncertainty when performing economic evaluation of healthcare interventions. Health technology assessment (Winchester, England). 1999;3(2):1-134.
31. Ahern AL, Wheeler GM, Aveyard P, Boyland EJ, Halford JCG, Mander AP, et al. Extended and standard duration weight-loss programme referrals for adults in primary care (WRAP): a randomised controlled trial. Lancet. 2017;389(10085):2214-25.
32. National Institute for Health and Care Excellence. Weight management: lifetsyle services for overweight or obesity adults, NICE guideline. London: NICE, 2014.
33. Kolotkin RL, Norquist JM, Crosby RD, Suryawanshi S, Teixeira PJ, Heymsfield SB, et al. One-year health-related quality of life outcomes in weight loss trial participants: comparison of three measures. Health and quality of life outcomes. 2009;7:53.
34. Peckmezian T, Hay P. A systematic review and narrative synthesis of interventions for uncomplicated obesity: weight loss, well-being and impact on eating disorders. Journal of eating disorders. 2017;5:15.
35. Herrett E, Shah AD, Boggon R, Denaxas S, Smeeth L, van Staa T, et al. Completeness and diagnostic validity of recording acute myocardial infarction events in primary care, hospital care, disease registry, and national mortality records: cohort study. BMJ (Clinical research ed). 2013;346:f2350.
36. Sterne JA, White IR, Carlin JB, Spratt M, Royston P, Kenward MG, et al. Multiple imputation for missing data in epidemiological and clinical research: potential and pitfalls. BMJ (Clinical research ed). 2009;338:b2393.
37. Lee KJ, Simpson JA. Introduction to multiple imputation for dealing with missing data. Respirology (Carlton, Vic). 2014;19(2):162-7.
38. Hayati Rezvan P, Lee KJ, Simpson JA. The rise of multiple imputation: a review of the reporting and implementation of the method in medical research. BMC medical research methodology. 2015;15:30.

For peer review only

For peer review only - http://bmjopen.bmj.com/site/about/guidelines.xhtml

139

For peer review only

For peer review only - http://bmjopen.bmj.com/site/about/guidelines.xhtml

Figure 1. Cost-Effectiveness plane, base case analysis assumptions. Based on 20,000 bootstrap replications, from 20 imputed datasets.

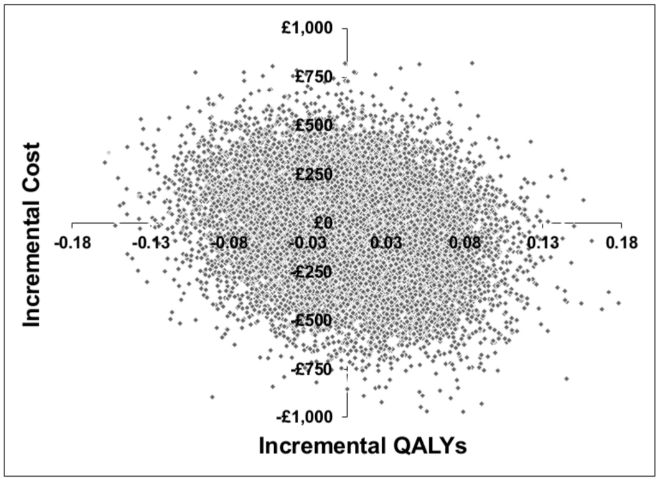

1

Figure 1. Cost-Effectiveness plane, base case analysis assumptions. Based on 20,000 bootstrap replications, from 20 imputed datasets.

296x209mm (300 x 300 DPI)

ew only

Figure 2. Cost-effectiveness acceptability curve showing the probability that 10TT is cost-effective vs usual care at different values of the maximum willingness to pay for a QALY (base-case assumptions)

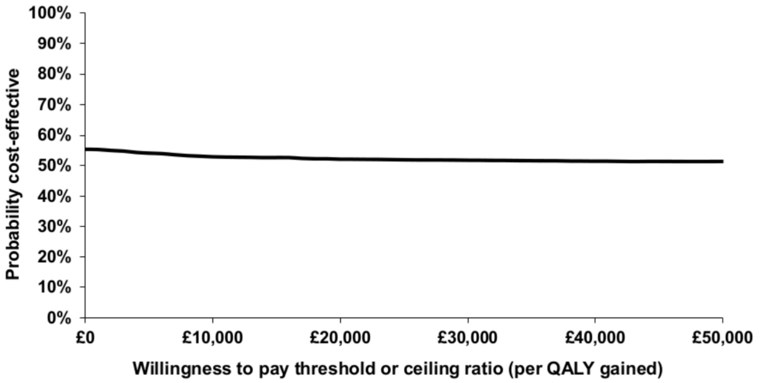

QALY = quality adjusted life year

Figure 2. Cost-effectiveness acceptability curve showing the probability that 10TT is cost-effective vs usual care at different values of the maximum willingness to pay for a QALY (base-case assumptions)

296x209mm (300 x 300 DPI)

BMJ

BMJ 2013;346:f1049 doi: 10.1136/bmj.f1049 (Published 25 March 2013) Page 1 of 6

RESEARCH METHODS & REPORTING

Consolidated Health Economic Evaluation Reporting Standards (CHEERS) statement

OPEN ACCESS

Don Husereau *senior associate; adjunct professor of medicine; senior scientist*[1 2 3], Michael Drummond *co-editor-in-chief, Value in Health; professor of health economics*[4], Stavros Petrou *professor of health economics*[5], Chris Carswell *editor*[6], David Moher *senior scientist*[7], Dan Greenberg *associate professor and chairman; visiting assistant professor*[8 9], Federico Augustovski *director; professor of public health*[10 11], Andrew H Briggs *William R Lindsay chair of health economics, health economics and health technology assessment*[12], Josephine Mauskopf *vice president of health economics*[13], Elizabeth Loder *chief of division; clinical epidemiology editor, BMJ*[14], on behalf of the CHEERS Task Force

[1]Institute of Health Economics, Edmonton, Canada; [2]Department of Epidemiology and Community Medicine. University of Ottawa, Ottawa, Canada; [3]University for Health Sciences, Medical Informatics and Technology, Hall in Tirol, Austria; [4]Centre for Health Economics, University of York, York, UK; [5]Warwick Medical School, University of Warwick, Coventry, UK; [6]Pharmacoeconomics, Adis International, Auckland, New Zealand; [7]Clinical Epidemiology Program, Ottawa Hospital Research Institute, Ottawa, Canada; [8]Department of Health Systems Management, Faculty of Health Sciences, Ben-Gurion University of the Negev, Beer-Sheva, Israel; [9]Center for the Evaluation of Value and Risk in Health, Tufts Medical Center, Boston MA, USA; [10]Health Economic Evaluation and Technology Assessment, Institute for Clinical Effectiveness and Health Policy (IECS), Buenos Aires, Argentina; [11]Universidad de Buenos Aires, Buenos Aires, Argentina; [12]Institute of Health and Wellbeing, University of Glasgow, Glasgow, Scotland; [13]RTI Health Solutions, Research Triangle Park NC, USA; [14]Division of Headache and Pain, Brigham and Women's/Faulkner Neurology, Faulkner Hospital, Boston MA, USA

Abstract

Economic evaluations of health interventions pose a particular challenge for reporting. There is also a need to consolidate and update existing guidelines and promote their use in a user friendly manner. The Consolidated Health Economic Evaluation Reporting Standards (CHEERS) statement is an attempt to consolidate and update previous health economic evaluation guidelines efforts into one current, useful reporting guidance. The primary audiences for the CHEERS statement are researchers reporting economic evaluations and the editors and peer reviewers assessing them for publication.

The need for new reporting guidance was identified by a survey of medical editors. A list of possible items based on a systematic review was created. A two round, modified Delphi panel consisting of representatives from academia, clinical practice, industry, government, and the editorial community was conducted. Out of 44 candidate items, 24 items and accompanying recommendations were developed. The recommendations are contained in a user friendly, 24 item checklist. A copy of the statement, accompanying checklist, and this report can be found on the ISPOR Health Economic Evaluations Publication Guidelines Task Force website (www.ispor.org/TaskForces/EconomicPubGuidelines.asp).

We hope CHEERS will lead to better reporting, and ultimately, better health decisions. To facilitate dissemination and uptake, the CHEERS statement is being co-published across 10 health economics and medical journals. We encourage other journals and groups, to endorse CHEERS. The author team plans to review the checklist for an update in five years.

Health economic evaluations are conducted to inform resource allocation decisions. Economic evaluation has been defined as "the comparative analysis of alternative courses of action in terms of both their costs and their consequences."[1] All economic evaluations assess costs, but approaches to measuring and valuing the consequences of health interventions may differ (see box).

Economic evaluations have been widely applied in health policy, including the assessment of prevention programmes (such as vaccination, screening, and health promotion), diagnostics, treatment interventions (such as drugs and surgical procedures), organisation of care, and rehabilitation. Economic evaluations are increasingly being used for decision making and are an important component of programmes for health technology assessment internationally.[2]

Correspondence to: D Husereau, 879 Winnington Ave, Ottawa, ON K2B 5C4, Canada donh@donhusereau.com

For peer review only - http://bmjopen.bmj.com/site/about/guidelines.xhtml

No commercial reuse: See rights and reprints http://www.bmj.com/permissions Subscribe: http://www.bmj.com/subscribe

BMJ 2013;346:f1049 doi: 10.1136/bmj.f1049 (Published 25 March 2013)

Forms of economic evaluation[1]

Specific forms of analysis reflect different approaches to evaluating the consequences of health interventions. Health consequences may be estimated from a single analytical (experimental or non-experimental) study, a synthesis of studies, mathematical modelling, or a combination of modelling and study information.

Cost consequences analysis examines costs and consequences without attempting to isolate a single consequence or aggregate consequences into a single measure

Cost minimisation analysis (CMA)—The consequences of compared interventions are required to be equivalent, and only relative costs are compared

Cost effectiveness analysis (CEA) measures consequences in natural units, such as life years gained, disability days avoided, or cases detected. In a variant of CEA, often called cost utility analysis, consequences are measured in terms of preference-based measures of health, such as quality adjusted life years or disability adjusted life years.

Cost benefit analysis—Consequences are valued in monetary units.

Readers should be aware that an economic evaluation might be referred to as a "cost effectiveness analysis" or "cost benefit analysis" even if it does not strictly adhere to the definitions above. Multiple forms may also exist within a single evaluation. Different forms of analysis provide unique advantages or disadvantages for decision making. The Consolidated Health Economic Evaluation Reporting Standards (CHEERS) statement can be used with any form of economic evaluation.

Reporting challenges and shortcomings in health economic evaluations

Compared with clinical studies, which report the consequences of an intervention only, economic evaluations require more reporting space for additional items, such as resource use, costs, preference related information, and cost effectiveness results. This creates challenges for editors, reviewers, and those who wish to scrutinise a study's findings.[3] There is evidence that the quality of reporting of economic evaluations varies widely and could potentially benefit from improved quality assurance mechanisms.[4 5]

With the increasing number of publications available, and opportunity costs from decisions based on misleading study findings, transparency and clarity in reporting are important. In addition, outside of economic evaluations conducted alongside clinical trials, there are no widespread mechanisms for warehousing economic evaluation data to allow for independent interrogation, such as ethics review proceedings, regulator dossiers, or study registries. Instead, independent analysis may rely on the record keeping of individual investigators.

Even if measures to promote transparency exist, such as registries, biomedical journal editors have increasingly promoted and endorsed the use of reporting guidelines. Endorsement of guidelines by journals for randomised controlled trials has been shown to improve reporting.[6] The combination of the risk of making costly decisions due to poor reporting with the lack of mechanisms that promote accountability makes transparency in reporting economic evaluations especially important and a primary concern among journal editors and decision makers.[3 7]

Aim and scope

The aim of the Consolidated Health Economic Evaluation Reporting Standards (CHEERS) statement is to provide recommendations, in the form of a checklist, to optimise reporting of health economic evaluations. The need for a contemporary reporting guidance for economic evaluations was recently identified by researchers and biomedical journal editors.[8] The CHEERS statement attempts to consolidate and update previous efforts [9-20] into a single useful reporting guidance.

The primary audiences for the CHEERS statement are researchers reporting economic evaluations and the editors and peer reviewers evaluating their publication potential. We hope the statement (which consists of a 24 item checklist and accompanying recommendations on the minimum amount of information to be included when reporting economic evaluations) is a useful and practical tool for these audiences and will improve reporting and, in turn, health and healthcare decisions. To best understand and apply the recommendations contained within the statement, we encourage readers to access the Explanation and Elaboration Report.[21]

Development of the CHEERS statement

The statement was developed by a task force supported by the International Society for Pharmacoeconomics and Outcomes Research (ISPOR), as part of a broader initiative to facilitate and encourage the interchange of expert knowledge and develop best practices. The CHEERS Task Force members were chosen by the chair of the task force primarily based on their longstanding academic expertise and contribution to the multidisciplinary field of health economic evaluation. In addition to four members of the task force with doctorates in economics and its sub-discipline of health economics (AHB, MD, JM, SP), members included experts in health technology assessment and decision making (FA, AHB, DH, MD, JM) and in clinical epidemiology and biostatistics (AHB, EL, DM), those in active clinical practice (EL, FA), and those with previous experience in reporting guideline development (MD, DM). All members are researchers in applied health and health policy, with five members currently serving as editors for journals in the field (AHB, CC, MD, DG, EL).

The CHEERS Task Force followed current recommendations for developing reporting guidelines.[22] Briefly, the need for new guidance was first identified through a survey of members of the World Association of Medical Editors. Of the 6% (55/965) who responded, 91% (n=50) indicated they would use a standard if one were widely available.[8] Next, published checklists or guidance documents related to reporting economic evaluations were identified from a systematic review and survey of task force members.[23] Both of these activities were used to create a preliminary list of items to include when reporting economic evaluations. Recommendations of the minimum set of reporting items were then developed through a modified Delphi panel process. Forty eight individuals identified by the task force with broad geographical representation and representing academia, biomedical journal editors, the pharmaceutical industry, government decision makers, and those in clinical practice were invited to participate. Thirty eight agreed to participate. Participants were asked to score importance on a Likert scale and the average scores, weighted by each individual's confidence in ability to score, were then used to rank items. A cut-off point was applied to the ranked list to determine the minimum number of items important for reporting.

The CHEERS statement recommendations have been independently reviewed and subsequently revised by task force members. The recommendations are entirely those of the task

For peer review only - http://bmjopen.bmj.com/site/about/guidelines.xhtml

No commercial reuse: See rights and reprints http://www.bmj.com/permissions Subscribe: http://www.bmj.com/subscribe

BMJ 2013;346:f1049 doi: 10.1136/bmj.f1049 (Published 25 March 2013)

force—the sponsors of the study had no role in study design, data analysis, data interpretation, or writing of the final recommendations. A more complete description of the methods and findings of the Delphi panel are found in the larger explanation and elaboration document.[21]

Checklist items

The final recommendations are subdivided into six main categories: (1) title and abstract; (2) introduction; (3) methods; (4) results; (5) discussion; and (6) other. The recommendations are contained in a user friendly, 24 item checklist (table) to aid users who wish to follow them. A copy of the checklist can also be found on the CHEERS Task Force website. (www.ispor.org/TaskForces/EconomicPubGuidelines.asp). In order to encourage dissemination and use of a single international standard for reporting, the task force approached 14 journals identified as either the largest publishers of economic evaluations or widely read by the medical and research community. Thirteen journals responded, and 10 expressed their ability and interest in endorsing this guidance. The CHEERS statement is being simultaneously published in *BMC Medicine, BMJ, BJOG: An International Journal of Obstetrics and Gynaecology, Clinical Therapeutics, Cost Effectiveness and Resource Allocation, The European Journal of Health Economics, International Journal of Technology Assessment in Health Care, Journal of Medical Economics, Pharmacoeconomics*, and *Value in Health*. To facilitate wider dissemination and uptake of this reporting guidance, we encourage other journals and groups to consider endorsing CHEERS.

Concluding remarks

As the number of published health economic evaluations continues to grow, we believe more transparent and complete reporting of methods and findings will be increasingly important to facilitate interpretation and comparison of studies. We hope the CHEERS statement, consisting of recommendations in a 24 item checklist, will be viewed as an effective consolidation and update of previous efforts and serve as a starting point for standard reporting going forward.

We believe the CHEERS statement represents a considerable expansion over previous efforts. The strength of our approach is that it was developed in accordance with current recommendations for the development of reporting guidelines, using an international and multidisciplinary team of editors and content experts in economic evaluation and reporting.[22] Similar to the approach taken with other widely accepted guidelines, we have defined a minimum set of criteria though a modified Delphi technique and have translated these into recommendations, an explanatory document with explanations, and a checklist. Unlike some previous reporting guidance for economic evaluation, we have also made every effort to be neutral about the conduct of economic evaluation, allowing analysts the freedom to choose different methods.

There may be several limitations to our approach. A larger Delphi panel with a different composition could have led to a different final set of recommendations.[24] Some less common approaches and contexts (such as public health, developing countries, and system dynamic models) for conducting health economic evaluation may not be well represented by our sample of experts. Additionally, like many Delphi panel processes, we based decisions to reject or accept criteria on arbitrary levels of importance. However, we feel the group recruited to create the statement is sufficiently knowledgeable of the more common applications of economic evaluation, and the rules used to select criteria were created a priori and are consistent with previous efforts.

We believe it will be important to evaluate the effects of implementation of this statement and checklist on reporting in future economic evaluations. As methods for the conduct of economic evaluation continue to evolve, it will also be important to revisit or extend the guidance. The CHEERS Task Force feels that this statement should be reviewed for updating five years from its release.

Competing interests: All authors have completed the ICMJE uniform disclosure form at www.icmje.org/coi_disclosure.pdf and declare: FA served as board member for the study funder; FA, AHB, CC, MD, DG, DH, EL, JM, and SP were provided support for travel to a face-to-face meeting to discuss the contents of the report; FA and MD have received payment from the study sponsor for serving as co-editors for *Value in Health*; no other relationships or activities that could appear to have influenced the submitted work.

Elizabeth Loder—*BMJ* clinical epidemiology editor—played no part in the peer review or decision making of this paper at the editorial level, and contributed solely as an author.

The International Society for Pharmacoeconomics and Outcomes Research (ISPOR) Health Economic Evaluation Publication Guidelines—CHEERS Task Force acknowledge the support of Elizabeth Molsen; Donna Rindress, who provided the initial leadership for this effort; and the reviewers and Delphi panel participants, who are named in the larger explanation and elaboration document and on the CHEERS Task Force website.

Contributors: All authors provided a substantial contribution to the design and interpretation of the protocol and guidance, as well as writing sections of drafts, revising based on comments received, and approving the final version. DH conducted the analysis of Delphi panel responses, drafted and revised the protocol and the drafts of this paper, and is the guarantor for the study.

Funding: All CHEERS Task Force members are volunteers. Support for this initiative was provided by the International Society for Pharmacoeconomics and Outcomes Research.

1 Drummond MF, Sculpher MJ, Torrance G, O'Brien J, Stoddart GL. *Methods for the economic evaluation of health care programmes*. 3rd ed. Oxford University Press, 2005.
2 Drummond MF, Schwartz JS, Jönsson B, Luce BR, Neumann PJ, Siebert U, et al. Key principles for the improved conduct of health technology assessments for resource allocation decisions. *Int J Technol Assess Health Care* 2008;24:244-58.
3 Rennie D, Luft HS. Pharmacoeconomic analyses. *JAMA* 2000;283:2158-60.
4 Neumann PJ, Stone PW, Chapman RH, Sandberg EA, Bell CM. The quality of reporting in published cost-utility analyses, 1976-1997. *Ann Intern Med* 2000;132:964.
5 Rosen AB, Greenberg D, Stone PW, Olchanski NV, Neumann PJ. Quality of abstracts of papers reporting original cost-effectiveness analyses. *Med Decis Making* 2005;25:424-8.
6 Turner L, Shamseer L, Altman DG, Schulz KF, Moher D. Does use of the CONSORT Statement impact the completeness of reporting of randomised controlled trials published in medical journals? A Cochrane review. *Syst Rev* 2012;1:60.
7 Drummond MF. A reappraisal of economic evaluation of pharmaceuticals. Science or marketing? *Pharmacoeconomics* 1998;14:1-9.
8 McGhan WF, Al M, Doshi JA, Kamae I, Marx SE, Rindress D. The ISPOR Good Practices for Quality Improvement of Cost-Effectiveness Research Task Force report. *Value Health* 2009;12:1086-99.
9 Task Force on Principles for Economic Analysis of Health Care Technology. Economic analysis of health care technology. A report on principles. *Ann Intern Med* 1995;123:61-70.
10 Drummond MF, Jefferson TO. Guidelines for authors and peer reviewers of economic submissions to the BMJ. *BMJ* 1996;313:275-83.
11 Gold MR. *Cost-effectiveness in health and medicine*. Oxford University Press, 1996.
12 Siegel JE, Weinstein MC, Russell LB, Gold MR. Recommendations for reporting cost-effectiveness analyses. Panel on Cost-Effectiveness in Health and Medicine. *JAMA* 1996;276:1339-41.
13 Nuijten MJ, Pronk MH, Brorens MJ, Hekster YA, Lockefeer JH, de Smet PA, et al. Reporting format for economic evaluation: Part II: Focus on modelling studies. *Pharmacoeconomics* 1998;14:259-68.
14 Vintzileos AM, Beazoglou T. Design, execution, interpretation, and reporting of economic evaluation studies in obstetrics. *Am J Obstet Gynecol* 2004;191:1070-6.
15 Drummond M, Manca A, Sculpher M. Increasing the generalizability of economic evaluations: recommendations for the design, analysis, and reporting of studies. *Int J Technol Assess Health Care* 2005;21:165-71.

For peer review only - http://bmjopen.bmj.com/site/about/guidelines.xhtml

BMJ 2013;346:f1049 doi: 10.1136/bmj.f1049 (Published 25 March 2013) Page 4 of 6

16 Ramsey S, Willke R, Briggs A, Brown R, Buxton M, Chawla A, et al. Good research practices for cost-effectiveness analysis alongside clinical trials: the ISPOR RCT-CEA Task Force report. *Value Health* 2005;8:521-33.

17 Goetghebeur MM, Wagner M, Khoury H, Levitt RJ, Erickson LJ, Rindress D. Evidence and value: impact on decisionmaking—the EVIDEM framework and potential applications. *BMC Health Serv Rev* 2008;8:270.

18 Davis JC, Robertson MC, Comans T, Scuffham PA. Guidelines for conducting and reporting economic evaluation of fall prevention strategies. *Osteoporos Int* 2010;22:2449-59.

19 Petrou S, Gray A. Economic evaluation using decision analytical modelling: design, conduct, analysis, and reporting. *BMJ* 2011;342:d1766.

20 Petrou S, Gray A. Economic evaluation alongside randomised controlled trials: design, conduct, analysis, and reporting. *BMJ* 2011;342:d1548.

21 Husereau D, Drummond M, Petrou S, Carswell C, Moher D, Greenberg D, et al. Consolidated Health Economic Evaluation Reporting Standards (CHEERS)—explanation and elaboration: a report of the ISPOR Health Economic Evaluations Publication Guidelines Task Force. *Value in Health* (forthcoming).

22 Moher D, Schulz KF, Simera I, Altman DG. Guidance for developers of health research reporting guidelines. *PLoS Med* 2010;7:e1000217.

23 Moher D, Simera I, Schulz K, Miller D, Grimshaw J, Hoey J, et al. Reporting guidelines for clinical research: a systematic review. Vancouver, BC, 2009. Available at: www.ama-assn.org/public/peer/abstracts-0912.pdf.

24 Campbell SM, Hann M, Roland MO, Quayle JA, Shekelle PG. The effect of panel membership and feedback on ratings in a two-round Delphi survey: results of a randomized controlled trial. *Med Care* 1999;37:964-8

Cite this as: *BMJ* 2013;346:f1049

This is an open-access article distributed under the terms of the Creative Commons Attribution Non-commercial License, which permits use, distribution, and reproduction in any medium, provided the original work is properly cited, the use is non commercial and is otherwise in compliance with the license. See: http://creativecommons.org/licenses/by-nc/2.0/ and http://creativecommons.org/licenses/by-nc/2.0/legalcode.

No commercial reuse: See rights and reprints http://www.bmj.com/permissions Subscribe: http://www.bmj.com/subscribe

BMJ 2013;346:f1049 doi: 10.1136/bmj.f1049 (Published 25 March 2013)

Table

Table 1| **CHEERS checklist—Items to include when reporting economic evaluations of health interventions**

Section/item	Item No	Recommendation	Reported on page No/ line No
**Title and abstract**			
Title	1	Identify the study as an economic evaluation or use more specific terms such as "cost-effectiveness analysis", and describe the interventions compared.	1
Abstract	2	Provide a structured summary of objectives, perspective, setting, methods (including study design and inputs), results (including base case and uncertainty analyses), and conclusions.	23
**Introduction**			
Background and objectives	3	Provide an explicit statement of the broader context for the study.	102-133
		Present the study question and its relevance for health policy or practice decisions.	121-133
**Methods**			
Target population and subgroups	4	Describe characteristics of the base case population and subgroups analysed, including why they were chosen.	102-109
Setting and location	5	State relevant aspects of the system(s) in which the decision(s) need(s) to be made.	104-107
Study perspective	6	Describe the perspective of the study and relate this to the costs being evaluated.	126
Comparators	7	Describe the interventions or strategies being compared and state why they were chosen.	121-122
Time horizon	8	State the time horizon(s) over which costs and consequences are being evaluated and say why appropriate.	129-130
Discount rate	9	Report the choice of discount rate(s) used for costs and outcomes and say why appropriate.	132-133
Choice of health outcomes	10	Describe what outcomes were used as the measure(s) of benefit in the evaluation and their relevance for the type of analysis performed.	174
Measurement of effectiveness	11a	*Single study-based estimates:* Describe fully the design features of the single effectiveness study and why the single study was a sufficient source of clinical effectiveness data.	174-183
	11b	*Synthesis-based estimates:* Describe fully the methods used for identification of included studies and synthesis of clinical effectiveness data.	N/A
Measurement and valuation of preference based outcomes	12	If applicable, describe the population and methods used to elicit preferences for outcomes.	N/A 174.
Estimating resources and costs	13a	*Single study-based economic evaluation:* Describe approaches used to estimate resource use associated with the alternative interventions. Describe primary or secondary research methods for valuing each resource item in terms of its unit cost. Describe any adjustments made to approximate to opportunity costs.	135-149
	13b	*Model-based economic evaluation:* Describe approaches and data sources used to estimate resource use associated with model health states. Describe primary or secondary research methods for valuing each resource item in terms of its unit cost. Describe any adjustments made to approximate to opportunity costs.	N/A
Currency, price date, and conversion	14	Report the dates of the estimated resource quantities and unit costs. Describe methods for adjusting estimated unit costs to the year of reported costs if necessary. Describe methods for converting costs into a common currency base and the exchange rate.	142.
Choice of model	15	Describe and give reasons for the specific type of decision-analytical model used. Providing a figure to show model structure is strongly recommended.	N/A
Assumptions	16	Describe all structural or other assumptions underpinning the decision-analytical model.	
Analytical methods	17	Describe all analytical methods supporting the evaluation. This could include methods for dealing with skewed, missing, or censored data; extrapolation methods; methods for pooling data; approaches to validate or make adjustments (such as half cycle corrections) to a model; and methods for handling population heterogeneity and uncertainty.	186-221
**Results**			
Study parameters	18	Report the values, ranges, references, and, if used, probability distributions for all parameters. Report reasons or sources for distributions used to represent uncertainty where appropriate. Providing a table to show the input values is strongly recommended.	Table 4
Incremental costs and outcomes	19	For each intervention, report mean values for the main categories of estimated costs and outcomes of interest, as well as mean differences between the comparator groups. If applicable, report incremental cost-effectiveness ratios.	254-259 241-244.
Characterising uncertainty	20a	*Single study-based economic evaluation:* Describe the effects of sampling uncertainty for the estimated incremental cost and incremental effectiveness parameters, together with the impact of methodological assumptions (such as discount rate, study perspective).	278-290

No commercial reuse: See rights and reprints http://www.bmj.com/permissions Subscribe: http://www.bmj.com/subscribe

BMJ 2013;346:f1049 doi: 10.1136/bmj.f1049 (Published 25 March 2013)

(continued)

Section/item	Item No	Recommendation	Reported on page No/ line No
	20b	*Model-based economic evaluation:* Describe the effects on the results of uncertainty for all input parameters, and uncertainty related to the structure of the model and assumptions.	N/A
Characterising heterogeneity	21	If applicable, report differences in costs, outcomes, or cost-effectiveness that can be explained by variations between subgroups of patients with different baseline characteristics or other observed variability in effects that are not reducible by more information.	217-219
**Discussion**			
Study findings, limitations, generalisability, and current knowledge	22	Summarise key study findings and describe how they support the conclusions reached. Discuss limitations and the generalisability of the findings and how the findings fit with current knowledge.	292-296 321-377
**Other**			
Source of funding	23	Describe how the study was funded and the role of the funder in the identification, design, conduct, and reporting of the analysis. Describe other non-monetary sources of support.	397-401
Conflicts of interest	24	Describe any potential for conflict of interest of study contributors in accordance with journal policy. In the absence of a journal policy, we recommend authors comply with International Committee of Medical Journal Editors recommendations.	406-408

For consistency, the CHEERS statement checklist format is based on the format of the CONSORT statement checklist

No commercial reuse: See rights and reprints http://www.bmj.com/permissions Subscribe: http://www.bmj.com/subscribe
